# Comparison of ChatGPT and DeepSeek on a Standardized Audiologist Qualification Examination in Chinese: Observational Study

**DOI:** 10.2196/79534

**Published:** 2025-11-28

**Authors:** Beier Qi, Yan Zheng, Yuanyuan Wang, Li Xu

**Affiliations:** 1 Beijing Tongren Hospital, Capital Medical University, Key Laboratory of Otolaryngology - Head and Neck Surgery (Capital Medical University), Ministry of Education, Beijing, China Beijing China; 2 Department of Hearing, Speech and Language Sciences Ohio University Athens, OH United States

**Keywords:** generative artificial intelligence, artificial intelligence, AI, ChatGPT, DeepSeek, audiology, medical education

## Abstract

**Background:**

Generative artificial intelligence (GenAI), exemplified by ChatGPT and DeepSeek, is rapidly advancing and reshaping human-computer interaction with its growing reasoning capabilities and broad applications across fields such as medicine and education.

**Objective:**

This study aimed to evaluate the performance of 2 GenAI models (ie, GPT-4-turbo and DeepSeek-R1) on a standardized audiologist qualification examination in Chinese and to explore their potential applicability in audiology education and clinical training.

**Methods:**

The 2024 Taiwan Audiologist Qualification Examination, comprising 300 multiple-choice questions across 6 subject areas (ie, basic hearing science, behavioral audiology, electrophysiological audiology, principles and practice of hearing devices, health and rehabilitation of the auditory and balance systems, and hearing and speech communication disorders [including professional ethics]), was used to assess the performance of the 2 GenAI models. The complete answering process and reasoning paths of the models were recorded, and performance was analyzed by overall accuracy, subject-specific scores, and question-type scores. Statistical comparisons were performed at the item level using the McNemar test.

**Results:**

ChatGPT and DeepSeek achieved overall accuracies of 80.3% (241/300) and 79.3% (238/300), respectively, which are higher than the passing criterion of the Taiwan Audiologist Qualification Examination (ie, 60% correct answers). The accuracies for the 6 subject areas were 88% (44/50), 70% (35/50), 86% (43/50), 76% (38/50), 82% (41/50), and 80% (40/50) for ChatGPT and 82% (41/50), 72% (36/50), 78% (39/50), 80% (40/50), 80% (40/50), and 84% (41/50) for DeepSeek. No significant differences were found between the two models at the item level (overall *P*=.79), with a small effect size (accuracy difference=+1%, Cohen *h*=0.02, odds ratio 0.90, 95% CI 0.53–1.52) and substantial agreement (κ=0.71). ChatGPT scored highest in basic hearing science (88%), whereas DeepSeek performed the best in hearing and speech communication disorders (84%). Both models scored lowest in behavioral audiology (ChatGPT: 70% and DeepSeek: 72%). Question-type analysis revealed that both models performed well on reverse logic questions (ChatGPT: 79/95, 83%; DeepSeek: 80/95, 84%) but performed moderately on complex multiple-choice questions (ChatGPT: 9/17, 53%; DeepSeek: 11/17, 65%). However, both models performed poorly on graph-based questions (ChatGPT: 2/11, 18%; DeepSeek: 4/11, 36%).

**Conclusions:**

Both GenAI models demonstrated strong professional knowledge and stable reasoning ability, meeting the basic requirements of clinical audiologists and suggesting their potential as supportive tools in audiology education. However, the presence of errors underscores the need for cautious use under educator supervision. Future research should explore their performance in open-ended, real-world clinical scenarios to assess practical applicability and limitations.

## Introduction

Artificial intelligence (AI), first conceptualized in 1956 [1], has made remarkable progress over the past decade in simulating human cognition and behavior, driving breakthroughs across multiple fields [2]. By constructing brain-inspired computing architectures, AI facilitates automated analysis and decision-making in complex tasks [3]. In recent years, generative AI (GenAI, a subfield of AI that uses generative models to produce text, images, videos, or other forms of data), a leading subfield of AI, has rapidly advanced, driven by algorithmic innovations and the exponential growth of data. Powered by large-scale language models (a language model trained with self-supervised machine learning on a vast amount of text, designed for natural language processing tasks, especially language generation), GenAI has demonstrated broad application potential across medicine, education, scientific research, commerce, and industry [4]. As an evolving form of AI equipped with logical reasoning capabilities, GenAI is fundamentally reshaping the way humans interact with computers.

In the field of education, the leading models of GenAI such as ChatGPT (OpenAI), Gemini Ultra (Google), Meta Llama 3, Anthropic Claude 3, and DeepSeek (DeepSeek) can not only efficiently respond to professional questions and generate a clear problem-solving process but also simulate the thinking process of experts to assist students in academic exploration, problem-solving, and decision-making training [4-6]. GenAI’s exceptional generative capabilities offer new avenues for scholarly research, increased learning, classroom improvement, and knowledge sharing, and the interest in how these tools can benefit health education is also growing. Recent studies have shown that GenAI performs well in general medical examinations, such as the United States Medical Licensing Examination, the National Pharmacy Licensing Examination, and the National Nurse Licensing Examination, and has also demonstrated expert-level reasoning ability and diagnostic accuracy in the OTO Chautauqua Otolaryngology specialty examination [7-10]. As the quality and quantity of training data largely determine GenAI’s ability to understand and generate text, its outstanding performance in English contexts may not necessarily extend directly to non-English applications. Existing studies have also found that GenAI’s accuracy varies across different medical examinations and fields [11,12], with particularly inconsistent performance in examinations conducted in non–English-speaking regions [13,14]. Therefore, it is essential to further explore GenAI’s performance in processing non-English information, especially its application potential in Chinese, the most widely spoken first language (12.3%) and the second most spoken language (13.8%) in the world [15].

Audiology is the scientific study of hearing, balance, and related disorders. Audiologists are health care professionals who deliver patient-centered care focused on preventing, identifying, diagnosing, and treating hearing and balance disorders using evidence-based approaches. These conditions can have wide-ranging effects (ie, medical, psychological, physical, social, educational, and occupational). By offering expert and personalized care, audiologists help reduce the impact of these disorders, ultimately enhancing patients’ overall well-being and quality of life. In the United States, the doctor of audiology program is typically a 3- or 4-year graduate program. Doctor of audiology students are recommended to take the Praxis Examination in Audiology before their final year of their graduate study. Passing the Praxis Examination is a requirement for the American Speech-Language-Hearing Association Certificate of Clinical Competence in Audiology and state professional licensure in almost all 50 states in the United States [16]. The Educational Testing Service is responsible for administering the Praxis Examination, and the questions in the examination are not published. In Taiwan, an equivalent examination to the Praxis Examination, titled the Taiwan Audiologist Qualification Examination (TAQE), is published online every year [17]. The TAQE is written in traditional Chinese.

Previous studies have begun to explore the feasibility of GenAI in audiology education. Wang et al [18] evaluated the performance of ChatGPT (GPT-4) on the 2023 version of TAQE, showing an overall accuracy of 75%. This study suggests that GenAI possesses a certain level of professional knowledge in audiology and has the potential to support teaching and information acquisition. However, its abilities are still limited by the accuracy and scope of its knowledge, and its feasibility needs further verification.

In this study, the 2024 version of the TAQE was used to compare the performance of GPT-4-turbo and DeepSeek-R1 in audiology professional examinations. We aimed to gain insights into the potential of GenAI models in understanding audiology questions written in Chinese and to assess their applicability in audiology education and clinical practice. At the model level, compared with GPT-4 used by Wang et al [18], GPT-4-turbo demonstrates enhanced inference efficiency and cost-effectiveness, making it more suitable for practical applications. On the other hand, DeepSeek, a Chinese-developed GenAI, is optimized for Chinese language understanding and generation, thereby enhancing its adaptability and effectiveness in Chinese contexts. In addition, it adopts a reinforcement learning framework and incorporates a series of distilled models, which maintain performance while enabling efficient deployment in resource-constrained environments [19].

On the basis of this background, we propose 2 research hypotheses:

- The primary hypothesis is that GenAI models (GPT-4-turbo and DeepSeek-R1) are capable of achieving passing-level performance on the TAQE, thereby reaching the knowledge structure and reasoning ability required of clinical audiologists and demonstrating their potential applicability in audiology education and clinical training.
- The secondary hypothesis is that compared with the general-purpose model (GPT-4-turbo), DeepSeek-R1 is expected to achieve superior performance in the TAQE, reflecting the advantages of language-specific optimization.

By comparing ChatGPT and DeepSeek, this study not only highlights the technological strengths of both models but also enables a comprehensive evaluation of these 2 leading GenAI systems in the context of professional examinations written in Chinese, thereby shedding light on whether language-specific models (eg, DeepSeek) are superior to general models (eg, ChatGPT).

## Methods

### Ethical Considerations

This study does not involve human participants and is therefore classified as a non–human subject study. As such, it does not require institutional review board approval or informed consent. All data used in this research were derived from publicly available question banks, and the analysis was conducted exclusively using AI models. The study strictly adheres to data use guidelines and ensures full transparency in model implementation and use.

### AI Models

Two GenAI models were used in this study: GPT-4-turbo (released in April 2024) and DeepSeek-R1 (released in May 2024). GPT-4-turbo offers enhanced efficiency, a 128K-token context, and multimodal input [20]. DeepSeek-R1 provides optimized code and mathematical reasoning, a 128K-token context, and open weights for reproducibility. Although newer versions are available, these versions were the latest available GenAI models at the time of this research.

### Test Materials

The test questions were from the officially released version of the 2024 TAQE [17]. The TAQE is a professional licensing examination for audiologists and covers six subject areas: (1) basic hearing science, (2) behavioral audiology, (3) electrophysiological audiology, (4) principles and practice of hearing devices, (5) health and rehabilitation of the auditory and balance systems, and (6) hearing and speech communication disorders (including professional ethics), with 50 questions in each subject area, for a total of 300 questions. All questions were in a multiple-choice format. The examination was written in traditional Chinese. All questions and answer sheets were downloaded from the official website [17] as a PDF document.

### Procedure

**Figure 1:**
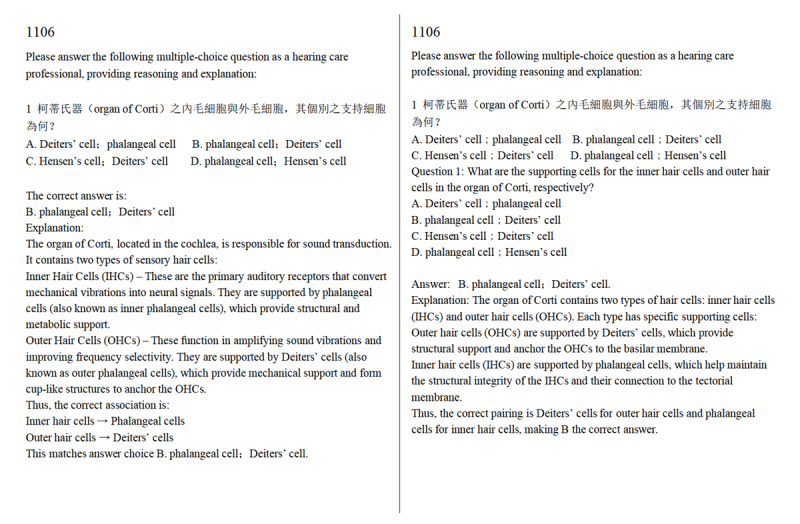
Examples of prompts and responses. Left: ChatGPT; right: DeepSeek. This example shows the first question from the basic auditory science examination. All Taiwan Audiologist Qualification Examination questions are multiple choice. The original examination questions are primarily in traditional Chinese and use bilingual (Chinese and English) terminology to express professional concepts.

We manually entered questions and options into the dialogue interface and recorded the model’s original responses, explanations, and answers. A fixed prompt, “Please answer the following multiple-choice question as a hearing care professional, providing reasoning and explanation,” was used to regulate the response style of the models and ensure the outputs were professionally relevant and interpretable (Figure 1). To ensure consistency and minimize potential human bias in the input process, all prompts were predetermined and entered by copy-and-paste rather than manual typing.

For graph-based questions, both the graph and the text were provided. Although the original test questions were in traditional Chinese, certain scientific terms (eg, the organ of Corti) were expressed in both Chinese and English. Both GenAI models were instructed to respond in English to minimize the impact of language differences. Additionally, the source of the test questions was concealed before testing to prevent the models from drawing on specific training data or external knowledge sources. ChatGPT completed all tests first (February 3-13, 2025), followed by DeepSeek (February 14-24, 2025).

To facilitate detailed error and reasoning analysis, the original questions were classified into 4 categories: graph based, complex, reverse logic, and standard (Table 1).

**Table 1:** Description of question categories.

Category	Definition in this study	Questions, n (%)	Example
Graph-based questions	Graph in the question or answer	11 (3.7)	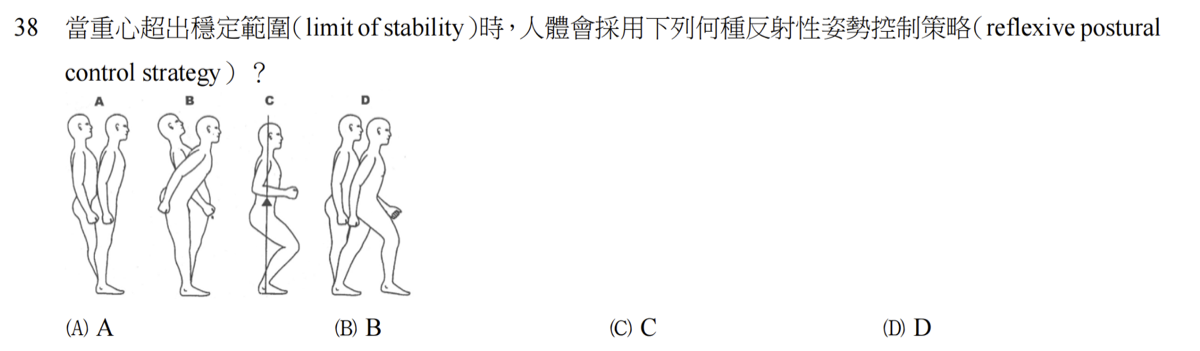
Complex multiple-choice questions	Each option consists of multiple knowledge points	17 (5.7)	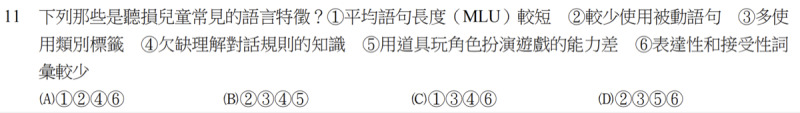
Reverse logic questions	Negative expressions in the questions, such as “wrong,” “avoid,” “inappropriate,” or “not including”	95 (31.7)	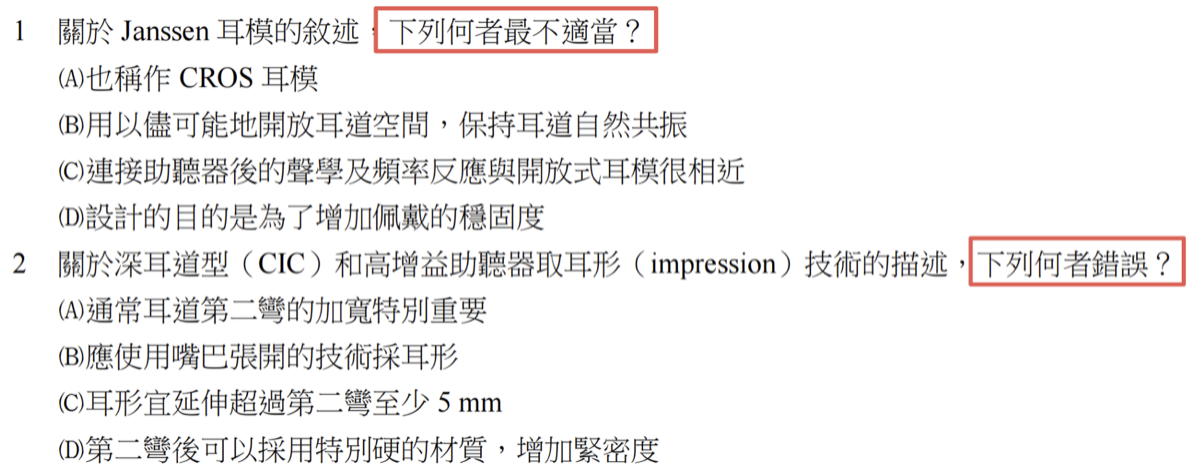
Standard multiple-choice questions	Others	177 (59)	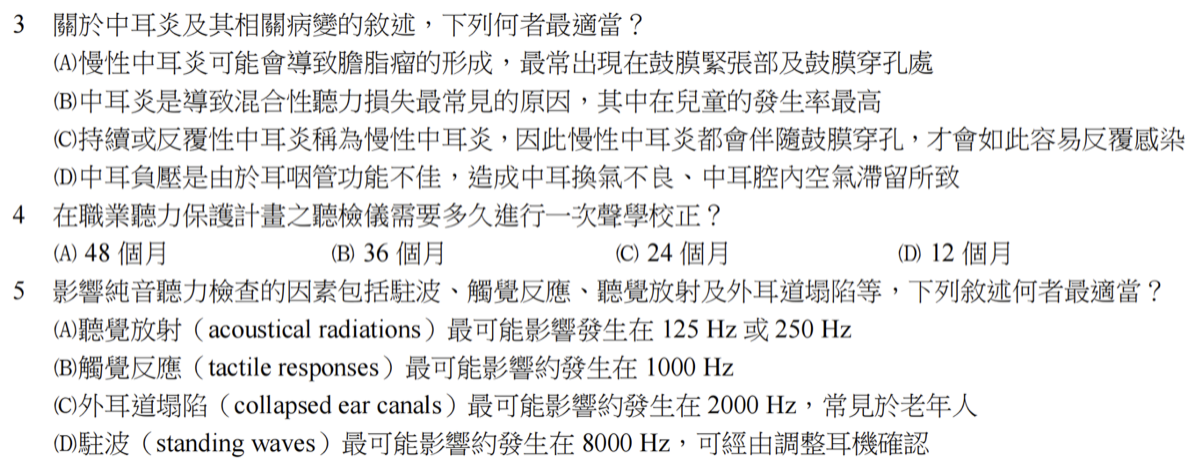

This study categorized the original questions into 4 categories, namely, diagram-based questions, complex multiple-choice questions, reverse logic questions, and standard multiple-choice questions, for detailed analysis. Table 1 provides definitions, percentages, and representative examples to illustrate the visual and conceptual differences between the different question types.

### Statistical Methods

Descriptive statistics were performed overall and by subject areas. Model performance was compared at the item level. The McNemar test was used to assess whether the 2 models differed in accuracy on the same items. Effect sizes were expressed as absolute differences in accuracy, odds ratios, and Cohen *h*. Kappa tests were used to test consistency. Statistical significance was set at *P*<.05. All analyses were performed using R (version 4.2.1).

## Results

**Figure 2:**
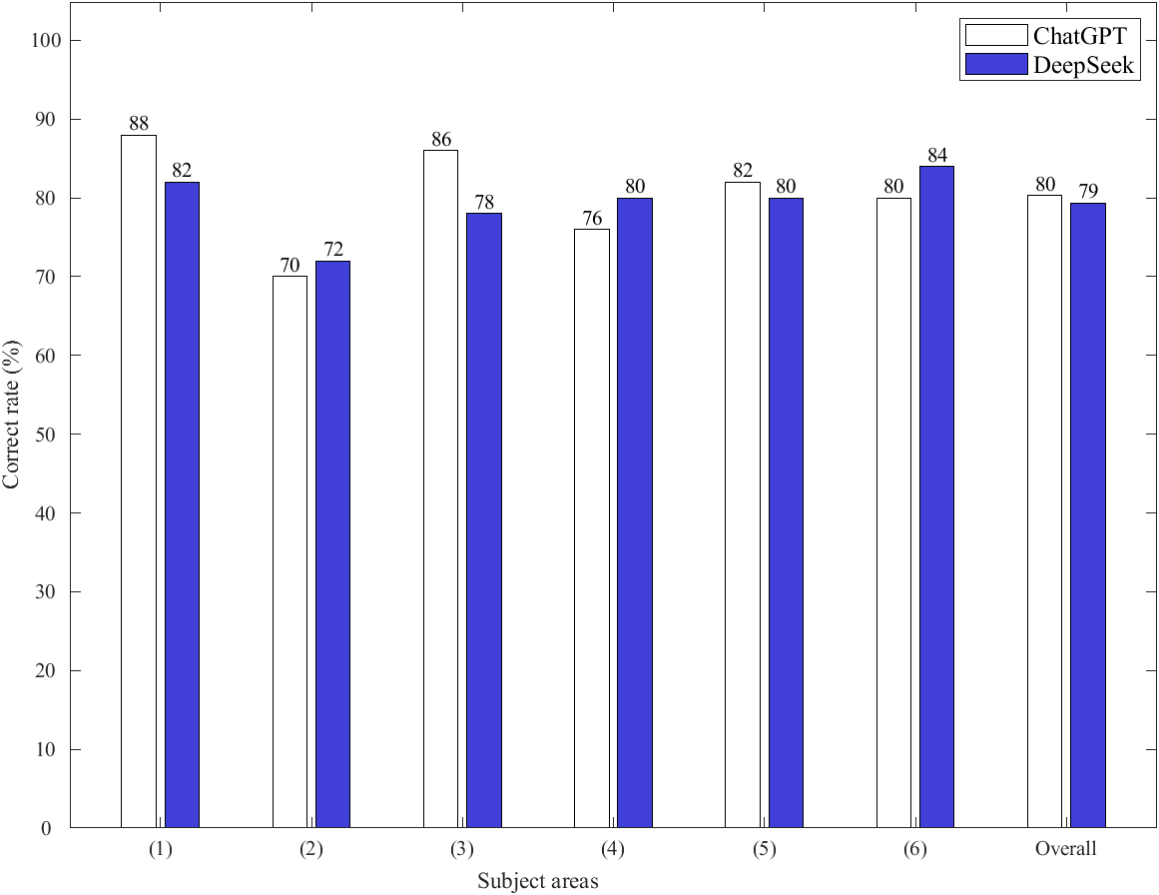
Performance on the 2024 Taiwan Audiologist Qualification Examination by ChatGPT and DeepSeek in the 6 subject areas (ie, basic hearing science, behavioral audiology, electrophysiological audiology, principles and practice of hearing devices, health and rehabilitation of the auditory and balance systems, and hearing and speech communication disorders [including professional ethics]) and the overall scores.

Both GenAI models achieved test scores exceeding the qualification examination’s passing criterion of 60% accuracy, demonstrating overall strong performance. ChatGPT attained an overall score of 80%, with subject-specific scores ranging from 70% to 88%. DeepSeek achieved an overall score of 79%, with subject-specific scores ranging from 72% to 84%, as shown in Figure 2. McNemar test, applied at the item level within each of the 6 subject areas (50 questions each) and in the pooled analysis across all 300 questions, showed no significant differences in accuracy between the 2 models (*P*=.34-.99; overall *P*=.79). The overall effect size was small (accuracy difference=+1%; Cohen *h*=0.02; odds ratio 0.90, 95% CI 0.53–1.52). Cohen κ indicated substantial agreement between the 2 models’ responses (κ=0.71, range 0.56-0.78), as presented in Table 2. These results indicate that the 2 models exhibited substantial response consistency despite minor fluctuations, suggesting highly similar overall capabilities in the audiologist qualification examination.

**Table 2:** Model capability comparison between ChatGPT and DeepSeek.

Subject area	GPT_ACC^a^	DS_ACC^b^	ACC_diff^c^	Cohen *h*	OR^d^	McNemar *P* value	κ
S1	0.88	0.82	+0.06	0.17	0.40 (0.08–2.06)	.45	0.78
S2	0.70	0.72	−0.02	−0.04	1.17 (0.39–3.47)	.99	0.56
S3	0.86	0.78	+0.08	0.21	0.43 (0.11–1.66)	.34	0.67
S4	0.76	0.80	−0.04	−0.10	2.00 (0.37–10.92)	.69	0.76
S5	0.82	0.80	+0.02	0.05	0.80 (0.21–2.98)	.99	0.72
S6	0.80	0.84	−0.04	−0.10	1.50 (0.42–5.32)	.75	0.70
Overall	0.803	0.793	+0.01	0.02	0.90 (0.53–1.52)	.79	0.71

^a^GPT_ACC: accuracy for ChatGPT.

^b^DS_ACC: accuracy for DeepSeek.

^c^ACC_diff: accuracy difference between GPT_ACC and DS_ACC.

^d^OR: odds ratio.

Subject-specific analysis showed that ChatGPT achieved its highest score in basic hearing science (88%), whereas DeepSeek performed best in hearing and speech communication disorders (including professional ethics; 84%). However, both models recorded their lowest scores in behavioral audiology, with ChatGPT at 70% and DeepSeek at 72% (Figure 2).

Question-type analysis revealed that both models underperformed on graph-based questions, with DeepSeek scoring 4 out of 11 (36% correct) and ChatGPT scoring 2 out of 11 (18% correct), both below the passing threshold. For complex multiple-choice questions, both approached the passing level, with DeepSeek scoring 11 out of 17 (65% correct) and ChatGPT scoring 9 out of 17 (53% correct). In reverse logic questions, both models performed well at comparable levels, with DeepSeek scoring 80 out of 95 (84% correct) and ChatGPT scoring 79 out of 95 (83% correct).

## Discussion

This study evaluated the performance of 2 GenAI models in a standardized audiologist qualification examination in Chinese. The results showed that (1) both GenAI models passed the qualification examination, indicating that they met the basic requirements of clinical audiologists and possessed the necessary audiological knowledge, and (2) the scores of the GenAI models varied across subjects, with the lowest performance in behavioral audiology. Performance also differed by question type: both models performed poorly on graph-based questions but showed good performance on reverse logic questions and (3) no overall advantage of DeepSeek over ChatGPT was observed, suggesting that language-specific optimization did not translate into a clear superiority in the audiology examination written in Chinese.

This study compared the performance of 2 GenAI models in a real audiologist qualification examination. This examination covers the core areas of audiology, fully reflects the breadth and depth of audiology, and is an important reference for evaluating audiology professional capabilities. The basic requirements for candidates include a degree in audiology or a related field and at least 6 months or 375 hours of clinical practice experience [21]. The passing criterion of the examination has been set at 60% correct, and the pass rate for the 2024 TAQE is 87.61%.

Our test program simulated the process of the candidate’s answer without external intervention. The overall accuracy of ChatGPT and DeepSeek were 80% and 79%, respectively. The results show that the 2 GenAI models can stably cope with the standardized (multiple-choice) audiology examination, have good audiology knowledge, and meet the basic standards for clinical audiologists. At the same time, the 2 GenAI models can provide a complete thinking process and clearly show the reasoning chain for solving problems, which makes them potentially useful in audiology education.

In this study, the performance of GenAI on the audiology examination (ie, a non-English examination) was similar to that of other professional examinations in Chinese, including the National Medical Qualification Examination, the National Pharmacist Qualification Examination, and the National Nursing Qualification Examination, with scores ranging from 73% to 84% using GPT-4.0 [10,22-24]. Although these scores are still lower than those of the English version, they are a significant improvement over GPT-3.5, indicating that GenAI is gradually adapting to the non-English examination environment.

In addition, Wang et al [18] evaluated the performance of GPT-4 on the 2023 TAQE, reporting an overall accuracy of 75%. In this study, testing on the 2024 TAQE, GPT-4-turbo (80% correct) and DeepSeek-R1 (79% correct) both outperformed the previous version of ChatGPT. Before concluding that improvements in the GenAI model led to better performance in audiology, we must consider that the difficulty level of the tests may differ between years. We reevaluated GPT-4-turbo on the 2023 TAQE and obtained a score of 76%, which was not significantly different from the score reported by Wang et al [18]. Thus, we do not have enough evidence to support the notion that the performance improvement is likely due to the improved GenAI model itself. To ensure comparability, this study followed the same approach as Wang et al [18], instructing the AI model to generate responses only in English and manually reviewing the accuracy of all outputs. Although no comprehension or accuracy issues attributable to bilingual processing were observed, potential bias arising from cross-lingual input and output should be carefully considered in future AI evaluation studies.

Both models scored lowest on behavioral audiology, likely due to the complexity of the questions that combine theoretical knowledge with clinical case analysis. For example, the basic knowledge questions are as follows:

Question 2. 聽力測試前進行耳鏡檢查之注意事項，下列何者錯誤?

Which of the following is incorrect regarding precautions for otoscopic examination before hearing tests?

Question 4. 在職業聽力保護計畫中，聽檢儀需多久進行一次聲學校正?

In a hearing conservation program, how often should the audiometer be acoustically calibrated?

Furthermore, the clinical case–based questions are as follows:

Question 18 針對雙側不對稱感音神經性聽力損失病人，根據語音辨識閾（SRT）及舒適聽閾推算遮蔽音設定.

For a patient with bilateral asymmetric sensorineural hearing loss, how should masking levels be set based on the speech recognition threshold (SRT) and the most comfortable listening level?

Question 24: 針對有非器質性聽損嫌疑的患者，從聽力檢查中辨別其表現特徵.

For a patient suspected of nonorganic hearing loss, how can audiological tests be used to identify characteristic patterns?

Compared to the structured questions relying on memorization or definitions, GenAI models are prone to reasoning biases when dealing with highly interactive and context-dependent questions. Additionally, the behavioral audiology contained the highest number of graph-based questions (5 graphs for 6 questions), which are the question types where both GenAI models performed the worst. These factors might contribute to the poor performance of both GenAI models in this module.

Meanwhile, the poor performance in graph-based questions is consistent with the findings of Wang et al [18] and may be partly due to the low image quality and resolution of the TAQE materials. These results suggest that current GenAI still relies on high-quality original images for effective graphical processing and also highlight limitations in multimodal processing, particularly in image-text integration and visual semantic understanding. However, the results of this study and Wang et al [18] were both lower than the 90.7% accuracy in the United States Medical Licensing Examination test reported by Yang et al [25]. This discrepancy may be attributed to language differences in the test materials [26,27]. In the first 2 studies, the stems of graph-based questions were presented in Chinese, whereas Yang et al [25] used English. Previous studies have shown that language effects are particularly evident in tasks requiring specific cultural background knowledge or relying on the latest literature and data in other languages [28], which may further account for the differences in model performance. In contrast, both GenAI models performed better on the reverse logic questions, demonstrating their strengths in causal reasoning and decision-support tasks.

DeepSeek performed on par with ChatGPT in the audiologist qualification examination written in Chinese (79% vs 80%). As a GenAI model optimized for the Chinese language, DeepSeek-R1 significantly increased its coverage of Chinese in the training corpus and adopted alignment strategies specifically tailored for Chinese during fine-tuning, making it more targeted in Chinese language applications [29].

One would expect that DeepSeek might demonstrate superior performance in professional tasks in Chinese contexts. Our results do not support such a conjecture. It is possible that a vast majority of literature and textbooks on audiology are written in English. The amount of text information on audiology written in Chinese is only a fraction of that in English. Therefore, optimized processing ability in Chinese would not benefit the overall performance of the GenAI in the field of audiology.

This study conducted a preliminary exploration of the application of GenAI in a standardized audiology test written in Chinese. A few limitations were noted. First, all test questions were multiple-choice questions, lacking open-ended questions. In actual clinical practice, audiologists need to not only master discrete knowledge points but also develop integrative reasoning skills to solve complex problems. Therefore, future evaluations should include open-ended questions to assess the ability of GenAI to handle actual clinical scenarios. Second, due to limited access to standardized audiology test materials, this study only examined the TAQE, whose content and format may differ from those used in other countries or regions. In addition, the official TAQE website only reports the annual number of examinees who passed and the overall passing rate, without providing category-specific performance data. These factors may limit the generalizability of the findings in a broader international context and prevent us from further comparing the performance differences between GenAI and human test takers across specific domains. Future studies with access to more granular category–level results would enable a more comprehensive evaluation of similarities and differences between GenAI and human candidates. Third, GenAI is a continuously evolving and updating tool. The versions used in this study may have been updated by the time of publication, so the results may not fully reflect the state-of-the-art performance of the GenAI models.

In conclusion, ChatGPT and DeepSeek demonstrated good professional knowledge and problem-solving ability in the standardized audiologist qualification examination. They also provide a complete reasoning process, enabling teachers and students to quickly grasp key concepts and thought processes, demonstrating their potential as supplementary tools for audiology education. Furthermore, AI’s superior ability to process massive data and accumulate knowledge allows it to be updated in real time, thus keeping pace with the evolving new technologies and methods in audiology, highlighting its value as a supportive tool for audiology education. However, GenAI responses still contain a certain error rate, and thus, their use in teaching requires careful judgment and guidance from educators. In addition, the performance of GenAI in open-ended tasks has not yet been fully validated, and its ability to foster humanistic qualities such as empathy and communication remains limited. Future research should prioritize incorporating open-ended tasks based on real-world scenarios to systematically evaluate the potential and limitations of generative AI in audiology education. Moreover, this study primarily focused on general-purpose models without fine-tuning or domain adaptation. It is reasonable to expect that domain-specific training could further enhance performance, which provides an important direction for future work.

## Data Availability

All data generated or analyzed during this study are included in this published paper and Multimedia Appendices 1-3.

## Appendix

Multimedia Appendix 1 ChatGPT transcripts.

Multimedia Appendix 2 DeepSeek transcripts.

Multimedia Appendix 3 Keys to the 2024 Taiwan Audiologist Qualification Examination.

## Abbreviations

AI: artificial intelligence
GenAI: generative artificial intelligence
TAQE: Taiwan Audiologist Qualification Examination
