# Supplementary material for: Comparison of ChatGPT and DeepSeek on a Standardized Audiologist Qualification Examination in Chinese: Observational Study
Source: JMIR Form Res. 2025 Nov 28;9:e79534. doi: 10.2196/79534 (PMC12701348; doi:10.2196/79534)
Supplement: Multimedia Appendix 1 [file formative_v9i1e79534_app1.pdf]

1106

Please answer the following multiple-choice question as a hearing care professional, providing reasoning and explanation:

1 柯蒂氏器（organ of Corti）之內毛細胞與外毛細胞，其個別之支持細胞為何？

A Deiters’ cell；phalangeal cell B phalangeal cell；Deiters’ cell

C Hensen’s cell；Deiters’ cell D phalangeal cell；Hensen’s cell

The correct answer is:

B. phalangeal cell；Deiters’ cell

Explanation:

The organ of Corti, located in the cochlea, is responsible for sound transduction. It contains two types of sensory hair cells:

Inner Hair Cells (IHCs) – These are the primary auditory receptors that convert mechanical vibrations into neural signals. They are supported by phalangeal cells (also known as inner phalangeal cells), which provide structural and metabolic support.

Outer Hair Cells (OHCs) – These function in amplifying sound vibrations and improving frequency selectivity. They are supported by Deiters’ cells (also known as outer phalangeal cells), which provide mechanical support and form cup-like structures to anchor the OHCs.

Thus, the correct association is:

Inner hair cells → Phalangeal cells

Outer hair cells → Deiters’ cells

This matches answer choice B. phalangeal cell；Deiters’ cell.

Question 2: About the Tympanic Membrane (Eardrum)

The incorrect statement is:

A. 鬆弛部（pars flaccida）由內而外有三層，分別為上皮層、纖維層、黏膜層

Explanation:

The tympanic membrane (eardrum) is a thin, cone-shaped membrane that separates the external ear from the middle ear and plays a key role in sound transmission. It consists of two main parts:

Pars Tensa (緊張部) – The larger, more rigid portion of the eardrum. It consists of three layers:

Outer epithelial layer (continuous with the external ear canal skin)

Middle fibrous layer (provides structural integrity)

Inner mucosal layer (continuous with the middle ear cavity lining)

Pars Flaccida (鬆弛部) – A smaller, more flexible region located at the upper part of the tympanic membrane. Unlike the pars tensa, it lacks a fibrous layer and consists only of two layers:

Outer epithelial layer

Inner mucosal layer

Since statement A incorrectly states that the pars flaccida has three layers (when it actually has only two), it is the incorrect statement.

Verification of Other Statements:

B. 鼓膜下方主要為緊張部（pars tensa） → ✅ Correct. The majority of the tympanic membrane is composed of the pars tensa.

C. 成人鼓膜面積大約 55～90 mm² → ✅ Correct. The surface area of the adult tympanic membrane is typically around 55-90 mm².

D. 整體呈一個錐狀體（cone-shaped），圓錐頂點朝向中耳方向 → ✅ Correct. The tympanic membrane is cone-shaped, with the apex (umbo) pointing inward toward the middle ear.

Thus, the incorrect statement is A.

Question 3: Interaural Time (Phase) Difference (IPD) and Sound Localization

The correct answer is:

A. 750 Hz

Explanation:

The human auditory system uses two primary binaural cues to localize sounds in the horizontal plane (azimuth localization):

Interaural Time Difference (ITD) or Interaural Phase Difference (IPD) – The difference in the arrival time of a sound between the two ears. This cue is effective for low-frequency sounds because low-frequency waves (longer wavelengths) can be compared for phase differences.

Interaural Level Difference (ILD) – The difference in sound intensity between the two ears due to the head shadow effect. This cue is effective for high-frequency sounds because high-frequency waves have shorter wavelengths and are more easily blocked by the head.

Research suggests that the IPD/ITD cue is most effective for sounds below approximately 1500 Hz, but it is particularly dominant for frequencies below 750 Hz.

A. 750 Hz → ✅ Correct. The central auditory system primarily relies on IPD for frequencies below approximately 750 Hz.

B. 1500 Hz → ❌ Higher than the typical range where phase differences are dominant.

C. 3000 Hz → ❌ At this frequency, the head shadow effect becomes more dominant, making interaural level differences (ILD) the primary cue.

D. 6000 Hz → ❌ High-frequency sounds rely almost entirely on ILD rather than ITD.

Thus, the correct answer is A. 750 Hz.

Question 4: Cochlear Sensitivity and Dynamic Range

The correct answer is:

B. 外毛細胞 (Outer Hair Cells, OHCs)

Explanation:

The cochlea is highly sensitive to different frequencies and has a wide dynamic range, allowing us to hear sounds from very soft to very loud. This is primarily due to the cochlear amplifier effect, which is mediated by outer hair cells (OHCs).

Outer Hair Cells (OHCs) actively enhance the movement of the basilar membrane through electromotility—they change length in response to sound, amplifying weaker signals and sharpening frequency selectivity. This enhances sensitivity and expands the dynamic range.

Inner Hair Cells (IHCs), while essential for transmitting sound signals to the auditory nerve, do not contribute to amplification.

Spiral Ganglion Cells (C) are neurons that transmit signals from the hair cells to the brain but do not enhance cochlear sensitivity.

Cochlear Nucleus Cells (D) are part of the auditory brainstem and process sound but do not directly contribute to the cochlear amplifier.

Thus, the correct answer is B. Outer Hair Cells.

Question 5: Characteristics of the Basilar Membrane

The correct answer is:

A. 僅①②④

Explanation:

The basilar membrane (BM) is responsible for frequency tuning in the cochlea. It varies in width, thickness, and stiffness along its length, which enables tonotopic organization (frequency mapping).

① 靠耳蝸頂部的基底膜比較寬 (The BM is wider at the apex of the cochlea) → ✅ Correct. The apical region (top) of the cochlea responds to low-frequency sounds and has a wider and more flexible basilar membrane.

② 靠耳蝸底部的基底膜比較窄 (The BM is narrower at the base of the cochlea) → ✅ Correct. The basal region (bottom) of the cochlea responds to high-frequency sounds and has a narrower and stiffer basilar membrane.

③ 靠耳蝸底部的基底膜比較厚 (The BM is thicker at the base of the cochlea) → ❌ Incorrect. The basilar membrane is actually thinner at the base and thicker at the apex.

④ 靠耳蝸頂部的基底膜張力較大 (The BM has greater tension at the apex of the cochlea) → ❌ Incorrect. The base of the basilar membrane is under more tension, making it stiffer, whereas the apex is more flexible.

Thus, the correct answer is A. 僅①②④.

Question 6: Muscles Involved in Eustachian Tube Opening

The correct answer is:

C. 僅①②④ (levator veli palatini, salpingopharyngeus, tensor veli palatini)

Explanation:

The Eustachian tube (auditory tube) connects the middle ear to the nasopharynx and helps equalize air pressure between the middle ear and the external environment. Its opening is controlled by specific muscles:

① Levator veli palatini → ✅ Correct. This muscle helps open the Eustachian tube by elevating the soft palate and exerting pressure on the tube.

② Salpingopharyngeus → ✅ Correct. This muscle assists in opening the Eustachian tube and helps elevate the pharynx during swallowing.

③ Tensor tympani → ❌ Incorrect. This muscle is involved in dampening loud sounds by pulling on the malleus but does not directly open the Eustachian tube.

④ Tensor veli palatini → ✅ Correct. This is the primary muscle responsible for actively opening the Eustachian tube during swallowing and yawning.

Since tensor tympani (③) is not involved in opening the Eustachian tube, the correct answer is C. 僅①②④.

Question 7: External and Middle Ear Characteristics

The correct answer is:

D. 耳膜為光滑（smooth）、半透明（translucent）的薄膜，平均厚度約 0.074mm，表面呈凹形，凹狀頂部稱為鼓膜臍（umbo）

Explanation:

Verification of Each Statement:

A. 外耳道平均寬度（width）約 9mm、高度（height）約 6.5 mm、長度（length）約 2.5 至 3.5 cm、呈 S 形，有兩個彎處 → ❌ Incorrect.

The external auditory canal is approximately 2.5 to 3.5 cm long, but the typical width is around 7 mm, not 9 mm.

It does have an S-shape with two bends, but the size discrepancy makes this option incorrect.

B. 外耳道與耳膜約呈 35 度的角度 → ❌ Incorrect.

The angle of the ear canal relative to the tympanic membrane varies among individuals but is closer to 45 degrees, not 35 degrees.

C. 耳道外側三分之二的部分其下方是軟骨，其餘部分則是硬骨 → ❌ Incorrect.

The external auditory canal's outer one-third is cartilaginous, while the inner two-thirds is bony. This statement incorrectly states the proportions.

D. 耳膜為光滑（smooth）、半透明（translucent）的薄膜，平均厚度約 0.074mm，表面呈凹形，凹狀頂部稱為鼓膜臍（umbo） → ✅ Correct.

The tympanic membrane (eardrum) is indeed:

Smooth and semi-transparent, allowing some light to pass through.

Approximately 0.074 mm thick.

Concave in shape, with the umbo as the central depressed point where the malleus attaches.

Thus, the correct answer is D.

Question 8: Factors Affecting Middle Ear Energy Transfer Efficiency

The correct answer is:

D. 傳遞頻率 (Transmission Frequency)

Explanation:

The middle ear functions as an impedance-matching system to efficiently transfer sound energy from the air-filled external ear to the fluid-filled cochlea. Several mechanical factors contribute to this efficiency:

A. 耳膜與圓窗面積比 (Tympanic Membrane to Oval Window Area Ratio) → ✅ Important Factor

The tympanic membrane is much larger than the oval window (about 17-20 times the area), which concentrates force onto a smaller surface, increasing pressure and improving energy transfer.

B. 耳膜屈面效應 (Curved Surface Effect of the Tympanic Membrane) → ✅ Important Factor

The concave shape of the tympanic membrane enhances sound transmission by improving vibration efficiency.

C. 聽小骨機械槓桿特性 (Ossicular Mechanical Lever Action) → ✅ Important Factor

The ossicles (malleus, incus, stapes) act as a lever system, increasing the force applied to the oval window by about 1.3 times, which helps in impedance matching.

D. 傳遞頻率 (Transmission Frequency) → ❌ Not a Major Factor

While different frequencies are transmitted differently due to cochlear mechanics, the middle ear's efficiency is primarily determined by mechanical structures rather than transmission frequency itself. The middle ear works most efficiently in the 500–4000 Hz range, but frequency itself is not a fundamental factor in impedance matching.

Thus, the correct answer is D. 傳遞頻率 because it is not a primary factor in middle ear energy transfer efficiency.

Question 9: Direction of Stereocilia Deflection Leading to Hair Cell Excitation

The correct answer is:

A. 耳蝸管外側 (Toward the Outer Side of the Cochlear Duct)

Explanation:

When the basilar membrane moves due to incoming sound waves, a shearing force occurs between the tectorial membrane and the stereocilia of hair cells in the Organ of Corti.**

The deflection of stereocilia determines whether the hair cell is excited (depolarized) or inhibited (hyperpolarized).

When the stereocilia bend toward the tallest stereocilium (kinocilium)—which is toward the outer side of the cochlear duct (A)—ion channels open, allowing K^+ and Ca^{2+} ions to enter, leading to hair cell excitation and neurotransmitter release.

If they bend in the opposite direction (toward the modiolus), ion channels close, leading to inhibition.

Thus, the correct answer is A. Toward the Outer Side of the Cochlear Duct because this direction triggers excitation in the hair cells.

Question 10: Nerve Crossing the Long Process of the Incus Below the Manubrium of the Malleus

The correct answer is:

D. 鼓索神經 (Chorda Tympani Nerve)

Explanation:

The chorda tympani nerve is a branch of the facial nerve (cranial nerve VII) that carries taste sensations from the anterior two-thirds of the tongue and parasympathetic fibers to the submandibular and sublingual salivary glands.

This nerve runs through the middle ear, crossing the long process of the incus (砧骨長柄) just below the manubrium of the malleus (鎚骨柄).

It does not contribute to hearing but is an important anatomical structure in ear surgeries.

Why the other options are incorrect:

A. 耳咽神經 (Glossopharyngeal Nerve, CN IX) → Incorrect; involved in pharyngeal sensation, not middle ear crossing.

B. 三叉神經 (Trigeminal Nerve, CN V) → Incorrect; provides sensation to the face but does not cross the middle ear ossicles.

C. 鐙骨肌神經 (Stapedius Nerve) → Incorrect; this is a branch of the facial nerve that controls the stapedius muscle but does not cross the incus.

Thus, the correct answer is D. 鼓索神經 (Chorda Tympani Nerve).

Question 11: Ion Concentration Differences Between Perilymph and Endolymph

The correct answer is:

B. 外淋巴為高鈉、低鉀，內淋巴為高鉀、低鈉

Explanation:

The cochlea contains two main types of fluids:

Perilymph (外淋巴液)

Found in the scala vestibuli and scala tympani

High sodium (Na^+), low potassium (K^+) → similar to extracellular fluid (ECF) or cerebrospinal fluid (CSF)

Important for maintaining ionic balance and supporting cochlear function

Endolymph (內淋巴液)

Found in the scala media

High potassium (K^+), low sodium (Na^+) → similar to intracellular fluid (ICF)

Essential for hair cell function, creating the endocochlear potential (~80-100 mV), which drives sensory transduction

Since endolymph is high in potassium and perilymph is high in sodium, the correct answer is:

✅ B. 外淋巴為高鈉、低鉀，內淋巴為高鉀、低鈉

Question 12: Mitochondrial Genetic Disorders

The correct answer is:

A. 主要是透過父親傳給下一代，與母親無關 (Incorrect statement)

Explanation:

Mitochondrial genetic disorders are exclusively inherited from the mother because mitochondria are transmitted through the cytoplasm of the egg cell. Sperm mitochondria are typically degraded after fertilization.

Why the other options are correct:

B. 人體細胞內有數百到數千個粒腺體，影響人類代謝需求較大的胞器 → ✅ Correct.

Each cell contains hundreds to thousands of mitochondria, which are essential for energy metabolism.

C. 臨床基因檢測可透過 MRTNR1 與 MTTS1 兩種測試 → ✅ Correct.

MRTNR1 and MTTS1 are common mitochondrial DNA (mtDNA) mutations linked to hearing loss.

D. 個體可能經胺基酸配醣體類抗生素（aminoglycoside）的暴露後，產生基因突變並引起感音性聽損 → ✅ Correct.

Individuals with m.1555A>G mutation in the MT-RNR1 gene are highly susceptible to aminoglycoside-induced ototoxicity, leading to sensorineural hearing loss.

Since mitochondrial disorders are inherited maternally (not paternally), the incorrect statement is:

❌ A. 主要是透過父親傳給下一代，與母親無關

Question 13: The most likely location for cochlear microphonic potential generation

Answer: B. Outer hair cells

Explanation:

Cochlear microphonic (CM) potential is an electrical potential generated in response to sound stimulation. It is primarily produced by the outer hair cells of the cochlea, as they play a major role in the mechanotransduction process, converting mechanical sound vibrations into electrical signals. Compared to inner hair cells, outer hair cells contribute more significantly to CM potential due to their electromotility and amplification functions. Therefore, the most likely site of CM potential generation is the outer hair cells.

Question 14: Recruitment phenomenon in hearing tests is most commonly observed in which condition?

Answer: B. Sensorineural hearing loss

Explanation:

Recruitment is a phenomenon in which soft sounds are not heard, but as the volume increases slightly, the sound suddenly seems much louder. This occurs due to damage to the outer hair cells in the cochlea, which affects their ability to amplify low-level sounds while still allowing louder sounds to be perceived. Sensorineural hearing loss (SNHL), particularly cochlear-related conditions such as presbycusis (age-related hearing loss) or noise-induced hearing loss, is most commonly associated with recruitment.

In contrast:

A. Auditory neuropathy and D. Neural hearing loss primarily affect the auditory nerve, leading to poor speech discrimination but not recruitment.

C. Conductive hearing loss results from outer or middle ear issues that block sound transmission, but since the inner ear remains functional, recruitment is not typically observed.

Question 15: Which is NOT included in Borg et al.'s desensitization-interference-injury protection theory regarding the purpose of the acoustic reflex?

Answer: D. Changing inner ear labyrinthine pressure to enhance high-frequency information reception

Explanation:

Borg et al. proposed that the acoustic reflex serves multiple functions, including:

Desensitization: Reducing sensitivity to self-generated sounds, such as chewing or speaking (Option A).

Interference reduction: Filtering out low-frequency components of speech to enhance high-frequency information perception (Option B).

Injury protection: Protecting the inner ear from loud sounds by reducing excessive sound transmission (Option C).

However, Option D is incorrect because the acoustic reflex does not function by altering labyrinthine pressure to enhance high-frequency sound perception. Instead, it primarily involves the contraction of the stapedius muscle, which stiffens the ossicular chain, thereby reducing the transmission of loud sounds.

Question 16: Which structure has the highest selectivity for tuning to a narrow range of interaural time differences (ITD)?

Answer: C. Inferior colliculus (IC)

Explanation:

Interaural time difference (ITD) is a crucial cue for localizing low-frequency sounds. The auditory pathway processes ITD in multiple brainstem and midbrain structures:

Cochlear nucleus (A): Primarily involved in early auditory signal processing, but not highly specialized for ITD tuning.

Superior olivary complex (SOC) (B): A key structure for ITD processing, particularly the medial superior olive (MSO), which integrates binaural time differences.

Inferior colliculus (IC) (C): This midbrain structure refines ITD processing with higher selectivity, allowing for fine-tuned localization of narrow ITD ranges. It integrates signals from the SOC and further enhances spatial resolution.

Medial geniculate body (MGB) (D): A thalamic relay station that processes auditory information before it reaches the auditory cortex but is less specialized for ITD tuning.

Thus, the inferior colliculus (IC) exhibits the most selective ITD tuning, allowing precise encoding of spatial sound information.

Question 17: Which statement about the superior olivary complex (SOC) is incorrect?

Answer: C. Only about 5% of SOC neurons receive excitatory input from one side and inhibitory input from the other.

Explanation:

The superior olivary complex (SOC) is a critical brainstem structure involved in processing binaural auditory information, particularly for sound localization.

A. Correct: The SOC is the first (lowest) structure in the auditory pathway to receive bilateral auditory input, making it essential for interaural time and level difference processing.

B. Correct: The majority of SOC neurons receive input from both ears, allowing them to process spatial hearing cues.

C. Incorrect: More than 5% of SOC neurons exhibit an excitatory-inhibitory (EI) response pattern, particularly in the lateral superior olive (LSO), which helps process interaural level differences (ILD). The actual percentage is much higher.

D. Correct: Some neurons in the SOC, particularly in the medial superior olive (MSO), respond primarily to contralateral sound stimuli, playing a role in interaural time difference (ITD) processing.

Thus, option C is incorrect because the proportion of EI-type neurons in the SOC is significantly higher than 5%.

Question 18: The anterior ventral cochlear nucleus (AVCN) primarily projects to which structure to process binaural sound and calculate sound localization?

Answer: A. Medial superior olive (MSO)

Explanation:

The anterior ventral cochlear nucleus (AVCN) is an important relay station in the auditory pathway that sends outputs to various auditory brainstem structures.

A. Correct: The AVCN projects to the medial superior olive (MSO), which is specialized in processing interaural time differences (ITD) for sound localization in low frequencies.

B. Incorrect: The lateral superior olive (LSO) primarily processes interaural level differences (ILD) for high-frequency sound localization and receives input from both AVCN and other nuclei.

C. Incorrect: The lateral lemniscus (LL) is an ascending auditory pathway but does not perform the direct ITD processing needed for localization.

D. Incorrect: The inferior colliculus (IC) is a midbrain structure that integrates auditory information but is not the primary target of the AVCN for sound localization.

Thus, the AVCN mainly projects to the MSO, where interaural time differences are processed to determine the location of a sound source.

Question 19: Which statement about binaural hearing is incorrect?

Answer: B. The periolivary nuclei primarily receive high-frequency input and interaural level difference (ILD).

Explanation:

Binaural hearing enables sound localization and spatial awareness by utilizing interaural time difference (ITD) for low frequencies and interaural level difference (ILD) for high frequencies. The superior olivary complex (SOC) and related brainstem nuclei play key roles in processing these cues.

A. Correct: The SOC integrates excitatory and inhibitory inputs from both ears, which is critical for sound localization. The medial superior olive (MSO) primarily processes ITD, while the lateral superior olive (LSO) processes ILD.

B. Incorrect: The periolivary nuclei are part of the SOC but do not primarily process high-frequency inputs and ILD. Instead, they are involved in the medial olivocochlear reflex, which controls cochlear outer hair cells and helps with adaptive hearing functions such as noise suppression.

C. Correct: The medial superior olive (MSO) is responsible for low-frequency sound processing and ITD, helping to determine where a sound originates based on the time delay between ears.

D. Correct: The dorsal nucleus of the lateral lemniscus (LL) responds to both interaural time and level differences, helping refine sound localization cues before they reach the inferior colliculus.

Thus, option B is incorrect because the periolivary nuclei do not primarily process ILD and high-frequency inputs; rather, they contribute to efferent auditory control.

Question 20: Which statement about binaural auditory encoding in the superior olivary complex is correct?

Answer: A. The medial superior olive (MSO) primarily receives low-frequency input and interaural time difference (ITD).

Explanation:

The superior olivary complex (SOC) processes binaural auditory information, with two main nuclei specializing in different localization cues:

A. Correct: The MSO processes low-frequency sounds and ITD, as longer wavelengths allow for phase differences between ears to be a reliable localization cue.

B. Incorrect: The LSO primarily processes ILD, not ITD, and is more sensitive to high-frequency sounds.

C. Incorrect: The MSO does not process high-frequency sounds or ILD; it is specialized for low-frequency ITD processing.

D. Incorrect: The LSO processes high-frequency sounds but uses ILD, not ITD, because high-frequency sounds have shorter wavelengths, making ITD unreliable at these frequencies.

Thus, option A is the correct answer, as the MSO is the primary nucleus for processing low-frequency ITD cues in binaural hearing.

Question 21: Which of the following is NOT a component of the cochlear nuclei?

Answer: B. Medial cochlear nucleus

Explanation:

The cochlear nuclei are the first relay stations in the auditory pathway, located in the brainstem. They receive direct input from the auditory nerve and are divided into three main components:

A. Anterior ventral cochlear nucleus (AVCN): Processes timing and intensity cues, contributing to sound localization.

C. Dorsal cochlear nucleus (DCN): Involved in complex sound processing, including spectral analysis.

D. Posterior ventral cochlear nucleus (PVCN): Assists in encoding sound intensity and timing.

There is no "medial cochlear nucleus" in the auditory system, making option B incorrect.

Question 22: Based on animal studies, bilateral auditory cortex lesions would impair which auditory ability?

Answer: C. Perceiving changes in frequency

Explanation:

The auditory cortex plays a crucial role in processing complex auditory features, including frequency discrimination, duration perception, and sound localization. Animal studies show that bilateral auditory cortex damage leads to significant impairments in frequency resolution.

A. Incorrect: Basic intensity perception occurs at lower levels of the auditory pathway (e.g., cochlear nucleus, inferior colliculus), so cortical lesions do not significantly impact loudness perception.

B. Incorrect: The onset of sound is primarily processed at brainstem levels (e.g., cochlear nucleus, superior olivary complex), so the auditory cortex is not essential for detecting sound onset.

C. Correct: The auditory cortex, particularly in the primary auditory area (A1), is crucial for processing frequency changes. Lesions in this area cause difficulty in distinguishing pitch variations.

D. Incorrect: While the auditory cortex contributes to sound duration perception, studies suggest that frequency discrimination is more affected by bilateral cortical lesions.

Thus, option C is correct, as the auditory cortex is essential for perceiving frequency changes.

Question 23: Which nerve is NOT located within the internal auditory meatus?

Answer: A. Trigeminal nerve (CN V)

Explanation:

The internal auditory meatus (IAM) is a canal in the temporal bone that transmits certain cranial nerves from the brainstem to the inner ear and face. The nerves passing through the IAM include:

B. Facial nerve (CN VII): Travels through the IAM before exiting at the stylomastoid foramen.

C. Cochlear nerve (a branch of CN VIII): Carries auditory signals from the cochlea to the brainstem.

D. Vestibular nerve (a branch of CN VIII): Transmits balance-related information from the vestibular system.

The trigeminal nerve (CN V) does NOT pass through the IAM. Instead, it exits the brainstem at the pons and travels through different foramina:

Ophthalmic branch (V1) → Superior orbital fissure

Maxillary branch (V2) → Foramen rotundum

Mandibular branch (V3) → Foramen ovale

Thus, option A is correct, as the trigeminal nerve is NOT located within the internal auditory meatus.

Question 24: Which statement about the composition of the superior olivary complex (SOC) is incorrect?

Answer: B. Dorsal superior olive

Explanation:

The superior olivary complex (SOC) is a critical brainstem structure involved in binaural hearing and sound localization. It consists of several nuclei, including:

A. Lateral superior olive (LSO): Processes interaural level differences (ILD) for high-frequency sound localization.

C. Trapezoid body: Contains crossing fibers from the cochlear nucleus to the SOC and plays a role in sound localization.

D. Preolivary nuclei: Small nuclei surrounding the main SOC structures, involved in auditory processing.

However, there is no "dorsal superior olive" as a recognized part of the SOC. The SOC consists of medial and lateral superior olivary nuclei, but no distinct "dorsal" subdivision.

Thus, option B is incorrect, as the dorsal superior olive does not exist as a recognized anatomical structure in the SOC.

Question 25: When two sine waves have completely opposite displacements at any instant in time, what is their starting phase difference?

Answer: C. 180°

Explanation:

A sine wave's phase represents its position in its cycle at a given point in time. If two sine waves are exact opposites (i.e., when one wave is at a peak, the other is at a trough, and vice versa), their phase difference is:

0° (Option A): The waves are identical (in phase).

90° (Option B): The waves are a quarter-cycle apart, meaning they are out of sync but not completely opposite.

180° (Option C): The waves are completely out of phase (i.e., they cancel each other out when summed).

270° (Option D): The waves are three-quarters out of phase but not directly opposite.

Thus, the correct answer is 180°, as two sine waves that are exact opposites in displacement have a phase difference of 180°.

Question 26: How does the length of inner hair cell (IHC) stereocilia change from the cochlear basal turn to the apical turn?

Answer: A. Gradually increases

Explanation:

The inner hair cells (IHCs) are sensory cells in the cochlea responsible for converting sound vibrations into neural signals. The stereocilia on these cells vary in length along the cochlea:

At the basal turn (near the oval window), the stereocilia are shorter, as this region responds to high-frequency sounds.

Moving toward the apical turn (at the cochlear tip), the stereocilia gradually lengthen, as this region responds to low-frequency sounds.

This gradual increase in stereocilia length supports the cochlear tonotopic organization, where different regions are tuned to specific frequencies.

Thus, the correct answer is A: The stereocilia gradually increase in length from the basal turn to the apical turn.

Question 27: Which neurotransmitter excites the central auditory nervous system?

Answer: C. Acetylcholine

Explanation:

In the central auditory pathway, neurotransmitters play a crucial role in either exciting or inhibiting neural activity.

A. Glutamine (Incorrect): Glutamine is a precursor to glutamate, which is excitatory. However, glutamine itself is not an excitatory neurotransmitter.

B. Epinephrine & Corticosteroid (Incorrect): These are not primary neurotransmitters in the auditory system; epinephrine functions more in the autonomic nervous system, and corticosteroids are involved in stress responses.

C. Acetylcholine (Correct): Acetylcholine acts as an excitatory neurotransmitter in various auditory pathways, including the medial olivocochlear system, which helps modulate auditory sensitivity.

D. GABA (Incorrect): Gamma-aminobutyric acid (GABA) is an inhibitory neurotransmitter, which reduces neural excitability rather than increasing it.

Thus, the correct answer is C. Acetylcholine, as it is a key excitatory neurotransmitter in the auditory system.

Question 28: At which nucleus do auditory fibers first cross between the left and right sides of the brainstem?

Answer: C. Superior olivary complex (SOC)

Explanation:

The central auditory pathway follows a hierarchical structure, where signals from the cochlea travel upward through various nuclei:

Cochlear nucleus (CN) – First relay station, located in the brainstem. No major crossing of fibers happens here.

Superior olivary complex (SOC) – First location where bilateral (crossed) auditory information is integrated, making it crucial for sound localization using interaural time and level differences (ITD & ILD).

Inferior colliculus (IC) – Receives already-processed bilateral input but is not the first crossing point.

Medial geniculate body (MGB) – A relay in the thalamus, not involved in initial crossing of fibers.

Thus, the correct answer is C. Superior olivary complex (SOC), as it is the first site where auditory fibers from both ears interact, allowing for binaural hearing and localization.

Question 29: Which statement about a square wave is the most incorrect?

Answer: B. A square wave contains only even harmonics

Explanation:
A square wave is a non-sinusoidal waveform that consists of a fundamental frequency and a series of harmonics. Let's analyze each option:

A. Correct: The fundamental frequency of a waveform is the reciprocal of its period (f=1Tf = \frac{1}{T}f=T1), which is true for all periodic signals, including square waves.

B. Incorrect: A square wave actually contains both odd harmonics (1st, 3rd, 5th, etc.), not just even harmonics. The harmonic structure follows the pattern: fn=(2n−1)f1f_n = (2n - 1) f_1fn =(2n−1)f1 meaning the frequencies are 1st, 3rd, 5th, 7th... harmonics, all odd multiples of the fundamental frequency. Thus, stating that it contains even harmonics is incorrect.

C. Correct: As frequency increases, harmonic amplitudes decrease proportionally to 1/n1/n1/n, meaning higher frequencies have lower amplitudes.

D. Correct: The duty cycle refers to the proportion of time the signal stays in its high state within a full cycle. Changing this ratio affects the wave's spectrum and waveform shape.

Thus, the most incorrect statement is B, as square waves do not contain even harmonics—they only include odd harmonics.

Question 30:

The correct answer is: B. The amplitude of simple harmonic motion decreases gradually as energy dissipates in the form of heat due to friction in the medium.

Explanation:

Damping refers to the process by which the energy of a vibrating system gradually decreases due to resistive forces, such as friction or air resistance. In simple harmonic motion (SHM), the amplitude decreases over time because energy is lost as heat due to friction within the medium. This concept is crucial in hearing care, as damping is used in hearing aids and earmold design to smooth frequency responses and reduce unwanted resonance or distortions.

Why are the other options incorrect?

A. Incorrect: The damping effect varies depending on the medium. For example, vibrations in air experience less damping than those in water because water has higher viscosity, leading to greater energy dissipation.

C. Incorrect: Damping does not cause energy accumulation; instead, it results in energy loss over time, reducing the amplitude of oscillations.

D. Incorrect: Damping occurs in all types of vibratory motion, not just in sinusoidal waves. It affects both simple harmonic and complex oscillatory motions, including non-sinusoidal and nonlinear vibrations.

In audiology, understanding damping is essential for optimizing hearing aid performance, as proper damping can help control sound peaks, minimize distortion, and improve overall sound quality for the user.

Question 31:

Analysis:

Waveform Analysis:

The waveform appears to be random and lacks periodicity, suggesting a type of noise rather than a structured sound like pure tones or music.

Amplitude Spectrum Analysis:

The spectrum shows higher energy concentration in the low-frequency range (below 1000 Hz) and a rapid decline in amplitude as frequency increases.

There is minimal energy above 2000 Hz, indicating a predominantly low-frequency sound.

Answer Explanation:

Given these observations, the most likely answer is:

C. Speech Noise (語音噪音)

Reasoning:

White Noise (A):

White noise has a flat spectrum with equal energy across all frequencies. The provided spectrum does not exhibit this characteristic; it shows a clear roll-off in higher frequencies.

Pink Noise (B):

Pink noise has a spectral density that decreases at a rate of 3 dB per octave, giving it more energy in lower frequencies but not as steep a decline as seen in the graph. The graph shows a sharper decline, suggesting it is not pink noise.

Speech Noise (C):

Speech noise typically has more energy in the low-frequency region (below 1000 Hz) and less in the higher frequencies. This matches the observed spectrum, where the energy is concentrated in the lower frequencies with a sharp decline in the higher range.

Babble Noise (D):

Babble noise, generated from multiple people talking simultaneously, often exhibits more spectral variability and extended higher-frequency content than shown here.

Clinical Relevance:

As a hearing care professional, identifying speech noise is crucial for effective hearing aid programming, ensuring appropriate noise reduction, and enhancing speech intelligibility for the wearer. Recognizing different types of noise aids in creating better auditory environments and improving patient outcomes.

Question 32: Frequency Components of a Square Wave

A square wave is composed of a fundamental frequency and its odd harmonics (i.e., odd multiples of the fundamental frequency).

Answer:

✅ D. 1000, 3000, 5000, 7000 Hz

Explanation:

A square wave contains only odd harmonics of its fundamental frequency:

If the fundamental frequency is 1000 Hz, the harmonics will be:

3rd harmonic: 1000 × 3 = 3000 Hz

5th harmonic: 1000 × 5 = 5000 Hz

7th harmonic: 1000 × 7 = 7000 Hz

The other answer choices contain even harmonics, which are not present in a square wave.

Question 33: Inner Ear Anatomy and Physiology

Analysis of Answer Choices:

A. ✅ Correct:

Outer hair cells (OHCs) are arranged in three rows, while inner hair cells (IHCs) are arranged in a single row along the cochlea.

B. ❌ Incorrect (Correct Answer):

Basilar membrane tuning follows the tonotopic organization:

High-frequency sounds peak at the base of the cochlea (closer to the oval window).

Low-frequency sounds peak at the apex (farther from the base).

The given statement incorrectly states that high-frequency waves peak farther from the cochlear base, which is the opposite of what occurs in reality.

C. ✅ Correct:

Type I auditory nerve fibers (90-95%) connect primarily to inner hair cells.

Type II auditory nerve fibers (5-10%) connect to outer hair cells.

D. ✅ Correct:

Each inner hair cell is innervated by 15–20 auditory neurons, forming a strong one-to-many connection.

Type II auditory nerve fibers have one fiber connecting to multiple outer hair cells (a many-to-one connection).

Final Answer:

✅ B is the incorrect statement.

Question 34: Attenuation of a Low-Pass Filter

A low-pass filter allows frequencies below the cutoff frequency (2000 Hz) to pass through while attenuating frequencies above it. The attenuation rate is given as 6 dB per octave (meaning the signal loses 6 dB for every doubling of frequency).

Step-by-Step Calculation:

Octaves from 2000 Hz to 8000 Hz:

2000 → 4000 Hz (1 octave) → -6 dB

4000 → 8000 Hz (1 octave) → -6 dB

Total attenuation: 6 dB + 6 dB = 12 dB

Answer:

✅ B. 12 dB

Question 35: Sound Pressure Level at a Greater Distance

Sound follows the inverse square law, which states that sound intensity decreases by 6 dB each time the distance is doubled.

Step-by-Step Calculation:

Initial distance = 6 meters, sound level = 60 dB

New distance = 24 meters (which is 4× farther)

Distance doubling steps:

6m → 12m → -6 dB (60 dB → 54 dB)

12m → 24m → -6 dB (54 dB → 48 dB)

Final sound level at 24 meters = 48 dB

Answer:

✅ D. 48 dB

Question 36: Superposition of Two Sinusoids with a 180° Phase Difference

When two sinusoidal waves of the same frequency and amplitude are 180 degrees out of phase, they are exactly opposite in their oscillations. This results in complete destructive interference, meaning the waves cancel each other out entirely.

Answer:

✅ A. The two waves completely cancel each other out.

Explanation:

If two sine waves are in phase (0° phase difference), their amplitudes add up, leading to constructive interference.

If two sine waves are 180° out of phase, one wave is at its peak while the other is at its trough, causing them to cancel out.

This principle is fundamental in noise cancellation, such as active noise-canceling headphones, which generate an inverse sound wave to eliminate unwanted noise.

Question 37: Resonators and Resonant Frequency

A resonator is a system that naturally oscillates at specific resonant frequencies, amplifying certain frequencies while attenuating others.

Analysis of Answer Choices:

A. ✅ Correct:

The resonant frequency is the frequency at which a resonator vibrates with the greatest amplitude when excited by an external force.

B. ✅ Correct:

The resonant frequency of a system depends on its reactance, which is determined by properties like stiffness and mass.

C. ❌ Incorrect (Correct Answer):

A smaller tuning fork actually has a higher resonant frequency, not a lower one.

Larger tuning forks vibrate at lower frequencies, while smaller ones vibrate at higher frequencies due to their reduced mass and stiffness properties.

D. ✅ Correct:

A higher resistance (damping) causes the resonance peak to be broader and less sharp, meaning the system is less selective in amplifying a particular frequency.

Final Answer:

✅ C. "A smaller tuning fork has a lower resonant frequency" is incorrect.

(A smaller tuning fork actually has a higher resonant frequency.)

Question 38: Calculating the Distance Between Adjacent Crests and Troughs

To solve this problem, we need to determine the wavelength (λ) of the sound wave and then find the distance between adjacent crests and troughs.

Step 1: Calculate the Wavelength (λ)

We use the wave equation:

λ=v×T\lambda = v \times Tλ=v×T

where:

v=344v = 344v=344 m/s (speed of sound)

T=0.01T = 0.01T=0.01 s (period)

λ=344×0.01=3.44 meters=344 cm\lambda = 344 \times 0.01 = 3.44 \text{ meters} = 344 \text{ cm}λ=344×0.01=3.44 meters=344 cm

Step 2: Find the Distance Between a Crest and a Trough

The wavelength (λ) is the distance between two consecutive crests or two consecutive troughs.

The distance between a crest and an adjacent trough is half the wavelength:
λ2=3442=172 cm\frac{\lambda}{2} = \frac{344}{2} = 172 \text{ cm}2λ=2344=172 cm

Final Answer:

✅ B. 172 cm

Question 39: Definition of 1000 Mel

The mel scale is a perceptual scale of pitch that reflects how humans perceive changes in frequency. The standard reference point is:

✅ 1000 mel corresponds to a 1000 Hz tone at 40 phons.

Phon is a unit of loudness level, and 40 phons corresponds to the perceived loudness of a 1000 Hz tone at 40 dB SPL.

The mel scale is nonlinear; lower frequencies have a more linear relationship with pitch, while higher frequencies require a greater increase in Hz to be perceived as equal pitch steps.

Final Answer:

✅ D. 1000 Hz, 40 phons

Question 40: A-Weighting and Equal Loudness Curve

A-weighting is commonly used in sound level meters to measure noise levels in a way that approximates human hearing sensitivity, particularly in moderate sound levels.

The human ear is less sensitive to low frequencies at lower sound levels.

The A-weighting curve is designed to approximate the 40-phon equal loudness curve, which represents how humans perceive sound intensity across different frequencies at moderate loudness levels.

Final Answer:

✅ B. 40-phon curve

Question 41: Factors Affecting the Ability to Detect Small Differences in Sound

The human ear's ability to distinguish small differences in sound stimuli is influenced by several key factors, including:

A. Intensity (✅ Correct factor): The ear's sensitivity to differences in loudness (e.g., Just Noticeable Difference in intensity).

B. Frequency (✅ Correct factor): The ability to distinguish pitch differences is frequency-dependent (e.g., frequency discrimination).

C. Time (✅ Correct factor): Temporal resolution affects the ability to detect small timing differences (e.g., gap detection thresholds).

However, D. Impedance (❌ Incorrect factor) refers to the resistance to sound transmission in the auditory system but does not directly influence the perception of small differences in sound stimuli.

Final Answer:

✅ D. Impedance (Impedance does not directly affect discrimination of small sound differences).

Question 42: Perception of a Sound Only in the Louder Ear

When two identical pure tones of the same frequency but different intensities are presented to both ears, the listener perceives the sound only in the ear receiving the louder signal.

This is known as the Stenger Principle, which is commonly used in audiology to detect non-organic (functional) hearing loss.

Explanation:

If a person claims to have hearing loss in one ear but responds inconsistently to tones presented in both ears at different intensities, the Stenger Test can reveal if the loss is real or feigned.

When a louder tone is played in the supposed "bad" ear and a softer tone in the "good" ear, a person with genuine hearing loss should still perceive the tone in the good ear.

However, if they do not respond, it suggests they are faking the hearing loss.

Final Answer:

✅ A. Stenger Principle

Question 43: Correct Statement About Binaural Hearing

Binaural hearing refers to the way both ears work together to improve sound perception, localization, and speech understanding in noisy environments. Let's analyze each option:

A. "When sounds reaching both ears lack low-frequency components, binaural fusion is easier."

❌ Incorrect. Binaural fusion refers to how the brain combines auditory inputs from both ears into a single percept. Low-frequency components are actually crucial for binaural fusion, particularly for interaural time differences (ITD), which help in sound localization.

B. "When there are significant frequency and intensity differences between ears, binaural beats do not occur."

❌ Incorrect. Binaural beats occur when two slightly different frequencies are presented to each ear, creating a perceived "beating" effect in the brain. Frequency and intensity differences influence binaural beats, but they do not eliminate them entirely.

C. "The closer the binaural hearing thresholds, the more significant the binaural loudness summation effect."

✅ Correct. Binaural summation means that sounds heard in both ears contribute additively to loudness perception. If both ears have similar thresholds, the perceived loudness increase is greater compared to cases where one ear has significantly worse hearing.

D. "Binaural summation at threshold typically improves hearing thresholds by 10-20 dB compared to monaural hearing."

❌ Incorrect. The improvement from binaural summation is usually around 3-6 dB, not as large as 10-20 dB.

Final Answer:

✅ C. The closer the binaural hearing thresholds, the more significant the binaural loudness summation effect.

Question 44: The Best Answer

Among the choices, the most significant factor that negatively impacts speech recognition is:

✅ B. Peak-clipping distortion

Reasoning:

Peak-clipping distorts the amplitude of the speech waveform, removing crucial information and making speech unnatural and harder to understand. This type of distortion directly affects phoneme clarity, which is critical for speech intelligibility.

Other options (like reverberation) can also affect recognition, but peak-clipping causes an immediate and direct loss of speech information, making it the best answer.

Question 45: Correct Order of Auditory Signal Transmission

The process of sound transmission from the outer ear to the inner ear follows a precise anatomical pathway:

Sound waves enter the ear canal and vibrate the tympanic membrane (eardrum).

The vibrations are transmitted through the ossicular chain: malleus (鎚骨) → incus (砧骨) → stapes (鐙骨).

The stapes footplate moves against the oval window (卵圓窗), creating fluid waves in the cochlea's scala vestibuli (前庭階).

Now, let's evaluate the choices:

A. 鼓膜 → 鎚骨 → 砧骨 → 鐙骨 → 卵圓窗 → 前庭階

✅ Correct. This is the correct sequence of sound transmission.

B. 鼓膜 → 鎚骨 → 砧骨 → 鐙骨 → 圓窗 → 前庭階

❌ Incorrect. The round window (圓窗) does not directly receive vibrations; it serves as a pressure release for the cochlear fluid movement.

C. 鼓膜 → 鎚骨 → 砧骨 → 鐙骨 → 卵圓窗 → 鼓室階

❌ Incorrect. The oval window (卵圓窗) transmits vibrations to the scala vestibuli (前庭階), not the scala tympani (鼓室階).

D. 鼓膜 → 鎚骨 → 砧骨 → 鐙骨 → 圓窗 → 鼓室階

❌ Incorrect. The round window (圓窗) is not part of the direct transmission pathway of sound.

Final Answer:

✅ A. 鼓膜 → 鎚骨 → 砧骨 → 鐙骨 → 卵圓窗 → 前庭階

Question 46: Correct Statement About Sone

Sone is a unit of perceived loudness, where:

1 sone is the perceived loudness of a 1000 Hz tone at 40 dB SPL (Sound Pressure Level).

The phon scale relates sound level to perceived loudness, where 40 phons = 1 sone.

As loudness doubles, the sone value doubles (e.g., 2 sones = a sound twice as loud as 1 sone).

Now, let's analyze the choices:

A. "One sone is defined as the loudness of a 1000 Hz pure tone at 40 dB HL."

❌ Incorrect. Sone is based on SPL (sound pressure level), not HL (hearing level).

B. "One sone is defined as the loudness of a 1000 Hz pure tone at 40 dB SL."

❌ Incorrect. SL (sensation level) is relative to an individual's threshold, whereas sone is based on absolute SPL.

C. "One sone is equivalent to 40 phons of loudness."

✅ Correct. By definition, 1 sone = 40 phons at 1000 Hz.

D. "At the same phon level, loudness perception changes with frequency."

❌ Incorrect. The phon scale already accounts for frequency-dependent loudness perception, meaning at the same phon level, perceived loudness remains consistent.

Final Answer:

✅ C. One sone is equivalent to 40 phons of loudness.

Question 47: Relationship Between Hit Rate and False Alarm Rate in ROC Curve

In the Receiver Operating Characteristic (ROC) curve, the hit rate (true positive rate) and false alarm rate (false positive rate) are influenced by response bias. When sensitivity remains constant, changing the response bias affects both rates in a predictable manner:

A liberal response bias increases both hit rate and false alarm rate (responding "yes" more often).

A conservative response bias decreases both hit rate and false alarm rate (responding "yes" less often).

This means the hit rate and false alarm rate are directly proportional, making the correct answer:

✅ C. Two are proportional (成正比).

Question 48: Identifying the Incorrect Statement About Frequency Difference Limen (DLF)

The Difference Limen for Frequency (DLF) refers to the smallest detectable change in frequency that a listener can perceive. Key principles include:

DLF decreases as frequency increases up to a certain point (~600–2000 Hz).

Weber's fraction (Δf/f) is smallest between 600–2000 Hz, meaning frequency discrimination is most precise in this range.

Weber's fraction varies with frequency (it is not a fixed value).

DLF is affected by intensity—better discrimination occurs at higher intensities.

Now, let's analyze the options:

A. "DLF decreases as frequency increases."

✅ Correct. DLF is smaller at higher frequencies, but only up to a certain range (~600–2000 Hz).

B. "Weber's fraction ($\Delta f/f$) is smallest between 600–2000 Hz, around 0.002."

✅ Correct. This is well-documented in auditory research.

C. "Weber's fraction depends on frequency range."

✅ Correct. Weber's fraction is not a constant—it varies with frequency.

D. "DLF is unrelated to sound intensity."

❌ Incorrect. DLF is affected by intensity—at higher sound levels, frequency discrimination improves.

Final Answer:

✅ D. DLF is unrelated to sound intensity. (Incorrect statement)

Question 49: Octave Interval in Hearing Tests

In audiometry, sound frequencies are commonly tested in octave intervals. An octave represents a doubling of frequency, meaning each frequency in the sequence is twice the previous one.

For example, standard audiometric test frequencies include:
125 Hz → 250 Hz → 500 Hz → 1000 Hz → 2000 Hz → 4000 Hz → 8000 Hz

Each step follows the pattern:

fnext=2×fpreviousf_{\text{next}} = 2 \times f_{\text{previous}}fnext =2×fprevious

Thus, the correct answer is:
✅ A. 2 times

Question 50: Meaning of Volume Representation in Tympanometry

Tympanometry measures middle ear function by varying air pressure in the external ear canal and recording changes in acoustic admittance (compliance). The vertical axis of a tympanogram represents compliance (sound flow ease), often expressed in cubic centimeters (c.c. or mL).

This measurement reflects how much acoustic energy is absorbed by the middle ear system compared to an equivalent air volume in the ear canal. The tympanometer calculates this by comparing the sound pressure level before and after introducing a probe tone into a controlled space.

Thus, the c.c./mL measurement reflects how much the measured acoustic admittance corresponds to an equivalent volume of air rather than the actual middle ear cavity volume.

The correct answer is:
✅ B. Fixed input sound pressure corresponds to an equivalent volume change

2106

Question 1: SOAP – Objective Data (O)

SOAP stands for Subjective (S), Objective (O), Assessment (A), and Plan (P) in medical documentation. The Objective (O) section includes measurable, observable, or testable results obtained through physical examination, audiological tests, and other diagnostic tools.

① Tympanogram Type A for both ears → Objective (Tympanometry results are measurable and recorded findings.) ✅

② Persistent tinnitus in the right ear → Subjective (Tinnitus is a symptom reported by the patient.) ❌

③ Dizziness for one week → Subjective (Dizziness is a symptom described by the patient.) ❌

④ Rinne test (+) → Objective (A physical examination test result.) ✅

⑤ Hearing deterioration in the left ear → Subjective (The patient's complaint, not a measurable result.) ❌

⑥ Mild bilateral sensorineural hearing loss → Objective (Audiometry results are documented findings.) ✅

⑦ Follow-up appointment in one month → Plan (P) (Follow-up scheduling belongs to the Plan section.) ❌

Correct answer:

✅ D. Only ① and ④

Question 2: Otoscopic Examination Precautions

Before performing a hearing test, proper otoscopic examination ensures there are no obstructions (e.g., cerumen, infections, perforations) affecting the results.

A. The procedure should be performed gently. → Correct (Gentle handling prevents discomfort and injury.) ✅

B. Ensure sufficient light in the otoscope. → Correct (Adequate lighting is crucial for a clear view of the ear canal and eardrum.) ✅

C. For adults, pull the pinna backward and upward before inserting the otoscope. → Correct (This straightens the ear canal for better visualization in adults.) ✅

D. For infants and young children, pull the pinna upward before inserting the otoscope. → Incorrect (For infants and young children, the pinna should be pulled downward and backward, not upward, due to the different anatomical angle of the ear canal.) ❌

Correct answer:

✅ D. Pulling the infant's pinna upward before inserting the otoscope (Incorrect practice)

Question 3: Otitis Media and Related Pathologies

Understanding middle ear infections and their complications is crucial for hearing care professionals. Let’s analyze each option:

A. Chronic otitis media may lead to cholesteatoma formation, most commonly occurring in the pars tensa and perforated areas of the tympanic membrane.

❌ Incorrect – Cholesteatoma is indeed a complication of chronic otitis media, but it most commonly forms in the pars flaccida (upper portion of the tympanic membrane) rather than the pars tensa (lower portion).

B. Otitis media is the most common cause of mixed hearing loss, with the highest incidence in children.

❌ Incorrect – Otitis media primarily causes conductive hearing loss due to fluid accumulation or tympanic membrane damage. While it can sometimes contribute to mixed hearing loss (if inner ear damage occurs), it is not the most common cause. Additionally, though otitis media is common in children, mixed hearing loss is more often associated with chronic conditions or other underlying pathologies.

C. Persistent or recurrent otitis media is called chronic otitis media; therefore, all chronic otitis media cases involve tympanic membrane perforation, making reinfection more likely.

❌ Incorrect – While many cases of chronic otitis media involve tympanic membrane perforation, not all do. Chronic otitis media with effusion (OME) does not necessarily involve perforation but can still lead to persistent fluid buildup and hearing issues.

D. Negative middle ear pressure is caused by Eustachian tube dysfunction, leading to poor ventilation and air retention in the middle ear cavity.

✅ Correct – Eustachian tube dysfunction prevents proper aeration of the middle ear, leading to negative pressure, which can cause tympanic membrane retraction and contribute to otitis media with effusion or other middle ear disorders.

Correct answer: ✅ D. Negative middle ear pressure is caused by Eustachian tube dysfunction, leading to poor ventilation and air retention in the middle ear cavity.

Question 4: Audiometer Calibration in Occupational Hearing Conservation Programs

In occupational hearing conservation programs, audiometers must be regularly calibrated to ensure accurate hearing assessments. The two main types of calibration are:

Biological/Listening Checks – Performed daily or before each use.

Acoustic Calibration – Required annually (every 12 months) to maintain accuracy according to OSHA (Occupational Safety and Health Administration) and ANSI (American National Standards Institute) guidelines.

Correct answer: ✅ D. 12 months (Annual calibration required).

Question 5: Factors Affecting Pure-Tone Audiometry

Several factors can influence pure-tone audiometry results, including standing waves, tactile responses, auditory radiation, and collapsed ear canals. Let's analyze each option:

A. Auditory radiation (acoustical radiation) most likely affects 125 Hz or 250 Hz.

✅ Correct – Auditory radiation refers to sound vibrations from the bone-conduction oscillator being transmitted to the external ear canal, especially at lower frequencies (125 Hz and 250 Hz). This can lead to unintended air-conduction stimulation, potentially affecting test results.

B. Tactile responses most likely affect around 1000 Hz.

❌ Incorrect – Tactile responses occur when bone-conducted sounds at high intensities (typically at low frequencies, such as 250 Hz and 500 Hz) are physically felt rather than heard. This can lead to false-positive responses in patients with severe hearing loss. 1000 Hz is generally not where tactile responses are most commonly observed.

C. Collapsed ear canals most likely affect around 2000 Hz and are common in older adults.

❌ Incorrect – Collapsed ear canals are more likely to affect mid to high frequencies, typically above 3000 Hz, rather than specifically at 2000 Hz. They are indeed more common in older adults due to reduced cartilage rigidity, leading to artificially elevated thresholds when using supra-aural headphones.

D. Standing waves most likely affect around 8000 Hz and can be confirmed by adjusting headphones.

❌ Incorrect – Standing waves occur due to wave interference in the ear canal, primarily affecting higher frequencies, around 6000 Hz, rather than 8000 Hz. They can cause inaccurate hearing threshold measurements, and adjusting headphone placement can help mitigate their effect.

Correct answer: ✅ A. Auditory radiation most likely affects 125 Hz or 250 Hz.

Question 6: Maximum Air-Bone Gap According to Rosowski & Relkin (2001)

Rosowski and Relkin (2001) studied the maximum difference between air conduction (AC) and bone conduction (BC) thresholds, also known as the maximum air-bone gap (ABG). This difference occurs because bone conduction thresholds cannot exceed air conduction due to the nature of sound transmission in the auditory system.

The maximum ABG is approximately 60-65 dB, as bone conduction can still stimulate the cochlea directly even when air conduction is impaired.

A greater ABG (e.g., 75 dB) is generally not physiologically possible because bone conduction never fully isolates the middle ear function.

Correct answer: ✅ C. 65 dB

Question 7: Hearing Disability Evaluation in Taiwan (2023 Regulations)

According to the 2023 Disability Evaluation Guidelines in Taiwan, the calculation of the overall bilateral hearing impairment percentage is based on different weightings for the better ear (优耳) and the worse ear (劣耳).

The better ear (优耳) contributes 2/3 (or 66.7%) of the overall hearing impairment calculation.

The worse ear (劣耳) contributes 1/3 (or 33.3%) of the overall hearing impairment calculation.

This method ensures that the better-functioning ear has a greater impact on the final disability percentage, reflecting the individual's actual hearing ability in daily life.

Correct answer: ✅ B. 2/3; 1/3

Question 8: Audiogram Format and Symbols

Audiograms use standardized symbols and formatting to represent air conduction (AC) and bone conduction (BC) thresholds, ensuring consistency in hearing assessment. Let's analyze each option:

A. The air conduction threshold symbols should be placed directly on the audiogram line.

✅ Correct – Air conduction thresholds (e.g., "O" for the right ear and "X" for the left ear) are plotted directly on the frequency-intensity grid of the audiogram at the intersection of the corresponding dB HL and frequency.

B. Half-octave frequency grid lines on the audiogram are represented as dashed lines.

✅ Correct – Frequencies like 750 Hz, 1500 Hz, 3000 Hz, and 6000 Hz are considered half-octave frequencies and are represented by dashed lines on standard audiograms.

C. Bone conduction threshold symbols are sometimes connected by dashed lines.

✅ Correct – Bone conduction thresholds (e.g., ">" for the left ear, "<" for the right ear) are sometimes connected by dashed lines when they are recorded across multiple frequencies. This is especially useful for visualizing trends in bone conduction hearing loss.

D. The spacing between grid lines for one octave and 10 dB differences is the same.

❌ Incorrect – The horizontal axis (frequency) follows a logarithmic scale, meaning octave intervals (e.g., 500 Hz → 1000 Hz → 2000 Hz) are not spaced equally. In contrast, the vertical axis (dB HL) follows a linear scale, meaning that 10 dB increments are evenly spaced.

Correct answer: ✅ D is incorrect.

Question 9: Clinical Manifestations of Meniere’s Disease

Meniere’s disease is an inner ear disorder characterized by episodes of vertigo, fluctuating hearing loss, tinnitus, and a feeling of fullness in the ear. Let’s analyze each option:

A. Caused by endolymphatic hydrops (inner ear fluid imbalance).

✅ Correct – Meniere’s disease is due to endolymphatic hydrops, which means excessive buildup of endolymph (inner ear fluid) leads to increased pressure, affecting balance and hearing.

B. Common symptoms include vertigo, ear fullness, and tinnitus.

✅ Correct – Classic symptoms of Meniere’s disease include:

Episodic vertigo (lasting minutes to hours)

Aural fullness (ear pressure sensation)

Tinnitus (ringing in the ear)

Fluctuating sensorineural hearing loss

C. The audiogram often shows low-frequency hearing loss.

✅ Correct – Early-stage Meniere’s disease typically presents with low-frequency sensorineural hearing loss, which may fluctuate over time. As the disease progresses, hearing loss can extend to higher frequencies.

D. The hearing loss is irreversible.

❌ Incorrect – Meniere’s-related hearing loss is initially reversible in early stages, meaning hearing can improve between attacks. However, in later stages, the hearing loss may become permanent due to progressive cochlear damage.

Correct answer: ✅ D. Meniere’s-related hearing loss is initially reversible, especially in early stages.

Question 10: Early Detection of Ototoxicity and High-Frequency Audiometry

Ototoxic medications (e.g., aminoglycoside antibiotics, cisplatin, loop diuretics) often damage the high-frequency regions of the cochlea first, leading to early high-frequency hearing loss. High-frequency audiometry can help detect changes before noticeable hearing loss occurs.

A. 8000 Hz

✅ Correct – Ototoxicity typically affects frequencies above 8000 Hz first, so testing at or beyond this range (e.g., 10,000–20,000 Hz, if available) can detect early signs of damage.

B. 2000 Hz

❌ Incorrect – Mid-frequency hearing is usually affected later, after high-frequency damage has already occurred.

C. 1000 Hz

❌ Incorrect – Damage at 1000 Hz usually occurs in more advanced stages of ototoxic hearing loss.

D. 500 Hz

❌ Incorrect – Low frequencies (such as 500 Hz) are typically the last to be affected by ototoxic drugs.

Correct answer: ✅ A. 8000 Hz (or higher) should be tested for early ototoxicity detection.

Question 11: Maximum Permissible Ambient Noise Level (MPANL) in a Hearing Test Environment

The maximum permissible ambient noise level (MPANL) varies depending on the type of transducer used in audiometry. Some transducers are more sensitive to environmental noise, requiring a quieter testing environment. Let's analyze each option:

A. Bone Conduction Vibrator

✅ Correct – MPANL is the highest for this transducer

Bone conduction testing bypasses the outer and middle ear, stimulating the cochlea directly.

Since it does not benefit from ear canal isolation, it is highly susceptible to ambient noise, leading to artificially elevated bone conduction thresholds if background noise is too high.

Therefore, the permissible noise level must be the lowest for accurate results, making MPANL the highest in comparison to other transducers.

B. Supra-aural Headphones (Over-the-ear headphones)

❌ Lower MPANL than bone conduction

These provide some noise isolation, but ambient noise can still leak in, affecting low-frequency hearing threshold measurements.

MPANL is higher than that for bone conduction vibrators but lower than that for insert earphones.

C. Sound Field Speakers (Free-field testing)

❌ Lower MPANL than bone conduction

Sound field testing (typically used for patients who cannot wear headphones, such as young children or cochlear implant users) is very sensitive to background noise.

However, it still has more noise tolerance than bone conduction vibrators because the stimuli are presented at higher sound levels.

D. Insert Earphones

❌ Lowest MPANL (most resistant to noise contamination)

Insert earphones provide the best isolation from ambient noise by sealing the ear canal.

This makes them the least affected by environmental noise, so they have the lowest MPANL requirement (i.e., the testing room does not need to be as quiet compared to other transducers).

Final Answer:

✅ A. Bone Conduction Vibrator – It requires the quietest environment for accurate testing, meaning it has the highest maximum permissible ambient noise level (MPANL).

Question 12: Factors for Air-Conduction Masking in Pure-Tone Audiometry

When determining whether air-conduction (AC) masking is necessary, we consider several key factors related to interaural attenuation (IA), air conduction, and bone conduction thresholds. Let's analyze each option:

A. Different frequencies have different IA values.
✅ Correct – Interaural attenuation (IA) varies by transducer and frequency:

Supra-aural headphones: IA ≈ 40 dB

Insert earphones: IA ≈ 50–60 dB (higher with deep insertion)

Bone conduction: IA ≈ 0 dB (minimal attenuation)
Since IA differs across frequencies and transducers, it must be considered in masking decisions.

B. The unmasked air-conduction threshold of the test ear.
✅ Correct – This threshold is essential because it helps determine whether sound might cross over to the non-test ear, which would require masking.

C. The bone-conduction threshold of the non-test ear.
✅ Correct – If cross-hearing occurs, the non-test ear's bone-conduction threshold helps determine if the sound is being heard through the better ear instead.

D. The test ear's stimulus level minus IA being less than the non-test ear's bone-conduction threshold.
❌ Incorrect – The correct masking rule is:
Masking is needed if:

Test ear AC threshold−IA≤Non-test ear BC threshold\text{Test ear AC threshold} - \text{IA} \leq \text{Non-test ear BC threshold}Test ear AC threshold−IA≤Non-test ear BC threshold

This option incorrectly suggests "less than" (<) instead of "equal to or less than" (≤), making it an incorrect statement.

Correct answer: ✅ D. The incorrect statement is "test ear stimulus - IA < non-test ear BC threshold."

Question 13: When to Apply Air-Conduction Masking with Insert Earphones

Masking is needed if the air-conduction threshold of the test ear minus IA is equal to or worse than the bone-conduction threshold of the non-test ear.

Given:

Non-test ear (NTE) air-conduction threshold at 2000 Hz = 30 dB HL

NTE bone-conduction threshold = 0 dB HL

Insert earphones have an IA ≈ 60 dB

Using the masking rule:

Test ear AC−IA≤Non-test ear BC\text{Test ear AC} - \text{IA} \leq \text{Non-test ear BC}Test ear AC−IA≤Non-test ear BC Test ear AC−60≤0\text{Test ear AC} - 60 \leq 0Test ear AC−60≤0 Test ear AC≥60\text{Test ear AC} \geq 60Test ear AC≥60

Thus, masking is needed when the test ear's air-conduction threshold is ≥ 65 dB HL (the closest answer choice).

Correct answer: ✅ C. 65 dB HL

Question 14: Reasons for Using Insert Earphones in Behavioral Audiometry

Insert earphones are commonly used in pure-tone audiometry due to their advantages over supra-aural (over-the-ear) headphones. Let’s analyze each option:

A. Allows the test subject to move their head more freely.

✅ Correct – Insert earphones are placed directly into the ear canal, making them less affected by head movement compared to supra-aural headphones, which may shift position.

B. Their interaural attenuation (IA) is lower than supra-aural headphones.

❌ Incorrect – Insert earphones actually have a higher IA (50–60 dB) compared to supra-aural headphones (IA ≈ 40 dB).

Higher IA reduces the risk of cross-hearing, meaning less masking is needed.

The statement that insert earphones have a "smaller IA" is incorrect.

C. Helps prevent ear canal collapse.

✅ Correct – Insert earphones bypass the outer portion of the ear canal, preventing collapsed ear canals that can artificially elevate air-conduction thresholds, especially in elderly patients.

D. Allows separate testing of each ear.

✅ Correct – Insert earphones provide better ear separation, ensuring that the test signal is delivered to the intended ear, which is important for accurate threshold measurement.

Final Answer:

✅ B is incorrect – Insert earphones have higher interaural attenuation (IA), not lower, compared to supra-aural headphones.

Correct answer: ✅ B. "Their IA is lower than supra-aural headphones" is incorrect.

Question 15: Interpretation of the Speech Recognition Performance-Intensity Function Graph

This graph represents speech recognition performance (word recognition score, WRS) as a function of speech presentation level in dB HL. Let's analyze each option:

A. This graph is called a speech recognition performance-intensity function.

✅ Correct – This type of graph is commonly known as a performance-intensity (PI) function for speech recognition. It illustrates the relationship between speech presentation level (x-axis) and the percentage of correctly recognized words (y-axis).

B. The y-axis represents the number of correct responses.

❌ Incorrect – The y-axis represents the percentage (%) of correctly identified speech stimuli, not the absolute number of correct responses.

C. Curves "a" and "b" represent normal hearing, both reaching 100%.

✅ Correct – In normal hearing individuals, the word recognition score (WRS) typically reaches 100% at a moderate speech presentation level (around 30-40 dB HL in this graph).

"a" and "b" both reach 100%, indicating normal hearing.

D. Curves "c" and "d" represent sensorineural hearing loss (SNHL) because they do not reach 100%.

✅ Likely correct – In sensorineural hearing loss (SNHL), the speech recognition score often does not reach 100% even at high presentation levels due to distorted auditory processing (retrocochlear dysfunction or cochlear damage).

Curves "c" and "d" show reduced maximum performance (≤ 80%), a common characteristic of SNHL.

However, some conductive hearing loss cases could also have reduced performance if there are additional cochlear factors.

Final Answer:

✅ Correct answers: A, C, and D. The best single answer choice is:

✅ C. "a and b represent normal hearing, both reaching 100%."

Question 16: Rollover Index Calculation in Performance-Intensity (PI) Functions

The rollover index (RI) is used to assess retrocochlear pathology and is calculated using the formula:

Rollover Index=PBmax−PBminPBmax\text{Rollover Index} = \frac{PB_{\text{max}} - PB_{\text{min}}}{PB_{\text{max}}}Rollover Index=PBmax PBmax −PBmin

Given:

PBmax (Maximum Speech Recognition Score) = 88%

PBmin (Minimum Speech Recognition Score at Higher Intensity) = 56%

RI=88−5688=3288=0.36RI = \frac{88 - 56}{88} = \frac{32}{88} = 0.36RI=8888−56 =8832 =0.36

Thus, the correct answer is:
✅ B. 0.36

Question 17: Speech Detection Threshold (SDT) vs. Speech Recognition Threshold (SRT)

Let’s analyze each statement:

A. SDT is also called Speech Awareness Threshold (SAT).
✅ Correct – Speech Detection Threshold (SDT) and Speech Awareness Threshold (SAT) are interchangeable terms. Both refer to the lowest level at which a patient can detect the presence of speech, without needing to understand or repeat it.

B. SDT is typically 12 dB lower than the Speech Recognition Threshold (SRT).
✅ Correct – Since SDT only requires detection (not understanding), it is usually lower (better) than the SRT by about 7-12 dB.

C. SDT is the lowest level where speech is heard 50% of the time.
✅ Correct – The definition of SDT is the lowest intensity at which speech can be detected in at least 50% of presentations.

D. The key difference between SRT and SDT is that SRT requires recognition, while SDT only requires detection.
✅ Correct – SRT (Speech Recognition Threshold) requires the patient to correctly repeat or identify words, whereas SDT only requires awareness of sound presence.

Since all other options are correct, the most inappropriate statement is:
❌ None of these statements are incorrect. However, if forced to choose the least precise statement, "B" might be debated as the difference is typically 7-12 dB rather than always exactly 12 dB.

Final Answer:
✅ There is no completely incorrect answer, but if required, the least precise statement would be B.

Question 18: Masking Level for Speech Discrimination in Asymmetric SNHL

Step 1: Understanding the Given Data

Left ear SRT = 25 dB HL

Right ear SRT = 70 dB HL

Presentation level for Right ear Word Discrimination Score (WDS) = 30 dB SL

This means the test word list will be played at: 70 dB HL+30 dB SL=100 dB HL70 \text{ dB HL} + 30 \text{ dB SL} = 100 \text{ dB HL}70 dB HL+30 dB SL=100 dB HL

Need to calculate the masking noise level for the left ear

Step 2: Masking Formula for Speech Discrimination

Effective Masking Level=Presentation Level in Test Ear−Interaural Attenuation+Safety Factor\text{Effective Masking Level} = \text{Presentation Level in Test Ear} - \text{Interaural Attenuation} + \text{Safety Factor}Effective Masking Level=Presentation Level in Test Ear−Interaural Attenuation+Safety Factor

Assuming Interaural Attenuation (IA) = 40 dB for speech with standard headphones:
100 dB HL−40 dB IA=60 dB HL100 \text{ dB HL} - 40 \text{ dB IA} = 60 \text{ dB HL}100 dB HL−40 dB IA=60 dB HL

Adding a safety factor (usually 10 dB): 60 dB HL+10=70 dB HL60 \text{ dB HL} + 10 = 70 \text{ dB HL}60 dB HL+10=70 dB HL

Thus, the correct masking level for the left ear is:
✅ B. 70 dB HL

Question 19: Best Practice for Measuring Speech Recognition Threshold (SRT)

Let's analyze each option:

A. Familiarizing the patient with word lists before testing can lower the threshold by 10 dB.
✅ Correct – Word familiarization improves accuracy and reduces SRT variability, often leading to a threshold improvement of around 5-10 dB.

B. Using or omitting a carrier phrase (e.g., "Say the word ___") does not significantly affect SRT results.
❌ Incorrect – Carrier phrases can affect speech perception by preparing the patient and influencing test reliability, especially in older adults or those with auditory processing difficulties.

C. The only method for SRT measurement is the "down 10, up 5" method.
❌ Incorrect – While the "down 10, up 5" method is common, other adaptive methods exist, such as the ascending method, particularly for patients with severe hearing loss.

D. SRT is the lowest level at which the patient can perfectly repeat all two-syllable words.
❌ Incorrect – SRT is not based on 100% accuracy; it is defined as the lowest level where at least 50% of the presented spondee words are correctly identified.

Final Answer:

✅ A. Familiarizing the patient with the word list before testing can lower the threshold by 10 dB.

Question 20: Identifying the Incorrect Statement About Speech Detection Threshold (SDT)

Let's analyze each option:

A. SDT is defined as the lowest level at which speech can be detected.

✅ Correct – SDT (Speech Detection Threshold) refers to the minimum sound level at which a person can detect the presence of speech, but they do not need to understand or repeat it.

B. SDT requires standardized speech test materials.

❌ Incorrect – SDT does not require standardized word lists like Speech Recognition Threshold (SRT). It can be measured using any speech-like stimulus, such as running speech, the patient's name, or familiar words, making this statement incorrect.

C. Research and clinical practice show SDT results are lower (better) than SRT results.

✅ Correct – SDT is typically better (lower) than SRT by around 7-12 dB because speech detection requires only awareness, whereas speech recognition requires identification of words.

D. SDT is commonly used for behavioral hearing tests in infants.

✅ Correct – Infants and individuals who cannot reliably repeat words often undergo SDT testing instead of SRT because it assesses their ability to detect speech stimuli rather than recognize words.

Final Answer:

✅ B. SDT requires standardized speech test materials. (Incorrect statement)

Question 21: Speech Discrimination Testing - Masking Noise Level and Type

When conducting a speech discrimination test, masking may be necessary in the non-test ear to prevent cross-hearing. The appropriate masking noise type and level should follow these principles:

A. Fixed 40 dB HL speech noise

❌ Incorrect – Masking should be based on the presentation level of the test ear rather than a fixed level.

B. Test ear presentation level minus 20 dB HL (speech noise)

❌ Incorrect – This would provide insufficient masking in many cases, as effective masking typically requires a level closer to the test ear level.

C. Test ear presentation level plus 20 dB HL (speech noise)

✅ Correct – The recommended masking level is typically 20 dB below the presentation level of the test ear to ensure effective masking without over-masking.

D. Fixed 40 dB HL narrowband noise

❌ Incorrect – Narrowband noise is not used for speech masking; speech noise (or white noise) is preferred.

Final Answer: ✅ C. Test ear presentation level plus 20 dB HL (speech noise).

Question 22: Masking-Level Difference (MLD) - Identifying the Incorrect Statement

MLD is a measure of binaural auditory processing and is useful for detecting brainstem dysfunctions. Let's analyze each statement:

A. MLD can be used to measure central auditory function.

✅ Correct – MLD is a central auditory processing test, particularly useful in assessing brainstem function.

B. The largest MLD is observed when comparing SπNo (out-of-phase signal) with SoNo (in-phase signal).

✅ Correct – The greatest masking release occurs when the signal is out of phase (Sπ) but noise is in phase (No) compared to both being in phase (SoNo).

C. Peripheral hearing loss does not affect MLD values.

❌ Incorrect – Peripheral hearing loss (especially sensorineural hearing loss) can reduce or eliminate MLD, as it impacts binaural hearing cues. This makes C the incorrect statement.

D. MLD testing is more sensitive to brainstem lesions than cortical lesions.

✅ Correct – Since MLD assesses binaural interaction at the brainstem level, it is more effective at detecting brainstem abnormalities rather than cortical ones.

Final Answer: ✅ C. Peripheral hearing loss does not affect MLD values. (Incorrect statement)

Question 23: Clinical Conditions Requiring Only PTA vs. Electrocochleography (ECochG)

Pure-Tone Audiometry (PTA) is a fundamental test for hearing assessment, but certain conditions require additional Electrocochleography (ECochG) testing, especially when cochlear or neural dysfunction is suspected. Let's analyze each option:

A. Cochlear Implant (CI) Pre-Evaluation

✅ Requires ECochG – CI candidates often require ECochG to assess cochlear function and auditory nerve integrity, especially in cases of neural degeneration or auditory neuropathy.

B. Superior Semicircular Canal Dehiscence (SSCD)

✅ Requires ECochG – SSCD affects inner ear pressure regulation, and ECochG can detect abnormal summating potentials (SP) and action potentials (AP) ratios, which are often elevated.

C. Meniere's Disease

✅ Requires ECochG – ECochG is commonly used in Meniere's disease diagnosis, as it helps detect endolymphatic hydrops, which is associated with the disorder.

D. Middle Ear Ventilation Tube (Post-Surgery Follow-up)

❌ Does NOT require ECochG – Middle ear ventilation tube follow-up mainly involves PTA and tympanometry to evaluate conductive hearing function and middle ear status, making ECochG unnecessary.

Final Answer: ✅ D. Middle Ear Ventilation Tube Post-Surgery Follow-up (Does NOT require ECochG).

Question 24: Indicators of Non-Organic (Functional) Hearing Loss

Non-organic or functional hearing loss (e.g., malingering or psychogenic hearing loss) presents inconsistencies in test results. Let's analyze the statements:

A. Lack of False Positive Responses

✅ Correct – Patients with true hearing loss often produce false positives due to their eagerness to hear sounds. A complete lack of false positive responses may indicate malingering.

B. Air Conduction Shows a Shadow Curve, but Bone Conduction Does Not

✅ Correct – In true unilateral hearing loss, if the non-test ear has normal hearing, a shadow curve appears due to cross-hearing. However, in functional hearing loss, bone conduction does not match expected patterns.

C. Inconsistent Thresholds, Retest Variability ≥ 15 dB

✅ Correct – Functional hearing loss often presents with large test-retest variability, as patients may guess or exaggerate responses. In genuine cases, threshold variability is generally ≤ 10 dB.

D. Discrepancy of ≥ 12 dB Between PTA and Speech Recognition Threshold (SRT)

❌ Incorrect – Normally, PTA and SRT should be within ±7 dB. In functional hearing loss, this gap is often much greater than 12 dB, sometimes exceeding 20–30 dB. Therefore, a 12 dB difference alone is not the best indicator of non-organic hearing loss.

Final Answer: ✅ D. PTA-SRT Difference of ≥ 12 dB (Most inappropriate indicator).

Question 25: Bone Conduction Testing with a Hearing Instrument vs. Traditional Tuning Fork Tests

Tuning fork tests are traditionally used for differentiating conductive vs. sensorineural hearing loss. However, some of these tests can also be performed using a bone conduction transducer from an audiometer. Let's analyze each test:

Schwabach Test ✅ (Can be done with bone conduction transducer)

This test compares the patient's bone conduction hearing with that of the examiner.

Since it relies entirely on bone conduction, it can be replicated with a bone conduction transducer.

Weber Test ✅ (Can be done with bone conduction transducer)

A midline test that helps differentiate unilateral conductive vs. sensorineural hearing loss.

It can be performed using a bone conduction transducer by placing it at the midline of the forehead.

Rinne Test ❌ (Not typically done with bone conduction transducer)

Compares air conduction (AC) vs. bone conduction (BC) using a tuning fork.

Since the transducer does not provide an air conduction reference, it is not a direct replacement for a tuning fork in this test.

Bing Test ✅ (Can be done with bone conduction transducer)

Assesses occlusion effect by opening/closing the ear canal while presenting a bone-conducted tone.

This can be replicated using a bone conduction transducer and manually opening/closing the ear canal.

Final Answer: ✅ C. ①②④ (Schwabach, Weber, Bing tests can be done with bone conduction transducer, but not Rinne test).

Question 26: Loudness Recruitment and the Pathological Site

Loudness recruitment refers to the abnormal growth of perceived loudness in patients with sensorineural hearing loss (SNHL), particularly in cochlear pathology.

A. External Ear (Outer Ear) ❌

Outer ear conditions (e.g., cerumen impaction, external otitis) cause conductive hearing loss, which does not result in recruitment.

B. Middle Ear ❌

Middle ear pathology (e.g., otitis media, ossicular chain dysfunction) also leads to conductive hearing loss, which does not involve loudness recruitment.

C. Cochlea ✅

Loudness recruitment is a hallmark of cochlear (sensory) hearing loss, particularly due to outer hair cell damage in the cochlea.

Common causes include noise-induced hearing loss, presbycusis, and ototoxicity.

D. Retrocochlear (Beyond the Cochlea, e.g., Auditory Nerve/Brainstem) ❌

Retrocochlear pathologies (e.g., acoustic neuroma, auditory neuropathy) often cause decruitment (reduced loudness perception), not loudness recruitment.

Final Answer: ✅ C. Cochlea (Loudness recruitment is associated with cochlear pathology).

Question 27: Identifying Occlusion Effect Using Tuning Fork Tests

The occlusion effect (OE) occurs when the ear canal is physically blocked, causing enhanced bone conduction perception, particularly for low-frequency sounds (≤1000 Hz). This effect is useful for distinguishing sensorineural vs. conductive hearing loss.

Analysis of the Tuning Fork Tests:

A. Weber Test ❌

This test determines lateralization in asymmetric hearing loss but does not directly assess occlusion effect.

B. Schwabach Test ❌

This compares the patient’s bone conduction hearing to that of the examiner, not the occlusion effect.

C. Bing Test ✅ (Correct Answer)

The Bing test is specifically designed to detect the occlusion effect by alternately closing and opening the ear canal while presenting bone conduction stimuli.

If the sound becomes louder when the canal is closed → normal hearing or sensorineural hearing loss (OE present).

If there is no change → conductive hearing loss (OE absent).

D. Rinne Test ❌

Compares air conduction (AC) vs. bone conduction (BC) but does not directly assess the occlusion effect.

Final Answer: ✅ C. Bing test (It is specifically designed to test for the occlusion effect).

Question 28: Tangible Reinforcement Operant Conditioning Audiometry (TROCA)

TROCA is a behavioral hearing test used for young children, typically 2 to 4 years old, who may not respond reliably to traditional pure-tone audiometry. It uses tangible rewards (e.g., tokens, toys, food) to reinforce correct responses.

Analysis of the Answer Choices:

A. Requires the child to press a button, then provides visual feedback ❌

TROCA uses tangible rewards (like small toys or food) rather than just visual feedback.

B. Easily results in undesirable false-negative responses ❌

TROCA is designed to increase motivation and minimize false negatives, making this statement incorrect.

C. Parents should not be in the room during the test ❌

Parents can be present to help the child feel comfortable, as long as they do not interfere with the testing process.

D. Uses tangible rewards like cookies, candies, and toys ✅ (Correct Answer)

TROCA is based on operant conditioning, where the child receives tangible reinforcement (small toys, food rewards) for correct responses.

Final Answer: ✅ D. TROCA is a hearing test that provides tangible rewards like cookies, candies, or toys.

Question 29: Low Birth Weight as a Hearing Risk Factor

Low birth weight is a known hearing risk factor in newborns. Premature infants and those with extremely low birth weight have a higher chance of sensorineural hearing loss (SNHL) due to:

Immature auditory system development

Hypoxia or perinatal complications

Ototoxic medication exposure

Neonatal infections (e.g., CMV, meningitis)

Analysis of the Answer Choices:

A. <1500 grams ✅ (Correct Answer)

The Joint Committee on Infant Hearing (JCIH) and WHO define very low birth weight (VLBW) as <1500 g, which is associated with a significantly higher risk of hearing loss.

B. <1800 grams ❌

No standard guidelines use this threshold for hearing risk categorization.

C. <2000 grams ❌

Although low birth weight (LBW) is <2500 g, the critical threshold for hearing risk is <1500 g.

D. <2200 grams ❌

This is considered moderate low birth weight, but it is not the standard for defining hearing risk.

Final Answer: ✅ A. <1500 grams (Very low birth weight is a significant risk factor for hearing loss).

Question 30: Hearing Assessment for Children with Developmental Delay

Children with developmental delays have a higher prevalence of hearing impairment, making accurate assessment essential. Testing must consider their cognitive, behavioral, and communication abilities.

Analysis of the Answer Choices:

A. Include middle ear function tests and otoacoustic emissions (OAE) ✅

Correct approach → Middle ear function (e.g., tympanometry) is crucial since otitis media is common in children with developmental delays. OAE tests cochlear function and is a standard screening tool.

B. Use descending approach (starting at high intensity and decreasing) ✅

Correct approach → A descending approach (starting at louder sounds and lowering intensity) helps maintain the child's attention and engagement.

C. Presenting 65 dB HL sound to check for startle reflex ❌ (Incorrect Answer)

Most inappropriate → The startle reflex (Moro reflex) is not a reliable hearing assessment for developmental delay because:

It only checks gross auditory response to loud sounds.

It does not assess frequency-specific thresholds needed for speech perception.

D. 25 dB HL as the passing threshold ✅

Correct approach → 25 dB HL is the standard threshold for normal hearing in behavioral audiometry for children.

Final Answer: ❌ C. Presenting 65 dB HL to check for a startle response is the least appropriate method.

Question 31: Behavioral Observation Audiometry (BOA) Analysis

Behavioral Observation Audiometry (BOA) is used for infants under 6 months who cannot provide conditioned responses to sound. Instead, clinicians observe reflexive responses to auditory stimuli, such as:

Startle reflex

Blinking

Sucking rate changes

Body or limb movement

Analysis of the Answer Choices:

A. 觀察的是制約化行為 (Observes conditioned behavior) ❌ (Incorrect Answer)

BOA does not rely on conditioned responses. Instead, it observes reflexive, unconditioned behaviors in response to sound.

Conditioned responses are used in Visual Reinforcement Audiometry (VRA) or Conditioned Play Audiometry (CPA), but not in BOA.

This is the correct answer because it is incorrect.

B. 個案容易產生習慣化現象 (Subjects easily habituate) ✅

Correct statement → Infants quickly habituate to repeated stimuli, making BOA less reliable over time.

C. 再測信度差 (Poor test-retest reliability) ✅

Correct statement → BOA has low test-retest reliability because infant responses are inconsistent and influenced by alertness, fatigue, and attention.

D. 有較高的受試者本身變異 (High subject variability) ✅

Correct statement → Infant responses vary greatly due to individual development, hearing sensitivity, and external factors (e.g., mood, hunger).

Final Answer: ❌ A. BOA observes conditioned behavior (Incorrect statement).

Question 32: Appropriate Stimulus for VRA (Visual Reinforcement Audiometry) Testing

Visual Reinforcement Audiometry (VRA) is used for infants around 6 months to 2.5 years old. It conditions the child to associate a sound with a visual reward (e.g., a toy animation).

AAA (2012) and Taiwan's Practice Recommendations:

The American Academy of Audiology (AAA, 2012) and Taiwan's clinical guidelines suggest starting with speech stimuli (broadband signal) before moving to pure tones.

Speech is a familiar and attention-grabbing sound, making it easier to condition the child before testing frequency-specific stimuli.

Analysis of Answer Choices:

A. 500 Hz → Not the best choice because low frequencies may be harder to detect in noisy environments.

B. 2 kHz → Important frequency but not the best starting stimulus.

C. 4 kHz → High frequencies are crucial for detecting noise-induced hearing loss, but not ideal for initial testing.

D. Speech stimuli → ✅ Correct answer

Speech sounds are the best initial stimulus because they engage infants more effectively.

After conditioning, pure tones (e.g., 2 kHz and 500 Hz) can be tested to assess frequency-specific hearing.

Final Answer: ✅ D. Speech stimuli

Question 33: Best Cross-Validation Tool for Hearing Threshold in Infants (<6 months old)

For infants younger than 6 months, behavioral observation audiometry (BOA) is commonly used. However, BOA is not very reliable, so objective tests are needed for cross-validation.

Analysis of Answer Choices:

A. Speech threshold test (SDT/SRT) → Not reliable for infants under 6 months since they cannot respond consistently to speech.

B. 1000 Hz tympanometry → Useful for assessing middle ear function, especially in newborns, but does not directly measure hearing threshold.

C. Auditory Brainstem Response (ABR) → ✅ Correct answer

Gold standard for newborn hearing assessment

Can estimate frequency-specific thresholds

Objective and reliable for infants under 6 months

D. Otoacoustic Emissions (OAE) → Useful for screening but does not measure hearing threshold directly. It only assesses cochlear function (outer hair cells).

Final Answer: ✅ C. Auditory Brainstem Response (ABR)

Question 34: Recommended Hearing Assessments for 13–18-Month-Olds (ASHA Guidelines)

For infants aged 13 to 18 months, the American Speech-Language-Hearing Association (ASHA) recommends a combination of behavioral, physiological, and middle ear function tests.

Key Considerations:

Visual Reinforcement Audiometry (VRA)

Primary behavioral test for this age group

Conditions infants to respond to sound by looking at a visual reward

Tympanometry

Evaluates middle ear function to detect issues like otitis media with effusion (OME)

Acoustic Reflex Testing (ARTs)

Ipsilateral ARTs are more commonly used in young infants

Contralateral ARTs are less reliable under 6 months but can be considered for older infants

Analysis of Answer Choices:

A. ①ABR, ④OAE, ⑥Contralateral ARTs → ❌ ABR and OAE are crucial for newborns but not the primary tests for this age group.

B. ①ABR, ④OAE, ⑤Ipsilateral ARTs → ❌ ABR is not prioritized at this age unless needed for specific cases (e.g., confirming sensorineural hearing loss).

C. ②VRA, ③Tympanometry, ⑥Contralateral ARTs → ✅ Correct answer

VRA is the primary test for behavioral hearing assessment.

Tympanometry ensures middle ear function is normal.

Contralateral ARTs can be useful for identifying neural dysfunction.

D. ②VRA, ③Tympanometry, ⑤Ipsilateral ARTs → ✅ Also a potentially correct option, but C is preferred due to the inclusion of contralateral ARTs, which ASHA recommends when applicable.

Final Answer: ✅ C. VRA, Tympanometry, Contralateral ARTs

Question 35: Best Hearing Assessment for a 7-Month-Old (JCIH 2007 Guidelines)

According to the Joint Committee on Infant Hearing (JCIH 2007), cross-checking multiple assessments is essential for pediatric hearing evaluations.

Key Considerations:

At 7 months, the infant can undergo behavioral and objective hearing assessments.

Visual Reinforcement Audiometry (VRA)

Gold standard for behavioral testing in infants older than 6 months

Auditory Brainstem Response (ABR)

Objective test to confirm hearing status, especially if there are risk factors or inconsistencies

Otoacoustic Emissions (OAE)

Checks cochlear (outer hair cell) function but does not assess neural pathways

Tympanometry

Evaluates middle ear function

Analysis of Answer Choices:

A. BO (Behavioral Observation) and VRA → ❌

BO is unreliable for this age group due to subject variability.

VRA is appropriate, but BO should be replaced with an objective test like ABR.

B. OAEs and Tympanometry → ❌

Good for screening but does not fully assess neural pathways or behavioral responses.

C. OAEs and Acoustic Reflexes → ❌

Does not provide behavioral hearing thresholds.

D. VRA and ABR → ✅ Correct answer

VRA for behavioral response

ABR for objective confirmation

Final Answer: ✅ D. VRA and ABR

Question 36: Behavioral Observation in One-Month-Old Infants

For newborns (around one month old), Behavioral Observation Audiometry (BOA) is used to detect involuntary responses to sound stimuli rather than conditioned responses, which develop in older infants.

Common Auditory Responses in Newborns:

A. Blink Reflex (眨眼反應) → ✅ Correct

Infants may blink in response to sudden loud sounds (acoustic startle response).

B. Startle Reflex / Moro Reflex (驚嚇反射) → ✅ Correct

Newborns often exhibit the Moro reflex (sudden extension of arms/legs) in response to loud sounds.

C. Turning Head Toward Sound Source (轉頭向聲源) → ❌ Incorrect

Head-turning behavior typically develops after 4–6 months of age, so it is not a reliable response in newborns.

D. Waking from Sleep Due to Sound (睡夢中被刺激音吵醒) → ✅ Correct

Sudden loud noises can wake a sleeping newborn, indicating auditory awareness.

Final Answer: ❌ C. Turning Head Toward Sound Source

Question 37: Clinical Application of Behavioral Observation Audiometry (BOA)

Behavioral Observation Audiometry (BOA) is mainly used to provide qualitative insights into an infant's auditory responses but has significant limitations in clinical settings.

Analysis of Answer Choices:

A. Can Be Used to Set Hearing Aids (可作為助聽輔具的設定依據) → ❌ Incorrect

BOA is not reliable or precise enough to determine hearing thresholds, making it unsuitable for programming hearing aids.

B. Can Be Used for Infant Hearing Screening (可用以施行嬰兒聽力篩檢) → ❌ Incorrect

Objective tests like OAE and ABR are the standard for newborn hearing screening, not BOA.

C. Can Be Used to Measure Hearing Thresholds (可用來測量聽覺閾值) → ❌ Incorrect

BOA does not provide precise hearing thresholds, as infant responses can be inconsistent and subjective.

D. Can Provide Important Clues About Infant Hearing Responses (對於嬰幼兒的聽覺反應可提供重要的線索) → ✅ Correct

BOA is useful for observing potential hearing issues but should be considered a supplementary tool rather than a definitive test.

Final Answer: ✅ D. Can Provide Important Clues About Infant Hearing Responses

Question 38: Description of Visual Reinforcement Audiometry (VRA)

Visual Reinforcement Audiometry (VRA) is commonly used for infants and toddlers, typically between 6 months and 2.5 years old, to assess their hearing thresholds using conditioned head-turning responses to auditory stimuli.

Analysis of Answer Choices:

① Infants Start Showing Listening Responses at 4–7 Months → ✅ Correct

At around 4–7 months, infants begin to exhibit a listening response (attentive reaction to sounds), which is a prerequisite for VRA.

② Preterm Infants Should Be Evaluated Based on Chronological (Not Corrected) Age → ❌ Incorrect

For preterm infants, we must consider their corrected age (adjusted for prematurity) rather than their chronological age to ensure developmental appropriateness.

③ Auditory Localization Develops Horizontally Before Vertically → ✅ Correct

Infants first develop horizontal sound localization (side-to-side turning) around 4-6 months, followed by vertical localization (up-down movement) later.

④ Besides Visual Reinforcement, Verbal Praise Can Also Be Used → ✅ Correct

Although visual reinforcement (e.g., animated toys, lights) is the primary reinforcement, verbal praise or social reinforcement can enhance responses.

Correct Answer: ✅ D. ①③④

Question 39: ASHA (1997) Guidelines for Play Audiometry in 3–5-Year-Old Children

Play Audiometry is used to test young children (3–5 years old) through engaging tasks (e.g., dropping a block in a bucket upon hearing a sound). According to ASHA (1997) guidelines, the test should include specific frequencies and pass criteria.

Analysis of Answer Choices:

500–4000 Hz, 15 dB HL → ❌ Incorrect

ASHA does not recommend including 500 Hz, as low-frequency noise in the test environment could lead to unreliable results.

500–4000 Hz, 20 dB HL → ❌ Incorrect

Same issue as above—500 Hz is not included in ASHA's recommended screening protocol.

1000–4000 Hz, 15 dB HL → ❌ Incorrect

Although the frequencies (1000–4000 Hz) are correct, the pass threshold should be 20 dB HL, not 15 dB HL.

1000–4000 Hz, 20 dB HL → ✅ Correct

ASHA (1997) recommends testing at 1000, 2000, and 4000 Hz at 20 dB HL for play audiometry screening.

Correct Answer: ✅ D. 1000–4000 Hz, 20 dB HL

Question 40: Hearing Protector Devices

Hearing protectors are essential in occupational and environmental noise exposure to prevent noise-induced hearing loss (NIHL). The main types of hearing protectors include:

Earplugs (inserted into the ear canal)

Semi-insert earplugs (held in place by a headband)

Earmuffs (covering the entire outer ear)

Analysis of Answer Choices:

A. Semi-insert earplugs provide the least attenuation and are suitable for intermittent noise exposure.

✅ Correct. Semi-insert earplugs (banded earplugs) offer less noise reduction than earplugs or earmuffs and are useful when quick and repeated removal is necessary (e.g., workers moving between noisy and quiet areas).

B. Wearing glasses reduces earmuff effectiveness by 10 dB; only earplugs should be used.

❌ Incorrect. While glasses do create gaps in the earmuff seal, the actual reduction is typically 5–8 dB, not necessarily 10 dB. Also, earmuffs can still be used effectively with proper adjustments, and double protection (earplugs + earmuffs) can compensate if needed.

C. For long-term wear, earmuffs are the most comfortable, followed by semi-insert, then earplugs.

❌ Incorrect. Comfort is subjective, but for extended wear, many people prefer foam earplugs due to their soft fit inside the ear canal. Earmuffs can become heavy or hot, especially in warm environments.

D. Compared to earplugs, earmuffs are better suited for situations where frequent removal is needed.

✅ Correct. Earmuffs are easier to remove and put back on quickly, making them ideal for jobs requiring frequent transitions between noisy and quiet areas. Earplugs require insertion into the ear canal, which takes longer and can be inconvenient.

Correct Answer: ✅ D. Earmuffs are better suited for environments where frequent removal is needed.

Question 41: Hearing Assessment for Hearing Aid Selection

Analysis of the Audiogram:

The right ear (右耳) has a severe to profound hearing loss (HTL 93 dB HL) across frequencies.

The left ear (左耳) has a mild to moderate hearing loss (HTL 41 dB HL) in the low and mid frequencies, with a sharper decline in the higher frequencies.

Bone conduction (BCL) results indicate a sensorineural component, and free field (FF) responses suggest a significant asymmetry between ears.

Evaluation of Answer Choices:

A. Left ear air-conduction threshold with masking

✅ Important: Since the right ear has severe loss, masking may be necessary to obtain accurate left ear thresholds when testing by air conduction. This helps in determining the true hearing levels for hearing aid fitting.

B. Bilateral bone conduction thresholds

✅ Important: Bone conduction testing confirms the type of hearing loss (sensorineural vs. conductive). If there is a conductive component, medical treatment might be needed before considering hearing aids.

C. Tone Auditory Brainstem Response (TABR) thresholds

❌ Least necessary: TABR is usually used to objectively estimate hearing thresholds when behavioral testing is unreliable (e.g., infants, malingerers). Since this audiogram already shows reliable air and bone conduction thresholds, TABR is not essential for hearing aid selection.

D. Consultation on current living environment

✅ Important: Understanding communication needs, background noise exposure, and daily activities is crucial for selecting an appropriate hearing aid style and features (e.g., noise reduction, directional microphones).

Correct Answer: ✅ C. TABR thresholds are the least necessary for hearing aid selection.

Question 42: Effects of Noise on Hearing

Evaluation of Answer Choices:

A. Low-frequency noise exposure causes greater threshold shifts than high-frequency noise exposure.

❌ Incorrect (Wrong Statement)

High-frequency noise (e.g., machinery, firearms) typically causes greater threshold shifts than low-frequency noise.

The cochlea is more vulnerable to high-frequency damage, as noise-induced hearing loss (NIHL) often starts at 3,000–6,000 Hz before affecting lower frequencies.

Low-frequency noise (e.g., traffic, industrial hum) can be disruptive but is less likely to cause significant auditory threshold shifts compared to high-frequency noise.

B. The maximum temporary threshold shift (TTS) occurs about half an octave above the noise exposure frequency.

✅ Correct

Research shows that TTS tends to peak around 0.5 octaves above the noise exposure frequency, due to cochlear mechanics and the traveling wave's peak displacement in the basilar membrane.

C. TTS is more likely to occur in individuals with pre-existing sensorineural hearing loss.

✅ Correct

Individuals with sensorineural hearing loss are more susceptible to further TTS, as their auditory system has reduced resilience to noise exposure.

D. Under a specific noise level, TTS reaches a maximum value called asymptotic threshold shift (ATS).

✅ Correct

ATS represents the maximum TTS that can be reached under prolonged noise exposure, meaning that after a certain period, the threshold shift no longer worsens.

Correct Answer: ✅ A. Low-frequency noise exposure does NOT cause greater threshold shifts than high-frequency noise.

Question 43: Possible Causes of Right Ear Tinnitus with Absent Acoustic Reflexes

Key Clinical Findings:

Right ear tinnitus

Mild sensorineural hearing loss (SNHL) in PTA

Absent acoustic reflex threshold (ART)

No middle ear infection, allergies, or nasal congestion

Evaluation of Answer Choices:

A. Cochlear pathology (耳蝸病變)

❌ Less likely

While cochlear damage can cause tinnitus and mild SNHL, it usually does not result in absent ART unless the hearing loss is moderate-severe.

B. Retrocochlear pathology (耳蝸後病變)

✅ Most likely

Retrocochlear lesions (e.g., vestibular schwannoma, auditory neuropathy) can cause:

Tinnitus

Mild SNHL

Absent ART (a key red flag for retrocochlear pathology)

Additional testing (MRI, ABR) is recommended.

C. Nasopharyngeal carcinoma (鼻咽癌)

❌ Less likely but possible

Nasopharyngeal carcinoma (NPC) can cause hearing loss and tinnitus if it compresses the Eustachian tube or auditory nerve, but it is typically associated with conductive hearing loss, not SNHL.

D. Psychosomatic disorder (身心症)

❌ Unlikely as the primary cause

While stress and psychological factors can worsen tinnitus, they do not explain the absent ART.

Correct Answer: ✅ B. Retrocochlear pathology (e.g., vestibular schwannoma, auditory neuropathy) is the most likely cause.

Question 44: Recommended Tests for Suspected Retrocochlear Pathology

Key Clinical Clues from the Previous Question (Q43):

Right ear tinnitus

Mild sensorineural hearing loss (SNHL)

Absent Acoustic Reflex Threshold (ART)

Possible retrocochlear pathology (e.g., vestibular schwannoma, auditory neuropathy)

Evaluation of Answer Choices:

A. PI-PB Function (Speech Recognition Test to Assess Roll-over Effect)

✅ Needed

PI-PB function helps determine if there is a rollover effect (i.e., a decline in word recognition scores at higher intensity levels).

Retrocochlear pathology often shows a significant rollover effect, making this test valuable.

B. Tone Decay Test

✅ Needed

This test assesses auditory nerve adaptation.

Retrocochlear lesions (e.g., vestibular schwannoma) typically cause a rapid decay in tone perception, making this an essential diagnostic tool.

C. TEN Test (Threshold Equalizing Noise Test)

❌ Not necessary

The TEN test is used to detect cochlear dead regions, which are more relevant in cases of sensorineural hearing loss due to cochlear pathology (e.g., severe hearing loss, presbycusis, ototoxicity).

Retrocochlear pathology does not typically involve cochlear dead regions, making this test unnecessary.

D. Neurodiagnostic Auditory Brainstem Response (ABR)

✅ Needed

ABR is a key test for detecting retrocochlear lesions, particularly vestibular schwannomas.

It evaluates waveform delays and interaural latency differences, helping to confirm neural dysfunction.

Correct Answer: ✅ C. TEN test is the least necessary for assessing suspected retrocochlear pathology.

Question 45: Otosclerosis – Identifying the Incorrect Statement

Evaluation of Answer Choices:

A. In later stages, mixed hearing loss is common.

✅ Correct statement

Otosclerosis begins as conductive hearing loss (CHL) due to stapes fixation.

In later stages, cochlear involvement leads to sensorineural hearing loss (SNHL), resulting in mixed hearing loss.

B. Typical onset is between the teenage years and 30s.

✅ Correct statement

Symptoms usually develop between 15–35 years old, though they can start earlier or later.

C. Both genetic and non-genetic factors contribute to its cause.

✅ Correct statement

Otosclerosis is partially hereditary (autosomal dominant inheritance with incomplete penetrance) but can also be influenced by hormonal, viral (e.g., measles), and environmental factors.

D. Males are twice as likely to develop otosclerosis as females.

❌ Incorrect statement

Females are actually more affected than males, with a 2:1 female-to-male ratio.

Hormonal factors (e.g., pregnancy, estrogen) may accelerate disease progression in women.

Correct Answer: ✅ D. The claim that men are twice as likely to have otosclerosis as women is incorrect.

Question 46:

Audiogram Interpretation:

Right Ear (O, □, unmasked bone conduction: ㄴ)

Air conduction thresholds show a significant air-bone gap, particularly in the low to mid frequencies (250 Hz to 2000 Hz).

This pattern is characteristic of conductive hearing loss in the mild to moderate range.

Left Ear (X, unmasked bone conduction: ㄴ)

Bone conduction and air conduction thresholds are within normal limits.

No significant air-bone gap is observed, suggesting no conductive hearing loss.

Evaluating Answer Choices:

(A) Left ear needs additional testing at 6000 Hz → Incorrect

The audiogram does not indicate a need for additional testing at 6000 Hz, as there is no significant high-frequency loss.

(B) Left ear at 4000 Hz requires masked bone conduction → Incorrect

Since the left ear's air and bone conduction thresholds are close, there is no air-bone gap, meaning masking is not required for bone conduction testing at 4000 Hz.

(C) Right ear air conduction at 250, 500, and 1000 Hz requires masking → Correct

A significant air-bone gap is present at low to mid frequencies in the right ear.

To accurately determine the degree of conductive hearing loss, masked air conduction testing is necessary.

(D) Right ear has mild to moderate conductive hearing loss → Correct

The air-bone gap in the right ear, with bone conduction thresholds within normal limits, strongly suggests conductive hearing loss in the mild to moderate range.

Best Answer: Both C and D are correct. However, if only one answer is allowed, D is the best choice, as it correctly describes the nature of the hearing loss.

Final Answer: D (Right ear has mild to moderate conductive hearing loss).

Question 47:

Audiogram Analysis:

Right Ear (○, solid line)

Shows a moderate to severe sensorineural hearing loss (SNHL), with thresholds worsening at higher frequencies.

Bone conduction (BCL) results are consistent with air conduction, confirming sensorineural involvement.

Left Ear (□, dashed line)

Shows a profound hearing loss, particularly at higher frequencies.

Bone conduction results indicate a mixed or conductive component.

Evaluating Answer Choices:

(A) Unmasked SRT: Right Ear 45 dB, Left Ear 70 dB → Incorrect

The right ear exhibits hearing loss that is more severe than 45 dB, so an SRT of 45 dB is unlikely.

The left ear has profound hearing loss, meaning an SRT of 70 dB is too optimistic.

(B) Tympanometry (Type A in both ears) → Incorrect

The left ear shows a significant conductive component, which suggests that Type A tympanometry (normal middle ear function) is unlikely.

A Type B or Type C result would be more consistent with conductive involvement.

(C) TABR: Right Ear 40 dB, Left Ear 90 dB → Correct

The right ear has a moderate-to-severe SNHL, making a TABR threshold of 40 dB plausible.

The left ear has profound hearing loss, making a TABR threshold of 90 dB realistic.

(D) Speech Discrimination Scores (SDS) at 70% in both ears → Incorrect

The left ear has profound hearing loss, so a high SDS score is unlikely.

SDS for profound loss is generally very poor or even absent.

Best Answer: ✅ (C) TABR: Right Ear 40 dB, Left Ear 90 dB.

This matches the audiogram findings the most accurately.

Question 48: Understanding Masking in Pure-Tone Audiometry with Supra-Aural Headphones

Masking is required to prevent the non-test ear from detecting sounds presented to the test ear, which can occur due to crossover hearing.

With supra-aural headphones, interaural attenuation (IA) is around 40 dB. This means if a sound presented to one ear is at least 40 dB louder than the bone conduction threshold of the other ear, it could be heard by the non-test ear, necessitating masking.

Evaluating Answer Choices:

(A) Test ear air-conduction (AC) threshold is 40 dB or more above the non-test ear bone-conduction (BC) threshold → Correct

This follows the standard masking rule: ACtest ear−BCnon-test ear≥40 dBAC_{\text{test ear}} - BC_{\text{non-test ear}} \geq 40 \text{ dB} ACtest ear −BCnon-test ear ≥40 dB

This situation indicates that the non-test ear might be detecting the tone through crossover, requiring masking.

(B) Both air-conduction thresholds differ by less than 40 dB → Incorrect

If the difference is less than 40 dB, there is no risk of crossover hearing, so masking is not required.

(C) Both bone-conduction thresholds are the same → Incorrect

The need for masking depends on air-bone gaps and interaural attenuation, not whether bone-conduction thresholds are identical.

(D) Test ear AC threshold is the same as non-test ear BC threshold → Incorrect

Masking is needed only when the test ear AC threshold is at least 40 dB higher than the non-test ear BC threshold.

If they are equal, crossover is unlikely, so masking is not necessary.

Correct Answer:

✅ (A) Test ear air-conduction threshold is 40 dB or more above the non-test ear bone-conduction threshold.
This follows the standard masking rule for supra-aural headphones to prevent crossover hearing.

Question 49:

To determine the optimal initial masking level at 2000 Hz using Turner's (2004) optimized masking method, we follow these steps:

Step 1: Identify the Key Thresholds

From the audiogram:

Left Ear (Test Ear) Air-Conduction (AC) Threshold at 2000 Hz ≈ 60 dB HL

Right Ear (Non-Test Ear) Bone-Conduction (BC) Threshold at 2000 Hz ≈ 10 dB HL

Interaural Attenuation (IA) for TDH-49 Headphones ≈ 40 dB

Step 2: Apply the Initial Masking Formula

Turner's optimized masking method suggests the initial masking level should be calculated as:

Initial Masking Level=Non-Test Ear BC+10 dB\text{Initial Masking Level} = \text{Non-Test Ear BC} + 10 \text{ dB}Initial Masking Level=Non-Test Ear BC+10 dB =10+10=20 dB HL= 10 + 10 = 20 \text{ dB HL}=10+10=20 dB HL

Since 20 dB is not an answer choice, let's consider the closest reasonable option.

Step 3: Check the Given Answer Choices

A) 5 dB → Too low, insufficient to prevent crossover.

B) 10 dB → Still lower than recommended masking level.

C) 45 dB → Higher than required for initial masking.

D) 65 dB → Excessive masking, leading to overmasking risks.

Step 4: Select the Best Answer

Since 10 dB is the closest answer to 20 dB in the provided choices, option B (10 dB HL) is the best choice.

✅ Correct Answer: B) 10 dB

Question 50: To determine whether speech masking is needed, let's analyze the given data step by step.

Step 1: Identify Key Values

Right Ear Speech Recognition Threshold (SRT): 0 dB HL

Left Ear Speech Recognition Threshold (SRT): 50 dB HL

Interaural Attenuation (IA) for TDH-49 Headphones: 40 dB HL

Best Bone-Conduction Threshold (BBC) of Right Ear: Assumed ~10 dB HL

Best Bone-Conduction Threshold (BBC) of Left Ear: Assumed ~50 dB HL

Step 2: Determine the Need for Masking

Speech masking is required when:

Test Ear SRT−Non-Test Ear BBC≥IA\text{Test Ear SRT} - \text{Non-Test Ear BBC} \geq \text{IA}Test Ear SRT−Non-Test Ear BBC≥IA

For the left ear (test ear) speech:

50 dB HL−10 dB HL=40 dB HL50 \text{ dB HL} - 10 \text{ dB HL} = 40 \text{ dB HL}50 dB HL−10 dB HL=40 dB HL

Since 40 dB HL (IA for TDH-49) is the cutoff, we check if it meets or exceeds IA.

If the difference is greater than or equal to IA, masking is needed.

Here, 40 dB HL meets IA, so masking is required for the right ear.

Step 3: Choose the Correct Answer

A) Incorrect – The right ear needs masking because the difference meets IA.

B) Incorrect – The SRT difference meets IA, so masking is needed.

C) Correct – The right ear requires masking because Left SRT – Right BBC ≥ IA (50 - 10 = 40 dB HL).

D) Incorrect – Masking decision is based on the better bone-conduction threshold of the non-test ear, not the test ear itself.

✅ Correct Answer: C) The right ear requires masking because the left ear's speech presentation level minus the right ear's best bone conduction threshold is greater than or equal to the IA value.

3106

Question 1: Tympanogram Gradient and Middle Ear Effusion

The tympanogram gradient refers to the steepness or sharpness of the peak in a tympanogram, which helps in assessing middle ear function. A lower gradient suggests a broader or flatter tympanogram, often associated with middle ear effusion (MEE).

A) <0.2 → Correct. A gradient less than 0.2 is strongly correlated with middle ear effusion, indicating poor middle ear compliance due to fluid accumulation.

B) 0.4–0.6 → Incorrect. This range represents a normal tympanogram.

C) >235 → Incorrect. This value is irrelevant because gradient measurements are typically between 0 and 1.

D) 51–114 → Incorrect. This range is more relevant to tympanometric peak pressure rather than gradient.

✅ Correct Answer: A) <0.2

Question 2: Admittance and Middle Ear Pathologies

Admittance (Y) refers to how easily sound passes through the middle ear system. The higher the admittance, the less resistance in the system.

A) Tympanic Membrane Perforation → Correct. When the eardrum is perforated, sound can pass more freely into the middle ear, leading to increased admittance.

B) Healed Tympanic Membrane Perforation → Incorrect. After healing, the eardrum regains its normal stiffness, reducing overall admittance.

C) Cerumen (Earwax) Blockage → Incorrect. Earwax in the external canal increases impedance, lowering admittance.

D) Middle Ear Effusion → Incorrect. Fluid in the middle ear restricts movement of the tympanic membrane and ossicles, reducing admittance.

✅ Correct Answer: A) Tympanic Membrane Perforation

Question 3: Tympanogram Type and Probe Tone Sound Pressure Level (SPL)

The probe tone sound pressure level (SPL) in the ear canal is highest when the middle ear system is the most resistant (least compliant) to sound transmission. This occurs when the tympanic membrane is stiff, preventing sound from efficiently passing through.

A) Type A → Incorrect. A normal tympanogram (Type A) represents normal middle ear function with balanced compliance, leading to moderate SPL in the ear canal.

B) Type B → Correct. Type B tympanograms (flat curve) indicate fluid in the middle ear (effusion) or a perforation with large volume, causing sound to be mostly reflected back into the ear canal, resulting in the highest SPL in the canal.

C) Type C → Incorrect. This type represents negative middle ear pressure (e.g., eustachian tube dysfunction) but still allows some sound to pass through, resulting in moderate SPL.

D) Type Ad → Incorrect. A hypermobile tympanic membrane (Type Ad) allows sound to pass more freely, reducing SPL in the ear canal.

✅ Correct Answer: B) Type B

Question 4: Acoustic Reflex Testing

Acoustic reflex testing evaluates the contraction of the stapedius muscle in response to loud sounds, which helps diagnose middle ear, cochlear, and retrocochlear disorders.

A) Includes acoustic reflex threshold (ART) and acoustic reflex decay testing → Correct. ART measures the softest sound that triggers the reflex, while reflex decay assesses whether the contraction is sustained over time.

B) Normal ART values range from 85–100 dB SPL → Correct. This range is typical for individuals with normal hearing.

C) Reflex decay testing assesses the increase in reflex response, with a 50% increase considered abnormal → Incorrect. Reflex decay actually measures the decrease in response over time, not an increase. A 50% decrease within 5 seconds at 500 or 1000 Hz is considered abnormal, suggesting retrocochlear pathology.

D) Measures stapedius muscle response to sound → Correct. The stapedius muscle contracts in response to loud sounds, reducing ossicular chain movement to protect the inner ear.

✅ Correct Answer: C) Reflex decay testing does not measure an increase in reflex response; it assesses a decrease over time.

Question 5: Acoustic Reflex Principles and Applications

Acoustic reflex (AR) is the involuntary contraction of the stapedius muscle in response to loud sounds, which reduces the transmission of sound energy to the cochlea.

A) The stimulus sound does not trigger a contralateral acoustic reflex → Incorrect. The acoustic reflex is bilateral, meaning a loud sound in one ear will trigger reflexes in both the ipsilateral and contralateral ears.

B) Acoustic reflex is typically detected when admittance increases by 0.01 mmhos → Correct. In clinical practice, an increase of at least 0.02 mmhos is often considered significant, but 0.01 mmhos is sometimes used as a minimum criterion.

C) When stimulus intensity increases, the tympanic membrane admittance change due to the acoustic reflex also increases → Correct in principle, but not the best choice. While reflex strength can increase with higher intensity, the relationship is not always linear, as reflex decay and neural adaptation affect responses at high levels.

D) The tensor tympani muscle contributes much more to middle ear impedance than the stapedius muscle → Incorrect. The stapedius muscle is the primary contributor to acoustic reflexes, while the tensor tympani has a smaller role and is not as functionally significant in acoustic reflex measurements.

✅ Correct Answer: B) Acoustic reflex is typically determined when admittance increases by at least 0.01 mmhos.

Question 6: Most Inappropriate Test Combination for Diagnosing Vestibular Schwannoma (Acoustic Neuroma)

Vestibular schwannoma (acoustic neuroma) is a retrocochlear disorder affecting the auditory nerve. The ideal diagnostic approach includes tests that assess neural function and speech perception in noise.

A) Pure-tone audiometry + tone decay test → Appropriate.

Tone decay helps identify retrocochlear pathology by testing whether a patient can sustain a pure tone over time without needing an intensity increase.

B) Middle ear function + acoustic reflex decay → Appropriate.

Acoustic reflex decay tests for rapid weakening of the reflex, which is a hallmark of retrocochlear pathology.

C) Speech audiometry + PI-PB (performance-intensity for phonetically balanced words) test → Appropriate.

PI-PB rollover (a decrease in word recognition at high intensities) is a key sign of neural dysfunction.

D) Electrocochleography (ECochG) + masking level difference (MLD) → Inappropriate.

ECochG measures cochlear potentials, which are typically normal in vestibular schwannoma.

Masking level difference (MLD) evaluates brainstem processing of binaural cues, which is not a primary test for retrocochlear pathology.

✅ Correct Answer: D) Electrocochleography (ECochG) + masking level difference (MLD) are not suitable for diagnosing vestibular schwannoma.

Question 7: Acoustic Reflex Decay Test

Acoustic Reflex Decay (ARD) is a clinical test used to assess retrocochlear dysfunction, particularly for vestibular schwannoma (acoustic neuroma). It measures whether the stapedius muscle contraction sustains over time when exposed to a prolonged loud sound.

A) Can record both ipsilateral and contralateral acoustic reflex decay responses → ✅ Correct.

Acoustic reflex decay can be tested in both ears, using either an ipsilateral (same ear) or contralateral (opposite ear) stimulus.

B) Test frequencies are typically 2000 Hz and 4000 Hz → ❌ Incorrect.

The standard clinical test uses 500 Hz and 1000 Hz because high frequencies (2000 Hz, 4000 Hz) are less reliable for detecting retrocochlear pathology.

C) Test intensity is typically 10 dB below the reflex threshold → ❌ Incorrect.

The correct intensity for ARD testing is 10 dB above the acoustic reflex threshold (ART), not below.

D) Commonly used to detect cochlear pathology → ❌ Incorrect.

ARD is primarily used for retrocochlear disorders (e.g., vestibular schwannoma), not cochlear dysfunction.

✅ Correct Answer: A) Can record both ipsilateral and contralateral acoustic reflex decay responses.

Question 8: Suppression Testing in Transient Evoked Otoacoustic Emissions (TEOAE)

TEOAE suppression testing assesses the medial olivocochlear (MOC) reflex, which helps in modulating cochlear responses by inhibiting outer hair cell activity.

A) Contralateral suppressor stimuli and ipsilateral stimulus cannot be applied simultaneously (simultaneous masking) → ❌ Incorrect.

Simultaneous masking is commonly used in suppression tests.

B) Contralateral suppressor stimulus and ipsilateral stimulus should use backward masking → ❌ Incorrect.

Backward masking (applying the suppressor sound after the stimulus) is not standard for TEOAE suppression.

C) Binaural suppression is less effective than contralateral suppression → ❌ Incorrect.

Binaural suppression is typically stronger than contralateral suppression because both medial olivocochlear (MOC) pathways are activated.

D) Ipsilateral suppressor stimulus and test stimulus should use forward masking → ✅ Correct.

Forward masking (applying the suppressor stimulus before the test stimulus) is used to test MOC function effectively.

✅ Correct Answer: D) Ipsilateral suppressor stimulus and test stimulus should use forward masking.

Question 9: Analysis of the Given Diagram

The diagram illustrates a probe system used in otoacoustic emission (OAE) testing. It includes:

Stimulus sound → Delivered into the ear canal.

Probe assembly → Comprising a receiver (microphone) to capture emissions and a speaker to present the stimulus.

Amplifier and filter → Processes recorded signals.

Analysis system (Averager) → Interprets results.

This setup is commonly used for evoked OAEs, such as:

Transient-Evoked Otoacoustic Emissions (TEOAE)

Distortion Product Otoacoustic Emissions (DPOAE)

Answering the Question: Which OAE Cannot Be Measured?

A) Spontaneous Otoacoustic Emissions (SOAE) → ✅ Correct (This Cannot Be Measured)

SOAEs are measured without an external stimulus, but this system has a stimulus generator.

This indicates the system is designed for evoked emissions rather than spontaneous emissions.

B) Transient-Evoked Otoacoustic Emissions (TEOAE) → ❌ Incorrect (Can Be Measured)

TEOAEs require a click or tone burst stimulus, which is present in the system.

C) Distortion Product Otoacoustic Emissions (DPOAE) → ❌ Incorrect (Can Be Measured)

DPOAEs are evoked using two simultaneous pure-tone stimuli (f1, f2), which can be delivered by the system.

D) Stimulus Frequency Otoacoustic Emissions (SFOAE) → ❌ Incorrect (Can Be Measured)

SFOAEs require a continuous pure-tone stimulus, which this system is capable of providing.

Final Answer:

✅ A) Spontaneous Otoacoustic Emissions (SOAE) cannot be measured with this system.

Question 10: DPOAE Frequency Calculation

Formula for the strongest DPOAE component:

fDP=2f1−f2f_{DP} = 2f_1 - f_2fDP =2f1 −f2

Given:

f1=1000f_1 = 1000f1 =1000 Hz

f2=1200f_2 = 1200f2 =1200 Hz

fDP=2(1000)−1200=2000−1200=800 Hzf_{DP} = 2(1000) - 1200 = 2000 - 1200 = 800 \text{ Hz}fDP =2(1000)−1200=2000−1200=800 Hz

Correct Answer: ✅ B) 800 Hz

Question 11: Cochlear Structure Most Related to OAE

Otoacoustic emissions (OAE) are primarily generated by outer hair cells (OHCs) in the cochlea. These cells amplify sound signals via their electromotility, enhancing cochlear sensitivity and frequency selectivity. Damage to the outer hair cells significantly reduces or eliminates OAE responses.

A) Vestibular aqueduct → ❌ Unrelated to OAE; involved in inner ear fluid balance.

B) Inner hair cells → ❌ Primarily responsible for sending auditory signals to the brain but do not produce OAE.

C) Outer hair cells → ✅ Correct! These generate OAEs through their active movement.

D) Stria vascularis → ❌ Important for cochlear ion homeostasis but not directly responsible for OAE.

Correct Answer: ✅ C) Outer Hair Cells

Question 12: Otoacoustic Emission (OAE) Characteristics

A) Adults have larger OAE amplitudes than infants → ❌ Incorrect

Infants generally have higher OAE amplitudes than adults due to their smaller ear canal volume, which enhances recorded emissions.

B) High-frequency OAE has a longer latency than low-frequency OAE → ✅ Correct

Latency (response delay) is inversely related to frequency.

High-frequency OAEs originate from the basal cochlea and have shorter travel distances → shorter latency.

Low-frequency OAEs originate from the apical cochlea, requiring longer travel time → longer latency.

C) Males have larger OAE amplitudes than females → ❌ Incorrect

Studies show females typically have larger OAE amplitudes than males, possibly due to hormonal or structural differences in cochlear mechanics.

D) Ipsilateral OAE suppression is greater than contralateral suppression → ❌ Incorrect

Contralateral (opposite ear) suppression of OAEs is generally stronger due to medial olivocochlear (MOC) pathway involvement.

✅ Correct Answer: B) High-frequency OAE latency is longer than low-frequency OAE latency.

Question 13: Efferent Pathway Components for OAE Suppression

The efferent auditory pathway involved in OAE suppression includes:

Cochlear nucleus (CN) → ✅ Part of the pathway

Superior olivary complex (SOC) → ✅ Major component of efferent control

Medial geniculate body (MGB) → ✅ Part of central auditory processing but not directly involved in OAE suppression

Superior colliculus (SC) → ❌ Incorrect (not part of the efferent auditory pathway)

The SC is a visual processing structure in the midbrain, not a primary component of the auditory efferent system.

✅ Correct Answer: A) Superior colliculus (SC)

Question 14: Clinical Purpose of Bone-Conducted Click-Evoked ABR

Correct Answer: D. 評估傳導性聽力損失之可能 (Evaluate the possibility of conductive hearing loss)

Explanation:

Bone-conducted click-evoked auditory brainstem response (ABR) is primarily used in audiology to assess conductive hearing loss by bypassing the outer and middle ear and directly stimulating the cochlea. If air-conduction ABR results suggest hearing loss but bone-conduction ABR shows normal responses, it indicates a conductive component (e.g., middle ear pathology such as otitis media or ossicular dysfunction).

Option A (Estimate specific frequency bone conduction hearing threshold): Incorrect because click stimuli do not provide frequency-specific information; tone bursts or chirps are better for this purpose.

Option B (Confirm air-conduction ABR threshold accuracy): Incorrect because bone conduction ABR is not primarily used to validate air-conduction results but rather to differentiate between sensorineural and conductive loss.

Option C (More accurately estimate 500 Hz pure-tone threshold than air conduction click ABR): Incorrect because click-evoked ABR is broadband and not well-suited for low-frequency assessment.

Question 15: ECochG and Auditory Neuropathy Diagnosis

Correct Answer: D. 耳蝸麥克風在密波及疏波極性短聲刺激下的表現 (Cochlear microphonic response under condensation and rarefaction stimuli)

Explanation:

Electrocochleography (ECochG) is useful in diagnosing auditory neuropathy spectrum disorder (ANSD) by analyzing the cochlear microphonic (CM) response. A key indicator of auditory neuropathy is the persistence of the cochlear microphonic across alternating polarities (condensation and rarefaction), suggesting normal outer hair cell function but impaired neural transmission.

Option A (Action potential behavior under condensation and rarefaction stimuli): Incorrect because ANSD typically affects neural responses, not action potentials specifically.

Option B (Summating potential under alternating polarity stimuli): Incorrect because summating potential is more related to cochlear mechanics (e.g., Ménière's disease) rather than auditory neuropathy.

Option C (Cochlear microphonic response consistency with increasing stimulus rate): Incorrect because cochlear microphonic does not show significant rate-dependent changes in ANSD, whereas neural responses like ABR do.

Thus, observing CM responses under different polarity stimuli is the most relevant diagnostic marker for auditory neuropathy.

Question 16: Effect of Increasing Modulation Frequency in ASSR

Correct Answer: A. 潛時縮短 (Latency shortens)

Explanation:

Auditory Steady-State Response (ASSR) uses amplitude or frequency-modulated tones to assess hearing thresholds objectively. The modulation frequency determines how the brain processes the auditory stimulus.

Increasing modulation frequency from 40 Hz to 80 Hz results in:

Shorter response latency because higher modulation frequencies activate faster neural synchrony in the brainstem.

More brainstem involvement (80 Hz responses originate primarily from the brainstem, while 40 Hz responses involve the auditory cortex).

Reduced response amplitude and phase coherence at very high modulation rates (above 80-100 Hz).

Option B (Phase coherence decreases): Incorrect because 80 Hz is still within an optimal range for brainstem responses, and phase coherence typically remains stable up to this point.

Option C (No effect on threshold estimation): Incorrect because modulation frequency can affect response detection, potentially influencing threshold estimations at extreme values.

Option D (Amplitude increases): Incorrect because higher modulation frequencies generally reduce response amplitudes due to decreased neural synchrony.

Thus, increasing the modulation frequency leads to shorter response latency while maintaining reasonable phase coherence.

Question 17: Enhancing ABR Wave Amplitudes

Correct Answer: D. 使用變頻音刺激（chirp stimuli）能集中刺激高頻的低閾值神經元 (Using chirp stimuli can focus on high-frequency, low-threshold neurons) → Most inappropriate statement

Explanation:

Wave amplitude in Auditory Brainstem Response (ABR) can be enhanced through various methods that increase neural synchrony and recruitment of auditory neurons.

Option A (Increasing stimulus intensity depolarizes more neurons): Correct. Higher intensity sounds recruit more auditory nerve fibers, leading to larger response amplitudes.

Option B (Moving the reference electrode closer to the cochlea enhances Wave I): Correct. Wave I is generated by the auditory nerve, so positioning electrodes near the cochlear promontory or ear canal enhances its detection.

Option C (Lowering stimulus rate enhances amplitude): Correct. Lower stimulus rates prevent neural adaptation, improving the robustness of response amplitudes.

Option D (Chirp stimuli enhance high-frequency, low-threshold neurons): Incorrect (most inappropriate) because:

Chirp stimuli are designed to compensate for the cochlear traveling wave delay, enhancing low-frequency responses, not high frequencies.

Instead of focusing on low-threshold neurons, chirp stimuli improve synchrony across all frequencies by aligning different frequency components over time.

Thus, the least appropriate statement is Option D, as chirp stimuli do not specifically enhance high-frequency, low-threshold neurons, but rather broaden neural synchrony across the cochlea.

Question 18: ABR Testing Methods – Most Inappropriate Statement

Correct Answer: B. 2 個月大新生兒骨導的耳間衰減值約為 0 dB，故需進行遮蔽 (The interaural attenuation (IA) for bone conduction in a 2-month-old infant is approximately 0 dB, so masking is required).

Explanation:

Auditory Brainstem Response (ABR) testing involves measuring electrical activity in the auditory pathway in response to sound stimuli. Different factors influence ABR test accuracy, including stimulus type, earphone selection, and interaural attenuation (IA) values.

Option A (As stimulus intensity decreases, latency increases significantly): Correct. Lower intensity sounds result in longer neural conduction times, increasing wave latencies.

Option C (Insert earphones reduce the need for masking): Correct. Insert earphones provide greater interaural attenuation (IA) (~50-60 dB), reducing cross-hearing and the need for masking compared to supra-aural headphones (~40 dB IA).

Option D (Alternating polarity stimuli reduce stimulus artifact and noise): Correct. Alternating between condensation and rarefaction clicks reduces electrical artifacts from transducers and improves ABR waveform clarity.

Option B (IA for bone conduction in infants is 0 dB, requiring masking): Incorrect (most inappropriate).

While bone conduction IA is lower in infants than in adults, it is not necessarily 0 dB across all conditions.

In early infancy, cranial sutures are not fully fused, leading to greater sound transmission between ears, but clinical masking is not always required unless cross-hearing is suspected.

Bone conduction ABR is often used without masking, as cross-hearing is less of a concern in cases of bilateral hearing loss.

Thus, Option B is the most inappropriate statement due to the overgeneralization that masking is always needed for bone conduction ABR in infants.

Question 19: Distinguishing SP (Summating Potential) and AP (Action Potential) in Electrocochleography (ECochG) – Most Inappropriate Statement

Correct Answer: B. 提高刺激速率至 100 Hz (Increasing the stimulus rate to 100 Hz).

Explanation:

Electrocochleography (ECochG) is a diagnostic test that measures cochlear potentials, particularly Summating Potential (SP) and Action Potential (AP), to assess inner ear function and diagnose disorders like Ménière's disease or auditory neuropathy.

To better differentiate SP from AP:

Option A (Using longer-duration tone bursts): Correct. Longer-duration stimuli enhance the SP component, making it easier to distinguish from AP.

Option C (Changing stimulus polarity at low intensities): Correct. This helps separate cochlear microphonics (CM) from SP/AP, improving diagnostic accuracy.

Option D (Lowering stimulus intensity to below 60 dB nHL): Correct. Lower intensities reduce neural recruitment, emphasizing SP over AP, which helps with separation.

Option B (Increasing stimulus rate to 100 Hz): Incorrect (most inappropriate).

Higher stimulus rates tend to reduce AP amplitude due to neural fatigue.

SP/AP differentiation becomes more difficult because higher rates blur transient responses, making the AP component harder to detect.

Typically, moderate stimulus rates (~7-11 Hz) are used in ECochG to balance temporal resolution and neural synchrony.

Thus, Option B is the most inappropriate statement as increasing the stimulus rate hinders rather than enhances the distinction between SP and AP.

Question 20: ABR I-III Normal, IV-V Delayed – Most Likely Dysfunctional Area

Correct Answer: C. 喙部腦幹（rostral brainstem）

Explanation:

Auditory Brainstem Response (ABR) waveforms represent neural activity along the auditory pathway:

Wave I → Distal Auditory Nerve (Cochlear Nerve)

Wave II → Proximal Auditory Nerve

Wave III → Cochlear Nucleus (Lower Brainstem)

Wave IV → Superior Olivary Complex

Wave V → Lateral Lemniscus / Inferior Colliculus (Rostral Brainstem)

If I-III interwave latency is normal, it suggests normal function of the auditory nerve and cochlear nucleus. However, a prolonged IV-V interwave latency indicates dysfunction in the upper brainstem regions, particularly:

Lateral lemniscus

Inferior colliculus

Thus, the most likely site of abnormality is the rostral brainstem (C).

Option A (Auditory nerve dysfunction): Incorrect, because Wave I-III latencies are normal, meaning the auditory nerve is functioning properly.

Option B (Cochlear nucleus dysfunction): Incorrect, since Wave III latency is normal, indicating no significant pathology in the cochlear nucleus.

Option D (Severe head injury): Not the best answer, as severe brain injury would typically cause more widespread ABR abnormalities, not just selective IV-V latency prolongation.

Question 21: Best ABR Testing Method Statement

Correct Answer: B. 變頻音（chirp）利用時間代償減少行波由高頻至低頻區之時間差，可使反應振幅增強

Explanation:

ABR testing methods can be optimized using different stimuli and recording techniques:

Option A (Insert earphones reduce interaural attenuation): Incorrect. Insert earphones actually increase interaural attenuation (IA) (~50-60 dB) compared to supra-aural headphones (~40 dB), making cross-hearing less likely and reducing the need for masking.

Option C (Click-generated traveling wave speeds up at the cochlear apex, called temporal dispersion): Incorrect.

The cochlear traveling wave slows down as it moves from base (high-frequency region) to apex (low-frequency region).

"Temporal dispersion" refers to the time lag between high and low-frequency activation, but it doesn't mean the wave accelerates at the apex.

Option D (Bone conduction ABR energy is focused at 500-1000 Hz and can exceed 60 dB HL): Incorrect.

Bone conduction ABR stimuli are typically limited to ≤ 50 dB HL to avoid skull distortions and non-auditory responses.

Energy is indeed strongest around 500-1000 Hz, but the output rarely exceeds 60 dB HL due to calibration and equipment limits.

Option B (Chirp stimuli enhance response amplitude by compensating for traveling wave delay): Correct.

Chirp stimuli are designed to compensate for cochlear traveling wave delays, ensuring that low- and high-frequency regions are stimulated simultaneously.

This leads to stronger neural synchrony and enhanced ABR wave amplitudes, making them ideal for threshold estimation.

Thus, Option B is the most appropriate statement regarding ABR testing methods.

Question 22: Typical Auditory Neuropathy (AN) Characteristics

Correct Answer: B. 聽性腦幹反應消失，耳聲傳射卻正常

Explanation:

Auditory Neuropathy Spectrum Disorder (ANSD) is characterized by impaired neural transmission of auditory signals despite normal outer hair cell function. This leads to:

Absent or severely abnormal Auditory Brainstem Response (ABR) → Due to dysfunction at the auditory nerve or synapses.

Normal Otoacoustic Emissions (OAE) → Because outer hair cells remain functional.

Evaluating the answer choices:

Option A (Normal ABR & Normal OAE) → Incorrect. ANSD patients have abnormal ABR, making this incorrect.

Option B (Absent ABR, Normal OAE) → Correct. This pattern is the hallmark of auditory neuropathy, confirming neural transmission issues while preserving outer hair cell function.

Option C (Normal ABR, Absent OAE) → Incorrect. This pattern suggests outer hair cell dysfunction (e.g., cochlear damage), not ANSD.

Option D (Absent ABR & Absent OAE) → Incorrect. This suggests severe cochlear damage or combined pathology, which is not typical of ANSD.

Thus, option B is the most appropriate answer.

Question 23: Least Appropriate Use of Auditory Evoked Potentials

Correct Answer: D. 不匹配負波（MMN）可使用與 ABR 相同之參數測量

Explanation:

Different types of auditory evoked potentials (AEPs) have distinct protocols, applications, and stimulus parameters.

Evaluating the answer choices:

Option A (ABR can be used in sleeping patients) → Correct and appropriate.

ABR is a brainstem-level response and is not affected by sleep, making it ideal for newborn hearing screening and objective auditory assessments.

Option B (LLR can use speech to assess higher-level auditory processing) → Correct and appropriate.

Late Latency Responses (LLR) originate from the auditory cortex and are useful in evaluating speech perception, auditory attention, and cognitive processing.

Option C (MLR can be used for diagnosing Auditory Processing Disorder (APD), but findings are inconclusive) → Correct and appropriate.

Middle Latency Response (MLR) reflects thalamocortical activity and has been explored for APD diagnosis, but its clinical use remains debated.

Option D (MMN can be recorded using ABR parameters) → Incorrect.

Mismatch Negativity (MMN) is a cortical response to sound pattern changes (e.g., frequency, duration).

It requires different stimulus paradigms than ABR, such as longer inter-stimulus intervals (ISI) and oddball paradigms to detect automatic auditory discrimination.

ABR uses clicks or tone bursts at high repetition rates, which are inappropriate for MMN.

Thus, option D is the least appropriate answer.

Question 24: Effects of Stimulation Rate on ABR

Correct Answer: C. 刺激速率加快，振幅變大 (Increasing the stimulus rate increases amplitude) – Incorrect statement

Explanation:

The effects of increasing stimulus rate in ABR testing include:

Increased latency (delayed wave peaks) → True (A & D are correct)

Higher stimulus rates increase neural fatigue and synaptic delay, resulting in longer wave latencies, especially for wave V.

Reduced amplitude → True (C is incorrect)

Faster rates reduce neural synchrony, leading to decreased wave amplitudes, particularly in early waves (I & III).

Wave V is more stable than Wave I → True (B is correct)

Wave V remains more robust than Wave I even at high rates, making it the most commonly used clinical marker in ABR.

Evaluating answer choices:

A (Higher stimulus rate → Increased latency) → ✅ Correct

B (Wave V is less affected in amplitude than Wave I) → ✅ Correct

C (Higher stimulus rate → Increased amplitude) → ❌ Incorrect (Higher rate reduces amplitude due to neural desynchronization)

D (Wave V latency is more affected than Wave I latency) → ✅ Correct

Thus, option C is the incorrect statement.

Question 25: Clinical Applications of ASSR

Correct Answer: D. 聽神經譜系病變的確診 (Diagnosis of auditory neuropathy spectrum disorder, ANSD) – Incorrect use

Explanation:

Auditory Steady-State Response (ASSR) is used for frequency-specific auditory threshold estimation but is not reliable for diagnosing auditory neuropathy spectrum disorder (ANSD).

Common clinical applications of ASSR:

Hearing threshold estimation (especially for infants & difficult-to-test populations) → ✅ Correct (A)

Hearing aid fitting and validation → ✅ Correct (B)

Cochlear implant candidacy evaluation → ✅ Correct (C)

Not effective for diagnosing ANSD → ❌ Incorrect (D)

ANSD involves abnormal neural transmission but often has normal outer hair cell function (OAE may be present while ABR is absent).

ABR & ECochG are more suitable for ANSD diagnosis, as they assess synchronous neural firing, which is absent or abnormal in ANSD.

Evaluating answer choices:

A (Hearing threshold estimation) → ✅ Correct use

B (Hearing aid effectiveness evaluation) → ✅ Correct use

C (Cochlear implant pre-surgical assessment) → ✅ Correct use

D (ANSD diagnosis) → ❌ Incorrect use

Thus, option D is the incorrect application of ASSR.

Question 26: Signal Averaging in Auditory Evoked Responses

Correct Answer: B. 信號會隨 n 的增加而增強 n 倍 (The signal increases n times with averaging) – Incorrect statement

Explanation:

Signal averaging is a technique used in Auditory Evoked Potentials (AEPs), including ABR, to enhance the signal-to-noise ratio (SNR).

Averaging improves the clarity of the response by emphasizing the consistent signal while reducing random noise.

The signal (ABR waves) remains constant and does not get canceled because it is phase-locked to the stimulus → ✅ A is correct

Noise, being random, cancels out over time (due to positive and negative fluctuations averaging to zero) → ✅ C is correct

The reduction in noise follows the square root of n (i.e., noise decreases at a rate of $1/\sqrt{n}$) → ✅ D is correct

The signal does NOT increase linearly with n (it remains constant while noise decreases) → ❌ B is incorrect

Evaluating answer choices:

A (Signal remains constant and does not cancel out) → ✅ Correct

B (Signal increases n times with averaging) → ❌ Incorrect (signal remains constant, noise decreases)

C (Noise cancels out due to its randomness) → ✅ Correct

D (Noise decreases at a rate of $1/\sqrt{n}$) → ✅ Correct

Thus, option B is the incorrect statement.

Question 27: Causes of ABR Latency Prolongation

Correct Answer: D. 刺激音播放速率下降 (Decreasing stimulus rate) – Not a cause of latency prolongation

Explanation:

ABR wave latencies can be affected by various physiological and technical factors:

Conductive hearing loss → ✅ A is correct

A middle ear issue (e.g., otitis media) delays sound transmission to the cochlea, leading to prolonged ABR latencies.

Immature central nervous system in newborns → ✅ B is correct

In infants, myelination of the auditory pathway is incomplete, causing delayed neural transmission and prolonged latencies.

Low stimulus intensity → ✅ C is correct

Near-threshold stimuli result in longer neural response times, causing increased wave latencies, particularly wave V.

Lower stimulus rate does NOT prolong latency → ❌ D is incorrect

Higher stimulus rates cause neural fatigue, leading to prolonged latencies.

Lower stimulus rates allow the auditory system to recover, making responses more stable but NOT delayed.

Evaluating answer choices:

A (Conductive hearing loss delays sound transmission) → ✅ Correct cause

B (Immature CNS in newborns causes delayed transmission) → ✅ Correct cause

C (Low intensity stimuli cause longer latency) → ✅ Correct cause

D (Lower stimulus rate prolongs latency) → ❌ Incorrect (low rates do NOT increase latency)

Thus, option D is NOT a cause of ABR latency prolongation.

Question 28: Reducing Noise in ABR Recordings

Correct Answer: C. 僅①②③ (Only differential amplification, bandpass filtering, and averaging are correct)

Explanation:

ABR signals are very small (microvolt level) and can be easily contaminated by muscle activity (EMG), electrical interference, and background noise. Several methods can help reduce noise and improve the signal-to-noise ratio (SNR):

Differential amplification (①) → ✅ Correct

This technique subtracts common noise from two electrodes to enhance the true ABR signal while reducing external interference.

Bandpass filtering (②) → ✅ Correct

ABR signals occur in the 100–3000 Hz range, so using a bandpass filter helps eliminate unwanted low- and high-frequency noise.

Averaging (③) → ✅ Correct

Since ABR signals are time-locked while noise is random, averaging multiple trials enhances the consistent response and cancels out random noise (√n noise reduction rule).

Sequential high-pass masking (④) → ❌ Incorrect

No such standard technique in ABR testing. High-pass filtering is used, but "sequential high-pass masking" is not a commonly used method in ABR recording.

Evaluating answer choices:

① Differential amplification → ✅ Correct (reduces common noise and enhances ABR signals)

② Bandpass filtering → ✅ Correct (removes unwanted frequencies)

③ Averaging → ✅ Correct (improves SNR by reducing random noise)

④ Sequential high-pass masking → ❌ Incorrect (not a standard ABR noise-reduction method)

Thus, the correct answer is C (①②③ only).

Question 29: Recording Auditory Evoked Potentials (AEPs)

Correct Answer: B. 反應鎖時（time-locked）但不鎖相（phase-locked） (Responses are time-locked but not phase-locked) – Incorrect statement

Explanation:

When recording Auditory Evoked Potentials (AEPs), including ABR, the neural responses must be both time-locked and phase-locked to the stimulus:

Time-locked (reaction occurs at the same time after stimulus) → Required for ABR

Phase-locked (response occurs at a consistent phase of the stimulus cycle) → ABR is phase-locked to the onset of the stimulus

Since ABR is both time-locked and phase-locked, saying that it is "time-locked but not phase-locked" is incorrect.

Evaluating answer choices:

A (Two-channel recording shares the same ground electrode) → ✅ Correct

In multi-channel AEP recordings, both channels can share a single ground electrode to prevent electrical interference.

B (Time-locked but NOT phase-locked) → ❌ Incorrect (ABR is both time-locked and phase-locked)

C (Stimulus onset asynchrony = 1/stimulus rate) → ✅ Correct

Stimulus onset asynchrony (SOA) is the interval between consecutive stimuli and is the inverse of the stimulus rate.

D (A2 represents the right earlobe in the 10-20 system) → ✅ Correct

A1 = left earlobe, A2 = right earlobe (International 10-20 system).

Thus, the correct answer is B (Time-locked but not phase-locked – incorrect statement).

Question 30: Female ABR Characteristics Compared to Males

Correct Answer: B. Shorter interpeak latencies, larger wave amplitudes

Explanation:

Auditory Brainstem Response (ABR) is influenced by physiological differences between males and females. Studies have shown that:

Females tend to have shorter interpeak latencies due to smaller head sizes and shorter auditory pathways, leading to faster neural conduction.

Females also exhibit larger wave amplitudes due to hormonal influences and cochlear function differences, which enhance neural synchrony.

Thus, option B is correct because it accurately describes these observed sex-related differences in ABR.

Question 31: Key Condition for ABR Detection of Vestibular Schwannoma (Acoustic Neuroma)

Correct Answer: C. The tumor has affected low-frequency auditory nerve fibers (Least appropriate condition for ABR detection)

Explanation:

ABR is most effective in detecting vestibular schwannomas when they affect high-frequency auditory nerve fibers, which are located on the outer portion of the auditory nerve and are more susceptible to compression. Key conditions for ABR detection include:

A. Substantial compression of the auditory nerve, blocking neural transmission

B. Involvement of a sufficient number of auditory nerve fibers

D. Neural desynchronization, which disrupts the ABR waveform

However, option C (the tumor affecting low-frequency fibers) is the least appropriate condition for ABR detection because low-frequency fibers are located deeper within the nerve bundle and are less affected in the early stages of tumor growth. ABR is less sensitive to lesions primarily affecting low-frequency fibers.

Thus, option C is the correct answer for the least appropriate condition.

Question 32: Auditory Steady-State Response (ASSR) and Modulated Stimuli

Correct Answer: C. Compared to 40 Hz modulation frequency, infants exhibit larger responses to 80 Hz modulated stimuli

Explanation:

Auditory Steady-State Response (ASSR) is an electrophysiological response to amplitude- or frequency-modulated stimuli. Different modulation frequencies elicit responses from different auditory structures:

Low modulation frequencies (e.g., 40 Hz) primarily originate from higher auditory centers, such as the auditory cortex, and are more affected by subject alertness.

High modulation frequencies (e.g., 80 Hz and above) are mainly generated from brainstem structures and are more robust in infants, as their cortical responses are still developing.

Thus, option C is correct because studies have shown that infants and young children generate stronger ASSR responses to higher modulation frequencies (e.g., 80 Hz) compared to lower frequencies (e.g., 40 Hz).

Analysis of incorrect options:

A is incorrect: Higher modulation frequencies (e.g., 80 Hz) are actually less affected by patient alertness.

B is incorrect: Higher modulation frequencies elicit responses primarily from the brainstem, not higher auditory centers.

D is incorrect: The 40 Hz response matures with age, but significant adult-like cortical responses do not fully develop until later in childhood, beyond age two.

Question 33: Clinical Applications of Auditory Late Latency Responses (LLR)

Correct Answer: D. LLR cannot be recorded in individuals with Auditory Neuropathy Spectrum Disorder (ANSD) (Least appropriate statement)

Explanation:

Auditory Late Latency Responses (LLR) are evoked potentials that assess cortical auditory processing and neural activity. They have multiple clinical applications, including:

A. Hearing aid fitting for infants: LLR can help estimate auditory thresholds in babies fitted with hearing aids.

B. Measuring auditory system maturation: LLR responses evolve with age and can be used to track neural development.

C. Evaluating the benefits of auditory prostheses: LLR is useful in assessing how well hearing aids or cochlear implants provide auditory stimulation.

However, option D is incorrect because LLR can still be recorded in individuals with Auditory Neuropathy Spectrum Disorder (ANSD).

ANSD is characterized by impaired neural synchrony but preserved outer hair cell function.

While early responses like ABR may be absent or abnormal in ANSD, later cortical responses (such as LLR) may still be measurable.

Therefore, it is incorrect to say that LLR cannot be recorded in ANSD cases.

Thus, option D is the least appropriate statement.

Question 34: Clinical Applications of Electrocochleography (ECochG)

Correct Answer: A. Cochlear microphonic (CM) can be used to assess inner hair cell function (Least appropriate statement)

Explanation:

Electrocochleography (ECochG) is a technique used to measure electrical potentials generated by the cochlea and auditory nerve. The key components of ECochG include:

Cochlear Microphonic (CM): This response reflects the function of outer hair cells (OHCs), not inner hair cells (IHCs). It represents the receptor potential that follows the stimulus waveform, primarily generated by OHCs.

Summating Potential (SP): Generated by hair cells, SP is often abnormally large in Ménière’s disease, which is why option C is correct.

Compound Action Potential (CAP): Represents the synchronized activity of auditory nerve fibers, influenced by both IHCs and auditory nerve synapses.

Option A is incorrect because CM reflects outer hair cell activity, not inner hair cell function. Inner hair cell dysfunction would be better assessed using compound action potential (CAP) or auditory brainstem response (ABR) rather than CM.

Analysis of other options:

B is correct: CAP is useful in assessing the communication between IHCs and auditory nerve fibers.

C is correct: Patients with Ménière’s disease often show an abnormally large SP amplitude due to excessive endolymphatic pressure.

D is correct: SP/AP ratio is an important marker for diagnosing Ménière’s disease.

Thus, option A is the least appropriate statement.

Question 35: Most Appropriate Statement About ECochG

Correct Answer: A. ECochG can be used intraoperatively to assess the effectiveness of cochlear implant placement

Explanation:

ECochG has several clinical applications, including monitoring cochlear function intraoperatively during cochlear implant (CI) surgery. This helps surgeons assess whether the electrode array is appropriately positioned and whether the cochlea is still functioning.

Analysis of other options:

B is incorrect: ECochG can estimate hearing sensitivity, particularly in low-frequency regions, so it can be used for threshold estimation.

C is incorrect: While an elevated SP/AP ratio is a key indicator of Ménière’s disease, a normal SP/AP ratio does not completely rule out the condition.

D is incorrect: A high SP/AP ratio is commonly associated with Ménière’s disease, but it is not exclusively diagnostic—other conditions may also influence this ratio.

Thus, option A is the most appropriate statement.

Question 36: Early Latency Response vs. Late Latency Response

Correct Answer: B. ③ Early latency responses are less affected by alertness, and ④ their response amplitudes are smaller.

Explanation:

Auditory evoked potentials (AEPs) are categorized based on their latency:

Early latency responses (e.g., Auditory Brainstem Response, ABR) occur within the first 10 ms after a stimulus and originate from the auditory nerve and brainstem.

Late latency responses (LLR) occur after 50 ms and originate from the auditory cortex, making them more dependent on higher cognitive processes.

Analysis of each option:

① Early latency responses require a lower stimulus rate? ❌ Incorrect

Early latency responses require a higher stimulus rate (e.g., 10-30 clicks per second in ABR). In contrast, late latency responses require a lower stimulus rate to avoid cortical adaptation.

② Early latency responses have lower frequency components? ❌ Incorrect

Early latency responses have high-frequency components (above 100 Hz), whereas late latency responses have lower frequency components (~1-30 Hz).

③ Early latency responses are less affected by alertness? ✅ Correct

Since they originate from the brainstem, early responses (like ABR) are minimally influenced by attention or alertness. In contrast, late responses rely on cortical processing and are greatly affected by the patient's state of wakefulness.

④ Early latency responses have smaller wave amplitudes? ✅ Correct

ABR wave amplitudes are much smaller than late latency responses (such as P1, N1, and P2). Late responses involve larger cortical neurons, leading to greater amplitudes.

⑤ Early latency responses have larger noise amplitudes? ❌ Incorrect

Background noise affects all responses, but early responses are less affected by muscle and movement artifacts compared to late responses.

Thus, option B (③ Early latency responses are less affected by alertness, and ④ their amplitudes are smaller) is the most appropriate choice.

Question 37: Diagnosis Based on Click ABR Findings

Correct Answer: B. Conductive Hearing Loss

Explanation:

The key ABR findings in this 5-year-old boy are:

Prolonged absolute latency of Wave V → Suggests a delay in sound transmission.

Normal interwave latencies (I-V, III-V, I-III) → Indicates normal neural conduction, ruling out retrocochlear pathology.

Abnormal bone conduction ABR → Suggests a hearing deficit beyond just an air-bone gap.

Conductive hearing loss (CHL) is the most likely diagnosis because:

CHL delays Wave V absolute latency due to a reduction in sound transmission through the outer/middle ear.

CHL preserves normal interwave latencies since neural conduction remains unaffected.

Bone conduction ABR is also abnormal, reflecting a true hearing loss rather than just an air conduction issue.

Analysis of other options:

A (Normal hearing) ❌ Incorrect → The delayed absolute Wave V latency and abnormal bone conduction ABR indicate hearing loss.

C (Sensorineural hearing loss) ❌ Incorrect → SNHL would cause both prolonged absolute latency AND abnormal interwave latencies.

D (Neural hearing loss) ❌ Incorrect → Auditory neuropathy would show abnormal interwave latencies, particularly affecting Wave I and V.

Thus, option B (Conductive Hearing Loss) is the most appropriate choice.

Question 38: Most Appropriate Statement About ANSD

Correct Answer: A. When stimulus polarity is reversed, the Cochlear Microphonic (CM) waveform shows phase inversion.

Explanation:

Auditory Neuropathy Spectrum Disorder (ANSD) is characterized by:

Preserved outer hair cell function (normal Otoacoustic Emissions, OAE)

Absent or abnormal Auditory Brainstem Response (ABR) due to neural dyssynchrony

Cochlear Microphonic (CM) response is present and inverts when stimulus polarity is reversed

Option A is correct because:

CM originates from outer hair cells, which remain functional in ANSD.

When the stimulus polarity is flipped (rarefaction vs. condensation clicks), CM also flips, confirming the presence of cochlear microphonics despite ABR abnormalities.

Analysis of other options:

B (Cochlear implants are not suitable for ANSD) ❌ Incorrect → Cochlear implants can improve hearing in many ANSD cases by bypassing the faulty auditory nerve synapses and directly stimulating the cochlea.

C (Both OAE and ABR are abnormal in ANSD) ❌ Incorrect → In ANSD, OAE is typically normal, while ABR is absent or severely distorted.

D (ASSR can diagnose ANSD) ❌ Incorrect → ASSR is not reliable for diagnosing ANSD because it measures steady-state responses, which may not detect neural dysynchrony. ANSD is best diagnosed with a combination of ABR and CM testing.

Thus, option A (CM phase inversion with polarity change) is the most appropriate choice.

Question 39: Optimal f2/f1 Ratio for Strongest DPOAE Response

Correct Answer: B. 1.22

Explanation:

Distortion Product Otoacoustic Emissions (DPOAE) are generated when the cochlea is stimulated by two pure tones (f1 and f2, where f2 > f1). The most prominent distortion product occurs at 2f1 - f2, and its strength depends on the ratio f2/f1.

Research has shown that the optimal f2/f1 ratio for the strongest DPOAE response is approximately 1.22. This ratio maximizes the cochlear nonlinear interaction, producing the strongest emissions detected by the probe microphone.

Analysis of other options:

A (1.11) ❌ Too low – Produces a weaker response.

C (1.33) ❌ Too high – The emission response decreases.

D (1.44) ❌ Much too high – The interaction between f1 and f2 weakens significantly.

Thus, option B (1.22) is the correct answer.

Question 40: aABR Pass but OAE Fail in a Newborn

Correct Answer: D. Mild Conductive Hearing Loss

Explanation:

In newborn hearing screening, two common tests are:

Automated Auditory Brainstem Response (aABR): Assesses the auditory pathway up to the brainstem and is sensitive to neural hearing loss.

Otoacoustic Emissions (OAE): Measures cochlear outer hair cell function and is affected by conductive and sensory hearing loss.

If a newborn passes aABR but fails OAE, the most likely reason is a mild conductive issue, such as vernix (ear canal debris), middle ear fluid, or mild otitis media. These conditions block the OAE response but do not significantly affect the neural transmission assessed by aABR.

Analysis of other options:

A (Moderate sensory hearing loss) ❌ Incorrect → A moderate sensory (cochlear) loss would cause both OAE and aABR to fail.

B (Mild neural hearing loss) ❌ Incorrect → Neural hearing loss typically affects aABR first, so a pass in aABR would not be expected.

C (Moderate neural hearing loss) ❌ Incorrect → This would lead to an aABR failure, not a pass.

Thus, option D (Mild Conductive Hearing Loss) is the most appropriate answer.

Question 41: Recommended Tools for Newborn Hearing Screening

Correct Answer: D. Auditory Steady-State Response (ASSR) (Not a recommended tool for newborn screening)

Explanation:

Newborn hearing screening aims to quickly and efficiently identify hearing loss, typically using:

Automated Auditory Brainstem Response (aABR) – Tests the integrity of the auditory nerve and brainstem pathways.

Transient Evoked Otoacoustic Emissions (TEOAE) – Assesses outer hair cell function, commonly used in universal newborn hearing screening (UNHS).

Distortion Product Otoacoustic Emissions (DPOAE) – Similar to TEOAE but can provide frequency-specific cochlear assessment.

Why is ASSR not recommended for newborn screening?

ASSR is time-consuming compared to aABR and OAE tests.

It requires the baby to be in a deeper sleep or sedation, which is not practical for routine screening.

ASSR is mainly used for threshold estimation in infants who fail the initial screening, rather than as a first-line screening tool.

Thus, option D (ASSR) is not a recommended newborn hearing screening tool.

Question 42: Tympanometry Interpretation for Pediatric Middle Ear Screening

Correct Answer: B. Compensated Static Admittance (YTM) and/or Tympanometric Width (TW)

Explanation:

Tympanometry is commonly used in children to assess middle ear function and detect issues like otitis media with effusion (OME). The best indicators for pass/fail criteria in pediatric middle ear screening are:

Compensated Static Admittance (YTM) – Measures middle ear mobility, with low values suggesting middle ear dysfunction.

Tympanometric Width (TW) – Measures the sharpness of the tympanogram peak; a broad TW may indicate middle ear pathology (e.g., fluid behind the eardrum).

Why are the other options incorrect?

A (TPP and/or tympanogram classification) ❌

Tympanometric Peak Pressure (TPP) alone is not a reliable pass/fail indicator because it can fluctuate due to normal Eustachian tube function.

C (Equivalent Ear Canal Volume (Vec) and/or tympanogram classification) ❌

Vec is used to detect perforations or impacted wax, but it does not directly indicate middle ear function.

D (YTM and/or tympanogram classification) ❌

Tympanogram classification (Type A, B, C) is useful but not sufficient alone; TW is a better criterion for screening purposes.

Thus, option B (YTM and/or TW) is the best choice for pediatric tympanometry screening.

Question 43: Most Inappropriate Statement About Newborn Hearing Screening & Intervention

Correct Answer: D. "For bilaterally deaf infants, cochlear implantation should be done before 8 months of age." (Incorrect statement)

Explanation:

Newborn hearing screening and early intervention follow the "1-3-6" guideline recommended by organizations like the Joint Committee on Infant Hearing (JCIH):

1 month → All newborns should have their hearing screened. (Correct, supports option A)

3 months → Infants who fail the screening should have a diagnostic ABR by 3 months to confirm hearing loss. (Correct, supports option B)

6 months → If hearing loss is confirmed, early intervention (e.g., hearing aids, speech therapy) should begin by 6 months. (Correct, supports option C)

Why is option D incorrect?

Cochlear implantation is typically recommended around 12 months of age, not before 8 months.

Before implantation, hearing aids and auditory training are usually attempted first.

Early implantation (as young as 9–12 months) can improve speech and language outcomes, but implantation before 8 months is not a standard recommendation.

Thus, option D is the most inappropriate statement.

Question 44: Recommended Hearing Monitoring for At-Risk Infants

Correct Answer: D. 6 years

Explanation:

Some newborns pass their initial hearing screening but have risk factors for delayed-onset or progressive hearing loss, such as:

Neonatal intensive care unit (NICU) stay (especially >5 days)

Cytomegalovirus (CMV) infection

Family history of childhood hearing loss

Certain syndromes (e.g., Usher, Pendred, Waardenburg, Alport, Jervell & Lange-Nielsen)

Ototoxic medication exposure

For these infants, hearing should be monitored periodically until at least age 6 because:

Late-onset hearing loss can develop in early childhood.

School-age hearing is critical for speech, language, and academic performance.

Analysis of other options:

A (3 years) ❌ Too short – Some progressive losses may not be evident by this age.

B (4 years) ❌ Still too short – Monitoring should extend into school age.

C (5 years) ❌ Better but still not ideal – Extending to 6 years ensures late-onset cases are caught.

Thus, option D (6 years) is the correct answer.

Question 45: Most Appropriate Hearing Assessment for a 5-Month-Old Infant

Correct Answer: D. "At this stage, electrophysiological testing should be the primary assessment method, supplemented by behavioral testing for further management and recommendations."

Explanation:

At 5 months of age, infants are still too young for certain behavioral hearing tests that require active participation. The most appropriate hearing evaluation methods include:

Electrophysiological testing (ABR, ASSR, OAE) → Provides objective auditory threshold estimates.

Behavioral responses (e.g., Minimum Response Level, MRL) → Can supplement results but may be variable at this age.

Since infants at 5 months lack the cognitive and motor skills required for conditioned responses, electrophysiological testing is the gold standard for obtaining reliable hearing thresholds.

Analysis of Other Options:

A (Use of 226 Hz for middle ear function testing) ✖ Incorrect

The appropriate probe tone for tympanometry in infants under 6 months is 1000 Hz, not 226 Hz.

226 Hz is used for older infants and adults because it better detects stiffened middle ear systems, but it underestimates middle ear dysfunction in younger infants due to their more compliant ear canal structures.

B (Using play conditioning for hearing threshold testing) ✖ Incorrect

Conditioned Play Audiometry (CPA) is used for toddlers (2–5 years old), not infants.

A 5-month-old is best tested using Visual Reinforcement Audiometry (VRA), which starts around 6 months when head-turning behavior becomes more reliable.

C (MRL and electrophysiology mismatch of 20 dB means unreliable behavioral testing) ✖ Incorrect

Differences between ABR and MRL can occur due to factors such as test environment and infant alertness.

A 20 dB difference is not necessarily a sign of unreliable results—it can reflect normal testing variations in infants.

Conclusion:

For a 5-month-old infant, electrophysiological tests (ABR, OAE, ASSR) should be the primary diagnostic tool, and behavioral observations can provide additional insights.

Thus, option D is the correct answer.

Question 46: The audiogram shows low-frequency hearing preservation with severe high-frequency loss, which is typical of sudden sensorineural hearing loss (SSHL) with residual low-frequency hearing. Now, let's analyze the given options based on electrophysiological testing:

Option A: If the patient has loudness recruitment, the absolute latencies of waves I, III, and V in click ABR may be normal

Correct. Loudness recruitment suggests that while cochlear outer hair cells may be affected, the auditory nerve conduction remains intact. As a result, click ABR latencies for waves I, III, and V can still fall within the normal range.

Option B: If waves I, III, and V are absent in click ABR, it is impossible to determine the lesion location

Correct. If click ABR shows no identifiable waveforms, the cause could be cochlear, auditory nerve, or central pathway dysfunction, making it difficult to pinpoint the exact site of the lesion.

Option C: There will be no wave V response in 500 Hz tone burst ABR

Correct. ABR is primarily designed to assess high-frequency neural transmission. Since low-frequency stimuli (e.g., 500 Hz tone burst) generate weak neural responses, wave V may not be detectable, especially in patients with severe hearing loss in the tested frequency range.

Option D: The acoustic reflexes are absent at all frequencies in both ears

Incorrect (Most inappropriate choice). The acoustic reflex (AR) depends on the integrity of the cochlea, auditory nerve, brainstem pathways, and facial nerve.

Since this patient retains low-frequency hearing, AR may still be present at low frequencies.

If AR is truly absent at all frequencies, this suggests auditory neuropathy spectrum disorder (ANSD) or central involvement, rather than just a cochlear hearing loss from SSHL.

Final Answer: The most inappropriate statement is Option D.

Question 47: Diagnosis of the Baseball Player's Left Ear Condition

Case Summary:

History: Head trauma to the left side

Symptoms: Persistent left ear hearing loss for 6 weeks

Findings: Moderate conductive hearing loss in the left ear, Normal tympanic membrane

Tympanometry: Left ear Ad type, Right ear A type

Analysis of the Answer Choices:

A. Auricular Hematoma (耳部血腫) → Incorrect

Auricular hematoma is a soft tissue injury affecting the external ear, commonly seen in wrestlers or rugby players.

It does not cause conductive hearing loss or an Ad-type tympanogram.

B. Serous Otitis Media (漿液性中耳炎) → Incorrect

Serous otitis media typically presents with a B-type tympanogram (flat curve) due to middle ear effusion.

The Ad-type tympanogram suggests hypermobility, not fluid accumulation.

C. Temporal Bone Fracture (顳骨骨折) → Unlikely

Temporal bone fractures may lead to sensorineural, conductive, or mixed hearing loss.

However, the absence of facial nerve symptoms and the specific finding of an Ad-type tympanogram suggests a more localized issue affecting the ossicular chain rather than a full fracture.

D. Ossicular Discontinuity (聽小骨斷裂) → Correct Answer

Ad-type tympanogram suggests ossicular hypermobility, which occurs when the ossicular chain is disrupted.

Head trauma is a common cause of ossicular chain discontinuity, especially at the incudostapedial joint.

Moderate conductive hearing loss aligns with this diagnosis.

✅ Final Answer: D. Ossicular Discontinuity (聽小骨斷裂).

Question 48: Electrophysiological and Audiological Tests

Analysis of the Answer Choices:

A. Electrocochleography (ECochG) cannot be used to measure hearing thresholds → Incorrect

ECochG can estimate auditory thresholds, particularly in low frequencies, though it is not commonly used for precise audiometric threshold assessment.

B. Auditory Brainstem Response (ABR) cannot measure hearing thresholds → Incorrect

ABR is frequently used to estimate hearing thresholds, especially for infants and difficult-to-test patients.

ABR thresholds correlate well with audiometric thresholds, particularly in the high frequencies (1kHz–4kHz).

C. Wave I of ABR is the same as the Action Potential (AP) in ECochG → Correct

Wave I of ABR originates from the distal auditory nerve and corresponds to the Action Potential (AP) in ECochG.

Both reflect synchronous neural firing from the cochlear nerve.

D. Auditory Steady-State Response (ASSR) cannot be used to measure hearing thresholds → Incorrect

ASSR is specifically designed for objective hearing threshold estimation, particularly in profound hearing loss cases.

It can provide frequency-specific audiometric threshold estimation in both infants and adults.

✅ Final Answer: C. Wave I of ABR is the same as the Action Potential (AP) in ECochG.

Question 49: Identifying the Incorrect Statement About Malingering (Functional Hearing Loss)

Analysis of Answer Choices:

A. Late latency responses (LLR) can help confirm the possibility of malingering → Correct

Late latency responses (LLR), such as P300 and mismatch negativity (MMN), are cortical auditory evoked potentials that require conscious auditory perception.

If a patient is truly malingering, these responses may still be present, revealing a discrepancy between objective electrophysiological findings and subjective behavioral thresholds.

B. A difference greater than 15 dB between pure-tone average (PTA) and speech recognition threshold (SRT) suggests possible malingering → Correct

Normally, PTA and SRT should closely match (within 5–10 dB).

If SRT is significantly better (≥15 dB difference) than PTA, it suggests inconsistent responses, raising suspicion of malingering.

C. The Stenger test can be used to detect unilateral malingering → Correct

The Stenger test is highly effective in detecting unilateral malingering by presenting a tone of equal frequency but differing intensities in both ears.

If the patient truly has unilateral hearing loss, they will only perceive the louder tone in the normal ear.

A malingering patient, however, may pretend not to hear the tone at all, revealing the deception.

D. Late latency responses (LLR) provide subcortical (sub-cortical) auditory function information → Incorrect (Most Incorrect Statement)

LLRs, such as P300 and MMN, originate from the auditory cortex, not the subcortical structures.

Subcortical auditory function is better assessed using Auditory Brainstem Response (ABR).

✅ Final Answer: D. Late latency responses provide subcortical auditory function information (Incorrect statement).

Question 50: Diagnosis of a Patient with Tinnitus, Dizziness with Loud Sounds, and Low-Frequency Conductive Hearing Loss

Key Clinical Findings:

Symptoms: Left-sided tinnitus and dizziness triggered by loud sounds (Tullio phenomenon)

Audiometry: Low-frequency conductive hearing loss in the left ear

Acoustic Reflex: Normal in the left ear

Vestibular Evoked Myogenic Potential (VEMP): Left P13-N23 amplitude is large, Threshold is lower than normal

Analysis of the Answer Choices:

A. Ménière's Disease → Incorrect

Ménière's disease typically presents with episodic vertigo, fluctuating low-frequency sensorineural hearing loss (not conductive loss), tinnitus, and aural fullness.

VEMP responses may be normal or reduced, but not increased with a lower threshold.

B. Otosclerosis → Incorrect

Otosclerosis is a common cause of low-frequency conductive hearing loss, but:

It is not associated with sound-induced dizziness (Tullio phenomenon).

Stapedial reflexes are usually absent or abnormal, not normal as seen in this patient.

VEMP thresholds are typically normal or elevated, not reduced.

C. Cholesteatoma → Incorrect

Cholesteatoma can cause conductive hearing loss, but it does not typically cause: Sound-induced dizziness (Tullio phenomenon), Increased VEMP amplitude and reduced threshold.

Additionally, cholesteatomas usually affect the middle ear space and would likely impact tympanometry and acoustic reflexes, which are normal here.

D. Superior Canal Dehiscence Syndrome (SCDS) → Correct

SCDS occurs due to a bony defect in the superior semicircular canal, leading to:

Tullio phenomenon (dizziness induced by loud sounds)

Low-frequency conductive hearing loss (third-window effect)

Enhanced VEMP responses (large amplitude, lower threshold) due to increased vestibular sensitivity

Normal acoustic reflexes support this diagnosis since middle ear function remains intact.

✅ Final Answer: D. Superior Canal Dehiscence Syndrome (SCDS).

4106

Question 1: Janssen Earmold

Understanding Janssen Earmold:

Janssen earmold is designed to maximize the openness of the ear canal to maintain natural resonance and reduce occlusion effects.

It is often used in CROS (Contralateral Routing of Signal) hearing aids to allow sound to enter naturally while still transmitting it to the better ear.

Analysis of the Answer Choices:

A. Also called a CROS earmold → Correct

Janssen earmolds are commonly used for CROS and BiCROS hearing aids.

B. Designed to keep the ear canal as open as possible and maintain natural resonance → Correct

The open design reduces occlusion effects and preserves natural acoustics.

C. Acoustic and frequency response are similar to open-fit earmolds → Correct

Since Janssen earmolds are highly open, their acoustic properties are similar to open-fit hearing aids, providing minimal occlusion.

D. Designed to improve wearing stability → Incorrect (Most Inappropriate Choice)

The primary purpose of Janssen earmolds is not to enhance stability but to keep the ear canal open.

In fact, their open design may reduce retention compared to traditional earmolds.

✅ Final Answer: D. Designed to improve wearing stability (Incorrect Statement).

Question 2: CIC and High-Gain Hearing Aid Ear Impressions

Understanding Deep Canal (CIC) and High-Gain Hearing Aid Ear Impression Techniques:

Deep ear impressions need to extend past the second bend of the ear canal to ensure a secure fit and prevent sound leakage.

Special techniques, such as open-jaw impressions, help create a stable fit, particularly for powerful hearing aids.

Analysis of the Answer Choices:

A. Widening at the second bend is important → Correct

Ensuring proper width at the second bend helps improve comfort and retention.

B. Impressions should be taken with an open mouth → Correct

Open-mouth impressions help prevent movement-related feedback and looseness, especially for high-gain hearing aids.

C. The impression should extend at least 5 mm past the second bend → Correct

A deep fit ensures a secure seal, particularly for CIC and high-gain hearing aids.

D. Using an extra-hard material after the second bend increases tightness → Incorrect (Wrong Statement)

Very hard materials past the second bend can cause discomfort and irritation.

Instead, a softer, more flexible material is preferred for deep impressions to accommodate ear canal movement.

✅ Final Answer: D. Using an extra-hard material after the second bend increases tightness (Incorrect Statement).

Question 3: Frequency Lowering Techniques

Understanding Frequency Lowering Strategies:

Frequency-lowering techniques are used in hearing aids to help individuals with high-frequency hearing loss by shifting inaudible high-frequency sounds into a lower, more audible range.

There are two primary methods:

Non-linear Frequency Compression (NLFC):

Compresses high frequencies into a smaller frequency range.

Each input frequency has a unique corresponding output frequency (one-to-one mapping).

Frequency Transposition (Frequency Shifting):

Moves high frequencies downward by overlapping them with lower frequencies.

Can enhance the perception of sounds like semivowels and nasals.

Analysis of Answer Choices:

A. Non-linear frequency compression ensures each input frequency has only one corresponding output frequency → Correct

NLFC maintains a one-to-one mapping, meaning each high-frequency input is compressed but does not overlap with another frequency.

B. Frequency transposition can enhance low-frequency speech cues like semivowels and nasals → Correct (Most Appropriate Choice)

Frequency transposition moves high-frequency components into lower-frequency regions, where they overlap with natural low-frequency speech sounds.

This reinforcement effect can improve the perception of semivowels (e.g., /w/, /j/) and nasals (/m/, /n/), which have strong low-frequency components.

C. The primary goal of frequency lowering is to lower the start frequency and increase the compression ratio → Partially Correct but Not the Best

While adjusting the start frequency and compression ratio is a strategy, the main objective is to improve speech intelligibility, not just detect high-frequency sounds.

D. The effectiveness of frequency lowering is fully proven and universally applicable for severe high-frequency hearing loss → Incorrect

While some studies show benefits, frequency-lowering effectiveness varies by individual and may not be beneficial for all patients with high-frequency hearing loss.

✅ Final Answer: B. Frequency transposition enhances low-frequency speech cues like semivowels and nasals (Most Appropriate Statement).

Question 4: Linear Frequency Compression Calculation

Understanding Linear Frequency Compression:

Linear frequency compression reduces the entire frequency range proportionally.

The formula for linear frequency compression follows:

foutput=finput×compression ratiof_{\text{output}} = f_{\text{input}} \times \text{compression ratio}foutput =finput ×compression ratio

Given:

5000 Hz is compressed to 3000 Hz, meaning the compression ratio is:

Compression Ratio=30005000=0.6\text{Compression Ratio} = \frac{3000}{5000} = 0.6Compression Ratio=50003000 =0.6

To find the new output frequency for 3000 Hz input:

foutput=3000×0.6=1800 Hzf_{\text{output}} = 3000 \times 0.6 = 1800 \text{ Hz}foutput =3000×0.6=1800 Hz

Analysis of Answer Choices:

A. 1000 Hz → Incorrect (Not derived from the given compression ratio).
B. 1800 Hz → Correct (Calculated based on the 0.6 compression ratio).
C. 2000 Hz → Incorrect (Does not match the computed value).
D. Insufficient information to determine → Incorrect (The compression ratio can be derived).

✅ Final Answer: B. 1800 Hz.

Question 5: Signal Processing in Hearing Aids and Improving SNR

Understanding Signal-to-Noise Ratio (SNR) Improvement:

SNR improvement means enhancing speech clarity in noisy environments.

Many hearing aid features process sound, but only directional microphones actively improve SNR by focusing on speech from the front and reducing background noise from other directions.

Analysis of Answer Choices:

A. Using vents, dampers, or sound bores → Incorrect

These modify acoustic resonance and prevent occlusion, but they do not improve SNR in noisy environments.

B. Multi-channel compression → Incorrect

Compression adjusts loudness levels across different frequency channels, but it does not enhance SNR.

C. Filter system adjusting frequency response → Incorrect

Adjusting the frequency response can amplify speech frequencies, but it does not directly enhance SNR.

D. Using directional microphones → Correct

Directional microphones focus on sounds from the front while reducing background noise from other directions, leading to a measurable improvement in SNR.

✅ Final Answer: D. Using directional microphones.

Question 6: Wireless Transmission and Hearing Aid T-Coils

Understanding Wireless Transmission in Hearing Aids:

T-coil (telecoil) technology is used in hearing aids to receive electromagnetic signals.

It is specifically designed to work with inductive loop systems (magnetic loops), which generate an electromagnetic field that T-coils pick up.

Analysis of Answer Choices:

A. Magnetic loops (induction loops) → Correct

Only magnetic loops work with T-coils because they transmit signals via electromagnetic fields.

B. Radio-frequency (FM) transmission → Incorrect

FM systems require dedicated receivers in hearing aids, not T-coils.

C. Infrared transmission → Incorrect

Infrared systems transmit sound via light waves, which cannot be picked up by T-coils.

D. Sound-field amplification systems → Incorrect

These amplify sound through speakers, benefiting all listeners but not using T-coil technology.

✅ Final Answer: A. Magnetic loops.

Question 7: Filter Structures in Hearing Aids

Understanding Filter Structures in Hearing Aids

Hearing aids use filter structures to shape and control frequency responses. Different architectures affect how signals are processed.

Serial filter structures: Process signals in a stepwise manner, where one filter's output feeds into the next.

Parallel filter structures: Process signals simultaneously across multiple frequency bands.

Serial-parallel filter structures: Combine both approaches to optimize frequency response and smooth gain adjustments.

Analysis of Answer Choices:

A. Avoiding an uneven gain-frequency response → Correct

Serial-parallel filter structures effectively smooth out gain-frequency curves, preventing unwanted dips and peaks in amplification.

This results in a more stable and predictable frequency response, which improves sound quality and speech intelligibility.

B. Limited frequency response selection → Incorrect

Filter structures shape frequency response, but they do not dictate the number of frequency response options available in the hearing aid.

C. Insufficient signal processing channels → Incorrect

The number of processing channels is determined by the digital signal processing (DSP) hardware, not by filter structure design.

D. Preventing peak clipping due to excessive volume → Incorrect

Peak clipping occurs when the signal exceeds the amplifier's dynamic range, leading to distortion.

This issue is typically managed by compression algorithms, not filter structures.

✅ Final Answer: A. Avoiding an uneven gain-frequency response curve.

Question 8: Bone-Anchored Hearing Aids (BAHA) vs. Traditional Air-Conduction Hearing Aids

Understanding BAHA vs. Air-Conduction Hearing Aids

Air-conduction hearing aids amplify sound and deliver it through the ear canal to the eardrum and middle ear.

Bone-anchored hearing aids (BAHA) bypass the outer and middle ear by transmitting vibrations directly to the cochlea through the skull bone, making them useful for conductive or mixed hearing loss, especially when the ear canal cannot be used.

Analysis of Answer Choices:

A. The larger the air-bone gap, the more effective air-conduction hearing aids are → Incorrect

A larger air-bone gap means more conductive hearing loss.

Air-conduction hearing aids become less effective as the air-bone gap increases because they rely on transmitting sound through the damaged middle ear.

B. The larger the air-bone gap, the greater the sensorineural hearing level an air-conduction hearing aid can provide → Incorrect

Air-conduction aids compensate for conductive loss, not sensorineural loss.

A large air-bone gap suggests conductive hearing loss, not greater sensorineural hearing ability.

C. Bone-anchored hearing aids are preferred for conductive hearing loss with chronic ear infections → Correct

Chronic ear infections can make wearing air-conduction hearing aids problematic due to moisture, irritation, and infections.

BAHA bypasses the infected middle ear, providing a more hygienic and effective solution.

D. The higher the bone-conduction threshold, the higher the satisfaction with BAHA → Incorrect

A higher bone-conduction threshold means worse cochlear function, leading to poorer BAHA outcomes.

BAHA works best when bone-conduction thresholds are within the normal or mild hearing loss range.

✅ Final Answer: C. Bone-anchored hearing aids are preferred for conductive hearing loss with chronic ear infections.

Question 9: Real-Ear Measurement (REM) and Gain Calculations

Understanding Real-Ear Measurement (REM)

Real-ear measurement (REM) assesses how a hearing aid amplifies sound inside the ear canal. It includes:

Real-ear aided gain (REAG): The gain of a hearing aid while worn.

Real-ear unaided gain (REUG): The natural resonance of the ear without amplification.

Real-ear insertion gain (REIG): The gain provided by the hearing aid, calculated as REIG = REAG - REUG.

Analysis of Answer Choices:

A. REAG - REIG = REUG → Incorrect

The correct formula is REIG = REAG - REUG.

This choice inverts the formula, making it incorrect.

B. Real-ear gain is affected by microphone location effects (MLE), including diffraction and resonance from the body, head, pinna, concha, and ear canal → Correct

MLE influences how sound is received by the hearing aid microphone, affecting gain measurement.

Head, pinna, and ear canal resonance impact high-frequency amplification.

C. Behind-the-ear (BTE) hearing aids create more microphone location effects than completely-in-canal (CIC) hearing aids at 2–4 kHz, especially from a 45-degree sound source → Correct

BTE microphones sit outside the ear canal, leading to more MLE in the 2–4 kHz range.

CIC aids sit deeper inside the ear, reducing MLE.

D. The typical REUR curve for adults should have a peak between 2.2–3.2 kHz → Correct

The external ear naturally amplifies sounds around 2.5–3 kHz, enhancing speech clarity.

This is a normal finding in REM measurements.

✅ Final Answer: A. REAG - REIG = REUG is incorrect. The correct formula is REIG = REAG - REUG.

Question 10: Enhancing High-Frequency Gain in Hearing Aids

Understanding High-Frequency Gain Adjustments

When hearing loss is concentrated in the high-frequency range, the hearing aid must effectively amplify high-frequency sounds while minimizing distortion and feedback. Several acoustic modifications can influence high-frequency gain.

Analysis of Answer Choices:

A. Gradually increasing the diameter of the sound bore → Correct

A larger sound bore diameter enhances high-frequency amplification by reducing resistance to high-frequency sounds.

Wide-diameter sound bores allow better transmission of high-frequency sounds, improving clarity.

B. Adding a vent → Incorrect

Venting mainly affects low-frequency gain, not high-frequency gain.

Larger vents reduce low-frequency amplification but do not significantly boost high frequencies.

C. Changing the tubing’s internal diameter → Partially correct but less effective than A

Wider tubing (e.g., Libby Horn tubing) can increase mid-to-high-frequency gain, but its effect is less significant than modifying the sound bore.

D. Extending the earmold length → Incorrect

A longer earmold length mainly affects low and mid frequencies by modifying resonance, but it does not significantly enhance high-frequency gain.

✅ Final Answer: A. Gradually increasing the diameter of the sound bore.

Question 11: Expansion Technology and Ideal Compression Ratio in Digital Hearing Aids

Understanding Expansion in Digital Hearing Aids

Expansion is used to reduce amplification for very soft sounds (e.g., background noise like air conditioning hum) while keeping speech sounds audible.

It is the opposite of compression: compression amplifies soft sounds, whereas expansion reduces low-level noise.

The compression ratio (CR) determines how much amplification is reduced for soft sounds.

Analysis of Answer Choices:

A. 1:1 (No Expansion Applied) → Incorrect

A 1:1 ratio means no change in amplification, so soft sounds would still be amplified at normal levels.

B. 1:2 (Incorrect Interpretation of CR) → Incorrect

Compression ratios are expressed with the input change first (e.g., 2:1, meaning an input change of 2 dB results in an output change of 1 dB).

A 1:2 ratio does not exist in standard hearing aid terminology.

C. 2:1 (Moderate Expansion) → Correct

A moderate compression ratio (e.g., 2:1) effectively reduces low-level environmental noise while maintaining speech clarity.

It balances noise reduction without overly distorting soft speech sounds.

D. 10:1 (Extreme Expansion) → Incorrect

A 10:1 ratio would overly suppress soft sounds, making even important speech cues inaudible.

✅ Final Answer: C. 2:1, as it effectively reduces low-level noise while preserving speech intelligibility.

Question 12: Effect of Directional Microphones and Digital Noise Reduction on Cognitive Effort

Understanding Cognitive Effort in Noise

Cognitive effort refers to the mental resources required to understand speech, especially in noisy environments.

Directional microphones (DMs) enhance speech understanding by focusing on sounds from the front while reducing background noise.

Digital noise reduction (DNR) reduces steady-state background noise but does not enhance speech clarity.

Findings from Desjardins (2016):

Both directional microphones and digital noise reduction significantly reduced cognitive effort when listening in noise.

This was measured through pupillometry (pupil dilation as an indicator of effort) and subjective listening effort scales.

Analysis of Answer Choices:

A. Only directional microphones significantly reduce cognitive effort → Incorrect

Directional microphones help improve speech clarity, but DNR also plays a role in reducing background noise distractions.

B. Only digital noise reduction significantly reduces cognitive effort → Incorrect

While DNR helps with listening comfort, it does not improve speech clarity like directional microphones do.

C. Both directional microphones and digital noise reduction significantly reduce cognitive effort → Correct

Desjardins (2016) found that the combination of DNR and DM significantly reduced cognitive effort, making speech easier to process in noise.

D. Neither directional microphones nor digital noise reduction significantly reduce cognitive effort → Incorrect

Research shows both features contribute to reducing cognitive effort in challenging listening environments.

✅ Final Answer: C. Directional microphones and digital noise reduction both significantly reduce cognitive effort in noisy environments.

Question 13: ANSI S3.22 Standard for Defining Hearing Aid Frequency Range

Understanding Frequency Range Definition in ANSI S3.22

The ANSI S3.22 standard sets guidelines for measuring hearing aid performance, including frequency response.

The frequency range of a hearing aid is defined as the range in which gain is at least 20 dB below a reference gain.

This reference gain is based on High-Frequency Average Gain (HFA), which is the average gain measured at 1000, 1600, and 2500 Hz.

Analysis of Answer Choices:

A. Full-on gain → Incorrect

Full-on gain is the maximum output of the hearing aid but is not used to define the frequency range.

B. High-frequency average gain (HFA) → Correct

ANSI S3.22 specifies that the hearing aid frequency range is defined as the range where gain falls 20 dB below the HFA gain.

This ensures a practical measure of usable frequency response.

C. Special purpose average gain → Incorrect

This is used for specific hearing aid fittings but is not the standard reference for defining frequency range.

D. Coupler aided gain → Incorrect

Coupler gain refers to standardized measurements in a test box, but it is not used for defining frequency range under ANSI S3.22.

✅ Final Answer: B. High-frequency average gain (HFA).

Question 14: Hearing Aid Technology in Noisy Environments

Analysis of Answer Choices:

A. Noise reduction technology significantly improves speech recognition in noise → Incorrect

Noise reduction (NR) reduces background noise discomfort but does not dramatically improve speech intelligibility in noise.

NR primarily helps increase listening comfort rather than enhancing speech clarity.

B. Omnidirectional microphones provide a more complete and clearer signal in noise than directional microphones → Incorrect

Omnidirectional microphones pick up sound equally from all directions, making them less effective in noisy environments.

Directional microphones focus on sound from the front, enhancing speech clarity in noise.

C. Reducing sudden loud noises minimizes loudness discomfort but significantly affects speech clarity → Incorrect

Impulse noise reduction protects the listener from sudden loud sounds (e.g., clattering dishes) but does not significantly impact speech clarity.

It is designed to maintain speech intelligibility while preventing discomfort.

D. Hearing aids use two omnidirectional microphones with a first-order subtraction algorithm to create directional effects, improving speech clarity in noise → Correct

Most modern directional microphones in hearing aids work by using two omnidirectional microphones and applying a first-order subtraction algorithm to enhance sounds from the front while reducing background noise.

This technology improves speech perception in noisy environments.

✅ Final Answer: D. Hearing aids often use two omnidirectional microphones with a first-order subtraction algorithm to enhance speech clarity in noise.

Question 15: User Complaints About Their Own Voice with Hearing Aids

Possible Causes of Self-Perceived Voice Changes:

Overamplification of low frequencies can cause a boomy or hollow sound, contributing to the occlusion effect (feeling of hearing one's own voice too loudly).

A blocked ear canal due to an earmold can cause resonance changes and increase the perception of one's voice.

Excessive high-frequency amplification can make a user's voice sound unnaturally sharp or harsh.

Analysis of Answer Choices:

A. The user forgot what their natural voice sounds like → Incorrect (least likely)

While adaptation plays a role, this is not a common primary cause of hearing aid voice complaints.

B. Excessive low-frequency amplification → Correct (possible cause)

Overamplification in low frequencies enhances bone-conducted sound, making the user's voice seem boomy or occluded.

C. Earmold causing excessive ear canal blockage → Correct (possible cause)

A tight-fitting earmold traps low-frequency sound inside the ear canal, worsening the occlusion effect.

D. Excessive high-frequency amplification → Correct (possible cause)

Overamplification of high frequencies may make the user's own voice sound unnaturally sharp or harsh.

✅ Final Answer: A. The user forgot what their natural voice sounds like (least likely cause).

Question 16. Regarding the previous question, which of the following is not a solution?

Correct Answer: D. Reducing the diameter of the existing vent

Explanation:

When fitting a hearing aid, reducing the occlusion effect or improving low-frequency amplification issues can be achieved through several methods:

A. Adding a vent: Helps reduce the occlusion effect and allows for a more natural sound.

B. Using open-fit domes: Reduces low-frequency amplification and improves comfort, especially for high-frequency hearing loss.

C. Using softer earmold materials: Increases wearing comfort and reduces pressure on the ear canal.

However, D. Reducing the diameter of the existing vent actually increases the occlusion effect by trapping more low-frequency sounds inside the ear canal. This would worsen the problem rather than solve it, making it the incorrect solution.

Question 17: Many hearing-impaired individuals also experience tinnitus. Since high-frequency sounds provide the best masking effect for tinnitus, which type of hearing aid is most recommended for a patient with mild sloping high-frequency hearing loss and tinnitus?

Correct Answer: B. Open fitting

Explanation:

Tinnitus is commonly associated with high-frequency hearing loss. Amplifying high frequencies using a hearing aid can help achieve a masking effect, reducing the perception of tinnitus. When choosing a hearing aid for mild sloping high-frequency hearing loss, the following factors are important:

Open fitting (B) allows natural low-frequency sound to enter the ear while providing high-frequency amplification. This reduces the occlusion effect and enhances tinnitus masking.

In-the-ear (ITE) (A) and Completely-in-canal (CIC) (C) hearing aids tend to block the ear canal, which can increase the occlusion effect and may not provide adequate high-frequency amplification.

Behind-the-ear (BTE) hearing aids (D) can be effective but are best when paired with an open-fit dome to prevent occlusion.

Therefore, B. Open fitting is the best option, as it effectively provides high-frequency amplification while maintaining a natural sound experience and reducing occlusion discomfort.

Question 18. For which of the following cases is the impact of signal-to-noise ratio (SNR) least important when fitting a hearing aid?

Correct Answer: A. Otosclerosis

Explanation:

Signal-to-noise ratio (SNR) is crucial for individuals who have difficulty understanding speech in noisy environments. The impact of SNR is particularly significant in conditions that affect speech processing and neural transmission rather than purely mechanical hearing loss. Let’s analyze each option:

A. Otosclerosis: This condition primarily affects the middle ear by causing fixation of the stapes, leading to conductive hearing loss. Since the cochlear and neural pathways remain intact, individuals with otosclerosis generally have good speech discrimination in quiet environments, making SNR less critical for their hearing aid fitting.

B. Presbycusis: Age-related hearing loss typically involves both cochlear damage and central auditory processing decline, making speech perception in noise difficult. Thus, SNR is an important factor.

C. Auditory neuropathy spectrum disorder (ANSD): This disorder affects neural transmission from the cochlea to the brain, leading to poor speech discrimination, especially in noise. SNR is a critical factor when considering hearing aids or cochlear implants.

D. Ménière’s disease: This condition causes fluctuating sensorineural hearing loss, often with distorted speech perception, particularly in noisy environments. SNR is an important factor in fitting hearing aids.

Since otosclerosis primarily affects conductive hearing without significant neural processing deficits, it is the condition least affected by SNR in hearing aid fitting.

Question 19. In infants with auditory neuropathy spectrum disorder (ANSD), which test result best predicts whether they will benefit from hearing aids?

Correct Answer: C. Presence of Cortical Auditory Evoked Potentials (CAEP)

Explanation:

Auditory neuropathy spectrum disorder (ANSD) affects neural synchronization, meaning traditional hearing tests like pure-tone audiometry may not fully reflect speech perception ability. When deciding on hearing aids for infants with ANSD, it is crucial to determine whether the auditory pathway can effectively process amplified sound. Let’s analyze each option:

A. Pure-tone hearing thresholds: These do not reliably predict speech perception in ANSD because neural synchrony issues affect sound processing independently of hearing thresholds.

B. ABR wave V latency: Auditory Brainstem Response (ABR) is often absent or abnormal in ANSD, making it unreliable for predicting hearing aid benefit.

C. Presence of Cortical Auditory Evoked Potentials (CAEP): If CAEP responses are present, it indicates that sound is reaching and being processed at the auditory cortex level, suggesting the infant may benefit from hearing aids. If absent, cochlear implants may be considered.

D. Otoacoustic Emissions (OAE) response: While OAEs are typically present in ANSD, they only reflect outer hair cell function and do not indicate how well the brain processes sound.

Since CAEP presence suggests functional auditory cortical processing, it is the best predictor of hearing aid benefit in infants with ANSD.

Question 20. Regarding the clinical significance of using Speech Intelligibility Index (SII) to verify the benefit of hearing aid fitting in infants, which statement is most appropriate?

Correct Answer: D. SII can be used as a reference for comparing different hearing aids.

Explanation:

The Speech Intelligibility Index (SII) is a numerical representation (ranging from 0 to 1 or 0% to 100%) that predicts how much of the speech signal is audible to a listener with a given hearing loss. It is commonly used in pediatric hearing aid fittings to evaluate the audibility of speech sounds across different frequencies. Let’s analyze each option:

A. SII only predicts performance at a specific test volume in noisy environments → ❌ Incorrect

SII is calculated across multiple input levels and environments, including both quiet and noisy conditions. It is not limited to a single volume or noise setting.

B. A higher SII means the hearing aid settings better match the infant’s gain requirements → ❌ Incorrect

While a higher SII generally correlates with better audibility, it does not always mean the gain settings are optimal. Other factors like comfort, speech distortion, and over-amplification risk must also be considered.

C. SII allows direct comparison of speech clarity between infants and adults → ❌ Incorrect

SII is age-dependent, and the relationship between audibility and speech understanding differs between infants and adults due to developmental differences in auditory processing. Direct comparisons are not valid.

D. SII can be used as a reference for comparing different hearing aids → ✅ Correct

SII provides an objective measurement of the potential speech audibility across different hearing aid models and settings, making it a useful tool for selecting the most appropriate device.

Thus, option D is the most appropriate statement regarding the clinical use of SII in pediatric hearing aid fittings.

Question 21. In a real-ear measurement system, if the hearing threshold is entered in dB HL and the test stimulus is entered in dB SPL, what is most likely to change in the SPL-o-gram when switching the audiometric transducer from insert earphones to supra-aural headphones?

Correct Answer: D. ① Hearing threshold, ② Gain target, ③ Real-ear aided response (REAR)

Explanation:

The SPL-o-gram is a graphical representation of the hearing threshold, speech input levels, and hearing aid responses in real-ear measurement (REM). Changing the earphone type used during audiometric testing affects how thresholds and gain targets are interpreted in dB SPL due to differences in earphone calibration and coupling to the ear. Let's examine each item:

① Hearing threshold → ✅ Changes

Insert earphones and supra-aural headphones have different reference equivalent threshold sound pressure levels (RETSPLs), meaning the conversion from dB HL to dB SPL will be different. This affects how thresholds appear on the SPL-o-gram.

② Gain target (prescription formula target values) → ✅ Changes

Prescriptive fitting formulas (e.g., DSL v5.0, NAL-NL2) rely on hearing thresholds in dB SPL to calculate gain targets. Since switching earphones affects how HL thresholds are converted to SPL, it impacts the calculated gain targets.

③ Real-ear aided response (REAR) → ✅ Changes

REAR represents the hearing aid output in the ear canal. Since the gain targets change, the REAR may also be adjusted to match the new targets.

Since all three elements (hearing threshold, gain target, and REAR) are affected, the correct answer is:

D. ①②③

Question 22. In real-ear measurement (REM) for open-fit hearing aids, if the earmold tip is 2.0 cm from the eardrum and the probe tube tip is 1.6 cm from the eardrum, which statement about measurement error is most reasonable?

Correct Answer: C. The measurement error for 3000 Hz does not exceed 3 dB.

Explanation:

In real-ear measurements, probe tube placement is crucial to ensure accurate sound pressure level readings near the eardrum. If the probe tube is positioned too far from the eardrum, standing waves (caused by sound reflections in the ear canal) can lead to measurement errors, particularly at higher frequencies.

Low frequencies (e.g., 500 Hz) are less affected by probe tube positioning, as their wavelengths are much longer than the probe tube misplacement. (A is incorrect.)

For mid-to-high frequencies (e.g., 2000 Hz and above), probe tube misplacement introduces progressively larger errors due to standing wave interference.

At 2000 Hz, measurement error is typically below 1 dB if the probe is slightly mispositioned. (B is correct in concept but not the best choice compared to C.)

At 3000 Hz, measurement error can be around 2-3 dB if the probe tube is off by 4 mm (as in this case). (C is the most reasonable choice.)

At 4000 Hz, measurement errors may exceed 5 dB due to increased standing wave effects. (D is incorrect, as the error can be greater than 5 dB.)

Since C (error ≤3 dB at 3000 Hz) best aligns with expected real-ear measurement errors, it is the correct answer.

Question 23. Which type of patient is most likely to experience off-frequency listening?

Correct Answer: B. Patients with cochlear dead regions.

Explanation:

Off-frequency listening occurs when a damaged area in the cochlea (e.g., a dead region) prevents sound detection at its corresponding frequency, causing the listener to rely on adjacent, undamaged regions for sound perception. This results in distorted or degraded frequency resolution. Let's examine each option:

A. Auditory neuropathy spectrum disorder (ANSD) → ❌ Incorrect

ANSD is characterized by poor neural synchronization, leading to difficulty in speech perception, especially in noise. However, it does not typically cause off-frequency listening.

B. Patients with cochlear dead regions → ✅ Correct

Dead regions in the cochlea mean that specific inner hair cells and auditory nerve fibers are nonfunctional. To compensate, the auditory system uses neighboring off-frequency regions to detect sound, leading to poorer frequency resolution and speech perception difficulties.

C. Patients with central auditory processing disorder (CAPD) → ❌ Incorrect

CAPD affects how the brain processes auditory signals rather than how the cochlea detects frequency information. These patients typically do not experience off-frequency listening.

D. Patients with conductive hearing loss → ❌ Incorrect

Conductive hearing loss primarily affects sound transmission through the outer/middle ear but does not alter the cochlear frequency-selective mechanisms responsible for off-frequency listening.

Thus, B. Patients with cochlear dead regions are most likely to experience off-frequency listening, making it the correct answer.

Question 24. Which hearing aid fitting formula considers tonal languages (e.g., Chinese) and non-tonal languages (e.g., English) in its calculation methods?

Correct Answer: B. NAL-NL2

Explanation:

Hearing aid prescription formulas are designed to optimize speech intelligibility and listening comfort. Among them, NAL-NL2 is unique in incorporating language differences, including tonal vs. non-tonal languages, into its gain prescription. Let's analyze each option:

A. FIG6 → ❌ Incorrect

FIG6 is an older formula primarily based on hearing threshold levels without considering linguistic differences, including tonal vs. non-tonal languages.

B. NAL-NL2 → ✅ Correct

NAL-NL2 (developed by the National Acoustic Laboratories) specifically adjusts gain settings for tonal languages like Mandarin.

Since tones in tonal languages rely on frequency contours rather than amplitude changes, NAL-NL2 reduces gain for lower frequencies to preserve natural intonation cues and improve speech clarity for tonal language users.

C. LGOB → ❌ Incorrect

LGOB (Lindemann, Gilkey, Ologbese, & Boothroyd) is an older loudness-based prescription formula and does not differentiate between tonal and non-tonal languages.

D. DSL (Desired Sensation Level) → ❌ Incorrect

DSL focuses on audibility and is commonly used for pediatric fittings, but it does not have a separate algorithm for tonal vs. non-tonal languages.

Since NAL-NL2 is the only fitting formula that accounts for tonal language characteristics, it is the correct answer.

Question 25. During which real-ear measurement (REM) procedure should clinicians be mindful of feedback interference?

Correct Answer: A. REAR (Real-Ear Aided Response)

Explanation: Hearing aid feedback occurs when amplified sound leaks out of the ear and is re-amplified by the hearing aid microphone, leading to a whistling sound. Feedback can distort real-ear measurements, especially when assessing the hearing aid output in the ear canal. Let’s analyze each option:

A. REAR (Real-Ear Aided Response) → ✅ Correct

REAR measures the actual sound output of the hearing aid in the ear canal.

If feedback occurs during the test, the measured response will be inaccurate due to excessive gain and unwanted sound artifacts.

Clinicians must monitor for feedback and adjust the hearing aid or earmold fit if necessary.

B. RECD (Real-Ear to Coupler Difference) → ❌ Incorrect

RECD measures the difference between sound levels in the ear canal vs. a standard coupler.

Since the measurement is performed without the hearing aid actively amplifying sound in the ear, feedback is not a concern.

C. REDD (Real-Ear to Dial Difference) → ❌ Incorrect

REDD measures the difference between audiometer dial settings and actual ear canal SPL.

It does not involve the hearing aid in operation, so feedback is not an issue.

D. REOR (Real-Ear Occluded Response) → ❌ Incorrect

REOR measures the attenuation effect of an earmold or hearing aid without amplification.

Since the hearing aid is not actively amplifying sound, feedback is not a concern.

Thus, A. REAR is the correct answer because it involves active amplification, making it the most susceptible to feedback interference during measurement.

Question 26. Regarding the relationship between Real-Ear-to-Coupler Difference (RECD) and Real-Ear-to-Dial Difference (REDD), which statement is incorrect?

Correct Answer: A. RECD = REDD + Reference Equivalent Threshold SPL (RETSPL)

Explanation: To understand the correct relationships between RECD, REDD, RETSPL, and SPL values, let's break down each term:

RECD (Real-Ear-to-Coupler Difference):

Measures the difference between the sound pressure level (SPL) in the ear canal and the SPL in a coupler when presented with the same sound signal.

Formula: RECD=Canal SPL−Coupler SPLRECD = \text{Canal SPL} - \text{Coupler SPL}RECD=Canal SPL−Coupler SPL

REDD (Real-Ear-to-Dial Difference):

Represents the difference between the audiometer dial setting (HL in dB) and the actual SPL measured in the ear canal.

Formula: REDD=Coupler SPL+RECDREDD = \text{Coupler SPL} + RECDREDD=Coupler SPL+RECD

RETSPL (Reference Equivalent Threshold SPL):

A calibration value that converts HL (dB) from the audiometer into SPL in the coupler.

Evaluating Each Answer Choice:

A. RECD = REDD + RETSPL → ❌ Incorrect

The correct relationship is: REDD=RECD+RETSPLREDD = RECD + RETSPLREDD=RECD+RETSPL

This means RECD alone does not equal REDD plus RETSPL.

Instead, REDD accounts for both RECD and RETSPL when converting from HL to SPL.

B. REDD = Coupler SPL – Dial HL → ✅ Correct

REDD represents the difference between the coupler-measured SPL and the audiometer dial setting in HL.

This formula correctly defines REDD.

C. RECD = Canal SPL – Coupler SPL → ✅ Correct

This is the standard definition of RECD: the difference in SPL between the real ear and the coupler.

D. Coupler SPL = Dial HL + RETSPL → ✅ Correct

RETSPL is used to convert HL from the audiometer dial into SPL in a standard coupler.

Since A is incorrect based on the mathematical relationships, it is the correct choice for this question.

Question 27. Which subjective self-assessment questionnaire is best suited to address the problem where patients struggle in specific listening situations and want clearer hearing in those contexts?

Correct Answer: B. Client Oriented Scale of Improvement (COSI)

Explanation: Most hearing aid self-assessment questionnaires present a broad range of hearing difficulties, but patients often experience challenges in only a few specific listening situations. The COSI questionnaire uniquely allows customization to focus on the individual's primary listening goals. Let's analyze the choices:

A. Abbreviated Profile of Hearing Aid Benefit (APHAB) → ❌ Incorrect

APHAB assesses hearing difficulties across standardized listening situations.

While useful for general benefit evaluation, it does not allow customization for a patient’s specific needs.

B. Client Oriented Scale of Improvement (COSI) → ✅ Correct

COSI is a customizable questionnaire where patients identify specific listening situations they struggle with.

It directly tracks improvements based on the patient’s personal hearing goals, making it the best option for this question.

C. Speech, Spatial and Qualities of Hearing Scale (SSQ) → ❌ Incorrect

SSQ assesses multiple aspects of hearing (speech perception, spatial hearing, and sound quality perception).

It is a comprehensive tool but does not focus on patient-specific difficulties.

D. Effectiveness of Auditory Rehabilitation (EAR) → ❌ Incorrect

EAR assesses general effectiveness of hearing rehabilitation programs, rather than focusing on specific hearing difficulties per patient.

Since COSI allows patients to define their own hearing priorities, it is the best answer.

Question 28. Which statement about the International Outcomes Inventory for Hearing Aids (IOI-HA) is correct?

Correct Answer: A. This questionnaire assesses hearing aid users across seven key areas: usage, benefit (activity limitation improvement), satisfaction, residual activity limitation, social impact, other effects, and quality of life improvement.

Explanation: The IOI-HA is a standardized, globally recognized tool designed to evaluate the real-world effectiveness of hearing aids across seven key dimensions:

Usage (How often the hearing aid is used)

Benefit (Improvement in activity limitations)

Satisfaction (Overall satisfaction with the hearing aid)

Residual activity limitation (Limitations that remain despite using the hearing aid)

Social impact (Impact on social interactions)

Other effects (Impact on significant others)

Quality of life improvement

Evaluating Each Answer Choice:

A. The questionnaire explores seven key areas, including hearing aid use, benefit, satisfaction, remaining limitations, social deprivation, other effects, and quality of life improvements. → ✅ Correct

These seven areas form the core framework of IOI-HA.

B. Each of the seven questions contains three sub-items and has been translated into 21 languages. → ❌ Incorrect

The IOI-HA consists of seven main questions, but it does not include three sub-items per question.

While the questionnaire has been translated into multiple languages, the exact number varies.

C. Each question is scored on a scale from 1 to 7, representing the most likely outcomes for hearing aid users. → ❌ Incorrect

IOI-HA uses a five-point scale (1–5) rather than a seven-point scale.

D. The IOI-HA divides questions into two categories, and for every 10 dB increase in better-ear hearing threshold, category one increases by 0.07 points while category two decreases by 0.1 points. → ❌ Incorrect

The IOI-HA does not include a formula that links hearing threshold changes to score changes in this way.

Since option A correctly describes the structure and purpose of IOI-HA, it is the correct answer.

Question 29. Which statement about subjective self-assessment questionnaires for children is correct?

Correct Answer: C. Children's Home Inventory of Listening Difficulties (CHILD) is suitable for children aged 3–12 years, with a total of 15 questions.

Explanation: Several pediatric self-assessment questionnaires are used to evaluate the listening experiences and benefits of amplification in children. Let's analyze each option:

A. Client Oriented Scale of Improvement for Children (COSI-C) is suitable for children aged 5 and above, with a total of 10 questions. → ❌ Incorrect

COSI-C is indeed used for children, but it does not have a fixed set of 10 questions.

Instead, it allows customization based on the child's specific listening goals, much like the adult COSI.

B. Meaningful Auditory Integration Scale (MAIS) is suitable for children aged 0.5 to 3 years, with a total of 10 questions. → ❌ Incorrect

MAIS is designed for infants and toddlers (birth to 3 years) to assess auditory behaviors, but it contains 10 main items divided into three categories (device use, spontaneous responses, and communication behaviors).

The description is partially correct, but the wording of the response does not fully align with its structure.

C. Children's Home Inventory of Listening Difficulties (CHILD) is suitable for children aged 3–12 years, with a total of 15 questions. → ✅ Correct

CHILD evaluates listening difficulties in real-world environments (home, school, and social settings).

It is designed for children aged 3–12 years and includes 15 questions, making this the correct answer.

D. Abbreviated Profile of Hearing Aid Performance for Children (APHAP-C) is suitable for children aged 5–10 years, with a total of 24 questions. → ❌ Incorrect

While APHAP exists for adults, APHAP-C is not widely recognized as a standardized pediatric version.

The original APHAP contains 24 questions, but its use for children aged 5–10 is not well established.

Since option C correctly describes the CHILD questionnaire, it is the best answer.

Question 30. Which questionnaire allows for a comparison of performance before and after hearing aid use?

Correct Answer: A. Abbreviated Profile of Hearing Aid Benefit (APHAB)

Explanation:

A good hearing aid verification questionnaire should measure performance in unaided and aided conditions to assess the improvement provided by the hearing aid. Let’s evaluate each option:

A. Abbreviated Profile of Hearing Aid Benefit (APHAB) → ✅ Correct

APHAB measures the percentage of difficulty in different listening environments both before and after using hearing aids.

The difference in scores provides a direct measure of hearing aid benefit, making it the best choice for this question.

B. Hearing Aid Review (HAR) → ❌ Incorrect

HAR focuses on user satisfaction and experiences after wearing hearing aids.

It does not compare pre- and post-hearing aid performance.

C. Shortened Hearing Aid Performance Inventory for the Elderly (SHAPIE) → ❌ Incorrect

SHAPIE is a simplified version of HAPI, designed for older adults, but it does not assess unaided vs. aided conditions directly.

D. Hearing Aid Performance Inventory (HAPI) → ❌ Incorrect

HAPI evaluates hearing aid performance but does not explicitly compare pre- and post-hearing aid use.

It is more focused on long-term satisfaction and adaptation.

Since APHAB measures unaided vs. aided listening conditions, it is the best tool for comparing hearing aid performance before and after fitting.

Question 31. According to ANSI S3.22 hearing aid testing standards, which statement about testing the OSPL90 (output sound pressure level for a 90 dB SPL input) frequency response curve is incorrect?

Correct Answer: B. A 90 dB SPL speech input signal is used.

Explanation:

ANSI S3.22 specifies standardized procedures for hearing aid testing, including the OSPL90 test, which measures the maximum output level of a hearing aid. Let’s break down each option:

A. The hearing aid volume control should be set to the full-on position. → ✅ Correct

OSPL90 is measured with the hearing aid set to its maximum volume (full-on gain) to determine the highest possible output level.

B. A 90 dB SPL speech input signal is used. → ❌ Incorrect

For OSPL90 testing, a pure-tone sweep or a tone burst signal is used, not a speech input signal.

The purpose is to measure the hearing aid’s maximum output across frequencies, not speech processing.

90 dB SPL is the input level, but it’s not speech—it’s a continuous tone or swept-tone signal.

C. This test can estimate the maximum output level of the hearing aid. → ✅ Correct

The OSPL90 curve provides critical information on the maximum output the hearing aid can deliver without distortion, ensuring it doesn't exceed a comfortable listening level.

D. The high-frequency average (HFA) result must be within 4 dB SPL of the manufacturer's specifications. → ✅ Correct

The ANSI standard requires that the HFA OSPL90 values measured should fall within ±4 dB SPL of the values specified by the manufacturer to ensure consistency and reliability.

Since option B incorrectly states that a speech input signal is used, it is the correct answer for being incorrect according to ANSI S3.22 standards.

Question 32. What is the main benefit of using the Fine Structure Processing (FSP) strategy in cochlear implants?

Correct Answer: B. Enhancing temporal and spectral resolution in the low-frequency range.

Explanation:

Fine Structure Processing (FSP) is an advanced coding strategy used in cochlear implants to enhance the perception of sound, particularly in the low-frequency range, which is crucial for music appreciation and pitch perception. Let's examine each option:

A. Enhancing temporal resolution across all frequency bands. → ❌ Incorrect

While FSP does improve temporal processing, its primary focus is on low-frequency enhancement rather than across all frequency bands.

The goal is to improve the perception of fine temporal cues in the low frequencies, essential for pitch and melody recognition.

B. Enhancing temporal and spectral resolution in the low-frequency range. → ✅ Correct

FSP enhances the temporal fine structure information in low frequencies, providing users with a more natural sound quality and better pitch perception.

This enhancement is particularly beneficial for music appreciation and the understanding of tonal languages, where fine temporal cues are crucial.

C. Improving only frequency resolution. → ❌ Incorrect

FSP improves both temporal and spectral resolution, particularly in the low-frequency range.

Limiting the benefit to only frequency resolution does not fully capture the technology's advantage.

D. Increasing the number of virtual stimulation channels. → ❌ Incorrect

While some coding strategies aim to increase the number of virtual channels, FSP primarily focuses on refining the quality of temporal and spectral information rather than increasing channel count.

Since option B accurately describes the dual benefit of improved temporal and spectral resolution in the low frequencies, it is the correct answer.

Question 33: HiResolution (HiRes) Sound Processing Strategy in Cochlear Implants

The most appropriate answer is:

D. Theoretically provides more details of speech variations over time.

Explanation:

HiResolution (HiRes) is an advanced sound processing strategy used in cochlear implants, particularly developed by Advanced Bionics. It aims to enhance the temporal and spectral resolution of sound, allowing for a more natural auditory experience.

Option A: Incorrect. HiRes does not increase the number of electrodes but utilizes the same set. However, it operates at a higher stimulation rate than Continuous Interleaved Sampling (CIS), improving temporal resolution.

Option B: Incorrect. HiRes does not specifically lower the cutoff frequency; rather, it improves temporal fidelity to enhance speech perception.

Option C: Partially correct but not the best answer. While HiRes involves a high stimulation rate, it does not necessarily sacrifice the number of simultaneously active electrodes. However, simultaneous stimulation may be limited to reduce channel interactions.

Option D: Correct. The primary goal of HiRes is to provide finer temporal and spectral details of speech, theoretically leading to better speech perception, especially in challenging listening environments.

Question 34: Current Steering in Cochlear Implants

The correct answer is:

B. Frequency domain.

Explanation:

Current steering is a technique used in cochlear implants to improve frequency resolution. It involves the simultaneous stimulation of adjacent electrodes with varying current levels to create "virtual channels," effectively increasing the number of perceivable frequency channels.

Option A (Intensity domain): Incorrect. Current steering does not primarily affect intensity coding but rather improves frequency resolution.

Option B (Frequency domain): Correct. Current steering enhances spectral resolution by interpolating between fixed electrode locations, allowing for finer frequency discrimination.

Option C (Time domain): Incorrect. While cochlear implants process signals in the time domain, current steering specifically targets spectral (frequency) resolution rather than temporal processing.

Option D (Spatial domain): Incorrect. Although current steering involves spatial aspects of electrode stimulation, its primary effect is on frequency perception, not spatial localization.

By improving spectral resolution, current steering enhances speech understanding, particularly in noisy environments and for tonal languages like Mandarin.

Question 35: Definition of Electrical Threshold in Cochlear Nucleus Implant

The correct answer is:

B. The electrical stimulation level that can be detected with 50% accuracy.

Explanation:

In Cochlear Corporation's Nucleus cochlear implants, the electrical threshold (T-level) refers to the lowest electrical current that a user can detect 50% of the time. This threshold is used to set the lower boundary of the implant's programming range, ensuring that soft sounds are audible but not too faint.

Option A: Incorrect. 100% detection is not required for defining the electrical threshold; it is based on 50% accuracy in detection.

Option B: Correct. The T-level is defined as the minimum electrical current at which the user can detect the signal 50% of the time.

Option C: Incorrect. While T-level is the smallest detectable signal, it is not based on 100% detection, but rather on a 50% detection rate.

Option D: Incorrect. This option describes speech discrimination rather than electrical threshold measurement.

Question 36: Purpose of Mapping High Frequencies to the Cochlear Base and Low Frequencies to the Apex

The correct answer is:

A. To match the cochlea’s natural tonotopic organization.

Explanation:

The cochlea has a tonotopic organization, meaning different frequencies are naturally processed at different locations along its structure:

High-frequency sounds are detected at the basal (bottom) region of the cochlea.

Low-frequency sounds are detected at the apical (top) region of the cochlea.

Cochlear implants follow this natural arrangement to enhance speech perception and sound quality.

Option A: Correct. The purpose of this frequency mapping is to align with the cochlea's natural tonotopic organization, which improves sound perception and speech recognition.

Option B: Incorrect. While electrical field interactions can occur between electrodes, the primary reason for this frequency mapping is to follow the natural tonotopic structure, not just to avoid interference.

Option C: Incorrect. Although modern cochlear implants use strategies to improve signal representation, the stimulation method remains pulsatile, not purely analog.

Option D: Incorrect. Preserving natural sound quality is important, but the main goal of this mapping is to match the cochlea's tonotopic organization, rather than just preventing unnatural sound quality.

By following the natural frequency distribution of the cochlea, cochlear implants can provide more natural and effective auditory perception for users.

Question 37: Situations Where Cochlear Implantation Can Proceed Without Hearing Aid Trial

The correct answer is:

B. Cochlear ossification due to meningitis.

Explanation:

A hearing aid trial of 3–6 months is typically recommended before considering cochlear implantation to assess the benefits of amplification. However, there are exceptions where immediate cochlear implantation is necessary or preferable:

Option A (Sudden hearing loss): Incorrect. Although sudden hearing loss may be severe, a hearing aid trial is often still considered to evaluate any residual hearing benefit before implantation.

Option B (Cochlear ossification due to meningitis): Correct. Meningitis-induced cochlear ossification can lead to rapid bone formation within the cochlea, which can make implantation difficult or impossible if delayed. Immediate implantation is recommended to prevent the cochlea from becoming fully ossified, thus preserving the opportunity for effective implantation.

Option C (Hearing loss after cholesteatoma surgery): Incorrect. While cholesteatoma can cause significant hearing loss, a hearing aid trial is typically still conducted unless ossification or other complications are present.

Option D (Hearing loss due to stroke): Incorrect. Hearing loss from a stroke is often central (affecting the brain rather than the cochlea) and may not benefit from cochlear implantation at all, but a hearing aid trial is still considered to assess residual hearing function.

Question 38: Hearing Assistance Technology (HAT) and Cochlear Implants

The most inappropriate statement is:

C. Using a cochlear implant with HAT does not help with speech perception in noise.

Explanation:

Hearing Assistance Technology (HAT) such as Telecoil, FM systems, and Bluetooth can be effectively used with cochlear implants to enhance listening in various environments, particularly in noise.

Option A: Correct. Telecoil can be used with cochlear implants to connect to hearing loops, enhancing listening in public places like theaters and churches.

Option B: Correct. FM systems can connect to cochlear implants via adapters, providing direct audio input from microphones or other audio sources, which is especially helpful in classrooms or noisy settings.

Option C: Incorrect. This statement is false because HAT devices are designed to improve speech perception in noise, which is a significant challenge for cochlear implant users. Utilizing these technologies can greatly enhance the listening experience in challenging acoustic environments.

Option D: Correct. When connecting HAT to a cochlear implant, it is important to adjust the audio-mixing ratios to balance the external audio input with the environmental sounds processed by the implant, ensuring optimal hearing in different situations.

By incorporating HAT with cochlear implants, users can achieve better speech understanding in noise, improved access to media, and greater ease of communication in diverse environments.

Question 39: Frequency Allocation in Cochlear Implants

The most appropriate answer is:

C. When certain electrodes must be deactivated, frequency allocation should be carefully considered.

Explanation:

Frequency allocation in cochlear implants determines how different frequencies of sound are assigned to specific electrodes along the cochlea. Proper allocation ensures that users receive an optimal representation of speech and environmental sounds.

Option A: Incorrect. Infants typically receive a broader frequency allocation range rather than a narrower one because early auditory stimulation is crucial for speech and language development.

Option B: Incorrect. Wider channel bandwidths can lead to a loss of spectral detail, reducing speech clarity. Narrower bandwidths improve frequency resolution, which is beneficial for distinguishing different speech sounds.

Option C: Correct. When certain electrodes must be turned off due to malfunction, non-auditory stimulation, or poor response, the frequency allocation must be adjusted to ensure that missing frequency information is reassigned to functioning electrodes. This helps maintain speech perception and sound quality.

Option D: Incorrect. While adult cochlear implant users typically have an upper frequency limit of around 8000 Hz, rather than 4000 Hz, the exact range varies depending on the implant manufacturer and programming.

Question 40: Cochlear Implant Mapping – Factors Affecting Intensity Domain

The correct answer is:

A. Frequency allocation.

Explanation:

Cochlear implant mapping involves setting stimulation parameters to optimize a user's hearing experience. The intensity domain relates to how the implant processes loudness, which is controlled by stimulation levels.

Option A (Frequency allocation): Correct. Frequency allocation determines which electrodes process specific frequencies but does not directly influence loudness perception (intensity domain). Instead, it affects pitch perception and spectral resolution.

Option B (Stimulation levels): Incorrect. Stimulation levels determine the strength of the electrical pulses, which directly impacts loudness.

Option C (Threshold of stimulation, T-levels): Incorrect. T-levels define the softest sounds that a user can detect, affecting loudness perception.

Option D (Upper-stimulation levels, C/M-levels): Incorrect. These levels determine the loudest comfortable sound and are essential for intensity mapping.

Thus, frequency allocation affects pitch perception but does not impact the intensity (loudness) domain, making Option A the correct answer.

Question 41: First Step in Cochlear Implant Programming (CI Programming)

The correct answer is:

D. Electrode impedance testing.

Explanation:

Cochlear implant programming (or mapping) is a step-by-step process that ensures the device is functioning properly and optimally adjusted for the user's hearing needs. The first step in this process is to check the electrode impedance to ensure that all electrodes are functioning properly and are making good contact with the cochlea.

Option A (T-level testing): Incorrect. T-level (threshold level) testing, which determines the softest sound the user can hear, is important but is not the first step.

Option B (C-level testing): Incorrect. C-level (comfort level) testing determines the loudest comfortable sound level, but it is performed later in the mapping process.

Option C (Loudness balancing test): Incorrect. This test is done after T-level and C-level testing to ensure smooth loudness perception across electrodes.

Option D (Electrode impedance testing): Correct. The first step in programming a cochlear implant is measuring electrode impedance to ensure all electrodes are functioning properly before setting stimulation levels.

Question 42: Effect of Adjusting Stimulation Rate on Mapping Parameters

The correct answer is:

A. Frequency allocation.

Explanation:

Stimulation rate refers to how quickly electrical pulses are delivered to the electrodes in a cochlear implant. Changes in stimulation rate primarily affect temporal resolution and can impact speech clarity and pitch perception, but they do not alter the fundamental frequency allocation, which is pre-determined by the programming software.

Option A (Frequency allocation): Correct. Frequency allocation refers to how different sound frequencies are assigned to specific electrodes, and it remains independent of the stimulation rate.

Option B (T-level): Incorrect. Adjusting the stimulation rate can affect perceived loudness, which may require adjustments to the threshold (T-level) to ensure audibility.

Option C (C-level): Incorrect. A higher stimulation rate may increase perceived loudness, requiring modifications to the comfort level (C-level) settings.

Option D (Dynamic range): Incorrect. Since both T-level and C-level can be affected by stimulation rate changes, the dynamic range (C-level minus T-level) can also be affected.

Thus, frequency allocation remains unchanged when adjusting the stimulation rate, making Option A the correct answer.

Question 43: The pulse width in cochlear implant stimulation refers to the duration of a single electrical pulse applied to an electrode. It is measured in microseconds (μs) and affects how electrical current is delivered to the auditory nerve.

Identifying Pulse Width in the Diagram:

Pulse width is the horizontal distance representing the duration of the pulse at a given amplitude.

In the provided diagram, Option C (③) appears to mark a horizontal segment of a pulse, which is likely indicating the pulse width.

Answer:

C. ③

Explanation:

① and ④: These likely represent pulse phases (e.g., negative and positive phases of a biphasic pulse).

②: This might indicate an artifact or leading/trailing edge of the pulse, but it does not represent pulse width.

③: This correctly represents the pulse width, which is the duration of one phase of the biphasic stimulation waveform.

In cochlear implants, adjusting pulse width can impact stimulation intensity and temporal resolution of sound processing, making it an essential parameter in CI mapping.

Question 44: Proper Placement of FM Microphones

The most appropriate answer is:

B. Placing the microphone and transmitter at the collar is more ideal than attaching it at the waist.

Explanation:

FM systems enhance speech perception in challenging listening environments, such as classrooms, by transmitting the speaker's voice directly to the listener's hearing device. Proper microphone placement is essential for optimal speech clarity.

Option A: Incorrect. Headset microphones generally provide better sound quality than clip-on (lapel) microphones because they remain consistently positioned near the mouth, reducing background noise.

Option B: Correct. The closer the microphone is to the speaker's mouth, the better the speech clarity. Placing it on the collar ensures a more stable and optimal distance (15–20 cm from the mouth), whereas waist-level placement reduces signal quality due to increased background noise.

Option C: Incorrect. Placing a built-in microphone inside clothing muffles the sound and reduces speech clarity, making it unsuitable for use.

Option D: Incorrect. Many FM microphones have directional settings, which can focus on the speaker's voice while reducing background noise. This means they are not limited to only collar placement.

Question 45: FM System Functionality and Best Practices

The most appropriate answer is:

A. FM systems improve signal quality and speech clarity better than any hearing aid processing alone.

Explanation:

FM systems are designed to improve the signal-to-noise ratio (SNR) by delivering speech directly to the listener's hearing device, bypassing environmental noise and distance issues.

Option A: Correct. FM systems outperform hearing aid signal processing alone in improving speech clarity, especially in noisy environments, because they reduce background noise and eliminate distance-related sound degradation.

Option B: Incorrect. FM systems are beneficial for both children and adults, including those in workplaces, conferences, and social gatherings—not just for children.

Option C: Incorrect. Multiple transmitters can be used (e.g., in classrooms where multiple speakers may need to be heard), and restricting to a single transmitter is unnecessary.

Option D: Incorrect. While frequent use is beneficial, FM systems should be used strategically based on the listening environment (e.g., in noisy settings, classrooms, meetings) rather than being worn at all times.

Thus, FM systems significantly enhance speech understanding beyond hearing aid processing alone, making Option A the best choice.

Question 46: Reverberation Radius in a Standard Classroom

The most appropriate answer is:

B. The reverberation radius can be used to estimate reverberation time.

Explanation:

The reverberation radius (also called the critical distance) is the point in a room where the direct sound from the speaker is equal in intensity to the reverberant (reflected) sound. It is an important concept in classroom acoustics as it affects speech intelligibility.

Option A: Incorrect. The height of the ceiling affects room acoustics and can influence the reverberation radius by changing how sound waves reflect. Higher ceilings often lead to longer reverberation times, reducing speech clarity.

Option B: Correct. The reverberation radius is related to the reverberation time (RT60), which measures how long it takes for a sound to decay by 60 dB. Knowing the reverberation radius can help acousticians estimate RT60 and make necessary adjustments to improve classroom acoustics.

Option C: Incorrect. The reverberation radius does change with the teacher's speaking volume. If the teacher speaks louder, the direct sound travels farther before becoming equal to the reverberant sound, thereby increasing the reverberation radius.

Option D: Incorrect. Different frequencies have different reverberation behaviors due to their wavelengths. Low-frequency sounds tend to have longer reverberation radii because they are less absorbed by walls and surfaces, while high-frequency sounds decay faster.

Thus, Option B is the correct answer because the reverberation radius is used to estimate reverberation time, which impacts speech intelligibility in classrooms.

Question 47: ANSI/IEEE C63.19 Standard for Hearing Aid and Mobile Phone Compatibility

The most appropriate answer is:

C. Both mobile phones and hearing aids are tested.

Explanation:

The ANSI/IEEE C63.19 standard is used to assess the electromagnetic compatibility (EMC) of hearing aids and mobile phones to ensure they work together without causing interference.

Option A: Incorrect. The standard tests both mobile phones and hearing aids, not just mobile phones.

Option B: Incorrect. The standard also tests mobile phones because their radio frequency (RF) emissions can cause interference with hearing aids.

Option C: Correct. The ANSI/IEEE C63.19 standard evaluates both hearing aids and mobile phones, ensuring that hearing aids are resistant to interference and that mobile phones meet electromagnetic compatibility requirements.

Option D: Incorrect. Both devices are tested to ensure compatibility and minimal interference.

Thus, Option C is the correct answer, as the standard covers both hearing aids and mobile phones to ensure better communication accessibility for hearing aid users.

Question 48: Regarding the advantages of middle ear implant hearing aids compared to traditional air conduction hearing aids, which of the following statements is the least appropriate?

Answer: B. Provides higher frequency response

Explanation:

Middle ear implant (MEI) hearing aids have several advantages over traditional air conduction hearing aids, including:

No feedback issues: Since MEI directly transmits vibrations to the middle ear structures, it eliminates acoustic feedback caused by the proximity of the microphone and receiver in traditional hearing aids. Thus, Option A is correct.

No occlusion effect: Since MEI does not require an earmold to block the ear canal, it avoids the low-frequency amplification discomfort often experienced with traditional hearing aids. Option C is correct.

Better speech clarity in quiet and noisy environments: MEI provides a more natural sound transmission without being affected by ear canal resonance or shape, potentially enhancing speech recognition in various environments. Option D is correct.

However, MEI does not necessarily provide a higher frequency response (Option B). The frequency response of a hearing aid is limited by its design and the physiological properties of the human auditory system. High-frequency sound transmission still faces challenges in middle and inner ear mechanics, making Option B the least appropriate statement.

Question 49: Which of the following is a common function of modern digital hearing aids?

Answer: B. Reduces the dynamic range of sound signals

Explanation:

Modern digital hearing aids focus on improving speech intelligibility, reducing noise interference, and enhancing listening comfort. Let's analyze each option:

A (Improves the unaided air conduction hearing threshold of the wearer): Hearing aids amplify sounds to help individuals with hearing loss perceive them, but they do not improve the wearer's unaided hearing threshold (i.e., hearing ability without the hearing aid). Thus, Option A is incorrect.

B (Reduces the dynamic range of sound signals): Modern digital hearing aids typically use wide dynamic range compression (WDRC) technology to compress the wide range of environmental sound intensities into the user's audible range. This prevents excessive loudness while maintaining speech clarity. Thus, Option B is correct.

C (Increases the auditory system's frequency resolution) & D (Improves cochlear frequency selectivity):

While hearing aids use spectral analysis to adjust amplification across different frequencies, they do not physiologically restore the cochlea's frequency resolution or selectivity. Damage to inner ear hair cells affects frequency discrimination, which cannot be fully restored by hearing aids. Thus, Options C and D are incorrect.

Considering these points, the correct answer is B.

Question 50: When evaluating speech perception scores in noise for a unilateral hearing aid user using an FM system in a sound field, which issue should be avoided to ensure accurate assessment?

Answer: A. Head shadow effect

Explanation: When evaluating speech perception in noise for a unilateral hearing aid user with an FM system in a sound field, certain acoustic effects can interfere with accurate assessment. Let's analyze each option:

A. Head shadow effect (頭影效應): The head creates a physical barrier to sound waves, particularly affecting high-frequency sounds, which have shorter wavelengths and are more easily attenuated. In a unilateral fitting, if the signal source is positioned on the unaided ear's side, the head shadow effect can reduce speech clarity and distort evaluation results. This is a critical factor to avoid during speech perception testing, as it can lead to misleadingly poor scores.

Thus, Option A is the correct answer.

B. Far-field effect (遠場效應): In a far-field situation, sound intensity decreases with distance due to the inverse square law. FM systems are specifically designed to minimize the far-field effect by transmitting the speaker's voice directly to the hearing aid, maintaining a consistent signal-to-noise ratio (SNR). While the far-field effect is relevant in general hearing aid use, it is not a primary concern in FM-assisted speech perception testing.

C. Standing wave effect (駐波效應): Standing waves occur when sound waves interfere with their reflections, leading to alternating zones of high and low intensity in a room. This can affect frequency response in a sound field but is usually managed with proper speaker placement and room acoustics. It is less directly relevant to FM system speech perception testing.

D. Harmonic effect (諧波效應): Harmonic effects relate to overtones produced by a sound source. While harmonics influence sound quality, they do not significantly impact speech perception scores in noise evaluations.

Conclusion: Among the listed factors, the head shadow effect is the most critical issue to avoid, as it can significantly distort speech perception results for a unilateral hearing aid user. Thus, the correct answer is A.

5106

Question 1: What is the correct order of the counseling process when providing counseling to individuals with hearing loss?

Answer: C. ②①④③⑤⑥

Explanation:

Effective counseling for individuals with hearing loss follows a structured process to ensure that they receive appropriate support, understand their condition, and take responsibility for their rehabilitation. The correct sequence is:

② Help the individual tell their story – The first step in counseling is to allow the individual to express their concerns, emotions, and experiences related to their hearing loss. This helps establish rapport and provides insight into their psychological and emotional state.

① Help the individual clarify the problem – Once the story has been shared, the next step is to help them identify specific difficulties they face due to hearing loss, such as communication challenges or emotional distress.

④ Help the individual take responsibility for their hearing problem – It is crucial that the individual acknowledges their hearing loss and understands that active participation is needed for successful rehabilitation.

③ Help the individual set rehabilitation goals – Setting clear and achievable goals ensures that the individual is motivated to improve their communication abilities and quality of life.

⑤ Develop a plan – Based on the established goals, a rehabilitation plan is created, which may include assistive devices, communication strategies, and auditory training.

⑥ Implement the plan – The final step is executing the rehabilitation plan and monitoring progress.

Thus, the correct order is ②①④③⑤⑥, making Option C the correct answer.

Question 2: When evaluating the effectiveness of an adult's aural rehabilitation plan based on hearing aid usage, which factor is least relevant?

Answer: D. Proficiency in using the hearing aid

Explanation:

When assessing the effectiveness of an aural rehabilitation plan, audiologists consider various aspects of hearing aid usage. Let’s analyze each option:

A. Duration of use (使用時長) – The amount of time the individual wears their hearing aid daily is a crucial factor in determining its effectiveness. Longer usage typically leads to better auditory adaptation and improved communication.

B. Listening environments (使用情境) – Understanding where and in what situations the individual uses their hearing aid helps evaluate its real-world benefits and identify any areas where additional support is needed.

C. Use of communication strategies (溝通策略的使用) – Effective hearing rehabilitation involves more than just hearing aids; individuals must also employ strategies like lip-reading, positioning, and repair strategies to enhance communication.

D. Proficiency in using the hearing aid (使用助聽器時的熟練度) – While being able to operate a hearing aid properly is important, it does not directly measure the success of the rehabilitation plan. The ultimate goal is improved communication and quality of life, not just technical proficiency.

Since Option D is the least relevant to assessing rehabilitation effectiveness, it is the correct answer.

Question 3: Regarding presbycusis (age-related hearing loss), which statement is the least appropriate?

Answer: D. Using hearing aids can resolve auditory processing problems.

Explanation:

Presbycusis is a progressive, sensorineural hearing loss associated with aging, primarily affecting high-frequency sounds and often accompanied by central auditory processing difficulties. Let’s analyze each option:

A. Phonemic regression is a common issue in presbycusis – Correct. Phonemic regression refers to a disproportionate difficulty in speech understanding, especially in noisy environments, which is not solely explained by the degree of hearing loss. This is due to central auditory processing decline in addition to peripheral hearing loss.

B. It may involve both peripheral and central auditory processing problems – Correct. Presbycusis often includes damage to the cochlea (peripheral) and dysfunction in the central auditory pathways, affecting speech perception even with amplification.

C. High-frequency sounds are usually harder to hear – Correct. Age-related hearing loss typically affects high-frequency hearing first, making it difficult to hear consonants such as /s/, /f/, and /th/, which are crucial for speech clarity.

D. Using hearing aids can resolve auditory processing problems – Incorrect. While hearing aids amplify sounds and improve audibility, they do not directly address central auditory processing deficits. Even with amplification, individuals with presbycusis may still struggle with speech perception, especially in noisy environments. Additional auditory training or assistive listening devices may be needed.

Thus, the least appropriate statement is D.

Question 4: In the CORE and CARE audiologic rehabilitation model, which CORE assessment component evaluates IQ, age, race, and gender?

Answer: C. Related personal factors

Explanation:

The CORE model is used to evaluate individuals before developing a rehabilitation plan. The four components are:

C (Communication status) – Assesses auditory ability, speechreading skills, and overall communication function.

O (Overall participation variables) – Evaluates the impact of hearing loss on social roles, work, and daily activities.

R (Related personal factors) – Includes individual characteristics such as age, gender, IQ, race, personality, and motivation, which influence rehabilitation outcomes.

E (Environmental factors) – Considers social support, work and living environment, and accessibility to hearing solutions.

Since IQ, age, race, and gender fall under personal characteristics, the correct answer is C. Related personal factors.

Question 5: According to the WHO (2001) International Classification of Functioning, Disability, and Health (ICF) framework, which statement is most related to participation restriction in hearing impairment?

Answer: C. Avoiding social interactions due to fear of not fitting in.

Explanation:

The ICF framework classifies disability into three components:

Impairment (problems with body function/structure, e.g., cochlear damage).

Activity limitation (difficulty performing tasks, e.g., speech understanding).

Participation restriction (difficulty engaging in life situations due to impairment).

Let’s analyze each option:

A. Low speech discrimination test score (<60%) – Incorrect. This measures impairment (functional hearing ability) rather than participation restriction.

B. Inability to respond in a quiet environment – Incorrect. This describes an activity limitation rather than a broader impact on participation.

C. Avoiding social interactions due to fear of not fitting in – Correct. Participation restriction refers to real-world social and emotional consequences of hearing loss, such as withdrawing from conversations, social isolation, or reduced involvement in activities.

D. Poor hearing aid benefit – Incorrect. While this can contribute to participation restriction, it primarily describes a functional limitation.

Since Option C directly describes a social participation issue, it is the correct answer.

Question 6: For an adult with hearing loss, which type of cue is most likely to improve their speech perception of the sentence: "很多人喜歡去夜市吃臭豆腐" (Many people like to go to the night market to eat stinky tofu)?

Answer: A. Providing "夜市" (night market) as a cue.

Explanation:

Speech perception can be improved with semantic, contextual, or linguistic cues. Let’s evaluate each option:

A. Providing "夜市" (night market) as a cue – Correct. This is a semantic cue, helping the listener anticipate the topic and likely words, making speech perception easier.

B. Giving "12 characters and six rhythmic cues" – Incorrect. While rhythm helps with speech segmentation, it does not provide meaning to aid recognition.

C. Asking, "What do you dislike doing?" – Incorrect. This is misleading because the sentence does not relate to dislikes.

D. Removing coarticulation effects (e.g., adding a pause between "豆" and "腐") – Incorrect. While this may enhance clarity, coarticulation is natural in fluent speech, and pauses may disrupt comprehension rather than help.

Since Option A provides a meaningful contextual clue, it is the most effective for improving speech perception.

Question 7: For a child with moderate sensorineural hearing loss using bilateral hearing aids, which pair of consonants would be the most difficult to distinguish?

Answer: A. /ㄊ/ (t) and /ㄎ/ (k)

Explanation: Children with moderate sensorineural hearing loss (SNHL) typically have reduced audibility of high-frequency sounds and poorer speech discrimination, especially for consonants that rely on high-frequency energy. Let’s analyze each option:

A. /ㄊ/ (t) and /ㄎ/ (k) – Most difficult

Both /ㄊ/ and /ㄎ/ are unvoiced plosives with high-frequency acoustic energy. /ㄊ/ (t) is an alveolar stop, while /ㄎ/ (k) is a velar stop, and both have weak low-frequency energy, making them harder to perceive, especially in noise. These sounds are primarily distinguished by high-frequency burst cues, which are often inaudible or distorted for children with moderate SNHL.

B. /ㄇ/ (m) and /ㄏ/ (h) – Easier to distinguish

/ㄇ/ (m) is a nasal consonant, and /ㄏ/ (h) is a fricative. Nasals have low-frequency energy, which remains more audible for individuals with moderate SNHL. /ㄏ/ (h) has a broader frequency range but may still be heard better than plosive bursts.

C. /ㄆ/ (p) and /ㄖ/ (r) – Easier to distinguish

/ㄆ/ (p) is a bilabial plosive, and /ㄖ/ (r) is a retroflex approximant. /ㄆ/ has low-frequency energy, making it more accessible. /ㄖ/ has mid-to-low frequency components, making it more distinguishable compared to high-frequency sounds.

D. /ㄈ/ (f) and /ㄋ/ (n) – Easier to distinguish

/ㄈ/ (f) is a fricative, while /ㄋ/ (n) is a nasal. Nasals have strong low-frequency energy, while fricatives, although often high-frequency, have more continuous energy cues that may still be partially perceived.

Conclusion: Among the given choices, /ㄊ/ (t) and /ㄎ/ (k) are the most difficult to differentiate for children with moderate sensorineural hearing loss due to their reliance on high-frequency burst cues, which are commonly affected by SNHL.

Thus, the correct answer is A. /ㄊ/、/ㄎ/.

Question 8: Which statement about speech perception assessment for individuals with hearing loss is the most appropriate?

Answer: C. Background noise can be included in adult speech perception tests.

Explanation: Speech perception assessments evaluate how well individuals with hearing loss recognize and understand speech. Let’s analyze each option:

A. "Infants and young children cannot be assessed" – Incorrect.

While traditional speech perception tests require verbal responses, young children and infants can be assessed using specialized methods such as:

Visual Reinforcement Audiometry (VRA)

Conditioned Play Audiometry (CPA)

Electrophysiological measures (e.g., cortical auditory evoked potentials)

Speech perception testing is possible even in very young children with appropriate techniques.

B. "Assessment tools must match the degree of hearing loss" – Partially correct but not the best answer.

While it is important to select appropriate materials based on the individual's hearing loss, speech perception testing also evaluates real-world communication ability, which may not always correlate directly with pure-tone thresholds.

Some tests assess residual hearing or aided performance to guide rehabilitation.

C. "Background noise can be included in adult tests" – Correct.

In real-life situations, speech is often heard in noisy environments.

Testing in background noise (e.g., speech-in-noise tests like QuickSIN or HINT) assesses an individual's ability to recognize speech in challenging listening conditions.

This provides valuable insights into the effectiveness of hearing aids and cochlear implants.

D. "No visual cues should be provided during testing" – Incorrect.

The presence or absence of visual cues (e.g., lipreading, facial expressions) depends on the test’s objective.

Some assessments specifically measure auditory-only perception, while others evaluate audiovisual integration (e.g., CUNY Sentence Test).

Thus, the most appropriate answer is C.

Question 9: Which statement about the speech assessment and intervention for children with profound hearing loss is the least appropriate?

Answer: B. "Suprasegmental errors vary, but voice quality is often unaffected."

Explanation:

Children with profound hearing loss often exhibit speech production difficulties due to limited auditory feedback. Let’s analyze each option:

A. "Common segmental errors include vowel centralization and final consonant omission" – Correct.

Profoundly deaf children often neutralize vowels, making them sound more like a central vowel.

They also tend to omit final consonants, as these are often high-frequency sounds that are difficult to perceive.

B. "Suprasegmental errors vary, but voice quality is often unaffected" – Incorrect.

Suprasegmental (prosodic) errors include monotone pitch, incorrect stress, and abnormal intonation.

Voice quality is often affected due to poor control of vocal intensity, pitch modulation, and resonance.

Many profoundly deaf children exhibit hoarse, breathy, or nasalized speech.

C. "Early cochlear implantation improves overall speech clarity compared to hearing aids alone" – Correct.

Research shows that children who receive cochlear implants at an early age tend to develop better speech clarity than those who rely solely on hearing aids.

This is because cochlear implants provide more access to high-frequency sounds, which are crucial for speech intelligibility.

D. "Cochlear implantation can improve language form, content, and use" – Correct.

Early cochlear implantation enhances syntax (form), vocabulary (content), and pragmatic (use) skills, leading to better language outcomes.

Children with cochlear implants typically show better grammar, larger vocabularies, and more appropriate social communication skills compared to those using hearing aids alone.

Since Option B is the least appropriate statement, it is the correct answer.

Question 10: Which statement about assessing conversational fluency in individuals with hearing loss is the most appropriate?

Answer: C. "Interview-based assessments make it harder to obtain quantitative data."

Explanation: Conversational fluency assessment evaluates how smoothly a person with hearing loss can engage in a conversation. Let's analyze each option:

A. "Transcription analysis is a structured interactive communication assessment method" – Correct but not the best answer.

Transcription analysis involves recording conversations, transcribing them, and analyzing patterns of communication breakdowns.

It is a qualitative method but may not be the most structured compared to standardized rating scales.

B. "The MLT (Mean Length of Turn) ratio, calculated as the total speaking turns of the hearing-impaired individual divided by another participant, above 10 indicates higher fluency" – Incorrect.

MLT ratio measures conversational turn-taking, but a ratio above 1.0 to 2.0 is typically expected in balanced conversations.

A ratio above 10 would likely indicate dominance in conversation, not necessarily fluency.

C. "Interview-based assessments make it harder to obtain quantitative data" – Correct.

Interviews provide rich qualitative insights but lack structured, numerical data compared to standardized tests or rating scales.

Responses may be subjective and harder to compare across individuals.

D. "The Client Oriented Scale of Improvement (COSI) is a closed-ended questionnaire" – Incorrect.

COSI is an open-ended questionnaire that allows individuals to identify and prioritize their specific communication goals.

It is not a fixed-response (closed-ended) scale like the APHAB or HHIE.

Since Option C best describes the limitation of interview-based assessments, it is the most appropriate answer.

Question 11: Which are common language characteristics of children with hearing loss?

Answer: A. ①②④⑥ (Shorter mean length of utterance, less use of passive sentences, difficulty understanding conversation rules, reduced expressive and receptive vocabulary.)

Explanation: Children with hearing loss often experience delayed language development due to reduced auditory input. Let's analyze each characteristic:

① Shorter Mean Length of Utterance (MLU) – Correct.

MLU refers to the average length of spoken sentences.

Hearing-impaired children tend to produce simpler, shorter sentences due to limited exposure to spoken language.

② Less use of passive sentences – Correct.

Passive sentence structures (e.g., "The book was read by the girl.") are more complex and require greater linguistic exposure, making them harder for hearing-impaired children to acquire.

③ More use of category labels – Incorrect.

Hearing-impaired children often struggle with vocabulary development and may use fewer category labels rather than more.

④ Difficulty understanding conversation rules – Correct.

Pragmatic language skills, such as taking turns, maintaining topics, and repairing communication breakdowns, are often impaired due to limited exposure to spoken interactions.

⑤ Poor role-playing skills – Incorrect.

Many hearing-impaired children can engage in pretend play with appropriate intervention, although their ability to use language within play may be affected.

⑥ Reduced expressive and receptive vocabulary – Correct.

Vocabulary development is one of the biggest challenges for hearing-impaired children, affecting both spoken and written language skills.

Since ①, ②, ④, and ⑥ are the most consistent language characteristics of children with hearing loss, option A is the correct answer.

Question 12: The Least Appropriate Consideration for Increasing the Difficulty of Auditory Test Materials

Answer: C - Changing speaking speed from fast to moderate

Explanation:

Increasing the difficulty of an auditory test typically involves making speech perception more challenging. Let's analyze each option:

A (Changing response mode from multiple-choice to open-ended): Open-ended responses require the listener to recall and articulate words without cues, making the task more difficult than multiple-choice questions.

B (Changing the environment from quiet to noisy): Background noise significantly affects speech comprehension, especially for individuals with hearing loss.

D (Changing the speaker from male to female): Male and female voices have different pitch and frequency characteristics. Female voices are generally higher in pitch, which can be more difficult for individuals with high-frequency hearing loss to understand.

However, C (Changing speaking speed from fast to moderate) is the least appropriate because reducing the speed actually makes speech easier to understand rather than harder. Faster speech increases processing difficulty, while moderate speed improves clarity. Thus, C is the least suitable option for increasing test difficulty.

Question 13: The Least Suitable Statement About Improving Music Perception in Cochlear Implant (CI) Users Through Auditory Training

Answer: C - Cochlear implant users typically train with music that is more bass-heavy

Explanation:

Cochlear implant (CI) users often face challenges in music perception due to limitations in pitch resolution and frequency discrimination. Let's examine each statement:

A (CI users tend to perceive rhythm better than other musical elements): Correct. Rhythm is primarily based on timing cues, which CIs can process relatively well compared to pitch or harmony.

B (The Whole Song Approach mainly focuses on timbre and melody training): Correct. This approach helps CI users improve their perception of overall musical structure rather than just isolated tones.

D (Limitations in place-pitch and rate-pitch coding affect melody perception): Correct. Due to the limited number of electrodes in CIs, fine pitch discrimination is challenging, which affects melody recognition.

However, C (CI users typically train with bass-heavy music) is incorrect because CIs do not favor low frequencies over high frequencies. In fact, low-frequency perception is often more difficult due to limited frequency resolution and electrical stimulation constraints. Instead, CI music training typically focuses on clear melodies, simple rhythms, and distinct instrumentations rather than emphasizing low bass sounds.

Question 14: Factors That Do NOT Predict Lipreading Performance

Answer: D - Duration of Deafness

Explanation:

Lipreading (speechreading) ability varies among individuals, and several cognitive and experiential factors contribute to performance. Let’s analyze each option:

A (Age): Correct. Age can influence lipreading ability, as younger individuals tend to have better neuroplasticity and cognitive adaptability, which aids in visual speech perception.

B (Prelingual deafness): Correct. Individuals who became deaf before acquiring spoken language (prelingual deafness) may develop stronger visual speech perception skills since they rely more on lipreading for communication.

C (Working memory): Correct. Working memory plays a crucial role in processing and retaining visual speech information, especially when filling in gaps due to ambiguous or missing visual cues.

However, D (Duration of deafness) is not a strong predictor of lipreading ability. Some individuals who have been deaf for a long time may develop strong lipreading skills, while others may not, depending on their training, cognitive abilities, and reliance on visual cues. The presence of early exposure and training is more critical than the duration of hearing loss itself.

Question 15: The Least Appropriate Expressive Repair Strategy for Individuals with Hearing Loss

Answer: D - Using questions to confirm the speaker’s topic

Explanation:

Expressive repair strategies help individuals with hearing loss ensure clear communication when their message is misunderstood. Let’s analyze each option:

A (Breaking long sentences into shorter phrases and repeating them): Correct. Shorter phrases are easier to process, reducing cognitive load for both the listener and the speaker.

B (Slowing down speech and enunciating more clearly): Correct. Speaking more slowly and clearly enhances intelligibility, making it easier for others to understand.

C (Using additional gestures): Correct. Non-verbal cues like gestures, facial expressions, and body language help convey meaning and clarify spoken messages.

However, D (Using questions to confirm the speaker’s topic) is the least appropriate because it is more of a receptive repair strategy rather than an expressive repair strategy. Expressive repair strategies focus on making one’s own speech clearer, while asking for confirmation about the speaker’s topic is a way to clarify incoming information, which belongs to receptive strategies.

Question 16: The Most Appropriate Statement About Total Communication (TC)

Answer: A - Also known as simultaneous communication

Explanation:

Total Communication (TC) is an approach that incorporates multiple modes of communication, including speech, sign language, finger spelling, writing, and visual aids, to facilitate communication for individuals with hearing loss. Let's analyze each option:

A (Also known as simultaneous communication): Correct. TC often involves the use of sign language and spoken language simultaneously, which is why it is also called simultaneous communication. It is designed to provide flexible communication options based on the needs of the individual.

B (Uses the sign language of the Deaf community as the first language while also incorporating spoken language of the mainstream society): Incorrect. This description aligns more with bilingual-bicultural (Bi-Bi) approaches, which prioritize sign language (e.g., American Sign Language) as the primary language. TC, on the other hand, does not prioritize sign language over speech; rather, it integrates various communication modes.

C (Emphasizes using spoken language first, then adding natural sign language for clarification): Incorrect. This approach describes cued speech or manually coded systems rather than TC, which does not strictly prioritize spoken language.

D (Emphasizes speaking while simultaneously using natural sign language): Incorrect. While TC encourages simultaneous use of speech and signs, it does not strictly require the use of natural sign language (e.g., ASL or BSL). Instead, TC often incorporates manually coded English (MCE), which follows the grammar and structure of spoken language.

Thus, A is the most appropriate choice.

Question 17: The Least Appropriate Statement About Factors Affecting Speech Intelligibility

Answer: D - For the same sentence, children's speech is clearer when repeating than when imitating

Explanation:

Speech intelligibility can be influenced by various factors, including context, listener experience, and visual cues. Let's evaluate each option:

A (Children's speech intelligibility is higher when reading a passage than when reading unrelated sentences): Correct. Context helps with speech production and listener comprehension, as familiar or meaningful content is easier to articulate clearly.

B (Listeners who have previously heard speech from children with hearing loss are better able to understand them): Correct. Familiarity with a specific speaker's speech patterns can improve comprehension, as listeners can better anticipate pronunciation and articulation patterns.

C (Seeing a child while listening improves intelligibility compared to hearing the voice alone): Correct. Visual cues, such as lip movements and facial expressions, enhance speech perception, particularly for individuals with hearing difficulties.

D (For the same sentence, children's speech is clearer when repeating than when imitating): Incorrect. Imitating (immediate repetition after a model) usually results in clearer speech than spontaneous

repetition because children can directly follow the speaker's articulation and prosody. When repeating from memory, they may introduce more errors, making their speech less intelligible.

Thus, D is the least appropriate statement.

Question 18: Incorrect Statement About the Auditory-Verbal Approach (AVA)

Answer: A - Emphasizes a multisensory teaching approach to promote listening and speaking

Explanation:

The Auditory-Verbal Approach (AVA) is a specialized rehabilitation method designed to maximize the use of residual hearing in individuals with hearing loss, particularly children with cochlear implants or hearing aids. It focuses on auditory-only learning to develop spoken language without relying on visual or tactile cues. Let's analyze each option:

A (Emphasizes a multisensory teaching approach to promote listening and speaking): Incorrect. AVA does not use a multisensory approach (which includes visual/manual cues like lipreading or sign language). Instead, it follows a strictly auditory-first philosophy, training children to listen and develop speech through hearing alone.

B (Guides parents or primary caregivers to be the main educators): Correct. AVA actively involves parents as key facilitators, integrating listening and spoken language strategies into daily life.

C (Uses developmental milestones of typical-hearing children as a reference for goal-setting): Correct. AVA follows a normal auditory and language development framework, helping children acquire language in a way that mimics hearing peers.

D (Uses specific speech techniques, such as full sentences and the auditory sandwich method): Correct. Strategies like the auditory sandwich (presenting information auditorily, supplementing if needed, then repeating it auditorily) help reinforce listening skills.

Thus, A is incorrect because AVA is not a multisensory approach but rather an auditory-only training method.

Question 19: Correct Statement About Principles of Formal Auditory Training

Answer: C - Only ①②④⑤

Explanation:

Formal auditory training aims to improve listening skills through structured exercises. Let's analyze each statement:

① Training with different speakers helps with generalization: Correct. Exposure to various voices, accents, and speech patterns enhances the ability to recognize speech in different situations.

② Non-speech training materials are only used for prelingually deafened young children: Correct. Early-stage auditory training for prelingually deaf children may include non-speech stimuli (e.g., environmental sounds) to develop basic auditory awareness before moving on to speech recognition.

③ A large number of training items should be presented in a short time: Incorrect. Overloading with too many stimuli can cause cognitive fatigue and reduce learning efficiency. Training should be structured and paced appropriately.

④ Auditory training activities should not mix analytical and synthetic stimuli: Incorrect. Effective auditory training can incorporate both analytic (focusing on phonemes or syllables) and synthetic (focusing on sentence meaning) approaches together for a well-rounded experience.

⑤ Practice should be at least 50 minutes daily, preferably at the same time: Correct. Consistency in training helps reinforce learning and integrate auditory skills into daily routines.

Thus, only ①, ②, ④, and ⑤ are correct, making option C the best choice.

Question 20: Auditory Response Level in the Hide-and-Seek Game

Answer: A - Detection (察覺)

Explanation:

In auditory skill development, detection (察覺) is the most basic level, referring to the ability to perceive the presence of sound. Let's analyze each option:

A (Detection/察覺): Correct. In the hide-and-seek game, the child is simply responding to the presence of a sound by hiding, indicating they have detected the auditory stimulus.

B (Discrimination/分辨): Incorrect. Discrimination involves distinguishing between different sounds (e.g., differentiating between "ba" and "da"), but the activity does not require this skill.

C (Recognition/辨識): Incorrect. Recognition requires associating a specific sound with its meaning (e.g., identifying a spoken word), which is beyond the task in the game.

D (Comprehension/理解): Incorrect. Comprehension requires understanding spoken language in context, but the child in this case is only responding to a sound cue rather than understanding speech.

Since the child's response is based solely on detecting sound rather than identifying or understanding it, A (Detection/察覺) is the most appropriate answer.

Question 21: Factor That Does NOT Affect Lexical Activation in the Neighborhood Activation Model (NAM) in Audiovisual Integration

Answer: C - Part of Speech (詞性)

Explanation:

The Neighborhood Activation Model (NAM) by Luce & Pisoni (1998) describes how spoken word recognition is influenced by the phonetic similarity of words. When applied to audiovisual integration, both auditory and visual cues contribute to activating potential word candidates. Let's analyze each factor:

A (Visual similarity/視覺上的相似度): Correct factor. Words that look similar when spoken (e.g., "pat" and "bat" having similar lip movements) can create lexical competition.

B (Auditory similarity/聽覺上的相似度): Correct factor. Words with similar phonetic structures (e.g., "cat" and "bat") are activated simultaneously, competing for recognition.

C (Part of speech/詞性): Incorrect factor. NAM is primarily concerned with phonetic and lexical properties, not grammatical categories like noun vs. verb. While part of speech may affect sentence-level comprehension, it does not directly influence lexical activation.

D (Word frequency/詞頻): Correct factor. High-frequency words are recognized faster and more easily activated than low-frequency words, impacting word competition.

Since part of speech (詞性) is not a core factor in lexical activation under NAM, C is the correct answer.

Question 22: Order of Analytic Lipreading Training from Easiest to Hardest

Answer: B - ①③②

Explanation:

Analytic lipreading training focuses on recognizing and differentiating phonemes based on visual characteristics of speech sounds (visemes). When ranking the difficulty level of distinguishing consonants, consider visual contrast, place of articulation, and voicing:

① (呆 vs. 塞): Easiest

These sounds have distinct places of articulation ("呆" /d/ is an alveolar stop, while "塞" /s/ is a fricative).

The difference between a stop and a fricative is visually noticeable.

③ (掰 vs. 拍): Moderate difficulty

Both are bilabial stops (/b/ vs. /p/).

The difference is voicing (/b/ is voiced, /p/ is voiceless), which is less visually distinct than the difference between a stop and a fricative.

② (掰 vs. 該): Most difficult

These have different places of articulation (bilabial /b/ vs. velar /g/).

Velar stops like /g/ are harder to see on the lips since they occur inside the mouth, making them more challenging to differentiate than bilabial contrasts.

Thus, the correct order from easiest to hardest is ① (呆 vs. 塞) → ③ (掰 vs. 拍) → ② (掰 vs. 該), which corresponds to option B.

Question 23: Group Comparison with Significant Lipreading Ability Differences

Answer: B - "Adult Males" vs. "Adult Females"

Explanation:

Lipreading ability varies due to cognitive, linguistic, and environmental factors, but some differences are more significant than others. Let's analyze each group:

A ("Middle-aged individuals with a high school education" vs. "Middle-aged individuals with a college education")

Not a significant factor in lipreading. Education may influence linguistic knowledge, but studies do not show strong differences in lipreading ability based solely on education level.

B ("Adult Males" vs. "Adult Females")

Significant difference.

Research indicates that females generally perform better in lipreading than males. This is attributed to greater use of facial cues, better phonological processing, and possibly greater exposure to communication-based tasks.

Correct answer.

C ("Young city-dwellers" vs. "Elderly city-dwellers")

Aging affects hearing, processing speed, and visual perception, but research does not show a consistent, significant decline in lipreading ability due to age alone. Some older adults may even compensate with experience.

D ("College students with average IQ" vs. "College students with high IQ")

IQ correlates with cognitive flexibility and language skills, but lipreading is more dependent on visual perception and phonological awareness than general intelligence. The difference is not large enough to be significant.

Thus, the group showing the most significant difference in lipreading ability is B ("Adult Males" vs. "Adult Females").

Question 24: Individual Adjustment Counseling Model Used by the Audiologist

Answer: A - Affective Model (情意模式)

Explanation:

In audiologic counseling, different models are used to help individuals adjust to hearing loss and hearing aids. Let’s analyze each option based on the scenario:

A (Affective Model/情意模式): Correct. This model focuses on addressing emotional responses and helping individuals process their feelings about hearing loss. In the given scenario, the audiologist allows the patient to express emotions through word selection and then discusses those feelings to reduce negative emotional barriers.

B (Informational Counseling Model/訊息諮商模式): Incorrect. This model is primarily educational, focusing on providing facts about hearing loss, assistive devices, and rehabilitation options. However, the scenario describes emotional exploration, not just information sharing.

C (Cognitive Model/認知模式): Incorrect. The cognitive model focuses on changing thoughts and beliefs about hearing loss (e.g., correcting misconceptions about hearing aids). While this may indirectly help with emotional adjustment, the audiologist in this scenario is focusing on emotional exploration rather than cognitive restructuring.

D (Psychosocial Model/社會心理模式): Incorrect. The psychosocial model considers social and interpersonal factors affecting hearing loss but is broader than what is described in the scenario. The audiologist is directly working with the individual's emotional response, making the affective model the best fit.

Thus, A (Affective Model/情意模式) is the most appropriate answer.

Question 25: Identifying a Non-Specific Repair Strategy

Answer: C - "What did you say?" (你說什麼？)

Explanation:

Repair strategies are techniques used by individuals with hearing loss to clarify misunderstandings in conversation. There are two types:

Specific Repair Strategies:

Provide clear guidance to the speaker on how to repair the breakdown.

Examples:

A (Repeating part of the message): "I heard ‘come at 3,’ but what was before that?" (Specific request)

B ("Can you rephrase that?"): The speaker is given a specific way to adjust communication. (Specific request)

D ("Are you saying you’ll come find me later?"): The listener is confirming information to clarify misunderstandings. (Specific request)

Non-Specific Repair Strategies:

Do not give the speaker useful feedback on what was missed.

Example:

C ("What did you say?"): This does not indicate what part of the message was unclear, making it a non-specific repair strategy.

Thus, the correct answer is C ("What did you say?" 你說什麼？), as it does not provide enough information for effective communication repair.

Question 26: Which Method Cannot Reduce Classroom Noise and Reverberation?

Answer: C - Raising the Ceiling (加高天花板)

Explanation:

Classroom acoustics significantly impact speech perception, especially for children with hearing difficulties. Let's evaluate each option:

A (Installing Carpet on the Floor): Correct. Carpets help absorb sound and reduce noise from footsteps and moving furniture, minimizing background noise.

B (Adding Plastic Pads to Desk Legs): Correct. Plastic pads reduce impact noise from moving desks and chairs, preventing unnecessary sound distractions.

C (Raising the Ceiling): Incorrect. A higher ceiling increases the space for sound waves to travel, leading to greater reverberation (echo). This makes speech harder to understand, worsening the classroom's acoustic environment. This does not reduce noise; it can make it worse.

D (Placing Bookshelves or Partitions in the Classroom): Correct. These structures help break up sound waves and reduce echoes, improving speech clarity.

Thus, C (Raising the Ceiling) is the correct answer because it does not reduce noise and may actually increase reverberation.

Question 27: Most Significant Factor Affecting Xinyi's Ability to Hear the Speaker

Answer: C - Distance Too Far, Speaker's Volume Decreases (距離太遠，說話者音量變小)

Explanation:

Xinyi can hear well in the classroom and communicate normally with peers, but struggles to hear the speaker over 40 meters away at the assembly. Let's analyze the possible causes:

A (Reverberation Blurring Speech): Unlikely. While reverberation can be an issue in enclosed spaces, outdoor environments typically do not cause significant echoes.

B (Excessive Noise Masking the Speaker's Voice): Unlikely. The scenario specifies that it was midday in a rural area, meaning background noise was not extreme. If noise were the main issue, Xinyi would struggle to hear classmates as well.

C (Distance Reducing Speaker Volume): Correct.

The inverse square law states that as distance doubles, sound intensity decreases significantly.

A speaker's voice weakens at greater distances, and Xinyi's hearing aids may not effectively compensate for this drop without additional support (e.g., FM systems).

D (Hearing Aid Insufficient Gain): Unlikely. Xinyi hears well in normal classroom settings, indicating her hearing aid is properly fitted. The issue arises due to the distance, not the hearing aid itself.

Thus, C (Distance Too Far, Speaker's Volume Decreases) is the best answer.

Question 28:

The most appropriate answer is B (Only ①②③).

Explanation:

① (Guiding the listener to check message coherence to ensure understanding) → This is a key strategy in dialogue-based therapy, as it helps the listener verify comprehension.

② (Guiding the speaker to communicate in a way that is easy to understand) → Clear and effective speech production is crucial for successful communication.

③ (Using linguistic rules and prior knowledge to interpret messages) → This reflects the cognitive aspect of dialogue-based therapy, where listeners use contextual and linguistic cues to derive meaning.

④ (Improving acoustic and optical conditions in the environment to enhance signals and reduce interference) → While optimizing the listening environment is beneficial, it falls more under environmental modifications rather than core strategies of dialogue-based therapy.

Since ④ focuses on external environmental factors rather than cognitive and interactive strategies essential to dialogue-based therapy, the correct answer is B (Only ①②③).

Question 29:

The correct answer is B (Developing individualized transition plans for students with hearing loss).

Explanation:

A (Evaluating and adjusting classroom acoustics for students with hearing loss) → Audiologists are responsible for assessing and improving classroom acoustics to create optimal listening conditions.

C (Regular electroacoustic analysis of hearing aids and FM systems for students with hearing loss) → Audiologists routinely conduct electroacoustic checks to ensure hearing devices are functioning properly.

D (Training classroom teachers to monitor and check auditory assistive devices for students) → Audiologists provide training for teachers to help them monitor and maintain hearing assistive technology.

B (Developing individualized transition plans for students with hearing loss) → Transition planning (e.g., preparing students for higher education or employment) is typically the responsibility of special education professionals, rehabilitation counselors, or transition coordinators, rather than audiologists.

Thus, the correct answer is B, as it falls outside the primary scope of an audiologist's role in managing hearing care within schools.

Question 30:

The correct answer is B (Listening in Spatialized Noise-Sentences Test, LISN-S).

Explanation:

The Multiple Auditory Processing Assessment (MAPA) is designed to evaluate the three key areas of Central Auditory Processing Disorder (CAPD) as outlined by ASHA (American Speech-Language-Hearing Association):

Monaural low-redundancy speech processing

Dichotic listening

Temporal processing

Let's analyze each answer choice:

A (Monaural Selective Auditory Attention Test, MSAAT) → This test assesses monaural low-redundancy speech processing, which is part of MAPA.

B (Listening in Spatialized Noise-Sentences Test, LISN-S) → This test evaluates spatial hearing abilities, particularly the ability to separate speech from noise based on spatial cues. It is not part of the standard MAPA framework.

C (Dichotic Digits Test) → This test measures dichotic listening skills, which are included in MAPA.

D (Pitch Pattern Test) → This assesses temporal pattern recognition, an essential component of CAPD evaluation and part of MAPA.

Since LISN-S primarily focuses on spatial auditory processing rather than the three key CAPD domains assessed in MAPA, the correct answer is B.

Question 31:

The correct answer is B (②③④).

Explanation:

Central Auditory Processing Disorder (CAPD) is characterized by difficulties in processing auditory information in the central nervous system. Let’s examine each statement:

① (Difficulty perceiving sounds) → Incorrect. CAPD is not typically associated with hearing sensitivity issues but rather with the brain's ability to process sounds correctly. Patients with CAPD usually have normal peripheral hearing.

② (Difficulty with sound localization) → Correct. CAPD can impact the ability to determine the direction from which a sound originates, as spatial auditory processing is often impaired.

③ (Diagnosed with CAPD in second grade) → Correct. CAPD is often diagnosed in school-aged children, typically around 7-8 years old, when auditory processing deficits become apparent in academic and social settings.

④ (Difficulty following instructions) → Correct. Individuals with CAPD often struggle with following verbal instructions, especially in noisy environments, due to deficits in auditory discrimination, memory, and sequencing.

Since ① is incorrect, but ②, ③, and ④ are correct, the best answer is B (②③④).

Question 32:

The correct answer is A (Low brainstem).

Explanation:

The Masking Level Difference (MLD) test assesses the ability of the auditory system to detect signals in noise when the phase of the signal or masker is altered. It primarily evaluates binaural interaction, which occurs in the low brainstem, specifically in structures such as the superior olivary complex (SOC) and cochlear nucleus.

A (Low brainstem) → Correct. The SOC, located in the low brainstem, plays a crucial role in binaural hearing and MLD. A lesion in this area often results in a reduced or absent MLD.

B (High brainstem) → The high brainstem (e.g., inferior colliculus) is involved in more complex auditory processing but is not the primary site for MLD sensitivity.

C (Auditory cortex) → The auditory cortex is involved in higher-level auditory processing but does not directly impact MLD, which is a brainstem-level phenomenon.

D (Corpus callosum) → The corpus callosum facilitates interhemispheric communication but is not directly involved in MLD.

Thus, MLD is most sensitive to lesions in the low brainstem, making A the correct answer.

Question 33:

The correct answer is B (Maximizing signal-to-noise ratio (SNR) is the most effective approach).

Explanation:

In managing Central Auditory Processing Disorder (CAPD) in educational settings, interventions aim to improve auditory signal clarity and reduce background noise. Let’s analyze each option:

A (Choosing a seat closest to the speaker is the most useful strategy) → Incorrect. While seating arrangement can help, it is not the most effective method. Children with CAPD often struggle with auditory processing even at close distances, especially in noisy environments.

B (Maximizing signal-to-noise ratio (SNR) is the most effective approach) → Correct. Improving SNR through FM/Remote Microphone (RM) systems, preferential seating, and classroom acoustic modifications is one of the best ways to enhance auditory input for children with CAPD.

C (Using an RM system can solve timing processing issues) → Incorrect. While RM systems improve SNR, they do not directly address temporal processing deficits that affect CAPD patients. Specific auditory training programs are needed for temporal processing issues.

D (All CAPD patients benefit from auditory training) → Incorrect. While auditory training can be helpful, its effectiveness varies depending on the specific auditory deficits of the individual. Not all CAPD patients respond equally to training.

Since improving SNR directly enhances speech clarity and learning outcomes for CAPD children, the best answer is B.

Question 34:

The correct answer is B (Because the loud noise they hear is tinnitus).

Explanation:

Older adults with sensorineural hearing loss (SNHL) often struggle with speech clarity, even when sounds are loud enough. Let’s analyze each option:

A (Because it is sensorineural hearing loss) → Likely. SNHL often results in distortion, making speech sound unclear even at sufficient volume. This is due to damage in the cochlea, particularly to inner hair cells.

B (Because the loud noise they hear is tinnitus) → Unlikely. While tinnitus is common in hearing loss, it typically manifests as a persistent ringing or buzzing sound, rather than causing speech to sound "jumbled" or "distorted." The issue described in the question is more related to auditory processing deficits rather than tinnitus.

C (The dynamic range of hearing has decreased) → Likely. A reduced dynamic range (loudness recruitment) makes it harder to tolerate loud sounds, which can make speech perception difficult.

D (This phenomenon is phonemic regression) → Likely. Phonemic regression refers to a disproportionate difficulty in speech understanding compared to the degree of hearing loss, often seen in older adults with central auditory processing decline.

Since tinnitus does not directly cause speech to sound "jumbled" or unclear, the least likely reason is B.

Question 35:

The correct answer is B (The effectiveness of tinnitus treatment only needs to be verified by well-designed clinical studies, and the results do not need to be reproducible).

Explanation:

For any medical treatment, including tinnitus management, effectiveness must be verified through well-designed clinical studies AND be reproducible to be considered valid. Let's evaluate each statement:

A (The effectiveness of tinnitus treatment should be determined by well-designed clinical studies) → Correct. Scientific validation through controlled trials is essential for assessing treatment efficacy.

B (The effectiveness of tinnitus treatment only needs to be verified by well-designed clinical studies, and the results do not need to be reproducible) → Incorrect. Reproducibility is a core principle of scientific research. A single study is not sufficient to confirm effectiveness—results must be consistently replicated across multiple studies.

C (There is no systematic evidence proving that herbal supplements, such as ginkgo, are effective for tinnitus) → Correct. Research has not shown consistent, significant benefits of ginkgo for tinnitus.

D (There is no systematic evidence proving that dietary supplements, such as vitamin B12, are effective for tinnitus) → Correct. No strong clinical evidence supports the use of dietary supplements like vitamin B12 for tinnitus treatment.

Since scientific research requires results to be reproducible, the incorrect statement is B.

Question 36:

The correct answer is D (Unilateral vestibular dysfunction causes greater dynamic visual acuity decline than bilateral dysfunction due to nystagmus).

Explanation:

Dynamic Visual Acuity (DVA) testing assesses the ability to maintain visual clarity during head movements and is commonly used to evaluate vestibulo-ocular reflex (VOR) function. Let's analyze each statement:

A (DVA evaluates vestibular function by stabilizing foveal vision via the VOR mechanism) → Correct. The VOR is responsible for maintaining stable vision during head movements by adjusting eye position accordingly.

B (Patients shake their heads actively or passively at 2 Hz (120–180 deg/sec) while taking a vision test) → Correct. This is the standard protocol for DVA testing.

C (A positive result is defined as a loss of three or more lines on the visual acuity chart compared to static vision) → Correct. A decline of three or more lines is considered clinically significant.

D (Unilateral vestibular dysfunction causes more severe DVA decline than bilateral dysfunction due to nystagmus) → Incorrect. Bilateral vestibular dysfunction (BVD) typically causes greater impairment in dynamic visual acuity because patients lack VOR function in both ears, severely compromising visual stability. Unilateral vestibular dysfunction (UVD) does cause DVA decline, but not necessarily worse than BVD, and nystagmus is not the primary cause of DVA reduction.

Since bilateral vestibular dysfunction generally leads to more severe DVA impairment, the incorrect statement is D.

Question 37:

The correct answer is B (The efferent output of cVEMP is via the 11th cranial nerve, the accessory nerve).

Explanation:

Cervical Vestibular Evoked Myogenic Potential (cVEMP) is a test that assesses saccular and inferior vestibular nerve function by measuring inhibitory responses in the sternocleidomastoid (SCM) muscle. Let's analyze each option:

A (cVEMP is an electromyographic response elicited by sound, taps, or electrical stimulation of the saccule) → Correct. The saccule is the primary receptor for cVEMP, which can be stimulated by air-conducted sound, bone-conducted vibration, or electrical stimuli.

B (The efferent output is via the 11th cranial nerve, the accessory nerve) → Incorrect. The correct efferent pathway is through the vestibular nuclei to the ipsilateral spinal accessory nucleus via the vestibulospinal tract, but the primary efferent nerve involved is the facial nerve (cranial nerve VII) and the vestibulospinal pathway, not the accessory nerve (cranial nerve XI).

C (The most commonly recorded muscle for cVEMP is the contralateral sternocleidomastoid (SCM) muscle) → Correct. cVEMP responses are typically recorded from the SCM muscle because it provides strong inhibitory responses to vestibular stimulation.

D (cVEMP is an inhibitory postsynaptic potential response) → Correct. cVEMP is a short-latency inhibitory response, meaning it suppresses muscle activity in the SCM upon vestibular stimulation.

Since cVEMP does not primarily involve the accessory nerve (cranial nerve XI) as its main efferent output, the incorrect statement is B.

Question 38:

The correct answer is B (B).

Explanation:

When the body's center of gravity moves beyond the limit of stability, the body employs postural control strategies to regain balance. The three primary strategies are:

Ankle Strategy – Used for small disturbances; involves subtle movements at the ankle joint.

Hip Strategy – Used for moderate disturbances; involves bending at the hips to shift the center of mass.

Stepping Strategy – Used for large disturbances; involves taking a step to prevent falling.

Analysis of the Image Options:

A (Ankle Strategy): Small forward-backward sway controlled by ankle movements.

B (Hip Strategy): Significant hip movement is used to counteract a larger shift in balance, especially when standing on a narrow or unstable surface.

C (Stepping Strategy): A step is taken to regain balance when stability is significantly compromised.

D (Normal Walking Posture): Represents typical gait rather than a balance recovery strategy.

Since the question refers to a condition where the center of gravity moves beyond the limit of stability, the hip strategy (B) is the most appropriate reflexive postural control response before resorting to stepping (C).

Question 39:

The correct answer is C (The recommended amount of ice water injected is 2 c.c.).

Explanation:

The cold caloric test (ice-water caloric test) is a diagnostic tool used to evaluate vestibular function, particularly in patients with suspected peripheral vestibular dysfunction or brainstem involvement.

A (Used to test the patient's residual vestibular function) → Correct. This test assesses whether the vestibular system still has functional responsiveness.

B (Should not be performed in patients with tympanic membrane perforation) → Correct. If the eardrum is perforated, water can enter the middle ear and potentially cause infection or other complications.

C (Recommended ice water volume is 2 c.c.) → Incorrect. The standard amount used in caloric testing is typically about 1 mL (1 c.c.) of ice water per ear. Larger volumes are not recommended because they do not improve accuracy and may cause discomfort.

D (Applicable for bilateral bithermal caloric testing, where the sum of four responses is less than 8 deg/s) → Correct. A total response of less than 8 deg/s across both ears indicates bilateral vestibular hypofunction (BVH).

Since 1 c.c. is the standard recommended volume, the incorrect statement is C.

Question 40:

The correct answer is B (Both tests are related to the vestibulo-ocular reflex, VOR).

Explanation:

Both Head-Shaking Nystagmus (HSN) Test and Video Head Impulse Test (vHIT) assess vestibular function, specifically the vestibulo-ocular reflex (VOR), which stabilizes vision during head movements.

A (Both tests are highly specific for detecting unilateral vestibular dysfunction) → Incorrect. While both tests are useful, vHIT has higher specificity for detecting unilateral vestibular hypofunction. HSN is less specific and can be influenced by central vestibular mechanisms.

B (Both tests are related to the vestibulo-ocular reflex, VOR) → Correct. Both tests assess how the VOR compensates for head movements and whether there is a vestibular deficit.

C (Both tests observe horizontal nystagmus) → Incorrect. vHIT measures catch-up saccades rather than just horizontal nystagmus. HSN primarily detects post-head-shaking nystagmus in cases of vestibular asymmetry.

D (Both tests are based on the central velocity storage mechanism) → Incorrect. The central velocity storage mechanism is mainly involved in prolonging rotational responses in the VOR, but vHIT primarily assesses the direct vestibular pathways rather than central compensation mechanisms.

Since both tests rely on the vestibulo-ocular reflex (VOR), the correct statement is B.

Question 41:

The correct answer is C (Hair cells are located in the crista, and their cilia extend into the cupula, which shifts to generate excitatory or inhibitory responses).

Explanation:

The semicircular canals detect angular acceleration and contain the ampulla, which houses the crista ampullaris (a sensory organ for detecting head movement).

A (Ampulla contains the crista and cupula, and the cupula contains otoconia) → Incorrect. The cupula does not contain otoconia. Otoconia (calcium carbonate crystals) are found in the utricle and saccule, not in the semicircular canals.

B (Otoliths are lighter than the cupula and endolymph, and they shift when endolymph flows) → Incorrect. Otoliths (ear stones) are denser than endolymph, allowing them to detect linear acceleration and gravity in the utricle and saccule, not in the semicircular canals.

C (Hair cells are in the crista, and their stereocilia extend into the cupula, which shifts and generates an excitatory or inhibitory response) → Correct. The cupula moves in response to endolymph flow, causing deflection of stereocilia on hair cells within the crista ampullaris, leading to excitation or inhibition.

D (When stereocilia bend toward kinocilia, hyperpolarization occurs, leading to excitation) → Incorrect. When stereocilia bend toward kinocilia, depolarization occurs, increasing neural firing and leading to excitation. Hyperpolarization occurs when the movement is in the opposite direction.

Thus, the correct answer is C.

Question 42:

The correct answer is C (The medial rectus is controlled by cranial nerve III, and the lateral rectus is controlled by cranial nerve VI).

Explanation:

The extraocular muscles are controlled by different cranial nerves:

Medial rectus (moves the eye inward) → Controlled by Cranial Nerve III (Oculomotor nerve)

Lateral rectus (moves the eye outward) → Controlled by Cranial Nerve VI (Abducens nerve)

Now, analyzing the choices:

A (CN II and CN III) → Incorrect. CN II (Optic nerve) is responsible for vision, not eye movement.

B (CN IV and CN V) → Incorrect. CN IV (Trochlear nerve) controls the superior oblique, and CN V (Trigeminal nerve) controls facial sensation and chewing muscles, not extraocular muscles.

C (CN III and CN VI) → Correct. CN III controls the medial rectus, and CN VI controls the lateral rectus.

D (CN IV and CN VI) → Incorrect. CN IV controls the superior oblique, not the medial rectus.

Thus, the correct answer is C.

Question 43:

The correct answer is B (The presence of horizontal nystagmus must indicate peripheral vestibular dysfunction).

Explanation:

Head-shaking nystagmus (HSN) is a test used to evaluate vestibular function by shaking the head and observing induced eye movements. It helps in detecting vestibular asymmetry.

A (Normal individuals rarely exhibit this type of nystagmus) → Correct. In healthy individuals, HSN is generally absent or very minimal because both vestibular systems function symmetrically.

B (The presence of horizontal nystagmus must indicate peripheral vestibular dysfunction) → Incorrect. HSN can be seen in both peripheral and central vestibular disorders. While unilateral peripheral vestibular lesions often cause HSN, central disorders (e.g., cerebellar dysfunction) can also result in abnormal HSN patterns.

C (Patients with cerebellar dysfunction may also exhibit nystagmus) → Correct. Cerebellar disorders can cause spontaneous or gaze-evoked nystagmus, and HSN may also be triggered.

D (A second-phase nystagmus may appear—after initial nystagmus, a short pause occurs, followed by nystagmus in the opposite direction) → Correct. This biphasic pattern is observed in some cases of vestibular imbalance.

Thus, the incorrect statement is B.

Question 44:

The correct answer is C (Nystagmus velocity).

Explanation:

The caloric test assesses unilateral vestibular weakness by stimulating the horizontal semicircular canal using warm or cold water/air. The most critical parameter in determining unilateral weakness (UW) is nystagmus velocity.

A (Nystagmus duration) → Incorrect. Duration is not a standard measure for UW.

B (Nystagmus amplitude) → Incorrect. While amplitude provides some insight, it is not used for UW calculation.

C (Nystagmus velocity) → Correct. The peak slow-phase velocity (SPV) is the primary parameter for calculating unilateral weakness. It reflects the strength of vestibular response.

D (Nystagmus latency) → Incorrect. Latency refers to the time from stimulus onset to nystagmus onset, but it is not used for calculating UW.

Thus, the correct answer is C.

Question 45

Which vestibular function test can evaluate only one side of the vestibular system at a time without being influenced by the other side?

Answer: B. 温差测试 (Caloric Test)

Explanation:

The caloric test is a specific vestibular function test that selectively stimulates one vestibular organ at a time. This is done by introducing warm or cold air or water into one ear canal, inducing endolymph flow changes in the lateral semicircular canal. This results in a measurable nystagmus response that is independent of the function of the opposite ear.

A. Head-shaking nystagmus test: This test assesses overall vestibular function by inducing head movements, but it does not isolate one side completely.

C. Posturography: This evaluates balance and postural control but does not isolate unilateral vestibular function.

D. Rotational chair test: This stimulates both vestibular organs simultaneously and does not allow for unilateral assessment.

Question 46

Which of the following statements about the vestibular system and postural control is incorrect?

Answer: A. 在老年人族群，測得頸前庭誘發肌電位（cVEMP）的振幅較成年人族群為小，然而眼前庭誘發肌電位（oVEMP）檢查的部分，老年人族群則沒有振幅減小的現象。

Explanation:

This statement is incorrect because both cVEMP and oVEMP amplitudes typically decrease with age.

cVEMP evaluates the saccule and inferior vestibular nerve, and its response is known to diminish with aging due to reduced vestibular function and muscle degeneration.

oVEMP evaluates the utricle and superior vestibular nerve, and while some studies suggest it may be more resilient to age-related decline, most evidence indicates that oVEMP amplitudes also reduce with aging.

Other statements are correct:

B. Older adults rely more on visual input to maintain balance due to vestibular and somatosensory decline.

C. Lower limb strength is crucial for postural stability and balance control.

D. Somatosensory feedback from the feet plays an essential role in postural maintenance.

Question 47

Which of the following statements about the pathophysiology of left vestibular neuritis is incorrect?

Answer: B. The spontaneous nystagmus and vertigo gradually subside within a few days because the function of the damaged left vestibular system gradually recovers.

Explanation:

Vestibular neuritis is caused by inflammation of the vestibular nerve, leading to an acute unilateral vestibular dysfunction.

A. Correct – Left vestibular neuritis results in an imbalance in vestibular input, causing severe vertigo, nausea, and spontaneous nystagmus beating to the right (toward the unaffected side).

B. Incorrect – The reduction of symptoms over time is primarily due to central vestibular compensation, not the actual recovery of the left vestibular function. Many patients continue to have residual vestibular deficits, even though symptoms improve.

C. Correct – The right vestibular nucleus increases its resting activity because the inhibitory input from the left vestibular nucleus is reduced. This imbalance contributes to nystagmus and vertigo.

D. Correct – Vestibular adaptation involves central compensation mechanisms within the vestibular nuclei and cerebellum, allowing the brain to adjust to the unilateral loss of function over time.

Question 48

A 54-year-old male developed acute vertigo after a cold, with spontaneous right-beating nystagmus and normal pure-tone audiometry. His vertigo resolved within a week after anti-vertigo medication. What is the most likely diagnosis?

Answer: C. Suspected left vestibular neuritis; the absence of vertigo does not necessarily mean vestibular function has fully recovered.

Explanation:

The patient's spontaneous right-beating nystagmus suggests left vestibular hypofunction, indicating possible left vestibular neuritis.

A. Incorrect – The absence of vertigo does not necessarily mean vestibular function has fully recovered. Central compensation can reduce symptoms, even if the left vestibular system remains impaired.

B. Incorrect – Right-beating nystagmus suggests a left-sided lesion, not a right-sided one.

C. Correct – The left vestibular system is likely affected, but the disappearance of vertigo does not mean full recovery of vestibular function. Residual vestibular deficits often persist, detectable through vestibular function tests (e.g., caloric test, head impulse test).

D. Incorrect – The right vestibular system is unlikely to be affected because the observed nystagmus beats toward the right, indicating left-side dysfunction.

Final Takeaway:

Vestibular neuritis typically results in prolonged vestibular dysfunction, even after symptoms resolve. Compensation, rather than full recovery of vestibular function, is often the reason for symptom improvement.

Question 49

Which of the following is NOT a typical symptom of superior semicircular canal dehiscence (SSCD)?

Answer: B. 跌落性發作 (Drop attack)

Explanation:

Superior semicircular canal dehiscence (SSCD) is caused by a thinning or absence of the bony covering of the superior semicircular canal, leading to abnormal inner ear pressure transmission and symptoms related to sound- and pressure-induced vestibular disturbances.

A. Paracusis Willisii (Correct Symptom) – This refers to the paradoxical improvement in hearing in noisy environments, which is not characteristic of SSCD. However, patients with SSCD often report autophony (hearing their own voice abnormally loud) and sound hypersensitivity.

B. Drop Attack (Incorrect Symptom) – Drop attacks are NOT a typical symptom of SSCD. They are more commonly associated with Ménière’s disease (Tumarkin’s crisis) or vestibular disorders with sudden balance loss.

C. Tullio Phenomenon (Correct Symptom) – This is sound-induced vertigo or dizziness, which is a hallmark of SSCD. It occurs because the dehiscence creates a "third window" in the inner ear, leading to abnormal fluid displacement.

D. Hennebert’s Sign (Correct Symptom) – This refers to pressure-induced vertigo or nystagmus, another common feature of SSCD due to its third-window effect.

Question 50

Which of the following correctly describes the pathophysiology of benign paroxysmal positional vertigo (BPPV)?

Answer: B. ② 脱落的耳石于半规管中 (canalithiasis) + ③ 脱落耳石附着于壶腹顶帽 (cupulolithiasis)

Explanation:

BPPV is caused by displaced otoconia (calcium carbonate crystals) from the utricle that enter the semicircular canals, leading to inappropriate endolymph movement when the head changes position.

① (Incorrect) Otoconia detachment occurs from the utricle, not directly from the ampulla.

② (Correct) Canalithiasis – This is the most common mechanism of BPPV, where free-floating otoconia move within the semicircular canal and displace endolymph, causing brief vertigo episodes.

③ (Correct) Cupulolithiasis – In this less common form, otoconia attach to the cupula of the semicircular canal, making it more sensitive to gravitational changes, leading to prolonged vertigo instead of brief episodes.

④ (Incorrect) Otoconia are denser than endolymph, allowing them to settle and cause symptoms. If they had the same density, they wouldn't affect the vestibular system significantly.

Thus, option B (②③) is the correct answer as both canalithiasis and cupulolithiasis are recognized mechanisms of BPPV.

6106

Question 1

Which of the following statements about dysphagia is the most appropriate?

Answer: C. 多數的小兒吞嚥困難源自特定的醫學診斷 (Most pediatric swallowing difficulties stem from specific medical diagnoses).

Explanation:

Dysphagia refers to difficulty swallowing and can affect different phases of the swallowing process (oral, pharyngeal, esophageal). It can occur due to neurological conditions, structural abnormalities, or other medical disorders.

A. Incorrect – The prevalence of dysphagia increases with age, particularly among older adults due to conditions such as stroke, Parkinson's disease, or muscle degeneration.

B. Incorrect – Dysphagia does not involve difficulties moving food from the stomach to the intestines, as this is a gastrointestinal issue rather than a swallowing disorder.

C. Correct – In children, swallowing disorders are often associated with medical conditions such as cerebral palsy, premature birth, neuromuscular disorders, or congenital abnormalities.

D. Incorrect – Frequent choking when swallowing saliva can be a sign of dysphagia, particularly in neurological or neuromuscular disorders.

Question 2

Which communication disorder is more common in females than in males?

Answer: C. 选择性缄默症 (Selective Mutism).

Explanation:

Most communication disorders occur more frequently in males than females, but selective mutism is one of the few that is more common in females. This condition is an anxiety-related disorder in which a child consistently fails to speak in specific social settings despite having normal speech ability in other situations.

A. Incorrect – Speech sound disorders (e.g., articulation disorders) are more common in males than females.

B. Incorrect – Hearing loss occurs at similar rates in both genders, though males are slightly more prone to noise-induced hearing loss.

C. Correct – Selective mutism is seen more frequently in females, likely due to differences in anxiety-related behaviors.

D. Incorrect – Fluency disorders (e.g., stuttering) are more common in males than females, with a ratio of about 4:1.

Thus, option C (Selective Mutism) is the correct answer, as it is one of the few communication disorders with a higher prevalence in females.

Question 3

Based on the patient's speech characteristics, what type of aphasia is most likely?

Answer: D. 運動型皮質間失語症 (Transcortical Motor Aphasia).

Explanation:

The patient's speech suggests difficulty initiating speech and repeating words, which are key features of transcortical motor aphasia (TMA).

A. Incorrect – Conduction Aphasia: Patients with conduction aphasia typically have relatively fluent speech but severe difficulty with repetition, often due to damage in the arcuate fasciculus. The patient in the example does not show these characteristics.

B. Incorrect – Transcortical Sensory Aphasia: This condition is characterized by poor comprehension and fluent but nonsensical speech (similar to Wernicke's aphasia), but repetition remains intact. In this case, the patient struggles to repeat words, suggesting a motor impairment rather than sensory.

C. Incorrect – Subcortical Aphasia: Subcortical aphasia can vary in symptoms but generally includes mild language impairments with no distinct repetition deficits like those seen in the patient.

D. Correct – Transcortical Motor Aphasia: TMA patients struggle with spontaneous speech production and repetition but usually have relatively intact comprehension. The patient's inability to repeat "地方" (place) and difficulty initiating speech fits this diagnosis.

Question 4

Which statement about communication disorders is the most inappropriate?

Answer: A. 言語障礙 (Speech disorders) 包含語用 (pragmatics), 說話流暢性 (fluency), 或音質的異常.

Explanation:

Speech and language disorders are distinct categories, and pragmatics (social language use) is related to language, not speech disorders.

A. Incorrect (Most inappropriate statement): Pragmatic difficulties are part of language disorders (not speech disorders), as they involve using language appropriately in social contexts, rather than issues with articulation, fluency, or voice.

B. Correct: Language disorders affect oral, written, or symbol-based communication, impairing the ability to understand, produce, or use language correctly.

C. Correct: Dysarthria is a speech disorder caused by neuromuscular impairments, leading to weak, slow, or imprecise speech production.

D. Correct: Individuals with intellectual disabilities often struggle with abstract language, which falls under language disorders rather than speech disorders.

Thus, option A is the most inappropriate because pragmatic difficulties belong to language disorders, not speech disorders.

Question 5

In speech development, when a child pronounces "飛機" (fēi jī) as "胚機" (pēi jī), which phonological process is occurring?

Answer: B. 塞音化 (Stopping).

Explanation:

Phonological processes are patterns of sound changes that young children use as they develop their speech skills.

A. 同化 (Assimilation) – Incorrect: Assimilation occurs when a sound changes to become more like a nearby sound (e.g., "狗狗" → "都都"). However, in this case, the initial sound is being replaced rather than influenced by surrounding sounds.

B. 塞音化 (Stopping) – Correct: This occurs when a fricative (擦音) is replaced by a stop (塞音). In this case, "f" (a fricative) is replaced with "p" (a stop), making "飛機" (fēi jī) sound like "胚機" (pēi jī). This is a common phonological process in young children.

C. 擦音化 (Fricativization) – Incorrect: This would mean that a stop sound is replaced by a fricative, which is the opposite of what is happening here.

D. 前置化 (Fronting) – Incorrect: Fronting occurs when a sound produced in the back of the mouth (like "k" or "g") is replaced by a sound produced in the front (like "t" or "d"). This is not the case in this example.

Thus, the correct answer is B. 塞音化 (Stopping).

Question 6

Which statement about stuttering is the most inappropriate?

Answer: C. 學齡階段盛行率女生高於男生 (In school-age children, the prevalence of stuttering is higher in females than in males).

Explanation:

Stuttering is a speech disorder characterized by disruptions in fluency, such as repetitions, prolongations, and blocks.

A. Correct: Stuttering typically begins in early childhood, often between 2 to 5 years old, making early childhood the most common period for onset.

B. Correct: A significant percentage of children recover naturally before the age of 6, with estimates ranging from 75-80% of early childhood stuttering cases resolving without intervention.

C. Incorrect (Most inappropriate statement): Stuttering is more common in males than females, with a male-to-female ratio of approximately 4:1 in school-aged children. This statement incorrectly suggests that females are more affected.

D. Correct: Genetics play a role in stuttering, and research indicates that family history is a significant risk factor, suggesting a hereditary component.

Thus, option C is the most inappropriate, as stuttering is significantly more common in males than females, not the other way around.

Question 7: What is the most appropriate method for assessing the language abilities of bilingual children?

Answer: C. 宜適時轉換語言符碼，以達最佳評估結果 (It is appropriate to switch language codes when necessary to achieve the best assessment outcome).

Explanation: When assessing bilingual children, it is crucial to consider both languages and how the child uses them in different contexts. Here’s why the other options are incorrect:

A. Incorrect – "Non-standardized assessments should not be used to avoid affecting evaluation results."

While standardized tests can provide structured data, non-standardized assessments (such as dynamic assessment and language sampling) are often necessary to get a comprehensive picture of a bilingual child’s abilities. Relying only on standardized tests may not account for linguistic and cultural diversity.

B. Incorrect – "Different languages share common structural elements, so cultural differences can be ignored."

While languages share some universal features, each language has unique grammar, syntax, and cultural influences. Ignoring cultural differences can lead to misinterpretation of the child’s language abilities.

C. Correct – "Code-switching should be used appropriately to achieve the best assessment results."

Bilingual children naturally switch between languages depending on context, and assessing both languages helps in identifying strengths and weaknesses accurately. Code-switching during assessment allows a more natural evaluation of their communication skills, rather than restricting them to a single language.

D. Incorrect – "Using an assessment tool in only one language is sufficient."

Bilingual children may have uneven language proficiency across their two languages, meaning that testing in only one language may underestimate their true abilities. Best practices recommend evaluating both languages to gain a complete understanding of their linguistic skills.

Thus, option C is the best choice, as allowing bilingual children to switch languages during evaluation provides a more accurate and comprehensive assessment of their abilities.

Question 8

Which statement about dynamic assessment is correct?

Answer: B. 評量的程序通常為「介入－評量－再介入」 (The assessment procedure usually follows the sequence of "intervention - assessment - re-intervention").

Explanation:

Dynamic assessment (DA) is an approach that focuses on learning potential rather than static performance. It evaluates how a child responds to intervention and whether they can learn with support.

A. Incorrect – "Dynamic assessment is a one-way, structured assessment method."

Dynamic assessment is interactive and flexible, rather than rigid and one-way. It focuses on how a child learns rather than just measuring what they already know.

B. Correct – "The assessment procedure usually follows the sequence of intervention - assessment - re-intervention."

Dynamic assessment follows the "test-teach-retest" model, where a baseline is established, intervention is introduced, and then the child is reassessed to observe improvement.

C. Incorrect – "Not suitable for children from diverse cultural or bilingual backgrounds."

Dynamic assessment is particularly useful for culturally and linguistically diverse children because it evaluates their ability to learn rather than their prior knowledge, which may be influenced by language exposure.

D. Correct but incomplete – "It is a non-standardized assessment method."

While dynamic assessment is a non-standardized method, the key correct answer here is B, as it describes the fundamental assessment procedure.

Thus, option B is the best choice, as it accurately describes the core process of dynamic assessment.

Question 9

Which statement about specific language impairment (SLI) is the most appropriate?

Answer: C. 特定型語言障礙兒童的腦造影顯示，其左右腦呈對稱 (Brain imaging studies of children with SLI show symmetrical left and right hemispheres).

Explanation:

Specific language impairment (SLI), now often referred to as Developmental Language Disorder (DLD), is a condition where a child has difficulty acquiring language despite normal cognitive abilities and no obvious neurological damage.

A. Incorrect – "SLI occurs at similar rates in males and females."

Studies show that SLI is more common in males than females, with a male-to-female ratio of approximately 3:1.

B. Incorrect – "Family history does not affect the prevalence of SLI."

Genetic factors play a significant role in SLI, with studies indicating that children with a family history of SLI are at higher risk of developing the disorder.

C. Correct – "Brain imaging studies of children with SLI show symmetrical left and right hemispheres."

Neuroimaging research suggests that children with SLI often show atypical brain asymmetry, with less left-hemisphere dominance for language processing compared to typically developing children. This more symmetrical brain structure may contribute to their language difficulties.

D. Incorrect – "In addition to poor language performance, their nonverbal IQ is significantly lower than that of typical children."

SLI is specifically a language disorder, meaning that nonverbal IQ is usually within the normal range. If nonverbal IQ were significantly impaired, the diagnosis would likely be a broader cognitive impairment rather than SLI.

Thus, option C is the best answer, as it correctly describes one of the neurological characteristics of children with SLI.

Question 10

Which statement about Augmentative and Alternative Communication (AAC) is the most inappropriate?

Answer: A. 使用 AAC 會限制口語表達的發展 (Using AAC will limit the development of verbal expression).

Explanation:

AAC (Augmentative and Alternative Communication) refers to tools and strategies that help individuals with communication difficulties. The goal of AAC is not to replace speech but to support and enhance communication.

A. Incorrect – "Using AAC will limit the development of verbal expression."

Research shows that AAC does not hinder speech development; in fact, it may promote language growth by giving users an effective way to communicate, reducing frustration, and encouraging interaction.

Many individuals who use AAC eventually develop or improve verbal speech when provided with the right support.

B. Correct – "Unaided AAC includes gestures and sign language."

Unaided AAC refers to communication methods that do not require external tools, such as gestures, facial expressions, and sign language.

C. Correct – "Aided AAC includes the use of tablets or communication boards."

Aided AAC involves tools such as communication boards, speech-generating devices, and tablets with AAC apps.

D. Correct – "Using pen and paper for communication is also a form of AAC."

Low-tech AAC includes writing, drawing, or pointing to symbols on a board, all of which help facilitate communication.

Thus, option A is the most inappropriate statement, as AAC does not restrict speech development but rather supports and enhances communication abilities.

Question 11

Which statement about intervention for Auditory Neuropathy Spectrum Disorder (ANSD) is the most inappropriate?

Answer: D. Berlin 等人（2010）指出助聽器對多數患者有幫助，人工電子耳僅對少數患者有效 (Berlin et al. (2010) stated that hearing aids help most patients, while cochlear implants are only effective for a few patients).

Explanation: Auditory Neuropathy Spectrum Disorder (ANSD) is a condition where sound reaches the inner ear normally, but neural transmission to the brain is disrupted. Intervention varies based on the severity of the condition and individual patient needs.

A. Correct – "Sometimes, delayed diagnosis prevents timely treatment planning."

Early diagnosis is crucial for effective intervention. Delayed diagnosis may lead to missed opportunities for early intervention, which is critical for speech and language development.

B. Correct – "Even after diagnosis, there is often debate over the best treatment approach."

ANSD varies widely among patients, and treatment options—such as hearing aids, cochlear implants, and auditory training—do not work equally well for everyone. This leads to ongoing discussions among professionals regarding the most effective intervention.

C. Correct – "Audiologists often initially try hearing aids for these children."

In many cases, hearing aids are initially fitted to assess whether amplification helps. However, some patients with ANSD do not benefit from hearing aids due to poor neural synchrony.

D. Incorrect – "Berlin et al. (2010) stated that hearing aids help most patients, while cochlear implants are only effective for a few patients."

Research suggests that hearing aids are often ineffective for ANSD patients with severe neural dysfunction, as the problem lies in neural transmission rather than amplification. Cochlear implants (CIs) have been shown to significantly benefit many ANSD patients, as they provide a more synchronized neural signal to the brain, improving speech perception. Berlin et al. (2010) and other researchers have actually suggested that CIs are a preferred treatment option for many ANSD patients who do not benefit from hearing aids.

Thus, option D is the most inappropriate statement, as cochlear implants have been found to be effective for many ANSD patients, not just a small number.

Question 12: Regarding otitis media, which of the following statements is the most appropriate?

Analysis of Each Option:

A: It is true that preschool children are highly susceptible to acute otitis media (AOM), with studies indicating that 80%-90% of children experience at least one episode before the age of 3. However, the peak incidence typically occurs between 6 months and 2 years, rather than 4 to 5 years. Therefore, this option contains an inaccurate age range.

B: Otitis media with effusion (OME) is defined as middle ear fluid persisting for more than 3 months (12 weeks). Common symptoms include ear fullness, hearing loss, and tinnitus, but without acute inflammatory pain. This definition and description are accurate.

C: According to the World Health Organization (WHO), chronic suppurative otitis media (CSOM) is characterized by persistent ear discharge (otorrhea) lasting more than 2 weeks to 3 months, depending on the definition used. The option states more than 6 months, which is incorrect.

D: Acute otitis media can lead to cholesteatoma, which disrupts the ossicles and results in significant conductive hearing loss. However, cholesteatoma is more commonly associated with chronic otitis media rather than acute otitis media. Therefore, while partially correct, this is not the best answer.

✅ Best Answer: B

Question 13: Regarding noise-induced hearing loss, which of the following statements is the least appropriate?

Analysis of Each Option:

A: Different types of noise have varying effects on hearing. Continuous noise (e.g., industrial noise) is generally more harmful than intermittent noise, as the auditory system has less time to recover. This statement is correct.

B: Using both earmuffs and earplugs together provides better noise protection. However, earplugs contribute more to noise reduction than earmuffs, as they directly block sound from entering the ear canal. While not entirely incorrect, the statement overemphasizes the role of earmuffs.

C: Noise-induced hearing loss (NIHL) typically presents as bilateral and symmetrical sensorineural hearing loss, primarily affecting high frequencies, especially around 4000 Hz. This statement is correct.

D: Noise-induced hearing loss is irreversible, but its progression does not necessarily slow down as hearing thresholds increase. If noise exposure continues, hearing loss can worsen significantly. This statement is incorrect.

✅ Least Appropriate Answer: D

Question 14: Regarding Mr. Wang, a long-term sufferer of Meniere's disease, which of the following statements is the most inappropriate?

Analysis of Each Option:

A: Meniere's disease is characterized by sensorineural hearing loss (typically moderate), vertigo, nausea, and vomiting. These symptoms occur due to abnormal fluid buildup in the inner ear, affecting the cochlea and vestibular system. This statement is correct.

B: Meniere's disease often causes fluctuating low- to mid-frequency hearing loss, which can make fitting a hearing aid challenging. The fluctuations mean that hearing thresholds can vary over time, affecting amplification settings. This statement is correct.

C: The most widely accepted cause of Meniere's disease is endolymphatic hydrops, or an abnormal accumulation of endolymph in the inner ear. The saccule is one of the inner ear structures that can be affected. While the exact cause is not fully understood, this statement aligns with current medical hypotheses. This statement is correct.

D: Meniere's disease typically affects one ear first, though in some cases, it may later involve both ears. However, it does not commonly start as a bilateral condition. Severe tinnitus and vertigo are common, but the claim that it always affects both ears simultaneously is incorrect.

✅ Most inappropriate answer: D

Question 15: Regarding the impact of recurrent otitis media on communication and learning in preschool children, which statement is the most inappropriate?

Analysis of Each Option:

A: Recurrent acute otitis media (RAOM) is typically defined as three or more episodes in six months or four or more episodes in a year. The condition is more prevalent during winter and spring, when respiratory infections are more common. This statement is correct.

B: Children with cleft lip and palate, craniofacial anomalies, or Down syndrome have a higher risk of recurrent otitis media due to structural differences in the Eustachian tube, leading to poor drainage and increased susceptibility to infections. This statement is correct.

C: If a child's hearing does not recover due to persistent middle ear effusion or recurrent infections, tympanostomy (grommet insertion) or myringotomy (eardrum incision) may be recommended to improve ventilation and prevent long-term hearing deficits. This statement is correct.

D: Recurrent otitis media typically causes temporary, mild to moderate conductive hearing loss, rather than immediate severe or profound hearing loss. The fluid buildup in the middle ear dampens sound transmission, but it does not usually result in severe or permanent hearing impairment unless complications arise. This statement is incorrect.

✅ Most inappropriate answer: D

Question 16: Regarding age-related hearing loss (ARHL), which of the following statements is the most inappropriate?

Analysis of Each Option:

A: Age-related hearing loss (ARHL), or presbycusis, is a sensorineural hearing loss that primarily affects the high frequencies due to degeneration of hair cells in the cochlea, especially in the basal region. This results in a more pronounced decline in high-frequency hearing compared to mid- and low-frequency hearing. This statement is correct.

B: Central auditory processing declines with age, leading to slower auditory processing speed and a relationship with cognitive decline. Older individuals may struggle with understanding speech, particularly in noisy environments, even when their peripheral hearing ability does not seem significantly impaired. This statement is correct.

C: ARHL often causes difficulty in speech perception, leading to frequent misinterpretation of words due to impaired auditory processing, especially in noisy environments. This statement is correct.

D: The reticular activating system and auditory cortical processing decline with age, not improve. As a result, speech recognition in challenging listening environments (e.g., background noise) worsens with aging, rather than improving. Therefore, the idea that older adults perform better than younger individuals in difficult listening situations is incorrect.

✅ Most inappropriate answer: D

Question 17: Regarding middle ear function assessment in infants, which of the following statements is the most inappropriate?

Analysis of Each Option:

A: When assessing newborn middle ear function using a 220 Hz probe tone, atypical tympanograms (e.g., W-shaped or notched curves) can occur due to the developmental characteristics of the neonatal middle ear. This statement is correct.

B: In newborns, the ear canal is highly compliant and susceptible to pressure changes, which can affect tympanometric measurements. This statement is correct.

C: Single-peaked tympanograms typically emerge after a few months of age as the middle ear and ear canal mature. However, the claim that a normal tympanogram can only be obtained after 10 months is incorrect—many infants show adult-like tympanograms as early as 4–6 months. This statement is incorrect.

D: Studies show that using high-frequency probe tones (e.g., 678 Hz or 1000 Hz) in infants yields a higher likelihood of obtaining a clear single-peaked tympanogram, compared to the conventional 226 Hz probe tone, which may not be sensitive enough to detect middle ear function in neonates. This statement is correct.

✅ Most inappropriate answer: C

Question 18: Regarding remote microphone systems, which of the following statements is the most appropriate?

Analysis of Each Option:

A: According to the American Speech-Language-Hearing Association (ASHA), an effective remote microphone system should provide an improvement in signal-to-noise ratio (SNR) of at least +15 dB, not 35 dB. A +35 dB SNR increase is unrealistic and significantly exceeds typical performance levels. This statement is incorrect.

B: A remote microphone system consists of a transmitter (microphone) and a receiver, not just a transmitter and an audio cable. The transmitter captures the speaker's voice and sends it to the receiver, which delivers it directly to the listener's hearing device. This statement is incorrect.

C: While many behind-the-ear (BTE) and receiver-in-the-canal (RIC) hearing aids are compatible with remote microphone systems, not all in-the-ear (ITE) hearing aids support this feature, particularly if they lack telecoils, Bluetooth, or direct audio input (DAI) ports. This statement is incorrect.

D: Remote microphone systems can be connected using different methods, including sound field systems (which amplify sound in a room), induction loop systems (telecoil-based), and direct audio input (DAI) connections. This statement correctly describes the primary connection methods.

✅ Best answer: D

Question 19: Regarding hearing loss, which of the following statements is the most appropriate?

Analysis of Each Option:

A: Noise-induced hearing loss (NIHL) can occur in both adults and children, especially with exposure to loud environments (e.g., concerts, personal audio devices, noisy workplaces). This statement is incorrect.

B: Noise-induced hearing loss primarily affects the high-frequency range (typically around 3000–6000 Hz), not the low-frequency range. The characteristic notch at 4000 Hz is a hallmark of NIHL. This statement is incorrect.

C: Congenital aural atresia (external auditory canal closure) causes conductive hearing loss, not mixed hearing loss. Mixed hearing loss occurs when there is both conductive and sensorineural damage, which is not a standard feature of aural atresia. This statement is incorrect.

D: Bacterial meningitis is a leading cause of severe to profound sensorineural hearing loss, often due to cochlear inflammation and ossification, which can rapidly lead to permanent damage to the auditory nerve. This statement is correct.

✅ Best answer: D

Question 20: Regarding Schuknecht's (1989) classification of congenital aural atresia, which statement is the most inappropriate?

Analysis of Each Option:

A: Schuknecht's classification divides congenital aural atresia into four types (Type A, B, C, and D), which describe different degrees of external auditory canal (EAC) closure and middle ear abnormalities. This statement is correct.

B: The classification includes:

Type A (Meatal Atresia): Narrowing or closure of the external ear canal but a mostly normal middle ear.

Type B (Partial Atresia): The ear canal is narrowed or closed, and the eardrum may not be visible, but the ossicles (middle ear bones) are usually normal.

Type C (Total Atresia): The external ear canal is completely closed, and middle ear structures are often affected.

This description aligns with Schuknecht's classification. This statement is correct.

C: Type B (Partial Atresia) is defined by a narrowed ear canal, but typically, the tympanic membrane is not visible. However, stating that the ossicles are always normal is incorrect—middle ear abnormalities can be present, although they are generally less severe than in Type C or D. This statement is therefore not entirely accurate.

D: Hypopneumatic atresia refers to poor mastoid pneumatization (air cell development) and potential abnormalities of the facial nerve within the temporal bone, which can make surgical correction more complex. This statement is correct.

✅ Most inappropriate answer: C

Question 21:

Analysis of the Audiogram and Answer Selection:

This audiogram shows both air conduction (circles) and bone conduction (brackets) results. Here's what we observe:

Bone conduction thresholds (brackets) are significantly better than air conduction thresholds (circles), but not normal—indicating a mixed hearing loss (both sensorineural and conductive components).

A significant air-bone gap is present, suggesting an abnormality in sound conduction through the outer or middle ear.

Bone conduction values are not in the normal range, meaning there is also an inner ear (sensorineural) component.

The circles represent air conduction thresholds without masking, which is the correct standard notation.

Evaluating Each Option:

A: ✅ Correct – This is a mixed hearing loss because it shows both conductive (air-bone gap) and sensorineural (elevated bone conduction thresholds) components.

B: ✅ Correct – There is conductive involvement, as evidenced by the air-bone gap, meaning outer or middle ear abnormalities are present.

C: ❌ Incorrect – If the inner ear function were completely normal, the bone conduction thresholds would be within normal limits (≤25 dB HL). However, the audiogram shows elevated bone conduction thresholds, indicating a sensorineural component. This statement is not appropriate.

D: ✅ Correct – Unmasked air conduction thresholds are typically represented by circles (right ear) and Xs (left ear) in standard audiometric notation.

Final Answer:

✅ Most inappropriate answer: C

Question 22: Which of the following is NOT a cause of sensorineural hearing loss (SNHL)?

Analysis of Each Option:

A. Cholesteatoma (膽脂瘤) – ❌ Incorrect

Cholesteatoma is primarily a cause of conductive hearing loss (CHL), not SNHL.

It occurs due to abnormal skin growth in the middle ear and can erode the ossicles, leading to sound conduction issues.

However, if left untreated, it may spread to the inner ear (labyrinthine fistula), causing mixed hearing loss.

Since this is not a primary cause of SNHL, this is the correct answer to the question.

B. Auditory Neuropathy (聽神經病變) – ✅ Correct

This is a sensorineural hearing loss condition where the auditory nerve does not properly transmit signals from the inner ear to the brain.

It often results in poor speech discrimination despite normal otoacoustic emissions (OAE).

C. Inner Ear Malformations (內耳發育異常) – ✅ Correct

Congenital malformations of the cochlea, such as Mondini dysplasia or Michel aplasia, can cause sensorineural hearing loss.

D. Hair Cell Damage (內耳毛細胞缺損) – ✅ Correct

Damage to the cochlear hair cells (e.g., due to ototoxic drugs, aging, or noise exposure) is a primary cause of SNHL.

✅ Most inappropriate answer: A (Cholesteatoma)

Question 23: Which of the following statements about the Eustachian tube is the most appropriate?

Analysis of Each Option:

A. Adult Eustachian tubes are longer than those of children – ✅ Correct

The Eustachian tube is shorter, more horizontal, and more flexible in children, making them more prone to middle ear infections (otitis media).

In adults, it becomes longer, more rigid, and more angled (~45 degrees downward), improving drainage and ventilation.

B. Patulous Eustachian Tube (ET dysfunction) does not cause autophony – ❌ Incorrect

Patulous Eustachian Tube (ETD) is when the tube stays abnormally open, leading to autophony (excessive self-hearing, like hearing one's own breathing and voice loudly).

This condition can be triggered by weight loss, neurological issues, or dehydration.

C. The Eustachian tubes of both adults and children are horizontal – ❌ Incorrect

Children's Eustachian tubes are more horizontal (~10 degrees), which makes drainage and ventilation less effective.

In contrast, adult Eustachian tubes are angled (~45 degrees downward), improving function.

D. Cleft palate does not affect Eustachian tube function – ❌ Incorrect

Cleft palate patients often experience chronic Eustachian tube dysfunction, leading to recurrent otitis media with effusion (OME) and conductive hearing loss.

This occurs because the muscles responsible for opening the tube (e.g., tensor veli palatini) are often dysfunctional.

✅ Best answer: A (Adult Eustachian tubes are longer than those of children).

Question 24: Which of the following statements about otosclerosis is the most inappropriate?

Analysis of Each Option:

A. More common in Caucasians, less common in Asians and African populations – ✅ Correct

Otosclerosis has a higher prevalence in Caucasians (~10%) and is less common in Asian and African populations (~1%).

B. Usually bilateral but can progress at different rates in each ear – ✅ Correct

Otosclerosis is typically bilateral, but asymmetry in hearing loss progression is common.

C. More common in males, especially during puberty – ❌ Incorrect

Otosclerosis is more common in females than in males.

It often worsens during pregnancy due to hormonal influences, rather than occurring predominantly during puberty.

D. Carhart notch (2000 Hz dip) is a key audiometric feature – ✅ Correct

The Carhart notch (dip at 2000 Hz in bone conduction) is a hallmark of otosclerosis due to stapes fixation.

✅ Most inappropriate answer: C (Otosclerosis is more common in males, especially in puberty).

Question 25: Which of the following statements about presbycusis (age-related hearing loss) is the most inappropriate?

Analysis of Each Option:

A. The likelihood of hearing loss increases with age – ✅ Correct

There is a strong positive correlation between aging and hearing loss prevalence.

Hearing loss affects more than 60% of individuals over 70 years old.

B. Strial (vascular) degeneration is the primary cause of presbycusis – ❌ Incorrect

While strial (vascular) presbycusis is one type, the most common cause is sensory and neural degeneration (loss of hair cells and nerve fibers).

Sensory presbycusis (high-frequency hair cell loss) and neural presbycusis (loss of spiral ganglion neurons) are more common.

C. Age-related hearing loss usually starts with high frequencies – ✅ Correct

Presbycusis first affects high frequencies (3,000–8,000 Hz) before progressing to mid and low frequencies.

D. Increasing volume is more effective than slowing speech for comprehension – ✅ Correct

Older adults with presbycusis struggle with speech clarity (due to poor temporal and frequency resolution), not just volume.

Speaking slowly does not always help, but clear articulation and moderate loudness improvements are effective.

✅ Most inappropriate answer: B (Vascular degeneration is the primary cause of presbycusis).

Question 26: According to ASHA (1997) guidelines for preschool hearing screening, which statement is the most appropriate?

Analysis of Each Option:

A. Choose 500, 2000, 4000 Hz; omit 1000 Hz to save time and assess low/high frequencies – ❌ Incorrect

1000 Hz should not be omitted because it is critical for speech perception.

B. Choose 500, 1000, 2000 Hz; omit 4000 Hz to save time as it is less important for speech – ❌ Incorrect

4000 Hz is important for detecting early high-frequency hearing loss, which could impact speech clarity.

C. Choose 1000, 2000, 4000 Hz; omit 500 Hz to save time and reduce environmental noise interference – ✅ Correct

ASHA recommends screening at 1000, 2000, and 4000 Hz at 20 dB HL.

500 Hz is often omitted due to increased susceptibility to environmental noise, which may cause false positives.

D. Recommended screening level is 10–15 dB – ❌ Incorrect

ASHA guidelines recommend screening at 20 dB HL (not 10–15 dB) for preschool children.

✅ Correct answer: C (1000, 2000, 4000 Hz; omit 500 Hz due to noise interference).

Question 27: Chen reports two issues—left ear tinnitus and dizziness when exposed to loud sounds. What does issue #2 indicate?

Analysis of Each Option:

A. Tullio phenomenon – ✅ Correct

The Tullio phenomenon refers to dizziness, vertigo, or nystagmus triggered by loud sounds.

It is commonly associated with superior semicircular canal dehiscence (SSCD) or perilymph fistula.

B. Hennebert’s sign – ❌ Incorrect

Hennebert’s sign is vertigo triggered by pressure changes in the ear (e.g., pneumatic otoscopy), not loud sounds.

It is often seen in congenital syphilis or perilymph fistula.

C. Hyperacusis – ❌ Incorrect

Hyperacusis is increased sensitivity to normal environmental sounds but does not necessarily cause vertigo.

D. Schwartze’s sign – ❌ Incorrect

Schwartze’s sign refers to reddish discoloration of the promontory in otosclerosis, unrelated to sound-induced dizziness.

✅ Correct answer: A (Tullio phenomenon).

Question 28 (Descriptions about individuals with hearing impairment)

The most inappropriate option is:

B. Cochlear implant users in the early stages of training can achieve excellent results in auditory discrimination training without using visual feedback.

Explanation:

In the early stages of cochlear implant adaptation, users typically require multiple sensory cues, including visual feedback (such as lip-reading or visual prompts), to enhance their auditory recognition skills. When first receiving cochlear implant stimulation, the brain needs time to learn how to process and interpret the new auditory signals. Visual feedback helps reinforce speech recognition and comprehension. Therefore, the claim that "excellent results can be achieved without visual feedback" is inaccurate.

Question 29 (Referral and screening for children with language disorders)

The most inappropriate option is:

C. After being referred to a speech therapy unit, a therapist can diagnose a child's language problem using a screening test.

Explanation:

Screening tests are primarily used to identify children who may be at risk of language delay or disorder, but they cannot serve as formal diagnostic tools. A proper diagnosis requires comprehensive assessment,

including standardized evaluation tools, professional observation, parent and teacher questionnaires, and a detailed analysis of the child's language comprehension, expression, articulation, grammar, and social communication skills. Since screening tests are only a preliminary step in identifying potential language issues, they cannot be used alone to confirm a diagnosis.

Question 30 (Phonological Processes in Young Children)

The correct answer (not included in phonological processes) is:

C. 硬顎化 (Palatalization)

Explanation:

Phonological processes are patterns of sound changes that young children typically use to simplify speech as they develop their language skills. Common phonological processes include:

A. Reducing a CVC syllable structure to a CV structure (e.g., saying "ba" instead of "bat") – This is an example of final consonant deletion, a common process in early speech development.

B. Repeating syllables (e.g., "baba" for "bottle") – This is known as reduplication, another normal phonological process.

D. Stopping (塞音化, Plosivization) – This occurs when a child replaces a fricative or affricate sound with a stop consonant (e.g., "tun" for "sun").

However, palatalization (硬顎化) refers to a phonological phenomenon where a non-palatal sound is pronounced with a palatal quality (e.g., "s" → "ʃ" as in "miss you" pronounced as "mish you"). This process is not considered a typical phonological process in young children's speech development.

Question 31 (Why Should Deaf Infants Be Exposed to Visual Language Early?)

The most appropriate answer is:

A. Utilizing the sensitive period for language acquisition to establish a strong language foundation.

Explanation:

Research has shown that early exposure to language—whether spoken or signed—is crucial for cognitive and linguistic development. The sensitive period for language acquisition, especially during the first few years of life, allows the brain to efficiently acquire and process language.

B. "Hearing loss severity prevents spoken language learning" – This is inaccurate because many deaf or hard-of-hearing children can develop spoken language skills with hearing aids, cochlear implants, and auditory-verbal therapy.

C. "Learning sign language helps develop spoken language later" – While early sign language exposure supports overall language development, it does not directly guarantee spoken language acquisition. However, it does prevent language deprivation.

D. "Deaf parents can only provide sign language input" – While Deaf parents often use sign language at home, many also expose their children to spoken language through various means, such as cochlear implants or bilingual approaches.

The primary goal of early visual language exposure is to ensure that the child acquires a solid linguistic foundation during the critical period, preventing language deprivation and supporting cognitive and social development.

Question 32 (Using Visual Attention to Provide Language Stimulation)

The most appropriate answer is:

D. When the infant makes eye contact with the caregiver, the caregiver can immediately provide language stimulation.

Explanation:

Visual attention plays a crucial role in early language development, particularly for infants with hearing impairments. Caregivers can take advantage of moments when the infant is looking at them to provide meaningful linguistic input.

A. "Only when the infant's attention is on the caregiver is language stimulation effective" – This is incorrect because infants can still absorb language even when not directly looking at the caregiver, especially through joint attention (e.g., when both the infant and caregiver focus on an object together).

B. "Hearing children use spoken language, so visual attention is unnecessary" – This is incorrect. Even hearing children benefit from visual cues, such as facial expressions and gestures, which support speech perception and social interaction.

C. "An infant’s visual attention cannot be prompted; caregivers must wait" – This is incorrect. Caregivers can actively engage an infant’s visual attention through gestures, facial expressions, and movements to create learning opportunities.

Thus, D is the most appropriate choice because making eye contact with the infant provides an ideal moment for the caregiver to engage in language stimulation, reinforcing communication skills.

Question 33 (Order of Early Speech Development in Infants)

The correct order is:

D. ②Cooing → ①Babbling → ④Reduplicated babbling → ③Variegated babbling

Explanation:

Infants go through predictable stages in speech development:

Cooing (咕咕聲, cooing) – Around 6–8 weeks, infants produce vowel-like sounds (e.g., "oooh" and "ahhh"), which are the earliest vocalizations not yet resembling speech.

Babbling (喃語, babbling) – Around 4–6 months, infants begin experimenting with consonant-vowel combinations (e.g., "ba", "da").

Reduplicated babbling (疊語, reduplicated babbling) – Around 6–9 months, infants produce repeated syllables, such as "bababa" or "dadada".

Variegated babbling (異體喃語, variegated babbling) – Around 9–12 months, infants begin to mix different consonants and vowels (e.g., "badigu"), making their speech sound more complex and closer to real words.

Thus, D (②→①→④→③) represents the correct sequence of typical infant speech development.

Question 34 (Assessing a Child's Pragmatic Language Skills)

The most appropriate answer is:

D. "Tell me what you would do if you got lost on the street."

Explanation:

Pragmatic language skills refer to the social use of language, including turn-taking, conversational skills, understanding social cues, and using language appropriately in different contexts. The best way to assess pragmatic language is through open-ended, real-life scenarios that require the child to demonstrate problem-solving, social reasoning, and conversational abilities.

A. "Point to the picture that matches the word I say." – This assesses receptive vocabulary, not pragmatic language.

B. "Rearrange the words to form a correct sentence." – This tests syntax and grammar, not pragmatic skills.

C. "Repeat what I say." – This evaluates verbal memory and articulation, not social communication.

D is the best choice because asking a child how they would react in a specific social situation helps assess their ability to understand social expectations, problem-solving skills, and how they use language in context.

Question 35 (Least Likely Diagnosis for a 2.5-Year-Old with Limited Imitation, Gestures, and Language Skills)

The least likely diagnosis is:

A. Language impairments (語言障礙, Language Impairments).

Explanation:

The child's symptoms include:

Limited verbal vocabulary (only ~5 words at 2.5 years old).

No use of gestures.

No imitation of others.

Difficulty understanding single-step instructions and questions.

These signs suggest a broader developmental issue rather than a specific language impairment alone. Let’s analyze each condition:

A. Language impairments (語言障礙, Language Impairments) – This term usually refers to specific language impairment (SLI), which affects grammar, vocabulary, or sentence structure but does not typically include difficulties with gestures, imitation, or social interaction. Given this child's symptoms, language impairment alone is unlikely to be the sole diagnosis.

B. Social communication disorder (社交溝通障礙, Social Communication Disorder, SCD) – The lack of gesture use and imitation suggests difficulty with social communication, making this a possible diagnosis.

C. Developmental delay (發展遲緩, Developmental Delay, DD) – Since the child has delays in multiple areas (language, cognition, and social interaction), a broader developmental delay is a strong possibility.

D. Language delay (語言遲緩, Language Delay) – The child's severely limited vocabulary and lack of comprehension suggest language delay, which is a probable diagnosis.

Thus, "language impairments" alone (A) is the least likely diagnosis, as the child’s symptoms extend beyond just language difficulties into broader cognitive and social delays.

Question 36 (Speech Recognition Threshold & Word Recognition Test)

The most inappropriate statement is:

B. "WRT most commonly tests three-syllable words."

Explanation:

The Speech Recognition Threshold (SRT) and Word Recognition Test (WRT) are essential assessments for evaluating a person's ability to hear and understand speech.

A. "SRT measures the lowest volume at which a person can correctly repeat at least 50% of speech stimuli." ✅ Correct – SRT determines the minimum intensity level at which a person can recognize and repeat familiar words (often spondee words, which are two-syllable words with equal stress, such as "baseball" or "hotdog").

B. "WRT most commonly tests three-syllable words." ❌ Incorrect – WRT typically uses monosyllabic (single-syllable) words, not three-syllable words. It evaluates a person's ability to recognize and repeat words at a comfortable listening level, helping assess speech discrimination ability.

C. "SRT and WRT help evaluate auditory processing skills." ✅ Correct – Both tests provide insights into a person's hearing ability and speech perception.

D. "WRT assesses a person's ability to recognize speech." ✅ Correct – WRT measures how well a person understands words clearly, not just hears them, which is crucial for understanding speech clarity in daily communication.

Thus, B is the least appropriate statement because WRT primarily uses monosyllabic words, not three-syllable words.

Question 37 (When Is Speech Therapy Referral Not Necessary?)

The situation that does not require referral is:

D. "A 7-year-old has distorted /s/ sounds after losing front teeth."

Explanation:

Children develop speech sounds at different ages, and some speech difficulties are developmentally appropriate or temporary.

A. "A 3.5-year-old cannot produce all vowel sounds." ✅ Needs referral – By age 3, children should have mastered most vowels. Persistent difficulty may indicate articulation or phonological delay.

B. "A 5-year-old cannot correctly say /ts/." ✅ Needs referral – The /ts/ sound (e.g., "cats") should be acquired by age 4-5. Difficulty at age 5 could indicate articulation issues requiring intervention.

C. "A 6-year-old says 'an' instead of 'ah'." ✅ Needs referral – This might indicate vowel distortion or phonological processing issues, which should be assessed by a speech therapist.

D. "A 7-year-old has distorted /s/ sounds after losing front teeth." ❌ Does NOT need referral – When children lose their front teeth, it can temporarily affect their production of sounds like /s/ and /z/. This is not a pathological speech issue but rather a natural and temporary change that typically resolves once the adult teeth emerge.

Thus, D is the least concerning scenario and does not require a speech therapy referral.

Question 38 (Suprasegmental Errors in Children with Hearing Loss)

The correct answer is:

A. 重音或聲調錯誤 (Stress or Tone Errors).

Explanation:

Suprasegmental features in speech refer to elements beyond individual speech sounds, such as intonation, stress, pitch, duration, and rhythm. Children with hearing loss often struggle with these features because they rely on hearing fine variations in pitch and duration, which can be difficult with hearing impairments.

Let’s analyze each option:

A. "Errors in stress or tone" ✅ Correct – These are suprasegmental features. Hearing-impaired children may have flat intonation, incorrect stress placement, or abnormal rhythm, affecting speech naturalness.

B. "Speaking with nasalization" ❌ Incorrect – Excessive or insufficient nasalization (hypernasality or hyponasality) is a resonance issue, not a suprasegmental error.

C. "Substituting a velar sound for an alveolar sound" ❌ Incorrect – This is a phonological or articulation error (segmental error), not a suprasegmental issue.

D. "Omitting the final /ao/ sound" ❌ Incorrect – This is also a segmental error involving sound omission, not a suprasegmental issue.

Conclusion:

Since suprasegmental errors involve speech rhythm, tone, and stress, A (errors in stress or tone) is the correct answer.

Question 39 (Atypical Speech Errors in English by Children with Hearing Loss)

The correct answer is:

A. 複韻母替代單韻母 (Replacing diphthongs with monophthongs).

Explanation:

Children with hearing loss often have predictable speech production errors due to difficulty perceiving certain acoustic features of sounds. The most common English phonetic errors among hearing-impaired children include:

B. "Replacing diphthongs with monophthongs" ✅ Typical – Diphthongs (e.g., /aɪ/ in "ride") require fine auditory discrimination, which can be difficult for hearing-impaired children. They may simplify diphthongs into monophthongs (e.g., /a/ instead of /aɪ/).

C. "Replacing voiceless sounds with voiced sounds" ✅ Typical – Hearing-impaired children may struggle to perceive voicing contrasts, leading to substitutions like /b/ for /p/ or /d/ for /t/.

D. "Replacing voiced sounds with voiceless sounds" ✅ Typical – Similarly, they may substitute voiced sounds with voiceless sounds (e.g., saying "pat" instead of "bat"), depending on their specific hearing loss and access to high-frequency speech cues.

However, A (replacing monophthongs with diphthongs) is not a typical error because diphthongs are more acoustically complex and require greater auditory feedback. Instead, hearing-impaired children tend to simplify speech, not make it more complex.

Thus, A is the correct answer because this type of error is not commonly seen in hearing-impaired speech.

Question 40 (Oral Communication in Children with Hearing Parents)

The most appropriate answer is:

D. 若學習手語可以在聽人與聾人社會中轉換 (Learning sign language allows them to navigate both hearing and Deaf communities).

Explanation:

Hearing-impaired children born to hearing parents who use spoken language face unique language development challenges. Let's examine each option:

A. "Cannot become part of the Deaf community." ❌ Incorrect – Deaf identity is not solely based on having Deaf parents. Many hearing-impaired children from hearing families later embrace Deaf culture and become part of the Deaf community.

B. "Learning spoken language from infancy eliminates language development problems." ❌ Incorrect – While early access to spoken language (especially with hearing aids or cochlear implants) can support language development, these children still face challenges in speech perception, phonological development, and communication fluency.

C. "Should develop spoken language first before learning sign language." ❌ Incorrect – Research shows that early bilingual exposure to both spoken and signed language enhances cognitive and linguistic abilities, rather than delaying spoken language.

D. "Learning sign language allows them to navigate both hearing and Deaf communities." ✅ Correct – Bilingualism in spoken and signed language provides communication flexibility, social connection in both hearing and Deaf cultures, and cognitive benefits.

Thus, D is the best answer, as bilingualism enables these children to participate in both hearing and Deaf communities successfully.

Question 41 (Sign Language Interpretation of "告訴" in Different Directions)

The most appropriate answer is:

D. 我對他們都一一講過了 (I have told each of them individually).

Explanation:

In sign language, the direction of movement and repetition of a sign can change its meaning. If a Deaf person signs "告訴" (tell) towards multiple directions, this suggests that they are communicating the same information to different people.

A. "He talks too much." ❌ Incorrect – This would involve signs related to "chatty" or "talking excessively," rather than repetition in different directions.

B. "I told him repeatedly." ❌ Incorrect – Repetition in the same direction (towards one person) might indicate repeated telling, but the question specifies movement in different directions.

C. "No matter how much I say, it's useless." ❌ Incorrect – This would be expressed differently in sign language, likely with an emphasis on negation or frustration.

D. "I have told each of them individually." ✅ Correct – Signing "告訴" repeatedly in different directions suggests addressing multiple people one by one.

Thus, D is the correct interpretation based on sign language grammar and spatial usage.

Question 42 (Chinese Grammar Sign Language)

The most appropriate answer is:

C. 中文文法手語屬於人工創造的手勢碼，不是約定俗成的語言 (Chinese Grammar Sign Language is an artificially created sign system, not a naturally evolved language).

Explanation:

Chinese Grammar Sign Language (中文文法手語), also known as Manually Coded Chinese (MCC), is a system that follows spoken and written Chinese grammar, rather than the natural structure of Taiwan Sign Language (TSL). Let's analyze the options:

A. "Compared to natural sign language, Deaf students understand Chinese Grammar Sign Language more easily." ❌ Incorrect – Deaf students generally learn natural sign language (TSL) more easily because it follows their cognitive and linguistic processing. MCC, which follows spoken Chinese syntax, is often harder to understand.

B. "Chinese Grammar Sign Language lacks spatial characteristics, making it confusing." ❌ Incorrect – While MCC does not fully utilize spatial grammar like TSL, it still incorporates some spatial elements (e.g., classifiers and directional signs).

C. "Chinese Grammar Sign Language is an artificially created system, not a naturally occurring language." ✅ Correct – MCC was designed for educational purposes to match spoken Chinese grammar, making it a manually coded system rather than a natural language like TSL.

D. "According to the National Languages Development Act, Chinese Grammar Sign Language is a national language." ❌ Incorrect – The National Languages Development Act (國家語言發展法) recognizes Taiwan Sign Language (TSL) as a national language, but not MCC, since MCC is not a natural language.

Thus, C is the best answer because MCC is an artificial sign system created for educational purposes, not a naturally evolved sign language.

Question 43 (Kata Kolok and the Deaf Community in Bali)

The most inappropriate statement is:

D. 該村 Kata Kolok 的傳承主要依賴學校教師 (The transmission of Kata Kolok mainly depends on school teachers).

Explanation:

Kata Kolok is a natural village sign language that emerged in a Balinese village with a high prevalence of hereditary deafness. Unlike manually coded languages, it evolved organically within the community and is used by both Deaf and hearing villagers.

Let's analyze each option:

A. "Kata Kolok is the cultural core of the village's Deaf community." ✅ Correct – Language is a fundamental part of Deaf culture. In this village, Kata Kolok is essential for social identity and communication.

B. "Hearing children learn Kata Kolok from an early age, so Deaf individuals are not marginalized." ✅ Correct – Unlike many societies where Deaf individuals face barriers, this village integrates Deaf and hearing people through a shared sign language, reducing marginalization.

C. "Hearing villagers do not view Deaf individuals as disabled." ✅ Correct – In this village, Deafness is seen as a normal part of life, and hearing villagers communicate naturally with Deaf individuals without treating them as disabled.

D. "The transmission of Kata Kolok mainly relies on school teachers." ❌ Incorrect – The primary mode of transmission for Kata Kolok is through daily interaction within the community, especially family members and peers, not formal education. While teachers may use it in classrooms, the natural learning process occurs in social settings.

Thus, D is the most inappropriate statement, as Kata Kolok is primarily passed down through social and family interactions, rather than being formally taught in schools.

Question 44 (Ethical Issues in Court Interpreting for a Deaf Defendant)

The most appropriate answer is:

B. 僅①② (Only ① and ②).

Explanation:

In legal settings, sign language interpreters must adhere to strict ethical guidelines to ensure fairness and accuracy. The interpreter in this scenario intentionally altered the translation to favor the defendant, which is a serious violation of professional ethics.

Let's analyze each statement:

① "The interpreter has a parentalism mindset (父母心態)." ✅ Correct – Parentalism (paternalism) occurs when an interpreter or authority figure acts in a protective or controlling manner, rather than maintaining

neutrality. In this case, the interpreter interfered in the judicial process by trying to "help" the Deaf defendant.

② "The interpreter violated the Registry of Interpreters for the Deaf (RID) Code of Ethics." ✅ Correct – The RID Code of Ethics requires interpreters to remain neutral and provide accurate translations. By altering the message, the interpreter violated professional standards and compromised legal integrity.

③ "The Constitution allows for leniency in sentencing for disadvantaged groups." ❌ Incorrect – While the legal system may consider mitigating factors, there is no constitutional principle stating that all disadvantaged groups automatically receive reduced sentences.

④ "Sign language interpreters must maintain confidentiality." ❌ Incorrect (in this context) – While interpreters do have a duty of confidentiality, this does not justify altering translations. The key ethical violation here is misrepresentation, not confidentiality issues.

Thus, the best answer is B (①② only), as the interpreter demonstrated parentalism and violated ethical guidelines, but the Constitution does not guarantee reduced sentencing for marginalized groups, and confidentiality is not the main issue in this case.

Question 45 Analysis:

In Taiwan, falsely claiming to be a licensed professional (such as an audiologist) and practicing without proper certification violates the Audiologist Act or related medical regulations. According to Article 30 of the Audiologist Act, individuals who impersonate an audiologist and practice without proper certification are subject to a fine ranging from NTD 30,000 to NTD 150,000. Thus, the correct answer is:

✅ C. A fine between NTD 30,000 and NTD 150,000

Question 46 Analysis:

According to Taiwan's Medical Care Act and relevant regulations, hearing test reports are considered part of a patient's medical records, and their retention period depends on the patient's age:

For individuals under 18 years old, records must be kept until they turn 18, plus an additional 7 years.

For adults (18 and older), medical records must be kept for at least 7 years.

In this case, Tzuyu is 16 years old, meaning her medical records must be retained until she turns 23 years old (16 + 7 years), which is a minimum of 7 years. Thus, the correct answer is:

✅ B. 7 years

Question 47 Analysis:

According to the Audiologist Act in Taiwan, audiologists are legally responsible for conducting assessments and rehabilitation related to hearing and auditory function. Their professional duties include:

(A) Non-organic hearing assessments – Audiologists assess functional hearing issues, including cases where there is no physical damage but hearing difficulties exist. ✅ (Legal duty)

(B) Evaluation of hearing assistive devices – Audiologists assess patients' needs and recommend appropriate hearing aids or assistive listening devices. ✅ (Legal duty)

(C) Auditory habilitation and rehabilitation – This involves helping individuals develop or regain auditory skills after hearing loss. ✅ (Legal duty)

(D) Selling hearing assistive devices – While audiologists can recommend and fit hearing aids, direct sales are considered a commercial activity and not a core duty of an audiologist. ❌ (Not a legal duty)

Thus, the correct answer is:

✅ D. Selling hearing assistive devices

Question 48 Analysis:

In Taiwan, professionals holding both audiologist and speech-language pathologist (SLP) licenses must follow strict regulations regarding practice registration:

(A) Registering in two places and practicing both professions – This is not allowed, as dual practice locations for both roles are not permitted. ❌

(B) Registered as an SLP but still fitting hearing aids – If one registers only as an SLP, they cannot legally perform audiology-related tasks such as hearing aid fitting. ❌

(C) Dual registration in two locations but practicing only one profession – The law does not allow professionals to register both professions at two locations while limiting their practice to only one. ❌

(D) Limited to one registration location, but cannot lend out the other license – Professionals can only register in one place for one profession, and lending out their other license to another person is strictly illegal. ✅

Thus, the correct answer is:

✅ D. Practice registration is limited to one location, and the other license cannot be lent to others.

Question 49 Analysis:

When an audiologist collects patient data for research and statistical analysis, ethical review is required to protect patient rights and privacy. In Taiwan, research involving human subjects, including the collection and analysis of patient data, falls under the Human Research Act.

The proper review authority for such research is the Institutional Review Board (IRB), also known as the Human Research Ethics Committee (人體試驗倫理委員會). This committee ensures that research involving human subjects follows ethical guidelines and protects participants from harm.

Thus, the correct answer is:

✅ D. Human Research Ethics Committee (人體試驗倫理委員會)

Question 50 Analysis:

According to the Audiologist Act in Taiwan, audiologists must join the local Audiologists' Association when practicing. If the association illegally refuses membership, it violates the law, and the competent authority (government agency overseeing professional associations) will impose penalties on the association.

As stated in the law, the fine for such a violation is between NTD 30,000 and NTD 150,000.

Thus, the correct answer is:

✅ C. NTD 30,000 to NTD 150,000
