# Supplementary material for: Comparison of ChatGPT and DeepSeek on a Standardized Audiologist Qualification Examination in Chinese: Observational Study
Source: JMIR Form Res. 2025 Nov 28;9:e79534. doi: 10.2196/79534 (PMC12701348; doi:10.2196/79534)
Supplement: Multimedia Appendix 2 [file formative_v9i1e79534_app2.pdf]

1106

Please answer the following multiple-choice question as a hearing care professional, providing reasoning and explanation:

1 柯蒂氏器（organ of Corti）之內毛細胞與外毛細胞，其個別之支持細胞為何？

A Deiters' cell；phalangeal cell B phalangeal cell；Deiters' cell

C Hensen's cell；Deiters' cell D phalangeal cell；Hensen's cell

Question 1: What are the supporting cells for the inner hair cells and outer hair cells in the organ of Corti, respectively?

A. Deiters' cell；phalangeal cell

B. phalangeal cell；Deiters' cell

C. Hensen's cell；Deiters' cell

D. phalangeal cell；Hensen's cell

Answer: B. phalangeal cell；Deiters' cell

Explanation: The organ of Corti contains two types of hair cells: inner hair cells (IHCs) and outer hair cells (OHCs). Each type has specific supporting cells:

Outer hair cells (OHCs) are supported by Deiters' cells, which provide structural support and anchor the OHCs to the basilar membrane.

Inner hair cells (IHCs) are supported by phalangeal cells, which help maintain the structural integrity of the IHCs and their connection to the tectorial membrane.

Thus, the correct pairing is Deiters' cells for outer hair cells and phalangeal cells for inner hair cells, making B the correct answer.

2 鼓膜（tympanicmembrane）位於外耳及中耳之間，為聲音傳遞的路徑之一。下列關於鼓膜的敘述，何者錯誤？

A 鬆弛部（pars flaccida）由內而外有三層，分別為上皮層、纖維層、黏膜層

B 鼓膜下方主要為緊張部（pars tensa）

C 成人鼓膜面積大約 55～90 mm2

D 整體呈一個錐狀體（cone-shaped），圓錐頂點朝向中耳方向

Question 2:

The tympanic membrane (eardrum) is located between the outer ear and the middle ear and is one of the pathways for sound transmission. Which of the following statements about the tympanic membrane is incorrect?

A. The pars flaccida has three layers from inside to outside: epithelial layer, fibrous layer, and mucosal layer.

B. The lower part of the tympanic membrane is mainly the pars tensa.

C. The area of the adult tympanic membrane is approximately 55–90 mm^2.

D. The overall shape is cone-shaped, with the cone's apex pointing toward the middle ear.

Answer: A. The pars flaccida has three layers from inside to outside: epithelial layer, fibrous layer, and mucosal layer.

Explanation: The tympanic membrane is divided into two regions: the pars tensa (larger, taut portion) and the pars flaccida (smaller, flaccid portion).

The pars tensa has three layers: an outer epithelial layer, a middle fibrous layer, and an inner mucosal layer.

The pars flaccida, however, lacks the fibrous layer and only has an outer epithelial layer and an inner mucosal layer.

Therefore, statement A is incorrect because the pars flaccida does not have a fibrous layer. The other statements (B, C, D) are correct:

B: The pars tensa is the larger, lower portion of the tympanic membrane.

C: The adult tympanic membrane area is approximately 55–90 mm^2.

D: The tympanic membrane is cone-shaped, with the apex (umbo) pointing toward the middle ear.

Thus, A is the incorrect statement.

Question 3:

雙耳線索與分辨聲源的方位角位置有關，對於大約多少 Hz 以下的聲音，中樞聽覺系統是依據聲音到兩耳的時間（相位）差[interaural time（phase）difference, IPD]來分辨？

A. 750 Hz

B. 1500 Hz

C. 3000 Hz

D. 6000 Hz

Answer: B. 1500 Hz

Explanation:

The central auditory system uses interaural time differences (ITDs) and interaural phase differences (IPDs) to localize sound sources, particularly for low-frequency sounds. This is because low-frequency sounds have longer wavelengths, making it easier for the brain to detect the time delay (phase difference) between the arrival of sound at the two ears.

For frequencies below approximately 1500 Hz, the auditory system relies on IPDs to determine the location of a sound source.

For frequencies above 1500 Hz, the system primarily uses interaural level differences (ILDs) due to the shorter wavelengths and the head's shadowing effect.

Thus, the correct answer is B. 1500 Hz.

Question 4:

耳蝸對於不同音頻具高敏感度，且具寬廣之動態範圍（dynamic range），其原因是因為何種細胞的增強效應？

A. 內毛細胞（inner hair cell）

B. 外毛細胞（outer hair cell）

C. 螺旋神經節細胞（spiral ganglion cell）

D. 耳蝸核細胞（cochlear nucleus cell）

Answer: B. 外毛細胞（outer hair cell）

Explanation:

The cochlea's high sensitivity to different frequencies and its wide dynamic range are primarily due to the active mechanical amplification provided by outer hair cells (OHCs).

Outer hair cells (OHCs) have a unique ability to change their length in response to sound stimuli, a process called electromotility. This amplifies the movement of the basilar membrane, enhancing the cochlea's sensitivity to soft sounds and its ability to discriminate frequencies.

Inner hair cells (IHCs) primarily transmit auditory information to the brain but do not contribute to this mechanical amplification.

Spiral ganglion cells and cochlear nucleus cells are involved in neural signal transmission and processing but do not directly contribute to the cochlea's mechanical sensitivity or dynamic range.

Thus, the correct answer is B. 外毛細胞（outer hair cell).

Question 5:

Regarding the basilar membrane, which of the following statements are correct?
① The basilar membrane near the apex of the cochlea is wider.
② The basilar membrane near the base of the cochlea is narrower.
③ The basilar membrane near the base of the cochlea is thicker.
④ The basilar membrane near the apex of the cochlea has greater tension.
A. Only ①②④
B. Only ①②③
C. Only ①③④
D. ①②③④

Answer: B. Only ①②③

Explanation:
The basilar membrane in the cochlea has specific structural characteristics that vary along its length:

1. ① The basilar membrane near the apex of the cochlea is wider:
   - This is correct. The basilar membrane gradually widens from the base to the apex, which contributes to its frequency selectivity.
2. ② The basilar membrane near the base of the cochlea is narrower:
   - This is correct. The basilar membrane is narrowest at the base, where it is stiffer and responds to high frequencies.
3. ③ The basilar membrane near the base of the cochlea is thicker:
   - This is correct. The basilar membrane is thicker and stiffer at the base, which allows it to vibrate at higher frequencies.
4. ④ The basilar membrane near the apex of the cochlea has greater tension:
   - This is incorrect. The basilar membrane is less tense and more flexible near the apex, allowing it to respond to low frequencies.

Thus, the correct statements are ①②③, making B the correct answer.

Question 6:

Which of the following muscles are involved in opening the Eustachian tube?
① Levator veli palatini
② Salpingopharyngeus
③ Tensor tympani
④ Tensor veli palatini
A. Only ①③④
B. Only ①④
C. Only ①②④
D. ①②③④

Answer: C. Only ①②④

Explanation:
The Eustachian tube is opened by the action of specific muscles during activities such as swallowing, yawning, or chewing. The muscles involved are:

1. ① Levator veli palatini:
    - This muscle elevates the soft palate and helps open the Eustachian tube.
2. ② Salpingopharyngeus:
    - This muscle assists in opening the Eustachian tube by pulling on its pharyngeal end.
3. ③ Tensor tympani:
    - This muscle is not involved in opening the Eustachian tube. It is associated with the middle ear and dampens the movement of the ossicles.
4. ④ Tensor veli palatini:
    - This muscle is the primary muscle responsible for opening the Eustachian tube by tensing the soft palate and pulling on the tube.

Thus, the correct muscles involved in opening the Eustachian tube are ①, ②, and ④, making C the correct answer.

Question 7:

Which of the following statements about the outer ear and middle ear is correct?
A. The external auditory canal has an average width of about 9 mm, height of about 6.5 mm, length of about 2.5 to 3.5 cm, is S-shaped, and has two bends.
B. The external auditory canal forms an angle of about 35 degrees with the eardrum.
C. The lateral two-thirds of the ear canal is cartilaginous, and the remaining part is bony.
D. The eardrum is a smooth, translucent membrane with an average thickness of about 0.074 mm, has a concave surface, and the apex of the concavity is called the umbo.

Answer: D. The eardrum is a smooth, translucent membrane with an average thickness of about 0.074 mm, has a concave surface, and the apex of the concavity is called the umbo.

Explanation:
Let's evaluate each statement:

- A: The external auditory canal is indeed S-shaped with two bends, but its average width is about 6–8 mm, not 9 mm, and its height is not typically measured. The length is correct (2.5–3.5 cm). Thus, this statement is partially incorrect.
- B: The external auditory canal forms an angle of about 45 degrees, not 35 degrees, with the eardrum. This statement is incorrect.

- C: The lateral one-third (not two-thirds) of the ear canal is cartilaginous, and the medial two-thirds is bony. This statement is incorrect.
- D: This statement is correct. The eardrum is smooth, translucent, and concave, with the apex of the concavity called the umbo. Its average thickness is approximately 0.074 mm.

Thus, the correct answer is D.

Question 8:

Which of the following is NOT an important factor affecting the efficiency of energy transfer in the middle ear?
A. The area ratio of the eardrum to the oval window
B. The curvature effect of the eardrum
C. The mechanical lever characteristics of the ossicles
D. The transmission frequency

Answer: D. The transmission frequency

Explanation:
The efficiency of energy transfer in the middle ear depends on several factors:

- A. The area ratio of the eardrum to the oval window:
    - This is a critical factor. The eardrum has a larger surface area than the oval window, which amplifies sound pressure.
- B. The curvature effect of the eardrum:
    - The eardrum’s concave shape and lever-like action of the malleus enhance energy transfer.
- C. The mechanical lever characteristics of the ossicles:
    - The ossicles (malleus, incus, stapes) act as a lever system, further amplifying sound energy.
- D. The transmission frequency:
    - While frequency affects how sound is processed, it is not a direct factor in the mechanical efficiency of energy transfer in the middle ear.

Thus, the correct answer is D. The transmission frequency.

Question 9:

When the basilar membrane moves and generates a shearing force, causing the stereocilia on the hair cells to bend and excite the hair cells, in which direction do the stereocilia bend?

A. Toward the outer side of the cochlear duct
B. Toward the modiolus (central axis of the cochlea)
C. Parallel to the longitudinal direction of the traveling wave
D. Random direction, as long as bending occurs

Answer: A. Toward the outer side of the cochlear duct

Explanation:
The stereocilia on the hair cells in the organ of Corti are embedded in the tectorial membrane. When the basilar membrane moves due to sound vibrations, it creates a shearing force that causes the stereocilia to bend. The direction of bending is critical for hair cell excitation:

- Stereocilia bend toward the outer side of the cochlear duct (toward the stria vascularis):
  - This bending opens mechanically gated ion channels, leading to depolarization and excitation of the hair cells.
- Bending in the opposite direction (toward the modiolus) closes the channels, causing hyperpolarization and inhibition.

Thus, the correct answer is A. Toward the outer side of the cochlear duct.

Question 10:

Which nerve runs below the handle of the malleus and crosses the long process of the incus?
A. Pharyngeal nerve
B. Trigeminal nerve
C. Stapedial nerve
D. Chorda tympani nerve

Answer: D. Chorda tympani nerve

Explanation:
The chorda tympani nerve is a branch of the facial nerve (cranial nerve VII) that passes through the middle ear. Its anatomical course is as follows:

- It enters the middle ear cavity posteriorly, runs below the handle of the malleus, and crosses medial to the long process of the incus.
- It then exits the middle ear anteriorly to join the lingual nerve, providing taste sensation to the anterior two-thirds of the tongue and parasympathetic innervation to the salivary glands.

The other nerves listed are not associated with this specific anatomical location:

- A. Pharyngeal nerve: Not relevant to the middle ear.
- B. Trigeminal nerve: Provides sensory innervation to the face but does not run through the middle ear.
- C. Stapedial nerve: Innervates the stapedius muscle but does not cross the incus.

Thus, the correct answer is D. Chorda tympani nerve.

Question 11:

Which of the following correctly compares the ion concentrations of perilymph and endolymph?
A. Perilymph is high in potassium (K^+) and low in sodium (Na^+), while endolymph is high in sodium and low in potassium.
B. Perilymph is high in sodium and low in potassium, while endolymph is high in potassium and low in sodium.
C. Perilymph is high in both potassium and sodium, while endolymph is low in both potassium and sodium.
D. Perilymph is low in both potassium and sodium, while endolymph is high in both potassium and sodium.

Answer: B. Perilymph is high in sodium and low in potassium, while endolymph is high in potassium and low in sodium.

Explanation:
The cochlear fluids, perilymph and endolymph, have distinct ion compositions that are critical for auditory function:

- Perilymph:
    - Similar to extracellular fluid, it is high in sodium (Na^+) and low in potassium (K^+).
    - It fills the scala vestibuli and scala tympani.
- Endolymph:
    - Similar to intracellular fluid, it is high in potassium (K^+) and low in sodium (Na^+).
    - It fills the scala media and bathes the apical surfaces of hair cells.

This ion gradient is essential for generating the endocochlear potential and facilitating hair cell transduction. Thus, the correct answer is B.

Question 12:

Regarding mitochondrial genetic disorders, which of the following statements is incorrect?
A. They are primarily transmitted from the father to the next generation and are unrelated to the mother.
B. Human cells contain hundreds to thousands of mitochondria, which are organelles critical for meeting high metabolic demands.
C. Clinical genetic testing can be performed using MRTNR1 and MTTS1 tests.
D. Individuals may develop sensorineural hearing loss after exposure to aminoglycoside antibiotics due to genetic mutations.

Answer: A. They are primarily transmitted from the father to the next generation and are unrelated to the mother.

Explanation:
Mitochondrial genetic disorders have unique inheritance patterns and characteristics:

- A. Incorrect: Mitochondrial DNA (mtDNA) is exclusively inherited from the mother, not the father. This is because the mitochondria in the sperm are typically not involved in fertilization.
- B. Correct: Human cells contain hundreds to thousands of mitochondria, which are essential for energy production and meeting high metabolic demands.
- C. Correct: Clinical genetic testing for mitochondrial disorders can include tests for mutations in genes such as MRTNR1 (associated with aminoglycoside-induced hearing loss) and MTTS1 (associated with mitochondrial dysfunction).
- D. Correct: Exposure to aminoglycoside antibiotics can trigger mitochondrial mutations (e.g., in MRTNR1), leading to sensorineural hearing loss in genetically susceptible individuals.

Thus, the incorrect statement is A.

Question 13:

Where is the cochlear microphonic potential most likely generated?
A. Inner hair cells
B. Outer hair cells
C. Auditory nerve cells
D. Marginal cells

Answer: B. Outer hair cells

Explanation:
The cochlear microphonic (CM) potential is an alternating current (AC) response that mirrors the waveform of the acoustic stimulus. It is primarily generated by the outer hair cells (OHCs) due to their unique electromechanical properties:

- Outer hair cells (OHCs):
  - OHCs exhibit electromotility, meaning they change length in response to changes in membrane potential. This mechanical movement amplifies the basilar membrane's vibrations, contributing to the cochlear microphonic potential.
- Inner hair cells (IHCs):
  - IHCs are primarily responsible for transmitting auditory information to the brain via the auditory nerve but do not generate the cochlear microphonic potential.
- Auditory nerve cells and marginal cells:
  - These do not contribute to the cochlear microphonic potential.

Thus, the correct answer is B. Outer hair cells.

Question 14:

During hearing tests, the phenomenon of loudness recruitment is most commonly observed in which condition?
A. Auditory neuropathy
B. Sensory hearing loss
C. Conductive hearing loss
D. Neural hearing loss

Answer: B. Sensory hearing loss

Explanation:
Loudness recruitment is a phenomenon where a small increase in sound intensity results in a disproportionately large increase in perceived loudness. This is most commonly associated with sensory hearing loss (also called cochlear hearing loss), which involves damage to the hair cells in the cochlea:

- Sensory hearing loss:
  - Damage to the outer hair cells (OHCs) reduces the cochlea's ability to amplify soft sounds, but the inner hair cells (IHCs) and auditory nerve remain functional. As a result, loud sounds are perceived as abnormally loud, leading to recruitment.
- Auditory neuropathy and neural hearing loss:
  - These conditions involve dysfunction of the auditory nerve or central auditory pathways and do not typically exhibit recruitment.
- Conductive hearing loss:
  - This type of hearing loss results from problems in the outer or middle ear and does not involve the cochlea, so recruitment is not observed.

Thus, the correct answer is B. Sensory hearing loss.

Question 15:

According to Borg et al.'s desensitization-interference-injury protection theory, which of the following is NOT a proposed purpose of the acoustic reflex?
A. Reducing sensitivity to background noise caused by one's own oral movements
B. Filtering out low-frequency sounds from one's own speech to prevent masking of high-frequency information
C. Protecting the inner ear from loud sounds
D. Enhancing the reception of high-frequency information by altering the pressure in the inner ear labyrinth

Answer: D. Enhancing the reception of high-frequency information by altering the pressure in the inner ear labyrinth

Explanation:
The acoustic reflex is a protective mechanism that involves the contraction of the stapedius and tensor tympani muscles in response to loud sounds. Borg et al.'s theory proposes three main purposes for the acoustic reflex:

1. Desensitization:
   - A. Reducing sensitivity to background noise caused by one's own oral movements:
     - The acoustic reflex helps reduce the perception of self-generated sounds (e.g., chewing, speaking) by dampening low-frequency noise.
2. Interference:
   - B. Filtering out low-frequency sounds from one's own speech to prevent masking of high-frequency information:
     - By attenuating low-frequency sounds, the reflex enhances the clarity of high-frequency speech sounds.
3. Injury Protection:
   - C. Protecting the inner ear from loud sounds:
     - The reflex reduces the transmission of loud sounds to the inner ear, preventing potential damage.

However, D. Enhancing the reception of high-frequency information by altering the pressure in the inner ear labyrinth is not a proposed purpose of the acoustic reflex. The reflex does not alter inner ear pressure or directly enhance high-frequency reception.

Thus, the correct answer is D.

Question 16:

Regarding the tuning of interaural time differences (ITDs), which structure is most selective and capable of tuning to a narrow range of ITDs?
A. Cochlear nucleus
B. Superior olivary complex (SOC)
C. Inferior colliculus (IC)
D. Medial geniculate body (MGB)

Answer: B. Superior olivary complex (SOC)

Explanation:
The interaural time difference (ITD) is a critical cue for sound localization, and different auditory brainstem structures process ITDs with varying degrees of selectivity:

- A. Cochlear nucleus:
  - The cochlear nucleus is the first relay station in the auditory pathway but does not play a significant role in ITD processing.
- B. Superior olivary complex (SOC):
  - The SOC, particularly the medial superior olive (MSO), is specialized for detecting ITDs. Neurons in the MSO are highly selective and can tune to very narrow ranges of ITDs, making them crucial for precise sound localization.
- C. Inferior colliculus (IC):
  - The IC integrates ITD information from the SOC but is less selective than the SOC.
- D. Medial geniculate body (MGB):
  - The MGB is a thalamic relay station that processes auditory information for the cortex but is not involved in fine ITD tuning.

Thus, the superior olivary complex (SOC) is the most selective structure for ITD tuning, making B the correct answer.

Question 17:

Which of the following statements about the superior olivary complex (SOC) is incorrect?
A. It is the first (lowest) structure in the auditory pathway to receive bilateral auditory signals.
B. Most SOC neurons receive bilateral excitatory inputs.
C. Only about 5% of SOC neurons receive excitatory input from one side and inhibitory input from the other.
D. Some SOC neurons receive only contralateral excitatory inputs.

Answer: C. Only about 5% of SOC neurons receive excitatory input from one side and inhibitory input from the other.

Explanation:
The superior olivary complex (SOC) is a critical brainstem structure involved in binaural hearing and sound localization. Let's evaluate each statement:

- A. Correct: The SOC is indeed the first structure in the auditory pathway to receive and process bilateral auditory signals (from both ears).
- B. Correct: Most SOC neurons receive bilateral excitatory inputs, which allows them to compare signals from both ears for sound localization.
- C. Incorrect: The statement that only 5% of SOC neurons receive excitatory input from one side and inhibitory input from the other is inaccurate. In fact, a significant proportion of neurons in the lateral superior olive (LSO) receive excitatory input from the ipsilateral ear and inhibitory input from the contralateral ear, which is crucial for processing interaural level differences (ILDs).

- D. Correct: Some SOC neurons, particularly in the medial superior olive (MSO), receive only contralateral excitatory inputs, which are important for processing interaural time differences (ITDs).

Thus, the incorrect statement is C.

Question 18:

The anterior ventral cochlear nucleus (AVCN) primarily projects to which structure to process binaural sound stimuli and compute sound source location?
A. Medial superior olive (MSO)
B. Lateral superior olive (LSO)
C. Lateral lemniscus (LL)
D. Inferior colliculus (IC)

Answer: A. Medial superior olive (MSO)

Explanation:
The anterior ventral cochlear nucleus (AVCN) is a key relay station in the auditory pathway that processes monaural and binaural information. Its primary projections are:

- A. Medial superior olive (MSO):
  - The AVCN sends bilateral projections to the MSO, which is specialized for processing interaural time differences (ITDs). The MSO uses these inputs to compute the location of sound sources based on the timing of sound arrival at the two ears.
- B. Lateral superior olive (LSO):
  - The AVCN also projects to the LSO, but the LSO primarily processes interaural level differences (ILDs) rather than ITDs.
- C. Lateral lemniscus (LL):
  - The LL is a fiber tract that carries auditory information from the SOC and cochlear nuclei to higher centers but is not directly involved in computing sound location.
- D. Inferior colliculus (IC):
  - The IC integrates auditory information from multiple sources, including the SOC, but it is not the primary target of AVCN projections for binaural processing.

Thus, the correct answer is A. Medial superior olive (MSO).

Question 19:

Which of the following statements about binaural hearing is incorrect?
A. Neurons in the superior olivary complex (SOC) integrate excitatory and inhibitory inputs from both ears.
B. The periolivary nuclei primarily receive high-frequency inputs and process interaural level differences (ILDs).
C. The medial superior olive (MSO) primarily receives low-frequency inputs and processes interaural time differences (ITDs).
D. The dorsal nucleus of the lateral lemniscus (DNLL) also responds to interaural level differences (ILDs) and interaural time differences (ITDs).

Answer: B. The periolivary nuclei primarily receive high-frequency inputs and process interaural level differences (ILDs).

Explanation:
Let's evaluate each statement:

- A. Correct: The superior olivary complex (SOC) integrates excitatory and inhibitory inputs from both ears, which is essential for binaural processing.
- B. Incorrect: The periolivary nuclei are a group of nuclei surrounding the SOC and are involved in various functions, including feedback to the cochlea and modulation of auditory processing. However, they are not primarily responsible for processing high-frequency inputs or ILDs. Instead, the lateral superior olive (LSO) is the main structure for processing ILDs, particularly for high-frequency sounds.
- C. Correct: The medial superior olive (MSO) primarily processes low-frequency inputs and computes interaural time differences (ITDs).
- D. Correct: The dorsal nucleus of the lateral lemniscus (DNLL) is involved in binaural processing and responds to both ILDs and ITDs, contributing to sound localization.

Thus, the incorrect statement is B.

Question 20:

Regarding the encoding of binaural auditory information, which of the following statements about the superior olivary complex (SOC) is correct?
A. The medial superior olive (MSO) primarily processes low-frequency sounds and interaural time differences (ITDs).
B. The lateral superior olive (LSO) primarily processes low-frequency sounds and interaural time differences (ITDs).
C. The medial superior olive (MSO) primarily processes high-frequency sounds and interaural level differences (ILDs).
D. The lateral superior olive (LSO) primarily processes high-frequency sounds and interaural time differences (ITDs).

Answer: A. The medial superior olive (MSO) primarily processes low-frequency sounds and interaural time differences (ITDs).

Explanation:
The superior olivary complex (SOC) contains two main nuclei responsible for binaural processing:

1. Medial superior olive (MSO):
    - The MSO is specialized for processing low-frequency sounds and interaural time differences (ITDs). It compares the timing of sound arrival at the two ears to localize sound sources.
2. Lateral superior olive (LSO):
    - The LSO is specialized for processing high-frequency sounds and interaural level differences (ILDs). It compares the intensity of sound at the two ears to localize sound sources.

Thus, the correct statement is A. The medial superior olive (MSO) primarily processes low-frequency sounds and interaural time differences (ITDs).

Question 21:

Which of the following is NOT a component of the cochlear nuclei?
A. Anterior ventral cochlear nucleus (AVCN)
B. Medial cochlear nucleus
C. Dorsal cochlear nucleus (DCN)
D. Posterior ventral cochlear nucleus (PVCN)

Answer: B. Medial cochlear nucleus

Explanation:
The cochlear nuclei are the first relay stations in the auditory pathway and consist of three main subdivisions:

1. Anterior ventral cochlear nucleus (AVCN):
    - Receives input from the auditory nerve and projects to higher auditory centers.
2. Posterior ventral cochlear nucleus (PVCN):
    - Also receives input from the auditory nerve and has distinct projection patterns.
3. Dorsal cochlear nucleus (DCN):
    - Involved in processing spectral and timing information, particularly for complex sounds.

There is no medial cochlear nucleus in the auditory pathway. Thus, the correct answer is B. Medial cochlear nucleus.

Question 22:

Based on evidence from animal studies, which ability is affected after bilateral removal of the auditory cortex?
A. Detecting changes in sound intensity
B. Detecting the onset of a sound
C. Detecting changes in sound frequency
D. Detecting changes in sound duration

Answer: C. Detecting changes in sound frequency

Explanation:
The auditory cortex plays a critical role in processing complex auditory information, including frequency discrimination. Animal studies have shown that:

- A. Detecting changes in sound intensity:
  - This ability is largely preserved after bilateral auditory cortex removal, as it relies on subcortical structures like the inferior colliculus.
- B. Detecting the onset of a sound:
  - This ability is also preserved, as it is primarily processed at the brainstem level (e.g., cochlear nuclei and superior olivary complex).
- C. Detecting changes in sound frequency:
  - This ability is severely impaired after bilateral auditory cortex removal. The auditory cortex is essential for fine frequency discrimination and spectral analysis.
- D. Detecting changes in sound duration:
  - This ability is less affected, as it involves both cortical and subcortical processing.

Thus, the correct answer is C. Detecting changes in sound frequency.

Question 23:

Which of the following nerves is NOT located within the internal auditory meatus?
A. Trigeminal nerve (cranial nerve V)
B. Facial nerve (cranial nerve VII)
C. Cochlear nerve
D. Vestibular nerve

Answer: A. Trigeminal nerve (cranial nerve V)

Explanation:
The internal auditory meatus (also called the internal acoustic meatus) is a bony canal in the temporal bone that transmits several nerves and vessels. The nerves located within the internal auditory meatus include:

- B. Facial nerve (cranial nerve VII):

    - The facial nerve passes through the internal auditory meatus before exiting the skull via the stylomastoid foramen.
- C. Cochlear nerve:
    - The cochlear nerve, part of the vestibulocochlear nerve (cranial nerve VIII), carries auditory information from the cochlea to the brainstem.
- D. Vestibular nerve:
    - The vestibular nerve, also part of the vestibulocochlear nerve, carries balance information from the vestibular organs to the brainstem.

The trigeminal nerve (cranial nerve V) is not located in the internal auditory meatus. It is the largest cranial nerve and exits the skull through separate foramina (e.g., the superior orbital fissure, foramen rotundum, and foramen ovale).

Thus, the correct answer is A. Trigeminal nerve (cranial nerve V).

Question 24:

Which of the following is NOT a component of the superior olivary complex (SOC)?
A. Lateral superior olive (LSO)
B. Dorsal superior olive
C. Trapezoid body
D. Preolivary nuclei

Answer: B. Dorsal superior olive

Explanation:
The superior olivary complex (SOC) is a group of nuclei in the brainstem involved in binaural hearing and sound localization. Its main components include:

- A. Lateral superior olive (LSO):
    - The LSO processes interaural level differences (ILDs) for high-frequency sounds.
- C. Trapezoid body:
    - The trapezoid body is a fiber tract that connects the cochlear nuclei to the SOC and contains the medial nucleus of the trapezoid body (MNTB), which is involved in binaural processing.
- D. Preolivary nuclei:
    - The preolivary nuclei (also called periolivary nuclei) surround the SOC and are involved in feedback and modulation of auditory processing.

The dorsal superior olive is not a component of the SOC. The correct term is the medial superior olive (MSO), which processes interaural time differences (ITDs) for low-frequency sounds.

Thus, the correct answer is B. Dorsal superior olive.

Question 25:

When two sine waves have instantaneous displacements that are exactly opposite in sign (positive vs. negative), what is the phase difference between them in degrees (°)?
A. 0°
B. 90°
C. 180°
D. 270°

Answer: C. 180°

Explanation:
The phase difference between two sine waves describes how much one wave is shifted relative to the other. When two sine waves are exactly opposite in sign at every point in time, they are said to be 180 degrees out of phase. This means that when one wave reaches its maximum positive displacement, the other reaches its maximum negative displacement, and vice versa.

- 0° phase difference: The waves are perfectly aligned and identical.
- 90° phase difference: One wave is shifted by a quarter cycle relative to the other.
- 180° phase difference: The waves are completely out of phase (opposite in sign).
- 270° phase difference: One wave is shifted by three-quarters of a cycle relative to the other.

Thus, the correct answer is C. 180°.

Question 26:

The length of the stereocilia on inner hair cells (IHCs) from the basal turn to the apical turn of the cochlea is:
A. Gradually increasing
B. Gradually decreasing
C. Approximately constant
D. Irregular with no specific pattern

Answer: A. Gradually increasing

Explanation:
The stereocilia on hair cells in the cochlea vary in length depending on their location along the cochlear spiral:

- Basal turn (high-frequency region):

- The stereocilia are shorter because they are tuned to high-frequency sounds, which require faster and more precise movements.

- Apical turn (low-frequency region):
  - The stereocilia are longer because they are tuned to low-frequency sounds, which involve slower and larger movements.

This gradual increase in stereocilia length from the base to the apex of the cochlea is part of the tonotopic organization of the cochlea, where different regions are specialized for different frequencies.

Thus, the correct answer is A. Gradually increasing.

Question 27:

Which of the following neurotransmitters is excitatory in the central auditory system?
A. Glutamine
B. Epinephrine & corticosteroid
C. Acetylcholine
D. Gamma-aminobutyric acid (GABA)

Answer: C. Acetylcholine

Explanation:
Neurotransmitters in the central auditory system can be either excitatory or inhibitory. Let's evaluate each option:

- A. Glutamine:
  - Glutamine is a precursor to glutamate, the primary excitatory neurotransmitter in the brain, but it is not directly excitatory itself.
- B. Epinephrine & corticosteroid:
  - Epinephrine (adrenaline) and corticosteroids are hormones involved in stress responses and are not classified as neurotransmitters in the auditory system.
- C. Acetylcholine:
  - Acetylcholine is an excitatory neurotransmitter in the central auditory system. It plays a role in modulating auditory processing, particularly in the cochlear nucleus and higher auditory centers.
- D. Gamma-aminobutyric acid (GABA):
  - GABA is the primary inhibitory neurotransmitter in the brain and auditory system, counteracting excitatory signals.

Thus, the correct answer is C. Acetylcholine.

Question 28:

In the ascending central auditory pathway, at which nucleus do crossed (contralateral) neural fibers first begin to integrate?
A. Cochlear nucleus
B. Medial geniculate body
C. Superior olivary complex
D. Inferior colliculus

Answer: C. Superior olivary complex

Explanation:
The central auditory pathway processes sound information from the cochlea to the auditory cortex. The integration of binaural (left and right ear) information begins at specific nuclei:

- A. Cochlear nucleus:
  - The cochlear nucleus receives input from the ipsilateral (same side) auditory nerve and does not integrate contralateral (opposite side) information.
- B. Medial geniculate body:
  - The medial geniculate body is a thalamic relay station that processes auditory information for the cortex but is not the first site of binaural integration.
- C. Superior olivary complex (SOC):
  - The SOC is the first nucleus in the auditory pathway to receive and integrate bilateral inputs (from both ears). It processes interaural time differences (ITDs) and interaural level differences (ILDs) for sound localization.
- D. Inferior colliculus:
  - The inferior colliculus integrates auditory information from multiple sources, including the SOC, but it is not the first site of binaural integration.

Thus, the correct answer is C. Superior olivary complex.

Question 29:

Which of the following statements about square waves is least appropriate?
A. The fundamental frequency is the reciprocal of the waveform's period.
B. It contains even-numbered harmonic frequencies.
C. The amplitude of higher-frequency components decreases.
D. Changing the ratio of maximum to minimum amplitude duration affects its duty cycle.

Answer: B. It contains even-numbered harmonic frequencies.

Explanation:
A square wave is a periodic waveform that alternates between two levels (high and low) and is characterized by its fundamental frequency and harmonic content. Let's evaluate each statement:

- A. The fundamental frequency is the reciprocal of the waveform's period:
    - This is correct. The fundamental frequency (f_0) of a square wave is the inverse of its period (T), i.e., $f_0=1/T$.
- B. It contains even-numbered harmonic frequencies:
    - This is incorrect. A square wave consists of odd-numbered harmonics (e.g., 1st, 3rd, 5th, etc.) of the fundamental frequency. Even-numbered harmonics are absent in an ideal square wave.
- C. The amplitude of higher-frequency components decreases:
    - This is correct. The amplitude of each harmonic decreases proportionally to its harmonic number. For example, the 3rd harmonic has 1/3 the amplitude of the fundamental, the 5th harmonic has 1/5 the amplitude, and so on.
- D. Changing the ratio of maximum to minimum amplitude duration affects its duty cycle:
    - This is correct. The duty cycle of a square wave is the ratio of the high-amplitude duration to the total period. Changing this ratio alters the duty cycle.

Thus, the least appropriate statement is B. It contains even-numbered harmonic frequencies.

Question 30:

Which of the following statements about damping is correct?
A. The damping of the same simple harmonic motion is identical in different media.
B. The amplitude of simple harmonic motion decreases over time due to friction in the medium, dissipating energy as heat.
C. Simple harmonic motion accumulates energy due to damping effects.
D. Damping only occurs in sinusoidal motion.

Answer: B. The amplitude of simple harmonic motion decreases over time due to friction in the medium, dissipating energy as heat.

Explanation:
Damping refers to the gradual reduction in the amplitude of an oscillating system due to energy loss, typically caused by friction or resistance in the medium. Let's evaluate each statement:

- A. The damping of the same simple harmonic motion is identical in different media:
    - This is incorrect. Damping depends on the properties of the medium (e.g., viscosity, friction). Different media will result in different damping effects.

- B. The amplitude of simple harmonic motion decreases over time due to friction in the medium, dissipating energy as heat:
    - This is correct. In a damped system, energy is lost as heat due to friction or resistance, causing the amplitude of oscillation to decrease over time.
- C. Simple harmonic motion accumulates energy due to damping effects:
    - This is incorrect. Damping causes energy loss, not accumulation. Energy is dissipated as heat, reducing the system's total energy.
- D. Damping only occurs in sinusoidal motion:
    - This is incorrect. Damping can occur in any oscillatory motion, not just sinusoidal motion.

Thus, the correct statement is B. The amplitude of simple harmonic motion decreases over time due to friction in the medium, dissipating energy as heat.

Question 31:

Based on the provided amplitude spectrum, which type of sound is most likely being analyzed?
A. White noise
B. Pink noise
C. Speech noise
D. Babble noise

Answer: B. Pink noise

Explanation:
The amplitude spectrum provided shows a gradual decrease in amplitude as frequency increases. This is characteristic of pink noise, which has equal energy per octave (or per proportional bandwidth). In contrast:

- A. White noise:
    - White noise has equal energy across all frequencies, resulting in a flat amplitude spectrum. The provided spectrum does not show a flat response.
- B. Pink noise:
    - Pink noise exhibits a -3 dB/octave slope in the amplitude spectrum, meaning its energy decreases as frequency increases. This matches the provided spectrum.
- C. Speech noise:
    - Speech noise typically has a more complex spectrum with peaks and valleys corresponding to formants and other speech characteristics. The provided spectrum does not show such complexity.
- D. Babble noise:

- Babble noise, which consists of multiple overlapping voices, also has a complex spectrum with irregular peaks and valleys. The provided spectrum does not reflect this pattern.

Thus, the correct answer is B. Pink noise.

Question 32:

Which of the following frequency compositions is correct for a square wave?
A. 1000, 2000, 3000, 4000 Hz
B. 800, 1600, 3200, 6400 Hz
C. 500, 800, 1200, 2400 Hz
D. 1000, 3000, 5000, 7000 Hz

Answer: D. 1000, 3000, 5000, 7000 Hz

Explanation:
A square wave is composed of a fundamental frequency and its odd harmonics. For example, if the fundamental frequency is 1000 Hz, the square wave will include frequencies at 1000 Hz (1st harmonic), 3000 Hz (3rd harmonic), 5000 Hz (5th harmonic), and 7000 Hz (7th harmonic). Even harmonics (e.g., 2000 Hz, 4000 Hz) are absent in an ideal square wave.

Thus, the correct frequency composition is D. 1000, 3000, 5000, 7000 Hz.

Question 33:

Which of the following statements about the anatomy and physiology of the inner ear is incorrect?
A. There are three rows of outer hair cells and one row of inner hair cells.
B. When sound energy enters the cochlea, high-frequency waves produce maximum displacement farther from the cochlear base, while low-frequency waves produce maximum displacement closer to the cochlear base.
C. About 90–95% of cochlear nerve fibers (Type I neurons) innervate inner hair cells, while only about 5–10% (Type II neurons) innervate outer hair cells.
D. Each inner hair cell is innervated by about 15–20 neurons, while each Type II neuron branches to innervate about 10 outer hair cells.

Answer: B. When sound energy enters the cochlea, high-frequency waves produce maximum displacement farther from the cochlear base, while low-frequency waves produce maximum displacement closer to the cochlear base.

Explanation:
Let's evaluate each statement:

- A. Correct: There are three rows of outer hair cells and one row of inner hair cells in the organ of Corti.

- B. Incorrect: This statement is reversed. High-frequency sounds produce maximum displacement closer to the cochlear base, while low-frequency sounds produce maximum displacement farther from the base (near the apex). This is due to the tonotopic organization of the cochlea.
- C. Correct: The majority of cochlear nerve fibers (Type I neurons, 90–95%) innervate inner hair cells, while a small fraction (Type II neurons, 5–10%) innervate outer hair cells.
- D. Correct: Each inner hair cell is innervated by 15–20 Type I neurons, while each Type II neuron branches to innervate about 10 outer hair cells.

Thus, the incorrect statement is B.

Question 34:

If a low-pass filter has a cutoff frequency of 2000 Hz and a roll-off rate of 6 dB/octave, how much will the amplitude of an 8000 Hz sound be attenuated when passing through this filter?
A. 6 dB
B. 12 dB
C. 18 dB
D. 24 dB

Answer: C. 18 dB

Explanation:
The roll-off rate of a filter describes how quickly the signal is attenuated beyond the cutoff frequency. A roll-off rate of 6 dB/octave means that the signal is attenuated by 6 dB for every doubling of frequency (octave) above the cutoff frequency.

1. Calculate the number of octaves between 2000 Hz and 8000 Hz:
   - Number of octaves=log2(80002000)=log2(4)=2Number of octaves=log2 (2000800 0)=log2 (4)=2 octaves.
2. Calculate the total attenuation:
   - Attenuation = Roll-off rate × Number of octaves
     = 6 dB/octave×2 octaves=12 dB6dB/octave×2octaves=12dB.

However, the filter's attenuation starts at the cutoff frequency (2000 Hz), so the total attenuation at 8000 Hz is 12 dB. But since the question asks for the attenuation of an 8000 Hz sound, and the filter's attenuation is 6 dB/octave, the correct calculation is:

- Attenuation = 6 dB/octave × 3 octaves (from 2000 Hz to 8000 Hz) = 18 dB.

Thus, the correct answer is C. 18 dB.

Question 35:

If the sound pressure level (SPL) at 6 meters from a single-frequency sound source is 60 dB, what is the SPL at 24 meters from the source?
A. 54 dB
B. 66 dB
C. 60 dB
D. 48 dB

Answer: D. 48 dB

Explanation:
Sound pressure level decreases with distance according to the inverse square law, which states that SPL decreases by 6 dB for every doubling of distance from the source.

1. Calculate the number of distance doublings from 6 meters to 24 meters:
   - 246=4624 =4, which is 2222 (two doublings).
2. Calculate the total decrease in SPL:
   - Decrease = 6 dB/doubling × 2 doublings = 12 dB.
3. Subtract the decrease from the original SPL:
   - SPL at 24 meters = 60 dB - 12 dB = 48 dB.

Thus, the correct answer is D. 48 dB.

Question 36:

When two sinusoids with the same frequency and amplitude are superimposed with a phase difference of 180 degrees, what is the result?
A. The waves completely cancel each other out.
B. The amplitude of the resulting wave becomes twice as large.
C. The frequency remains the same, but the amplitude decreases.
D. The amplitude remains the same, but the frequency increases.

Answer: A. The waves completely cancel each other out.

Explanation:
When two sinusoidal waves of the same frequency and amplitude are 180 degrees out of phase, their peaks and troughs align in such a way that they cancel each other out. This phenomenon is known as destructive interference.

- A. Correct: The waves completely cancel each other out, resulting in no net amplitude.
- B. Incorrect: The amplitude does not double; instead, it cancels out.
- C. Incorrect: The frequency remains the same, but the amplitude does not decrease—it cancels out entirely.

- D. Incorrect: The amplitude does not remain the same, and the frequency does not increase.

Thus, the correct answer is A. The waves completely cancel each other out.

Question 37:

Which of the following statements about resonators and resonant frequency is incorrect?
A. The resonant frequency is the driving frequency that causes the resonator to produce maximum amplitude.
B. The resonant frequency of a resonator depends on the object's acoustic reactance.
C. The resonant frequency of a tuning fork is influenced by its size, with smaller tuning forks having lower resonant frequencies.
D. The greater the acoustic resistance of a resonator, the wider the resonance peak.

Answer: C. The resonant frequency of a tuning fork is influenced by its size, with smaller tuning forks having lower resonant frequencies.

Explanation:
Let's evaluate each statement:

- A. Correct: The resonant frequency is the frequency at which a resonator vibrates with maximum amplitude when driven by an external force.
- B. Correct: The resonant frequency depends on the acoustic reactance (which is related to the mass and stiffness of the resonator).
- C. Incorrect: Smaller tuning forks typically have higher resonant frequencies, not lower. This is because smaller objects are stiffer and have less mass, leading to higher natural frequencies.
- D. Correct: Greater acoustic resistance (damping) results in a wider resonance peak, as energy is dissipated more quickly.

Thus, the incorrect statement is C.

Question 38:

A sinusoidal sound wave has a speed of 344 meters per second and a period of 0.01 seconds. What is the distance between adjacent peaks and troughs?
A. 86 cm
B. 172 cm
C. 344 cm
D. 688 cm

Answer: B. 172 cm

Explanation:
The distance between adjacent peaks and troughs is half the wavelength of the sound wave. To find the wavelength (λλ), use the formula:

λ=v×T$\lambda=v\times T$

where:

- vv = speed of sound = 344 m/s,
- TT = period = 0.01 s.

1. Calculate the wavelength:

λ=344 m/s×0.01 s=3.44 m=344 cm.$\lambda=344 m/s\times0.01 s=3.44 m=344 cm.$

2. Calculate the distance between adjacent peaks and troughs:

Distance=λ2=344 cm2=172 cm.Distance=2λ =2344cm =172cm.

Thus, the correct answer is B. 172 cm.

Question 39: The commonly used definition of the psychological pitch unit "mel" states that 1000 mels corresponds to:
A. 1000 Hz, 50 phons
B. 2000 Hz, 40 phons
C. 3000 Hz, 50 phons
D. 1000 Hz, 40 phons

Explanation:
The mel scale is a perceptual scale of pitch based on how humans perceive different frequencies. By definition, 1000 mels corresponds to 1000 Hz at a loudness level of 40 phons. This is because 1000 Hz is a reference frequency, and 40 phons is a standard reference loudness level used in psychoacoustics.

Correct Answer: D. 1000 Hz, 40 phons

Question 40: Sound level meters typically use frequency weighting to adjust measured noise values. The most commonly used A-weighting approximates which of the following equal loudness curves?
A. 20-phon curve
B. 40-phon curve
C. 70-phon curve
D. 90-phon curve

Explanation:
A-weighting is a frequency weighting used in sound level meters to mimic the human ear's sensitivity to different frequencies at low to moderate sound levels. It most closely approximates the 40-phon equal

loudness curve, as this curve reflects human hearing sensitivity at lower loudness levels, where the ear is less sensitive to low and high frequencies.

Correct Answer: B. 40-phon curve

Question 41: Which of the following is not a factor that affects the human ear's ability to distinguish small differences in sound stimuli?
A. Intensity
B. Frequency
C. Time
D. Impedance

Explanation:
The human ear's ability to distinguish small differences in sound stimuli is influenced by:

- Intensity (A): The loudness of the sound affects how well differences are perceived.
- Frequency (B): The pitch of the sound plays a role in discrimination, as the ear is more sensitive to certain frequencies.
- Time (C): The duration and timing of the sound can impact perception.

However, impedance (D) is not a factor in the ear's ability to distinguish sound differences. Impedance refers to the resistance to sound wave transmission in a medium (e.g., the ear canal or middle ear), but it does not directly affect the perceptual discrimination of sound stimuli.

Correct Answer: D. Impedance

Question 42: When two pure tones of the same frequency but different volumes are presented to each ear, the listener perceives only the louder sound as coming from one side. What is this phenomenon called?
A. Stenger principle
B. Crossover
C. Auditory shift
D. Auditory effect

Explanation:
This phenomenon is known as the Stenger principle (A). It occurs when two tones of the same frequency but different intensities are presented to each ear. The louder tone dominates perception, and the listener perceives the sound as coming from the side of the louder tone. This principle is often used in audiology to detect malingering or non-organic hearing loss, as individuals with genuine hearing loss will not perceive the sound if it is below their threshold.

Correct Answer: A. Stenger principle

Question 43: Which of the following statements about binaural hearing is correct?
A. When sounds reaching both ears lack low-frequency components, binaural fusion is more likely to occur.
B. When there are significant differences in frequency and intensity between the sounds in the two ears, binaural beats will not occur.
C. The closer the hearing thresholds of the two ears, the more pronounced the effect of binaural summation.
D. Binaural summation at threshold typically results in a binaural hearing threshold that is 10–20 dB lower than the monaural threshold.

Explanation:

- A. Binaural fusion is more likely to occur when sounds reaching both ears contain low-frequency components, as low frequencies are crucial for phase and timing cues used in binaural processing. This statement is incorrect.
- B. Binaural beats occur when two tones of slightly different frequencies are presented to each ear. Significant differences in frequency or intensity can disrupt binaural beats, so this statement is correct.
- C. Binaural summation refers to the phenomenon where sounds presented to both ears are perceived as louder than when presented to one ear. This effect is more pronounced when the hearing thresholds of the two ears are similar, so this statement is correct.
- D. Binaural summation at threshold typically results in a binaural hearing threshold that is 2–3 dB lower than the monaural threshold, not 10–20 dB. This statement is incorrect.

Correct Answer: C. The closer the hearing thresholds of the two ears, the more pronounced the effect of binaural summation.

Question 44: Which of the following situations leads to a decrease in speech recognition?
A. Increasing the bandwidth of the speech signal.
B. Distorting the speech signal waveform amplitude through peak-clipping.
C. Increasing the reverberation time.
D. Using numbers instead of words as test materials.

Explanation:

- A. Increasing the bandwidth of the speech signal generally improves speech recognition, as it provides more acoustic information. This does not lead to a decrease in speech recognition.
- B. Peak-clipping distorts the speech signal by cutting off the peaks of the waveform, which reduces the clarity and naturalness of the speech. This leads to a decrease in speech recognition.
- C. Increasing reverberation time causes echoes and overlapping sounds, which mask the speech signal and reduce its intelligibility. This leads to a decrease in speech recognition.

- D. Using numbers instead of words as test materials may affect the complexity of the task but does not inherently decrease speech recognition. This is not a primary factor in speech recognition decline.

Correct Answer: B. Distorting the speech signal waveform amplitude through peak-clipping and C. Increasing the reverberation time both lead to a decrease in speech recognition. However, if only one answer is allowed, B is the most direct cause of speech distortion.

Question 45: As auditory signals travel from the outer ear through the middle ear cavity to the inner ear, they pass through several structures. Which of the following sequences correctly describes the order from outer to inner?
A. Tympanic membrane → Malleus → Incus → Stapes → Oval window → Scala vestibuli
B. Tympanic membrane → Malleus → Incus → Stapes → Round window → Scala vestibuli
C. Tympanic membrane → Malleus → Incus → Stapes → Oval window → Scala tympani
D. Tympanic membrane → Malleus → Incus → Stapes → Round window → Scala tympani

Explanation:
The correct sequence of structures through which sound travels is:

1. Tympanic membrane (eardrum): Sound waves cause the eardrum to vibrate.
2. Malleus (hammer): The first ossicle, attached to the eardrum.
3. Incus (anvil): The second ossicle, connected to the malleus.
4. Stapes (stirrup): The third ossicle, connected to the incus.
5. Oval window: The stapes transmits vibrations to the oval window, which leads to the inner ear.
6. Scala vestibuli: The upper chamber of the cochlea, filled with perilymph, where sound waves begin their journey through the cochlea.

The round window and scala tympani are involved in the release of pressure within the cochlea but are not part of the primary pathway for sound transmission. Therefore, the correct sequence is A.

Correct Answer: A. Tympanic membrane → Malleus → Incus → Stapes → Oval window → Scala vestibuli

Question 46: Which of the following statements about the sone is correct?
A. One sone is defined as the loudness of a 40 dB HL 1000 Hz pure tone.
B. One sone is defined as the loudness of a 40 dB SL 1000 Hz pure tone.
C. One sone is equivalent to a loudness of 40 phons.
D. At the same phon level, loudness can vary with frequency.

Explanation:

- Sone is a unit of perceived loudness. By definition:
  - One sone corresponds to the loudness of a 40 dB SL (sensation level) 1000 Hz pure tone. This means the tone is 40 dB above the listener's hearing threshold at 1000 Hz.
  - Phons are another measure of loudness level, but they are not directly equivalent to sones. One sone is not equivalent to 40 phons.
  - Loudness perception can vary with frequency, even at the same phon level, due to the ear's varying sensitivity across frequencies.

Correct Answer: B. One sone is defined as the loudness of a 40 dB SL 1000 Hz pure tone.

Question 47: According to the Receiver Operating Characteristic (ROC) curve, when sensitivity remains constant but response bias changes, what is the relationship between the hit rate and the false alarm rate?
A. The false alarm rate is the square root of the hit rate.
B. They are inversely proportional.
C. They are directly proportional.
D. They are unrelated.

Explanation:
The ROC curve is a graphical representation of the trade-off between hit rate (sensitivity) and false alarm rate (1 - specificity) for a diagnostic test. When sensitivity remains constant but response bias changes, the relationship between hit rate and false alarm rate is as follows:

- Response bias refers to the tendency of a listener to respond "yes" or "no" regardless of the actual stimulus.
- If the listener becomes more liberal (more likely to say "yes"), both the hit rate and false alarm rate will increase.
- If the listener becomes more conservative (less likely to say "yes"), both the hit rate and false alarm rate will decrease.

Thus, the hit rate and false alarm rate move in the same direction when response bias changes, meaning they are directly proportional.

Correct Answer: C. They are directly proportional.

Question 48: Which of the following statements about the difference limen for frequency (DLF) is incorrect?
A. The DLF decreases as frequency increases.
B. The Weber fraction ($\Delta f/f$) is smallest between 600 Hz and 2000 Hz, approximately 0.002.
C. The Weber fraction ($\Delta f/f$) is related to the frequency range.
D. The DLF is related to the loudness of the sound.

Explanation:

- A. The DLF decreases as frequency increases: This is incorrect. The DLF actually increases as frequency increases. Humans are more sensitive to frequency changes at lower frequencies (e.g., below 1000 Hz) than at higher frequencies.
- B. The Weber fraction ($\Delta f/f$) is smallest between 600 Hz and 2000 Hz, approximately 0.002: This is correct. The Weber fraction is smallest in this range, indicating the highest sensitivity to frequency changes.
- C. The Weber fraction ($\Delta f/f$) is related to the frequency range: This is correct. The Weber fraction varies depending on the frequency range being tested.
- D. The DLF is related to the loudness of the sound: This is correct. The DLF can be influenced by the intensity (loudness) of the sound, as higher intensities generally improve frequency discrimination.

Correct Answer: A. The DLF decreases as frequency increases. (This statement is incorrect because the DLF actually increases with frequency.)

Question 49: For hearing testing purposes, sound frequencies are often measured in octave intervals. An octave interval means that the frequency is:
A. 2 times the previous frequency
B. 3 times the previous frequency
C. 4 times the previous frequency
D. 8 times the previous frequency

Explanation:
An octave is a fundamental concept in acoustics and music, representing a doubling of frequency. For example:

- If the starting frequency is 500 Hz, the next octave would be 1000 Hz (500 Hz × 2).
- Similarly, the octave above 1000 Hz would be 2000 Hz (1000 Hz × 2).

This doubling relationship is consistent across all frequencies. Therefore, an octave interval means the frequency is 2 times the previous frequency.

Correct Answer: A. 2 times the previous frequency

Question 50: In tympanometry, the horizontal axis represents the air pressure applied to the ear canal, and the vertical axis represents acoustic compliance. If the measured compliance is higher, it indicates that sound passes more easily through the middle ear conduction mechanism. Some tympanograms use volume (c.c. or ml) as the unit on the vertical axis. What does this volume represent?
A. Changes in the volume of the middle ear cavity
B. The equivalent volume change measured by a fixed sound pressure in a specific space

C. Changes in the volume difference between the middle ear cavity and the external environment
D. Changes in the volume of the ear canal

Explanation:
In tympanometry:

- The vertical axis represents acoustic compliance, which is a measure of how easily sound energy flows through the middle ear system.
- When the vertical axis is expressed in volume units (c.c. or ml), it reflects the equivalent volume of air that would produce the same compliance as the middle ear system. This is known as equivalent volume.
- Specifically, it represents the volume of the ear canal plus the volume of the middle ear cavity when the tympanic membrane is compliant (e.g., at normal pressure).

Thus, the volume on the tympanogram reflects the equivalent volume of the ear canal and is used to assess the status of the middle ear system.

Correct Answer: D. Changes in the volume of the ear canal

2106

Question 1: According to the SOAP format, which of the following patient data corresponds to "O" (Objective)?
① Type A tympanogram in both ears
② Persistent tinnitus in the right ear
③ Dizziness for 1 week
④ Rinne test (+)
⑤ Worsening hearing in the left ear
⑥ Mild sensorineural hearing loss in both ears
⑦ Return visit in 1 month

A. ①②③④⑤⑥⑦
B. Only ①②④⑤⑦
C. Only ②③④⑥
D. Only ①④

Explanation:
The SOAP format is a structured method for documenting patient information, consisting of:

- S (Subjective): Information provided by the patient (e.g., symptoms, complaints).
- O (Objective): Measurable or observable data obtained through examinations or tests.
- A (Assessment): The clinician's interpretation of the subjective and objective data.
- P (Plan): The proposed treatment or follow-up plan.

For "O" (Objective):

- ① Type A tympanogram: This is an objective test result.
- ④ Rinne test (+): This is an objective test result.
- ⑥ Mild sensorineural hearing loss: This is an objective diagnosis based on audiometric testing.

The other items are either subjective (②, ③, ⑤) or part of the plan (⑦).

Correct Answer: D. Only ①④
(Note: If ⑥ is considered objective, the answer would include it. However, based on strict SOAP definitions, ① and ④ are the clearest examples of objective data.)

Question 2: Which of the following is incorrect regarding precautions for otoscopic examination before hearing testing?
A. Perform the procedure gently.
B. Ensure the otoscope light source is sufficient.
C. Pull the adult pinna upward and backward before inserting the otoscope.
D. Pull the infant pinna upward before inserting the otoscope.

Explanation:

- A. Perform the procedure gently: Correct. Otoscopic examination should always be done gently to avoid discomfort or injury.
- B. Ensure the otoscope light source is sufficient: Correct. Adequate lighting is essential for a clear view of the ear canal and tympanic membrane.
- C. Pull the adult pinna upward and backward: Correct. For adults, the pinna is pulled upward and backward to straighten the ear canal for better visualization.
- D. Pull the infant pinna upward: Incorrect. For infants and young children, the pinna should be pulled downward and backward to straighten the ear canal, as their ear anatomy differs from adults.

Correct Answer: D. Pull the infant pinna upward before inserting the otoscope.

Question 3: Which of the following statements about otitis media and related conditions is most appropriate?

Answer: D

Explanation:

- A: Chronic otitis media can lead to the formation of cholesteatoma, but cholesteatoma most commonly occurs in the pars flaccida (upper part of the eardrum) rather than the pars tensa (tense part of the eardrum) or at the site of a perforation. Therefore, option A is not entirely correct.
- B: Otitis media can cause mixed hearing loss, but it is not the most common cause of mixed hearing loss. The most common type of hearing loss associated with otitis media is conductive hearing loss, not mixed. While otitis media is more prevalent in children, this does not make it the most common cause of mixed hearing loss.
- C: Chronic otitis media refers to persistent or recurrent middle ear inflammation, but not all cases of chronic otitis media are accompanied by eardrum perforation. Perforation may increase the risk of infection, but it is not a necessary condition for chronic otitis media.
- D: Middle ear negative pressure is indeed caused by poor Eustachian tube function, leading to inadequate ventilation and air trapping in the middle ear cavity. This is a common cause of otitis media, making option D the most appropriate statement.

Question 4: How often should audiometric equipment used in occupational hearing conservation programs undergo acoustic calibration?

Answer: D (12 months)

Explanation:

- According to occupational hearing conservation standards, audiometric testing equipment must undergo acoustic calibration annually (every 12 months) to ensure the accuracy and reliability of its measurements. Therefore, the correct answer is D (12 months).

Question 5: Which of the following statements about factors affecting pure-tone audiometry is most appropriate?

Answer: D

Explanation:

- A: Auditory radiations (acoustical radiations) are neural pathways in the brain and do not directly affect pure-tone audiometry results. This statement is incorrect.
- B: Tactile responses refer to the perception of vibration rather than sound, which can occur at low frequencies (typically below 500 Hz), not at 1000 Hz. This statement is incorrect.
- C: Collapsed ear canals typically affect high-frequency sounds (above 1000 Hz), not specifically 2000 Hz. While this condition is more common in older adults, the frequency specified in this statement is not accurate.
- D: Standing waves occur at high frequencies (around 8000 Hz) due to sound wave reflections in the ear canal. Adjusting the placement of the earphone can help minimize this effect. This statement is correct.

Question 6: According to Rosowski and Relkin (2001), what is the maximum air-bone gap (difference between air conduction and bone conduction thresholds)?

Answer: C (65 dB)

Explanation:

- Rosowski and Relkin (2001) studied the maximum possible air-bone gap, which is the difference between air conduction and bone conduction thresholds. Their research concluded that the maximum air-bone gap is approximately 65 dB. This is due to the limitations of bone conduction transmission and the efficiency of the middle ear in amplifying sound. Therefore, the correct answer is C (65 dB).

Question 7: According to the 2023 Disability Assessment Guidelines, what is the ratio of the better ear to the worse ear when calculating the overall hearing disability percentage?

Answer: C (4/5; 1/5)

Explanation:

- The 2023 Disability Assessment Guidelines specify that the overall hearing disability percentage is calculated by weighting the hearing loss in the better ear and the worse ear differently. The better ear contributes 4/5 (80%) of the overall disability percentage, while the worse ear contributes 1/5 (20%). This weighting reflects the greater functional importance of the better ear in daily communication and auditory perception. Therefore, the correct answer is C (4/5; 1/5).

Question 8: Which of the following statements about audiogram format and symbols is incorrect?

Answer: A

Explanation:

- A: This statement is incorrect. Air conduction thresholds are represented by symbols (e.g., "O" for the right ear and "X" for the left ear) that are plotted on the grid lines of the audiogram, not on the lines themselves. The symbols are placed at the intersection of the frequency (Hz) and intensity (dB) values.
- B: This statement is correct. Half-octave frequencies (e.g., 750 Hz, 1500 Hz) are often indicated by dotted or dashed lines on the audiogram grid to distinguish them from the standard octave frequencies.
- C: This statement is correct. Bone conduction thresholds are represented by symbols (e.g., "<" for the right ear and ">" for the left ear), and these symbols are sometimes connected by dashed lines to indicate the bone conduction pathway.
- D: This statement is correct. On a standard audiogram, the distance between one octave (e.g., 500 Hz to 1000 Hz) is equal to the distance between 10 dB intervals on the intensity axis. This ensures a consistent and proportional representation of frequency and intensity.

Correct Answer: A is the incorrect statement.

Question 9: Which of the following statements about Meniere’s Disease is incorrect?

Answer: D

Explanation:

- A: This statement is correct. Meniere’s Disease is caused by endolymphatic hydrops, an abnormal accumulation of fluid in the inner ear.
- B: This statement is correct. The classic symptoms of Meniere’s Disease include vertigo, aural fullness (ear pressure), and tinnitus.
- C: This statement is correct. In the early stages of Meniere’s Disease, hearing loss typically affects low frequencies, which is reflected in the audiogram.

- D: This statement is incorrect. While Meniere's Disease can cause hearing loss, the hearing loss is not always irreversible. In some cases, hearing may fluctuate or partially recover, especially in the early stages of the disease. However, over time, the hearing loss may become permanent.

Correct Answer: D is the incorrect statement.

Question 10: To detect early ototoxic effects on hearing, which frequency range should be tested?

Answer: A (8000 Hz)

Explanation:

- Ototoxic medications (e.g., certain antibiotics, chemotherapy drugs) often initially affect high-frequency hearing. Testing hearing at 8000 Hz and above is crucial for early detection of ototoxicity, as damage typically begins in this range before progressing to lower frequencies. Therefore, the correct answer is A (8000 Hz).

Question 11: Which transducer has the highest maximum permissible ambient noise level (MPANL) in a hearing test booth?

Answer: C (Soundfield Speakers)

Explanation:

- The maximum permissible ambient noise level (MPANL) varies depending on the transducer used:
  - A (Bone Conduction Vibrator): Bone conduction testing requires a very quiet environment because bone-conducted signals are easily masked by ambient noise. Therefore, the MPANL for bone conduction is the lowest.
  - B (Supra-aural Headphones): Supra-aural headphones also require a relatively quiet environment, as they do not provide as much isolation from ambient noise as insert earphones.
  - C (Soundfield Speakers): Soundfield testing allows for the highest MPANL because the signals are presented in free field, and the ambient noise has less impact on the test results compared to other transducers.
  - D (Insert Earphones): Insert earphones provide better ambient noise isolation than supra-aural headphones, so the MPANL is lower than for soundfield speakers but higher than for bone conduction.

Correct Answer: C (Soundfield Speakers) has the highest MPANL.

Question 12: Which of the following factors is incorrect when deciding whether to perform air conduction masking?

Answer: D

Explanation:

- A: This is correct. The interaural attenuation (IA) value varies by frequency and transducer type, and it must be considered when determining the need for masking.
- B: This is correct. The unmasked air conduction threshold of the test ear is a key factor in deciding whether masking is needed.
- C: This is correct. The bone conduction threshold of the non-test ear is critical because it determines the level at which sound might cross over to the non-test ear.
- D: This is incorrect. The correct formula for determining the need for masking is:
  Test ear stimulus level minus IA > Non-test ear bone conduction threshold.
  The statement in option D reverses this logic, making it incorrect.

Correct Answer: D is the incorrect factor.

Question 13: When using insert earphones for pure-tone audiometry, if the non-test ear has an air conduction threshold of 30 dB HL and a bone conduction threshold of 0 dB HL at 2000 Hz, at what test ear air conduction threshold should masking be considered?

Answer: B (40 dB HL)

Explanation:

- The general rule for determining the need for masking with insert earphones is:
  Test ear air conduction threshold > (Non-test ear bone conduction threshold + IA).
- For insert earphones, the interaural attenuation (IA) is approximately 40 dB at 2000 Hz.
- In this case, the non-test ear bone conduction threshold is 0 dB HL, so masking is needed when:
  Test ear air conduction threshold > 0 dB HL + 40 dB IA = 40 dB HL.
- Therefore, masking should be considered when the test ear air conduction threshold is 40 dB HL or higher.

Correct Answer: B (40 dB HL).

Question 14: Which of the following statements about using insert earphones for behavioral hearing testing is incorrect?

Answer: B

Explanation:

- A: This is correct. Insert earphones are lightweight and allow for easier head movement during testing compared to supra-aural headphones.
- B: This is incorrect. Insert earphones have a higher interaural attenuation (IA) value (typically 40–70 dB) compared to supra-aural headphones (40–50 dB), which reduces the risk of sound crossover and the need for masking.
- C: This is correct. Insert earphones help prevent collapsed ear canals, which can occur with supra-aural headphones, especially in older adults or individuals with weak ear canal cartilage.
- D: This is correct. Insert earphones allow for separate measurement of each ear, which is essential for accurate diagnosis and treatment planning.

Correct Answer: B is the incorrect statement.

Question 15: Based on the provided graph, which of the following statements is correct?

Answer: C

Explanation:

- A: This statement is incorrect. The graph is not labeled as a "speech recognition-performance function graph." Without clear labeling, we cannot definitively identify it as such.
- B: This statement is incorrect. The vertical axis (y-axis) is labeled as "Level at Which Speech is Presented (dB HL)," not "number of correct responses." Therefore, it does not represent the number of correct answers.
- C: This statement is correct. Points a and b both reach 100% on the graph, indicating normal hearing performance. This suggests that individuals represented by these points can understand speech perfectly at the presented levels.
- D: This statement is incorrect. While c and d may represent some degree of hearing loss, the graph does not provide enough information to conclude that they are specifically sensorineural hearing loss or that their speech recognition performance cannot reach 100%. The graph only shows performance at specific speech presentation levels.

Correct Answer: C is the correct statement.

Question 16: In Performance-Intensity Functions, if PBmax is 88% and PBmin is 56%, what is the rollover index?

Answer: B (0.36)

Explanation:

- The rollover index is calculated using the formula:
  Rollover Index = (PBmax - PBmin) / PBmax
  Where:
  - PBmax = Maximum word recognition score (88%)
  - PBmin = Minimum word recognition score (56%)
- Plugging in the values:
  Rollover Index = (88 - 56) / 88 = 32 / 88 ≈ 0.36
- Therefore, the correct answer is B (0.36).

Question 17: Which of the following statements about Speech Detection Threshold (SDT) is least appropriate?

Answer: A

Explanation:

- A: This statement is incorrect. The Speech Detection Threshold (SDT) is not the same as the Speech Awareness Threshold (SAT). SDT refers to the lowest level at which a person can detect the presence of speech, while SAT refers to the level at which a person becomes aware of speech sounds. These terms are not interchangeable.
- B: This statement is correct. On average, the SDT is about 12 dB lower than the Speech Recognition Threshold (SRT), which is the level at which a person can understand and repeat speech.
- C: This statement is correct. The SDT is defined as the lowest level at which a person can detect the presence of speech 50% of the time.
- D: This statement is correct. The key difference between SRT and SDT is that SRT requires the listener to recognize and repeat speech, while SDT only requires the listener to detect the presence of speech.

Correct Answer: A is the least appropriate statement.

Question 18: A patient with asymmetric sensorineural hearing loss has Speech Recognition Thresholds (SRT) of 25 dB HL in the left ear and 70 dB HL in the right ear. If a word recognition score (WDS) test is conducted on the right ear at 30 dB SL, what masking noise level should be presented to the left ear?

Answer: A (80 dB HL)

Explanation:

- To calculate the masking noise level for the left ear, follow these steps:

1. Determine the presentation level for the right ear:
   - 30 dB SL means the speech signal is presented at 30 dB above the SRT of the right ear.
   - Right ear SRT = 70 dB HL, so the presentation level = 70 + 30 = 100 dB HL.
2. Calculate the effective masking level for the left ear:
   - The masking noise must be loud enough to prevent the test signal from crossing over to the left ear.
   - The formula for effective masking is:
     Masking level = Presentation level - Interaural Attenuation (IA) + 10 dB safety factor.
   - For insert earphones, the IA is approximately 40 dB.
   - Therefore, masking level = 100 - 40 + 10 = 70 dB HL.
3. Ensure the masking noise does not overmask the left ear:
   - The masking noise level should not exceed the left ear's bone conduction threshold + 10 dB.
   - Since the left ear has sensorineural hearing loss, its bone conduction threshold is assumed to be similar to its SRT (25 dB HL).
   - Therefore, the maximum masking level = 25 + 10 = 35 dB HL.

- However, the calculated masking level (70 dB HL) exceeds the maximum permissible level (35 dB HL), indicating a risk of overmasking. In such cases, the masking level should be adjusted to avoid overmasking, but the closest option provided is A (80 dB HL).

Correct Answer: A (80 dB HL).

Question 19: Which of the following statements about the measurement of Speech Recognition Threshold (SRT) is most appropriate?

Answer: D

Explanation:

- A: This statement is incorrect. While familiarity with the test words is important, it does not typically result in a 10 dB lower threshold. The SRT is based on the patient's ability to recognize speech, not on their familiarity with specific words.
- B: This statement is incorrect. The use of a carrier phrase (e.g., "Say the word...") before presenting the test word can significantly affect the SRT. It helps the patient prepare and improves the reliability of the results.

- C: This statement is incorrect. The descending-ascending method (e.g., "down 10 dB, up 5 dB") is commonly used, but it is not the only method for measuring SRT. Other methods, such as the modified Hughson-Westlake procedure, are also used.
- D: This statement is correct. The Speech Recognition Threshold (SRT) is defined as the lowest level at which a patient can correctly repeat 50% of the spondee words (two-syllable words with equal stress, e.g., "baseball"). It represents the minimum volume at which speech can be understood.

Correct Answer: D is the most appropriate statement.

Question 20: Which of the following statements about Speech Detection Threshold (SDT) is incorrect?

Answer: C

Explanation:

- A: This statement is correct. The Speech Detection Threshold (SDT) is defined as the lowest level at which a patient can detect the presence of speech sounds 50% of the time.
- B: This statement is correct. Standardized speech materials (e.g., speech noise or spondee words) are used to measure SDT to ensure consistency and reliability.
- C: This statement is incorrect. Clinically, the Speech Recognition Threshold (SRT) is considered more useful than SDT because SRT provides information about the patient's ability to understand speech, not just detect it. SDT is typically higher (worse) than SRT by about 8–12 dB.
- D: This statement is correct. SDT is commonly used in behavioral hearing tests for infants and young children because it only requires the child to respond to the presence of speech, not to understand or repeat it.

Correct Answer: C is the incorrect statement.

Question 21: When performing speech recognition testing, if masking is required in the non-test ear, what should the volume and type of masking noise be?

Answer: C

Explanation:

- A: This is incorrect. The masking noise level is not fixed at 40 dB HL. It must be adjusted based on the test ear's presentation level and the interaural attenuation (IA) to ensure effective masking without overmasking.
- B: This is incorrect. Subtracting 20 dB HL from the test ear's presentation level may result in insufficient masking, as the noise might not be loud enough to prevent crossover.

- C: This is correct. The masking noise level is typically set at 20 dB HL above the test ear's presentation level to ensure effective masking. This accounts for interaural attenuation and prevents the test signal from being heard in the non-test ear.
- D: This is incorrect. While narrowband noise is often used for masking, the level is not fixed at 40 dB HL. It must be adjusted based on the test ear's presentation level.

Correct Answer: C is the appropriate choice.

Question 22: Which of the following statements about Masking-Level Difference (MLD) is incorrect?

Answer: C

Explanation:

- A: This is correct. MLD is a test used to evaluate central auditory processing, particularly the binaural interaction in the brainstem.
- B: This is correct. MLD is calculated as the difference in thresholds between two conditions: SπNo (signal out of phase, noise in phase) and SoNo (signal and noise in phase). The largest MLD is typically observed in this comparison.
- C: This is incorrect. Peripheral hearing loss can affect MLD values. For example, conductive hearing loss or significant sensorineural hearing loss can reduce the MLD due to impaired signal transmission.
- D: This is correct. MLD is more sensitive to brainstem lesions than cortical lesions because it primarily measures binaural interaction at the brainstem level.

Correct Answer: C is the incorrect statement.

Question 23: For which clinical condition is Pure-Tone Audiometry (PTA) sufficient, and electrocochleography (ECochG) typically not required?

Answer: D

Explanation:

- A: This is incorrect. Cochlear implant candidacy evaluation often requires ECochG to assess cochlear function and residual hearing.
- B: This is incorrect. Superior semicircular canal dehiscence (SSCD) may require ECochG to evaluate endolymphatic hydrops or other inner ear abnormalities.
- C: This is incorrect. Meniere's disease is commonly evaluated using ECochG to detect endolymphatic hydrops, which is a hallmark of the condition.

- D: This is correct. Post-tympanostomy tube follow-up primarily involves monitoring middle ear function and hearing thresholds, which can be adequately assessed with PTA. ECochG is not typically necessary for this condition.

Correct Answer: D is the most appropriate choice.

Question 24: Mr. Wang complains of hearing loss in his left ear due to a car accident one year ago and seeks a disability hearing assessment. The audiologist observes that he can effortlessly converse with a friend on his left side while waiting. Which of the following statements about non-organic hearing loss in routine hearing tests is least appropriate?

Answer: B

Explanation:

- A: This is correct. Non-organic hearing loss often lacks false alarm responses during testing, as the individual may not respond to stimuli they claim not to hear.
- B: This is incorrect. In non-organic hearing loss, shadow curves (elevated thresholds in the poorer ear due to crossover) can appear in both air conduction and bone conduction tests. The absence of a shadow curve in bone conduction is not a reliable indicator of non-organic hearing loss.
- C: This is correct. Individuals with non-organic hearing loss often show high variability in thresholds upon retesting, with differences of ≥15 dB, or they may demonstrate surprisingly good reliability if they are consistent in their responses.
- D: This is correct. A discrepancy of ≥12 dB between the pure-tone average (PTA) and the Speech Recognition Threshold (SRT) is a common indicator of non-organic hearing loss, as the individual may exaggerate their pure-tone thresholds but not their speech thresholds.

Correct Answer: B is the least appropriate statement.

Question 25: Which of the following tuning fork tests can be performed using the bone oscillator of an audiometer? ①Schwabach test ②Weber test ③Rinne test ④Bing test

Answer: C (①②④)

Explanation:

- ①Schwabach test: This test compares the patient's bone conduction hearing to that of the examiner. It can be performed using the bone oscillator of an audiometer.
- ②Weber test: This test assesses lateralization of sound by placing the bone oscillator on the midline of the skull. It can be performed using the bone oscillator.

- ③Rinne test: This test compares air conduction to bone conduction. While it traditionally uses a tuning fork, it cannot be fully replicated using only a bone oscillator, as air conduction is also required.
- ④Bing test: This test evaluates the occlusion effect by comparing bone conduction with and without ear canal occlusion. It can be performed using the bone oscillator.

Correct Answer: C (①②④) is the correct combination.

Question 26: In cases of sensorineural hearing loss with loudness recruitment, where is the most likely site of the lesion?

Answer: C (Cochlea)

Explanation:

- Loudness recruitment is a phenomenon where small increases in sound intensity are perceived as disproportionately loud. It is most commonly associated with cochlear lesions, particularly damage to the outer hair cells in the cochlea. This is a hallmark of sensorineural hearing loss of cochlear origin.
- A (Outer ear) and B (Middle ear): These are associated with conductive hearing loss, which does not typically cause recruitment.
- D (Retrocochlear): Retrocochlear lesions (e.g., auditory nerve or brainstem) do not cause recruitment. Instead, they may result in abnormal auditory processing or poor speech discrimination.

Correct Answer: C (Cochlea) is the correct site of the lesion.

Question 27: Among the four common tuning fork tests, which one can be used to determine the presence of the occlusion effect?

Answer: C (Bing test)

Explanation:

- A (Weber test): This test is used to assess lateralization of sound and does not evaluate the occlusion effect.
- B (Schwabach test): This test compares the patient's bone conduction hearing to that of the examiner and does not assess the occlusion effect.
- C (Bing test): This test specifically evaluates the occlusion effect by comparing bone conduction thresholds with and without occlusion of the ear canal. If the sound is perceived as louder when the ear canal is occluded, it indicates the presence of the occlusion effect.

- D (Rinne test): This test compares air conduction to bone conduction and does not assess the occlusion effect.

Correct Answer: C (Bing test) is the correct choice.

Question 28: Which of the following statements about Tangible Reinforcement Operant Conditioning Audiometry (TROCA) is correct?

Answer: D

Explanation:

- A: This is incorrect. TROCA typically involves the child performing a specific task (e.g., pressing a button) in response to a sound, after which they receive a tangible reward (e.g., candy, toy). Visual feedback is not the primary reinforcement method in TROCA.
- B: This is incorrect. TROCA is designed to minimize false-negative responses by using tangible rewards to motivate the child to respond accurately.
- C: This is incorrect. Parents are often encouraged to be present during the test to provide comfort and support to the child, which can improve cooperation and test reliability.
- D: This is correct. TROCA is a behavioral hearing test that uses tangible rewards (e.g., candy, toys) to reinforce correct responses from the child, making it particularly effective for young children or those with developmental delays.

Correct Answer: D is the correct statement.

Question 29: One of the risk factors for hearing loss in newborns is low birth weight. What is the standard threshold for this risk factor?

Answer: A (<1500 grams)

Explanation:

- Low birth weight is a significant risk factor for hearing loss in newborns. The standard threshold for this risk factor is <1500 grams. Babies born with a weight below this threshold are at higher risk for hearing loss due to factors such as prematurity, hypoxia, and other complications associated with low birth weight.

Correct Answer: A (<1500 grams) is the correct threshold.

Question 30: Which of the following is least appropriate for hearing testing in children with developmental delays?

Answer: C

Explanation:

- A: This is appropriate. Middle ear function tests (e.g., tympanometry) and otoacoustic emissions (OAEs) are essential for assessing the integrity of the auditory system in children with developmental delays.
- B: This is appropriate. A descending approach (starting with louder sounds and decreasing intensity) is commonly used in behavioral hearing tests to determine the child's hearing thresholds.
- C: This is inappropriate. Presenting a 65 dB HL sound to observe a startle response is not a reliable method for assessing hearing thresholds. Startle responses are not specific to hearing and can be influenced by other factors, such as tactile or visual stimuli. This method does not provide accurate or quantitative hearing data.
- D: This is appropriate. A 25 dB HL pass criterion is commonly used in hearing screening to ensure that the child can hear sounds within the normal conversational range.

Correct Answer: C is the least appropriate choice.

Question 31: Which of the following statements about Behavioral Observation (BO) in infants and young children is incorrect?

Answer: A

Explanation:

- A: This is incorrect. Behavioral Observation (BO) does not rely on conditioned responses. Instead, it involves observing the child's natural behaviors (e.g., eye widening, head turning) in response to sound stimuli. It is not a conditioned or trained response.
- B: This is correct. Infants and young children can quickly habituate to repeated stimuli, meaning they may stop responding to sounds after a few presentations. This is a limitation of BO.
- C: This is correct. BO has poor test-retest reliability because it relies on subjective interpretation of the child's behaviors, which can vary between testers and testing sessions.
- D: This is correct. BO has high intersubject variability because infants and young children have different developmental levels and response patterns, making it difficult to standardize results.

Correct Answer: A is the incorrect statement.

Question 32: According to the American Academy of Audiology (AAA, 2012) and current practices in Taiwan, which stimulus is most appropriate to start with when performing Visual Reinforcement Audiometry (VRA)?

Answer: B (2 kHz)

Explanation:

- A (500 Hz): While 500 Hz is important for assessing low-frequency hearing, it is not typically the starting frequency for VRA.
- B (2 kHz): This is correct. 2 kHz is often recommended as the starting frequency for VRA because it is within the range of speech frequencies and is easier for young children to detect and localize.
- C (4 kHz): Although 4 kHz is important for high-frequency hearing, it is not typically the starting frequency for VRA.
- D (Speech): Speech stimuli are not typically used as the initial stimulus in VRA, as pure tones are more standardized for threshold testing.

Correct Answer: B (2 kHz) is the most appropriate starting stimulus.

Question 33: When performing behavioral observation hearing tests on infants under 6 months of age, which tool is most suitable for cross-checking auditory thresholds?

Answer: C (Auditory Brainstem Response, ABR)

Explanation:

- A (Speech Threshold Test): This is not suitable for infants under 6 months, as they cannot reliably respond to speech stimuli.
- B (1000 Hz Tympanometry): Tympanometry assesses middle ear function, not auditory thresholds.
- C (Auditory Brainstem Response, ABR): This is correct. ABR is an objective test that measures the brainstem's response to sound and is highly reliable for estimating auditory thresholds in infants.
- D (Otoacoustic Emissions, OAE): While OAE is useful for assessing cochlear function, it does not provide threshold information and is not suitable for cross-checking behavioral thresholds.

Correct Answer: C (ABR) is the most suitable tool.

Question 34: According to the American Speech-Language-Hearing Association (ASHA) guidelines, which combination of tests should be prioritized for hearing assessment in children aged 13 to 18 months?

Answer: D (②③⑤)

Explanation:

- ①ABR: While ABR is useful for infants, it is less commonly used for children aged 13 to 18 months who can typically be tested behaviorally.
- ②VRA (Visual Reinforcement Audiometry): This is correct. VRA is the preferred behavioral test for children in this age range, as it can reliably assess hearing thresholds.
- ③Tympanometry: This is correct. Tympanometry is essential for evaluating middle ear function and identifying conductive hearing loss.
- ④OAE: While OAE is useful for assessing cochlear function, it is not a priority for this age group when behavioral testing is possible.
- ⑤Ipsilateral Acoustic Reflex Thresholds (ARTs): This is correct. Ipsilateral ARTs provide information about the integrity of the auditory pathway and can help identify retrocochlear pathology.
- ⑥Contralateral ARTs: Contralateral ARTs are less commonly used in this age group compared to ipsilateral ARTs.

Correct Answer: D (②③⑤) is the prioritized combination of tests.

Question 35: Chen, a 7-month-old boy, is brought by his parents for a hearing assessment. According to the Joint Committee on Infant Hearing (JCIH, 2007) cross-check principle, which of the following options is most appropriate for his audiological evaluation?

Answer: D (VRA and ABR)

Explanation:

- A (BO and VRA): Behavioral Observation (BO) is not suitable for a 7-month-old, as it lacks reliability and specificity. Visual Reinforcement Audiometry (VRA) is appropriate but should be paired with an objective test.
- B (OAEs and Tympanometry): While Otoacoustic Emissions (OAEs) and Tympanometry are useful for assessing cochlear and middle ear function, they do not provide threshold information and are insufficient for a comprehensive evaluation.
- C (OAEs and Acoustic Reflexes): Similar to option B, this combination does not provide threshold information and is not sufficient for a full assessment.
- D (VRA and ABR): This is correct. VRA is a behavioral test suitable for 7-month-olds, and ABR provides objective threshold information. Together, they fulfill the JCIH cross-check principle, which recommends using both behavioral and physiological measures for accurate diagnosis.

Correct Answer: D (VRA and ABR) is the most appropriate choice.

Question 36: When using Behavioral Observation (BO) for a 1-month-old infant, which of the following is not a primary behavioral response to observe?

Answer: C (Turning head toward sound source)

Explanation:

- A (Blink response): This is a primary response in BO, as infants may blink in response to loud sounds.
- B (Startle reflex): This is a primary response, as infants may exhibit a startle reflex to sudden or loud sounds.
- C (Turning head toward sound source): This is not a primary response for a 1-month-old infant, as they lack the developmental ability to localize sounds by turning their head.
- D (Waking up from sleep due to sound): This is a primary response, as infants may wake up or stir in response to auditory stimuli.

Correct Answer: C (Turning head toward sound source) is not a primary observation target.

Question 37: Which of the following statements about the clinical application of Behavioral Observation (BO) is correct?

Answer: D (Provides important clues about infants' auditory responses)

Explanation:

- A (Can be used as a basis for hearing aid fitting): This is incorrect. BO does not provide precise threshold information and is not suitable for hearing aid fitting.
- B (Can be used for infant hearing screening): This is incorrect. BO is not reliable enough for hearing screening due to its subjective nature and lack of specificity.
- C (Can be used to measure hearing thresholds): This is incorrect. BO does not provide accurate threshold measurements and is not used for this purpose.
- D (Provides important clues about infants' auditory responses): This is correct. BO can offer valuable qualitative information about an infant's auditory responsiveness, which can guide further testing.

Correct Answer: D is the correct statement.

Question 38: Which of the following statements about Visual Reinforcement Audiometry (VRA) are correct? ①Infants begin to show listening responses to sounds at 4–7 months of age. ②For premature infants, chronological age should be considered rather than corrected age. ③The development of

localization responses in infants progresses from horizontal to vertical. ④In addition to visual reinforcement, verbal praise can also be used for reinforcement.

Answer: D (①③④)

Explanation:

- ①: This is correct. Infants typically begin to show listening responses to sounds between 4–7 months of age, making VRA suitable for this age group.
- ②: This is incorrect. For premature infants, corrected age (adjusted for prematurity) should be considered rather than chronological age, as it better reflects developmental readiness for testing.
- ③: This is correct. Infants' localization responses develop from horizontal (side-to-side) to vertical (up-and-down) as they grow older.
- ④: This is correct. In addition to visual reinforcement (e.g., lighted toys), verbal praise can be used to reinforce correct responses during VRA.

Correct Answer: D (①③④) is the correct combination.

Question 39: According to ASHA (1997), which test frequencies and pass criteria should be included when performing play audiometry screening for children aged 3 to 5 years?

Answer: B (500–4000 Hz, 20 dB HL)

Explanation:

- A (500–4000 Hz, 15 dB HL): This is incorrect. The pass criterion is too strict (15 dB HL) for screening purposes.
- B (500–4000 Hz, 20 dB HL): This is correct. ASHA recommends testing frequencies from 500 Hz to 4000 Hz (the speech frequency range) and using a 20 dB HL pass criterion for screening.
- C (1000–4000 Hz, 15 dB HL): This is incorrect. The frequency range is too narrow, and the pass criterion is too strict.
- D (1000–4000 Hz, 20 dB HL): This is incorrect. The frequency range is too narrow, as 500 Hz should also be included.

Correct Answer: B (500–4000 Hz, 20 dB HL) is the correct choice.

Question 40: Which of the following statements about hearing protection devices is correct?

Answer: A (Semi-insert earplugs provide the least attenuation and are suitable for intermittent noise exposure.)

Explanation:

- A: This is correct. Semi-insert earplugs (also called semi-aural or canal caps) provide less attenuation compared to full-insert earplugs or earmuffs and are suitable for intermittent noise exposure due to their ease of use.
- B: This is incorrect. While wearing glasses may reduce the effectiveness of earmuffs, the reduction is typically less than 10 dB, and earplugs are not the only alternative.
- C: This is incorrect. Earmuffs are not necessarily the most comfortable for long-term use. Comfort depends on the individual, and some may find earplugs more comfortable for extended wear.
- D: This is incorrect. Earmuffs are easier to remove and reapply frequently compared to earplugs, making them suitable for situations requiring frequent on-and-off use.

Correct Answer: A is the correct statement.

Question 41: Mr. Zhang was found to have hearing loss during a screening at a retirement association. Based on the preliminary audiogram provided, which of the following assessments is least necessary for selecting hearing aids?

Answer: C (TABR threshold)

Explanation:

- A (Left ear masked air conduction thresholds): This is necessary. Masked air conduction thresholds ensure accurate measurement of hearing thresholds in the presence of asymmetrical hearing loss or cross-hearing, which is critical for fitting hearing aids.
- B (Bone conduction thresholds for both ears): This is necessary. Bone conduction thresholds help determine the type of hearing loss (conductive, sensorineural, or mixed) and are essential for appropriate hearing aid selection.
- C (TABR threshold): This is least necessary. Tone-ABR (TABR) is typically used for objective threshold estimation in infants or individuals who cannot provide reliable behavioral responses. For an adult like Mr. Zhang, behavioral audiometry (air and bone conduction) is sufficient for hearing aid fitting.
- D (Consultation on current living environment): This is necessary. Understanding Mr. Zhang's daily listening environments (e.g., quiet vs. noisy) is crucial for selecting hearing aids with appropriate features (e.g., noise reduction, directional microphones).

Correct Answer: C (TABR threshold) is the least necessary assessment for hearing aid selection in this case.

Question 42: Which of the following statements about the effects of noise on hearing is incorrect?

Answer: A

Explanation:

- A: This is incorrect. Low-frequency noise exposure typically causes less threshold shift compared to high-frequency noise exposure. High-frequency noise is more damaging to the cochlea, particularly the basal region, which is responsible for high-frequency hearing.
- B: This is correct. The maximum temporary threshold shift (TTS) usually occurs at a frequency half an octave above the frequency of the noise exposure.
- C: This is correct. TTS is more likely to occur in individuals with pre-existing sensorineural hearing loss, as their auditory system is more vulnerable to additional noise damage.
- D: This is correct. Asymptotic threshold shift (ATS) refers to the maximum TTS that can occur after prolonged exposure to a specific noise level, regardless of the duration of exposure.

Correct Answer: A is the incorrect statement.

Question 43: A 36-year-old woman, Ah Mei, has been troubled by right ear tinnitus. She has no symptoms of otitis media, nasal allergies, or nasal congestion. Her PTA shows mild sensorineural hearing loss, and her Acoustic Reflex Threshold (ART) test shows no response. What is the most likely diagnosis?

Answer: B (Retrocochlear pathology)

Explanation:

- A (Cochlear pathology): While cochlear pathology (e.g., noise-induced hearing loss, presbycusis) can cause tinnitus and sensorineural hearing loss, the absence of acoustic reflexes (ART) suggests a problem beyond the cochlea.
- B (Retrocochlear pathology): This is the most likely diagnosis. The combination of unilateral tinnitus, mild sensorineural hearing loss, and absent acoustic reflexes strongly suggests a retrocochlear lesion, such as an acoustic neuroma or other auditory nerve pathology.
- C (Nasopharyngeal carcinoma): While nasopharyngeal carcinoma can cause unilateral hearing loss and tinnitus, it is typically associated with other symptoms (e.g., nasal obstruction, epistaxis), which Ah Mei does not have.
- D (Psychosomatic disorder): Psychosomatic disorders can cause tinnitus, but they do not explain the objective findings of hearing loss and absent acoustic reflexes.

Correct Answer: B (Retrocochlear pathology) is the most likely diagnosis.

Question 44: Based on the previous question, which of the following tests is least necessary for the audiologist to recommend or perform?

Answer: C (TEN test)

Explanation:

- A (Speech recognition test to obtain PI-PB function): This is necessary. Speech recognition testing can provide valuable information about the patient's ability to understand speech, which is important for diagnosing retrocochlear pathology.
- B (Tone Decay Test): This is necessary. The Tone Decay Test helps identify abnormal adaptation, which is often associated with retrocochlear lesions.
- C (TEN test): This is least necessary. The Threshold Equalizing Noise (TEN) test is used to diagnose dead regions in the cochlea, which is not relevant to retrocochlear pathology. It is more applicable to cochlear hearing loss.
- D (Neurodiagnostic ABR): This is necessary. Neurodiagnostic ABR is critical for evaluating the integrity of the auditory nerve and brainstem, which is essential for diagnosing retrocochlear lesions.

Correct Answer: C (TEN test) is the least necessary test in this case.

Question 45: Which of the following statements about otosclerosis is incorrect?

Answer: D

Explanation:

- A: This is correct. In the later stages of otosclerosis, the disease can progress to involve the cochlea, resulting in a mixed hearing loss (conductive and sensorineural components).
- B: This is correct. The typical symptoms of otosclerosis (e.g., progressive hearing loss, tinnitus) often begin in late teens to early 30s.
- C: This is correct. Otosclerosis has both genetic and non-genetic factors contributing to its development, including hormonal changes and viral infections.
- D: This is incorrect. Otosclerosis is more common in women than in men, with a ratio of approximately 2:1. Hormonal factors, such as pregnancy, are thought to play a role in this disparity.

Correct Answer: D is the incorrect statement.

Question 46: Based on the provided audiogram, which of the following statements is correct?

Answer: C

Explanation:

- A (Left ear needs additional testing at 6000 Hz): This is incorrect. The audiogram does not indicate a specific need for testing at 6000 Hz for the left ear. The standard frequencies (250 Hz to 8000 Hz) are typically sufficient for a comprehensive evaluation.
- B (Left ear 4000 Hz needs masked bone conduction thresholds): This is incorrect. Masked bone conduction thresholds are typically required when there is a significant air-bone gap or asymmetrical hearing loss. The audiogram does not show a clear indication for masking at 4000 Hz for the left ear.
- C (Right ear air conduction thresholds at 250, 500, and 1000 Hz need masked air conduction thresholds): This is correct. If there is a significant difference between the air conduction thresholds of the right and left ears, masking is necessary to ensure accurate measurement of the right ear's thresholds at these frequencies.
- D (Right ear has mild to moderate conductive hearing loss): This is incorrect. The audiogram does not provide enough information to conclude that the right ear has conductive hearing loss. Conductive hearing loss is characterized by an air-bone gap, which is not evident from the provided data.

Correct Answer: C is the correct statement.

Question 47: Mr. Zhang, a kitchen worker, underwent a Pure-Tone Audiometry (PTA) test due to difficulty hearing (results shown in the audiogram). Which of the following additional test results is most consistent with this audiogram?

Answer: C (TABR results: right ear 40 dB, left ear 90 dB)

Explanation:

- A (Unmasked SRT: right ear 45 dB, left ear 70 dB): This is inconsistent with the audiogram. The Speech Recognition Threshold (SRT) should closely match the pure-tone average (PTA) of the better ear. The right ear PTA is around 50 dB, so an SRT of 45 dB is plausible, but the left ear PTA is around 105 dB, making an SRT of 70 dB unlikely.
- B (Bilateral Type A tympanograms): This is inconsistent with the audiogram. Type A tympanograms indicate normal middle ear function, but the audiogram shows a significant air-bone gap in the left ear, suggesting possible conductive or mixed hearing loss, which would not align with normal middle ear function.
- C (TABR results: right ear 40 dB, left ear 90 dB): This is consistent with the audiogram. The Tone-ABR (TABR) thresholds should approximate the behavioral thresholds. The right ear PTA is around 50 dB, and the left ear PTA is around 105 dB, so TABR results of 40 dB (right) and 90 dB (left) are plausible and consistent with the audiogram.
- D (Bilateral Speech Discrimination Scores (SDS) of 70%): This is inconsistent with the audiogram. The left ear shows severe hearing loss (105 dB), which would likely result in a much lower SDS than 70%. The right ear, with moderate hearing loss (50 dB), might have a higher SDS, but bilateral scores of 70% are unlikely given the severity of the left ear's hearing loss.

Correct Answer: C is the most consistent with the audiogram.

Question 48: When using supra-aural headphones for pure-tone audiometry, under which condition is masking of the non-test ear required?

Answer: A (When the air conduction threshold of the test ear and the bone conduction threshold of the non-test ear differ by 40 dB)

Explanation:

- A: This is correct. Masking is required when the air conduction threshold of the test ear is 40 dB or more above the bone conduction threshold of the non-test ear. This is because sound presented to the test ear can cross over to the non-test ear via bone conduction, potentially leading to inaccurate results.
- B: This is incorrect. Masking is not required when the air conduction thresholds of both ears differ by less than 40 dB, as the risk of crossover is minimal.
- C: This is incorrect. If the bone conduction thresholds of both ears are the same, masking is not necessary unless the air conduction thresholds differ significantly.
- D: This is incorrect. If the air conduction threshold of the test ear is the same as the bone conduction threshold of the non-test ear, masking is not required because there is no significant difference to cause crossover.

Correct Answer: A is the condition under which masking is required.

Question 49: Ms. Huang, a 55-year-old woman, experienced tinnitus in her left ear for three days and underwent a hearing test using TDH-49 headphones (report shown in the audiogram). According to Turner's (2004) optimized masking method for asymmetric hearing loss, what is the best initial masking level for air conduction at 2000 Hz?

Answer: C (45 dB)

Explanation:

- Turner's optimized masking method involves calculating the initial masking level based on the non-test ear's bone conduction threshold and the interaural attenuation (IA) for the specific transducer used. For TDH-49 headphones, the IA is approximately 40 dB.
- The formula for the initial masking level is:
  Initial Masking Level = Non-test ear bone conduction threshold + IA + 10 dB safety factor.
- From the audiogram, the non-test ear's bone conduction threshold at 2000 Hz is approximately 5 dB HL.

- Plugging in the values:
  Initial Masking Level = 5 dB + 40 dB + 10 dB = 55 dB.
- However, the closest option provided is 45 dB, which is a reasonable approximation for clinical purposes.

Correct Answer: C (45 dB) is the best initial masking level.

Question 50: Based on the previous question, if the Speech Recognition Threshold (SRT) is 0 dB HL for the right ear and 50 dB HL for the left ear, is further speech masking testing required, and what is the correct reasoning?

Answer: C (Masking is required for the right ear because the left ear's speech output level minus the right ear's best bone conduction threshold is greater than or equal to the interaural attenuation (IA) value.)

Explanation:

- A: This is incorrect. Masking is required when the difference between the ears' SRTs exceeds the interaural attenuation (IA) value. The IA for TDH-49 headphones is approximately 40 dB, and the difference between the right and left ear SRTs is 50 dB, which exceeds the IA. Therefore, masking is necessary.
- B: This is incorrect. Masking is required because the difference between the ears' SRTs (50 dB) exceeds the IA value (40 dB), indicating potential crossover of the signal to the non-test ear.
- C: This is correct. Masking is required for the right ear because the left ear's speech output level (50 dB) minus the right ear's best bone conduction threshold (0 dB) equals 50 dB, which is greater than the IA value (40 dB). This indicates that the signal presented to the left ear could cross over to the right ear, necessitating masking.
- D: This is incorrect. The calculation should compare the left ear's speech output level to the right ear's best bone conduction threshold, not the left ear's bone conduction threshold. The right ear's bone conduction threshold is the relevant value for determining crossover.

Correct Answer: C is the correct reasoning.

3106

Question 1: Which tympanogram gradient is most associated with middle ear effusion?

Answer: A (<0.2)

Explanation:

- A (<0.2): This is correct. A low gradient (<0.2) is strongly associated with middle ear effusion, as it indicates a stiff or non-compliant middle ear system, often due to fluid accumulation.
- B (0.4–0.6): This is incorrect. A gradient in this range is considered normal and is not associated with middle ear effusion.
- C (>235): This is incorrect. This value does not relate to tympanogram gradients and is not relevant to middle ear effusion.
- D (51–114): This is incorrect. This range does not correspond to tympanogram gradients and is unrelated to middle ear effusion.

Correct Answer: A (<0.2) is most associated with middle ear effusion.

Question 2: Which condition results in the highest total admittance value?

Answer: A (Perforated eardrum)

Explanation:

- A (Perforated eardrum): This is correct. A perforated eardrum results in the highest total admittance because sound can easily pass through the perforation into the middle ear, reducing resistance and increasing the ease of sound transmission.
- B (Healed eardrum perforation): This is incorrect. A healed perforation typically results in normal or near-normal admittance, not the highest value.
- C (Earwax blockage): This is incorrect. Earwax blockage reduces admittance by obstructing the ear canal and increasing resistance to sound transmission.
- D (Middle ear fluid): This is incorrect. Middle ear fluid reduces admittance by stiffening the middle ear system, making it harder for sound to pass through.

Correct Answer: A (Perforated eardrum) results in the highest total admittance.

Question 3: Which type of tympanogram results in the highest sound pressure level (SPL) of the probe tone in the ear canal?

Answer: A (Type A)

Explanation:

- A (Type A): This is correct. A Type A tympanogram indicates normal middle ear function, with the eardrum and ossicular chain efficiently transmitting sound. This results in the highest SPL of the probe tone in the ear canal.
- B (Type B): This is incorrect. A Type B tympanogram (flat) is associated with conditions like middle ear effusion or eardrum perforation, which reduce the SPL of the probe tone.
- C (Type C): This is incorrect. A Type C tympanogram indicates negative middle ear pressure, which reduces the SPL of the probe tone compared to Type A.
- D (Type Ad): This is incorrect. A Type Ad tympanogram (hypercompliant) is associated with a highly mobile eardrum, such as in ossicular discontinuity, but it does not result in the highest SPL of the probe tone.

Correct Answer: A (Type A) results in the highest SPL of the probe tone.

Question 4: Which of the following statements about acoustic reflex testing is least appropriate?

Answer: C

Explanation:

- A: This is correct. Acoustic reflex testing includes both acoustic reflex threshold testing and acoustic reflex decay testing.
- B: This is correct. In individuals with normal hearing, the acoustic reflex threshold typically occurs at 85–100 dB SPL.
- C: This is incorrect. Acoustic reflex decay testing measures the reduction (not increase) in the reflex over time. A 50% reduction in the reflex within 10 seconds is considered abnormal, not a 50% increase.
- D: This is correct. Acoustic reflex testing evaluates the stapedius muscle's response to sound stimulation.

Correct Answer: C is the least appropriate statement.

Question 5: Which of the following statements about the principles and applications of the acoustic reflex is most appropriate?

Answer: C

Explanation:

- A: This is incorrect. A stimulus sound can elicit a contralateral acoustic reflex (in the opposite ear) as well as an ipsilateral reflex (in the same ear).

- B: This is incorrect. The presence of an acoustic reflex is typically determined by a change in tympanic membrane admittance of 0.02 mmhos or more, not 0.01 mmhos.
- C: This is correct. As the stimulus intensity increases, the change in tympanic membrane admittance caused by the acoustic reflex also increases.
- D: This is incorrect. The stapedius muscle (not the tensor tympani) is primarily responsible for the acoustic reflex and contributes more significantly to changes in middle ear impedance.

Correct Answer: C is the most appropriate statement.

Question 6: For the diagnosis of acoustic neuroma, which combination of tests with ABR is least appropriate?

Answer: C

Explanation:

- A: This is appropriate. Pure-tone audiometry combined with tone decay testing can help identify retrocochlear pathology, such as acoustic neuroma.
- B: This is appropriate. Middle ear function testing combined with acoustic reflex decay testing is useful for detecting retrocochlear lesions, including acoustic neuroma.
- C: This is least appropriate. Speech audiometry and PI-PB (Performance-Intensity Function for Phonetically Balanced Words) testing are more relevant for assessing speech recognition and cochlear function, not specifically for diagnosing acoustic neuroma.
- D: This is appropriate. Cochlear potential testing combined with masking level difference (MLD) can provide valuable information about retrocochlear pathology.

Correct Answer: C is the least appropriate combination.

Question 7: Which of the following statements about clinical acoustic reflex decay testing is most appropriate?

Answer: A

Explanation:

- A: This is correct. Acoustic reflex decay testing can record both ipsilateral (same ear) and contralateral (opposite ear) reflex decay responses.
- B: This is incorrect. The test frequencies for acoustic reflex decay are typically 500 Hz and 1000 Hz, not 2000 Hz and 4000 Hz.
- C: This is incorrect. The stimulus level for acoustic reflex decay testing is usually 10 dB above the acoustic reflex threshold, not 10 dB below.

- D: This is incorrect. Acoustic reflex decay testing is primarily used to detect retrocochlear pathology (e.g., acoustic neuroma), not cochlear lesions.

Correct Answer: A is the most appropriate statement.

Question 8: Which of the following statements about Transient Evoked Otoacoustic Emissions (TEOAE) suppression testing is correct?

Answer: A

Explanation:

- A: This is correct. Contralateral suppressor stimuli and ipsilateral stimuli should not use simultaneous masking because it can interfere with the accurate measurement of TEOAEs.
- B: This is incorrect. Backward masking is not typically used in TEOAE suppression testing.
- C: This is incorrect. Binaural suppression generally produces a greater suppression effect compared to contralateral suppression.
- D: This is incorrect. Forward masking is not typically used for ipsilateral suppression in TEOAE testing.

Correct Answer: A is the correct statement.

Question 9: Based on the hardware configuration of the hearing test instrument shown in the image, which of the following tests cannot be performed?

Answer: D (Stimulus Frequency Otoacoustic Emissions, SFOAE)

Explanation:

- A (Spontaneous Otoacoustic Emissions, SOAE): This test can be performed with the described hardware. SOAEs are measured without an external stimulus, and the system includes a microphone and analyzer, which are sufficient for detecting these emissions.
- B (Transient Evoked Otoacoustic Emissions, TEOAE): This test can be performed. The hardware includes a probe with a stimulus generator and a microphone, which are necessary for presenting clicks or tone bursts and recording the evoked responses.
- C (Distortion Product Otoacoustic Emissions, DPOAE): This test can be performed. The system includes the necessary components to generate two primary tones and measure the resulting distortion products.
- D (Stimulus Frequency Otoacoustic Emissions, SFOAE): This test cannot be performed with the described hardware. SFOAE testing requires a system capable of generating a continuous pure

tone and simultaneously measuring the evoked emissions at the same frequency, which is not supported by the typical configuration shown.

Correct Answer: D (SFOAE) cannot be performed with the described hardware.

Question 10: When performing Distortion Product Otoacoustic Emissions (DPOAE) with stimulus frequencies of 1000 Hz and 1200 Hz, what is the most prominent OAE frequency generated?

Answer: B (800 Hz)

Explanation:

- DPOAEs are generated at frequencies that are mathematical combinations of the two primary stimulus frequencies (f1 and f2). The most prominent DPOAE frequency is typically at 2f1 - f2.
- Given f1 = 1000 Hz and f2 = 1200 Hz, the calculation is:
  2f1 - f2 = 2(1000) - 1200 = 2000 - 1200 = 800 Hz.
- Therefore, the most prominent OAE frequency is 800 Hz.

Correct Answer: B (800 Hz) is the most prominent OAE frequency.

Question 11: Which cochlear structure is most closely related to Otoacoustic Emissions (OAEs)?

Answer: C (Outer hair cells)

Explanation:

- A (Vestibular aqueduct): This is incorrect. The vestibular aqueduct is related to the inner ear's fluid balance and is not involved in OAE generation.
- B (Inner hair cells): This is incorrect. Inner hair cells are primarily responsible for transmitting sound signals to the brain, not for generating OAEs.
- C (Outer hair cells): This is correct. Outer hair cells are responsible for amplifying sound vibrations and are directly involved in the generation of OAEs.
- D (Stria vascularis): This is incorrect. The stria vascularis is involved in maintaining the endocochlear potential but is not directly related to OAE generation.

Correct Answer: C (Outer hair cells) is most closely related to OAEs.

Question 12: Which of the following statements about Otoacoustic Emissions (OAEs) is correct?

Answer: D (Ipsilateral suppression of OAEs is greater than contralateral suppression.)

Explanation:

- A (Adult OAE amplitudes are larger than those of infants): This is incorrect. Infants typically have larger OAE amplitudes than adults due to the higher activity of their outer hair cells.
- B (High-frequency OAEs have longer latencies than low-frequency OAEs): This is incorrect. High-frequency OAEs generally have shorter latencies compared to low-frequency OAEs.
- C (Male OAE amplitudes are larger than those of females): This is incorrect. Females typically have larger OAE amplitudes than males, possibly due to differences in cochlear mechanics.
- D (Ipsilateral suppression of OAEs is greater than contralateral suppression): This is correct. Ipsilateral suppression (suppression on the same side as the stimulus) is generally stronger than contralateral suppression (suppression on the opposite side).

Correct Answer: D is the correct statement.

Question 13: Which structure is not part of the descending neural pathway involved in EOAE suppression?

Answer: A (Superior colliculus, SC)

Explanation:

- A (Superior colliculus, SC): This is correct. The superior colliculus is involved in visual and multisensory processing, not in the auditory pathway for EOAE suppression.
- B (Medial geniculate body, MGB): This is incorrect. The medial geniculate body is part of the auditory pathway and is involved in processing auditory information.
- C (Cochlear nucleus, CN): This is incorrect. The cochlear nucleus is the first relay station in the auditory pathway and is involved in EOAE suppression.
- D (Superior olivary complex, SOC): This is incorrect. The superior olivary complex plays a key role in binaural hearing and is part of the descending pathway for EOAE suppression.

Correct Answer: A (Superior colliculus) is not part of the descending neural pathway for EOAE suppression.

Question 14: What is the primary clinical purpose of performing bone-conduction click-evoked Auditory Brainstem Response (ABR)?

Answer: D (To assess the possibility of conductive hearing loss)

Explanation:

- A (To estimate bone-conduction hearing thresholds at specific frequencies): This is incorrect. Bone-conduction ABR is not typically used to estimate specific frequency thresholds.

- B (To confirm the accuracy of air-conduction click-evoked ABR thresholds): This is incorrect. Bone-conduction ABR is not primarily used to validate air-conduction ABR thresholds.
- C (To more accurately estimate pure-tone thresholds at 500 Hz compared to air-conduction clicks): This is incorrect. Bone-conduction ABR is not specifically used for estimating 500 Hz thresholds.
- D (To assess the possibility of conductive hearing loss): This is correct. Bone-conduction ABR is used to differentiate between conductive and sensorineural hearing loss by bypassing the outer and middle ear.

Correct Answer: D is the primary clinical purpose.

Question 15: When using Electrocochleography (ECochG) to diagnose auditory neuropathy, what is the key diagnostic criterion?

Answer: D (The presence of cochlear microphonic (CM) responses to both condensation and rarefaction click stimuli)

Explanation:

- A (The action potential (AP) response to condensation and rarefaction click stimuli): This is incorrect. In auditory neuropathy, the action potential is typically absent or abnormal, but this is not the primary diagnostic criterion.
- B (The presence of summating potential (SP) with alternating polarity click stimuli): This is incorrect. The summating potential is not the primary indicator of auditory neuropathy.
- C (The consistency of cochlear microphonic (CM) responses with increased stimulus rates): This is incorrect. While CM responses are important, their consistency with increased stimulus rates is not the key diagnostic criterion.
- D (The presence of cochlear microphonic (CM) responses to both condensation and rarefaction click stimuli): This is correct. In auditory neuropathy, the cochlear microphonic is typically present and robust, while the auditory brainstem response (ABR) is absent or abnormal. The CM response to both condensation and rarefaction clicks is a key diagnostic feature.

Correct Answer: D is the key diagnostic criterion.

Question 16: When performing Auditory Steady-State Response (ASSR) testing, if the modulation frequency of the stimulus is increased from 40 Hz to 80 Hz, which of the following statements is most appropriate?

Answer: B (Phase coherence decreases)

Explanation:

- A (Latency shortens): This is incorrect. Increasing the modulation frequency does not significantly affect the latency of the ASSR.
- B (Phase coherence decreases): This is correct. As the modulation frequency increases, the phase coherence (the consistency of the response phase) tends to decrease, making the response less stable.
- C (Does not affect hearing threshold estimation): This is incorrect. Higher modulation frequencies can affect the accuracy of hearing threshold estimation, especially in infants and young children.
- D (Amplitude increases): This is incorrect. Increasing the modulation frequency generally results in a decrease in response amplitude, not an increase.

Correct Answer: B is the most appropriate statement.

Question 17: Which of the following statements about enhancing Auditory Brainstem Response (ABR) wave amplitudes is least appropriate?

Answer: D (Using chirp stimuli to concentrate stimulation on high-frequency, low-threshold neurons)

Explanation:

- A (Increasing stimulus intensity to depolarize more nerve cells): This is correct. Increasing the stimulus intensity can enhance ABR wave amplitudes by activating more nerve cells.
- B (Moving the reference electrode closer to the cochlea to make Wave I more prominent): This is correct. Positioning the reference electrode closer to the cochlea can improve the detection of Wave I.
- C (Reducing the stimulus rate): This is correct. Lowering the stimulus rate can enhance ABR wave amplitudes by allowing more time for neural recovery between stimuli.
- D (Using chirp stimuli to concentrate stimulation on high-frequency, low-threshold neurons): This is incorrect. Chirp stimuli are designed to synchronize the activation of neurons across different frequency regions of the cochlea, not just high-frequency, low-threshold neurons. This synchronization enhances the overall ABR response, not specifically high-frequency responses.

Correct Answer: D is the least appropriate statement.

Question 18: Which of the following statements about ABR testing methods is least appropriate?

Answer: B (The interaural attenuation for bone conduction in a 2-month-old newborn is approximately 0 dB, so masking is required.)

Explanation:

- A (As stimulus intensity decreases, the increase in latency becomes greater): This is correct. Lower stimulus intensities result in longer latencies for ABR waves.
- B (The interaural attenuation for bone conduction in a 2-month-old newborn is approximately 0 dB, so masking is required): This is incorrect. The interaural attenuation for bone conduction is typically 0 dB, but masking is not always required unless there is a significant air-bone gap or asymmetrical hearing loss.
- C (Using insert earphones can reduce the need for masking): This is correct. Insert earphones provide higher interaural attenuation (40–70 dB) compared to supra-aural headphones, reducing the need for masking.
- D (Using alternating polarity stimuli can reduce stimulus artifact): This is correct. Alternating polarity helps cancel out stimulus artifacts, improving the clarity of the ABR response.

Correct Answer: B is the least appropriate statement.

Question 19: Which of the following statements about distinguishing the Summating Potential (SP) from the Action Potential (AP) in Electrocochleography (ECochG) is least appropriate?

Answer: B (Increasing the stimulus rate to 100 Hz)

Explanation:

- A (Using longer-duration tone bursts as stimuli): This is correct. Longer-duration tone bursts can help separate the SP from the AP by providing a more sustained response.
- B (Increasing the stimulus rate to 100 Hz): This is incorrect. High stimulus rates (e.g., 100 Hz) can cause adaptation and reduce the amplitude of the AP, making it harder to distinguish from the SP.
- C (Changing stimulus polarity at low intensities): This is correct. Changing the stimulus polarity can help differentiate the SP (which is polarity-sensitive) from the AP (which is less affected by polarity).
- D (Reducing stimulus intensity to below 60 dB nHL): This is correct. Lower stimulus intensities can help isolate the SP, as the AP may be less prominent at lower levels.

Correct Answer: B is the least appropriate statement.

Question 20: If a patient's ABR shows normal I-III interpeak latencies but prolonged IV-V interpeak latencies, where is the most likely site of dysfunction?

Answer: C (Rostral brainstem)

Explanation:

- A (Auditory nerve): This is incorrect. Dysfunction of the auditory nerve would typically affect Wave I and the I-III interpeak latency.
- B (Cochlear nucleus): This is incorrect. Dysfunction of the cochlear nucleus would typically affect Wave II and the I-III interpeak latency.
- C (Rostral brainstem): This is correct. Prolonged IV-V interpeak latency suggests dysfunction in the rostral brainstem, which includes the lateral lemniscus and inferior colliculus.
- D (Severe head injury): This is incorrect. While severe head injury can affect ABR, it does not specifically explain the isolated prolongation of the IV-V interpeak latency.

Correct Answer: C (Rostral brainstem) is the most likely site of dysfunction.

Question 21: Which of the following statements about Auditory Brainstem Response (ABR) testing methods is most appropriate?

Answer: B (Chirp stimuli use time compensation to reduce the time difference of the traveling wave from high to low frequencies, enhancing response amplitude.)

Explanation:

- A (Insert earphones reduce interaural attenuation): This is incorrect. Insert earphones actually increase interaural attenuation (40–70 dB) compared to supra-aural headphones, reducing the need for masking.
- B (Chirp stimuli use time compensation to reduce the time difference of the traveling wave from high to low frequencies, enhancing response amplitude): This is correct. Chirp stimuli are designed to synchronize the activation of neurons across different frequency regions of the cochlea, reducing temporal dispersion and enhancing the ABR response amplitude.
- C (Click stimuli cause the traveling wave to accelerate at the apex of the cochlea, a phenomenon called temporal dispersion): This is incorrect. Temporal dispersion refers to the spreading out of neural responses due to differences in travel time along the cochlea, not acceleration at the apex.
- D (Bone-conduction headphones concentrate energy around 500–1000 Hz, and their output can exceed 60 dB HL): This is incorrect. Bone-conduction headphones do not typically exceed 60 dB HL in output, and their energy is not specifically concentrated around 500–1000 Hz.

Correct Answer: B is the most appropriate statement.

Question 22: Which of the following statements about typical auditory neuropathy is most appropriate?

Answer: B (ABR is absent, but OAEs are present.)

Explanation:

- A (ABR is normal, and OAEs are normal): This is incorrect. In auditory neuropathy, the ABR is typically absent or abnormal, while OAEs may be present.
- B (ABR is absent, but OAEs are present): This is correct. Auditory neuropathy is characterized by absent or abnormal ABR due to dyssynchronous neural activity, while OAEs are often preserved because outer hair cell function remains intact.
- C (ABR is normal, but OAEs are absent): This is incorrect. A normal ABR with absent OAEs suggests cochlear hearing loss, not auditory neuropathy.
- D (ABR is absent, and OAEs are absent): This is incorrect. Absent OAEs with absent ABR suggests cochlear hearing loss, not auditory neuropathy.

Correct Answer: B is the most appropriate statement.

Question 23: Which of the following statements about the application of auditory evoked potentials is least appropriate?

Answer: D (Mismatch Negativity, MMN, can be measured using the same parameters as ABR.)

Explanation:

- A (ABR can be used in sleeping patients): This is correct. ABR is an objective test that does not require patient participation and can be performed while the patient is asleep.
- B (LLR can use speech stimuli to assess higher-level auditory function): This is correct. Late Latency Responses (LLR) can be elicited by speech stimuli to evaluate cortical auditory processing.
- C (MLR can be used to diagnose auditory processing disorders, but the results are inconclusive): This is correct. Middle Latency Responses (MLR) have been explored for diagnosing auditory processing disorders (APD), but their diagnostic utility is not yet definitive.
- D (MMN can be measured using the same parameters as ABR): This is incorrect. Mismatch Negativity (MMN) is a cortical response that requires different parameters (e.g., oddball paradigm, slower stimulus rates) compared to ABR, which focuses on brainstem responses.

Correct Answer: D is the least appropriate statement.

Question 24: Which of the following statements about the effect of stimulus rate on Auditory Brainstem Response (ABR) is incorrect?

Answer: C (Increasing the stimulus rate increases the amplitude.)

Explanation:

- A (Increasing the stimulus rate increases latency): This is correct. Faster stimulus rates result in longer latencies due to reduced neural recovery time.
- B (Wave V amplitude is less affected than Wave I amplitude): This is correct. Wave V is more resistant to changes in stimulus rate compared to Wave I, which is more sensitive to rate increases.
- C (Increasing the stimulus rate increases the amplitude): This is incorrect. Faster stimulus rates typically decrease the amplitude of ABR waves due to neural adaptation and reduced synchronization.
- D (Wave V latency is more affected than Wave I latency): This is correct. Wave V latency is more sensitive to changes in stimulus rate compared to Wave I.

Correct Answer: C is the incorrect statement.

Question 25: Which of the following is not a clinical application of Auditory Steady-State Response (ASSR)?

Answer: D (Diagnosis of auditory neuropathy spectrum disorder.)

Explanation:

- A (Predicting hearing thresholds): This is correct. ASSR is commonly used to estimate hearing thresholds, especially in infants and individuals who cannot provide reliable behavioral responses.
- B (Evaluating hearing aid effectiveness): This is correct. ASSR can be used to assess the benefit of hearing aids by measuring responses to amplified sounds.
- C (Preoperative evaluation for cochlear implants): This is correct. ASSR can help determine candidacy for cochlear implants by providing objective threshold estimates.
- D (Diagnosis of auditory neuropathy spectrum disorder): This is incorrect. ASSR is not typically used to diagnose auditory neuropathy spectrum disorder (ANSD). Instead, ABR and OAE tests are more commonly used for this purpose.

Correct Answer: D is not a clinical application of ASSR.

Question 26: Which of the following statements about signal averaging in auditory evoked responses is incorrect?

Answer: B (The signal increases n times as n increases.)

Explanation:

- A (The signal is mostly constant and is not canceled out during averaging): This is correct. The signal is consistent across trials and is reinforced during averaging.

- B (The signal increases n times as n increases): This is incorrect. The signal increases by a factor of n, but the amplitude of the signal increases by a factor of √n, not n times.
- C (Noise is mostly random and is often canceled out during averaging): This is correct. Noise is random and tends to cancel out as the number of averages increases.
- D (Noise decreases by a factor of the square root of n): This is correct. The noise amplitude decreases by a factor of √n as the number of averages increases.

Correct Answer: B is the incorrect statement.

Question 27: Which of the following is not a cause of prolonged ABR latency values?

Answer: D (Decreasing the stimulus presentation rate.)

Explanation:

- A (Conductive hearing loss in the individual): This is correct. Conductive hearing loss can prolong ABR latencies due to reduced sound transmission to the cochlea.
- B (Incomplete development of the central nervous system in newborns): This is correct. In newborns, the immature central nervous system can result in prolonged ABR latencies.
- C (Stimulus intensity near hearing threshold): This is correct. When the stimulus intensity is close to the hearing threshold, ABR latencies are typically prolonged.
- D (Decreasing the stimulus presentation rate): This is incorrect. Decreasing the stimulus rate generally shortens ABR latencies because it allows more time for neural recovery between stimuli.

Correct Answer: D is not a cause of prolonged ABR latency values.

Question 28: Which of the following methods or measures can be used to reduce noise and obtain a better signal in Auditory Brainstem Response (ABR)? ①Differential amplification ②Bandpass filtering ③Averaging ④Sequential high-pass masking

Answer: C (Only ①②③)

Explanation:

- ①Differential amplification: This is correct. Differential amplification helps reduce common-mode noise by amplifying the difference between two input signals.
- ②Bandpass filtering: This is correct. Bandpass filtering removes noise outside the frequency range of interest, improving the signal-to-noise ratio.
- ③Averaging: This is correct. Averaging multiple responses reduces random noise while reinforcing the consistent ABR signal.

- ④Sequential high-pass masking: This is incorrect. Sequential high-pass masking is not a standard method for reducing noise in ABR recordings.

Correct Answer: C (Only ①②③) are effective methods for reducing noise in ABR.

Question 29: Which of the following statements about recording auditory evoked potentials is least appropriate?

Answer: B (The response is time-locked but not phase-locked.)

Explanation:

- A (In dual-channel recording, different channels share the same ground electrode): This is correct. In dual-channel recordings, the same ground electrode is typically used for both channels.
- B (The response is time-locked but not phase-locked): This is incorrect. Auditory evoked potentials are both time-locked and phase-locked to the stimulus, meaning they occur at a consistent time and phase relative to the stimulus.
- C (Stimulus onset asynchrony and stimulus rate are reciprocals): This is correct. Stimulus onset asynchrony (SOA) is the time between successive stimuli and is the reciprocal of the stimulus rate.
- D (According to the international 10-20 electrode placement system, A2 represents the right earlobe): This is correct. In the 10-20 system, A2 refers to the right earlobe electrode.

Correct Answer: B is the least appropriate statement.

Question 30: Compared to males, which of the following statements about females' ABR is correct?

Answer: B (Shorter interpeak latencies and larger amplitudes.)

Explanation:

- A (Shorter interpeak latencies and smaller amplitudes): This is incorrect. Females typically have larger amplitudes, not smaller.
- B (Shorter interpeak latencies and larger amplitudes): This is correct. Females generally exhibit shorter interpeak latencies and larger amplitudes in ABR due to differences in head size and neural conduction velocity.
- C (Longer interpeak latencies and smaller amplitudes): This is incorrect. Females do not have longer interpeak latencies or smaller amplitudes.
- D (Longer interpeak latencies and larger amplitudes): This is incorrect. Females do not have longer interpeak latencies.

Correct Answer: B is the correct statement.

Question 31: Which of the following statements about using ABR to detect vestibular schwannoma (acoustic neuroma) is least appropriate?

Answer: C (The tumor has affected low-frequency auditory nerve fibers.)

Explanation:

- A (The tumor has substantially compressed the auditory nerve and blocked neural transmission): This is correct. ABR can detect vestibular schwannoma when the tumor compresses the auditory nerve, disrupting neural transmission.
- B (The tumor has affected enough auditory nerve fibers): This is correct. ABR abnormalities are more likely when a significant number of auditory nerve fibers are affected.
- C (The tumor has affected low-frequency auditory nerve fibers): This is incorrect. Vestibular schwannomas typically affect high-frequency auditory nerve fibers first, not low-frequency fibers.
- D (The tumor has substantially compressed the auditory nerve and caused desynchronization): This is correct. ABR can detect vestibular schwannoma when the tumor causes desynchronization of neural activity.

Correct Answer: C is the least appropriate statement.

Question 32: Which of the following statements about Auditory Steady-State Response (ASSR) and its amplitude-modulated stimuli is correct?

Answer: D (The 40 Hz steady-state response becomes similar to adults after the age of two.)

Explanation:

- A (Higher modulation frequencies are more affected by the subject's alertness): This is incorrect. Lower modulation frequencies (e.g., 40 Hz) are more affected by alertness, while higher frequencies (e.g., 80 Hz) are less affected.
- B (Higher modulation frequencies primarily originate from higher auditory systems, such as the auditory cortex): This is incorrect. Lower modulation frequencies (e.g., 40 Hz) are associated with cortical responses, while higher frequencies (e.g., 80 Hz) are associated with brainstem responses.
- C (Infants and young children show larger responses to 80 Hz modulation frequencies compared to 40 Hz): This is incorrect. Infants and young children typically show larger responses to 40 Hz modulation frequencies, not 80 Hz.
- D (The 40 Hz steady-state response becomes similar to adults after the age of two): This is correct. The 40 Hz ASSR matures and becomes similar to adult responses after the age of two.

Correct Answer: D is the correct statement.

Question 33: Which of the following statements about the clinical application of late latency auditory evoked potentials is least appropriate?

Answer: D (Cannot be measured in individuals with auditory neuropathy spectrum disorder, ANSD.)

Explanation:

- A (Can be used as a reference for hearing aid settings in infants with hearing loss): This is correct. Late latency responses can provide information about cortical processing, which is useful for fitting hearing aids.
- B (Can be used as a tool to measure the maturity of the auditory system): This is correct. Late latency responses reflect the development of the auditory cortex and can be used to assess auditory system maturity.
- C (Can evaluate the effectiveness of hearing assistive devices): This is correct. Late latency responses can help assess the benefit of hearing aids or cochlear implants by measuring cortical responses to sound.
- D (Cannot be measured in individuals with auditory neuropathy spectrum disorder, ANSD): This is incorrect. Late latency responses can be measured in individuals with ANSD, although the responses may be abnormal due to dyssynchronous neural activity.

Correct Answer: D is the least appropriate statement.

Question 34: Which of the following statements about the clinical application of electrocochleography (ECochG) is least appropriate?

Answer: A (Cochlear microphonic, CM, can be used to confirm the function of inner hair cells.)

Explanation:

- A (CM can be used to confirm the function of inner hair cells): This is incorrect. The cochlear microphonic (CM) reflects the activity of outer hair cells, not inner hair cells.
- B (Compound action potential, CAP, can be used to confirm synaptic communication between inner hair cells and afferent auditory fibers): This is correct. The CAP is generated by the synchronous firing of auditory nerve fibers in response to inner hair cell activity.
- C (In Ménière's disease, the summating potential, SP, amplitude is often abnormally large): This is correct. In Ménière's disease, the SP is often enlarged due to endolymphatic hydrops.
- D (The SP/AP amplitude ratio is a useful tool for diagnosing Ménière's disease): This is correct. An elevated SP/AP ratio is a hallmark of Ménière's disease and is commonly used in diagnosis.

Correct Answer: A is the least appropriate statement.

Question 35. Which of the following descriptions of electrocochleography (ECochG) is most appropriate?

Options:
A. Can be used intraoperatively to assess the effectiveness of cochlear implant placement
B. Cannot be used to evaluate hearing thresholds
C. A normal SP/AP amplitude ratio rules out Ménière's disease
D. A higher SP/AP amplitude ratio indicates Ménière's disease

Correct Answer: B. Cannot be used to evaluate hearing thresholds

Explanation:

- A. Can be used intraoperatively to assess the effectiveness of cochlear implant placement
  Electrocochleography (ECochG) is primarily used to assess cochlear function, particularly in cases of inner ear disorders like Ménière's disease. It is not used to evaluate the effectiveness of cochlear implants, so this option is incorrect.
- B. Cannot be used to evaluate hearing thresholds
  This is correct. ECochG is used to diagnose inner ear pathologies, not to directly measure hearing thresholds. Hearing thresholds are typically assessed using pure-tone audiometry.
- C. A normal SP/AP amplitude ratio rules out Ménière's disease
  While an abnormal SP/AP ratio (typically >0.4) may suggest Ménière's disease, a normal ratio does not completely rule it out. Therefore, this option is incorrect.
- D. A higher SP/AP amplitude ratio indicates Ménière's disease
  A higher SP/AP ratio (>0.4) can be an indicator of Ménière's disease, but it is not definitive. Other inner ear disorders can also cause an elevated ratio, so this option is not entirely correct.

Question 36. Compared to late latency responses, which of the following statements about early latency responses is most appropriate?

① Requires a lower stimulation rate
② Has a lower frequency composition
③ Less affected by alertness
④ Has smaller response amplitudes
⑤ Has larger noise amplitudes

Options:
A. ①②
B. ③④
C. ②③
D. ③⑤

Correct Answer: B. ③④

Explanation:

- ① Requires a lower stimulation rate
  Early latency responses (e.g., ABR) typically require higher stimulation rates to elicit responses, so this statement is incorrect.

- ② Has a lower frequency composition
  Early latency responses (e.g., ABR) primarily reflect high-frequency auditory nerve activity, so this statement is incorrect.

- ③ Less affected by alertness
  This is correct. Early latency responses (e.g., ABR) are generated by the brainstem and are not influenced by the subject's level of alertness or attention.

- ④ Has smaller response amplitudes
  This is correct. Early latency responses (e.g., ABR) generally have smaller amplitudes because they reflect brainstem activity.

- ⑤ Has larger noise amplitudes
  Early latency responses (e.g., ABR) typically require signal averaging to reduce noise, so noise amplitudes are not necessarily larger. This statement is incorrect.

In conclusion, the correct answer is B. ③④.

Question 37. A 5-year-old boy has prolonged absolute latency of wave V in air-conducted click ABR at near-threshold levels, normal interwave latencies, and abnormal bone-conducted click ABR. What is the most likely diagnosis for this boy?

Options:
A. Normal hearing
B. Conductive hearing loss
C. Sensorineural hearing loss
D. Neural hearing loss

Correct Answer: B. Conductive hearing loss

Explanation:

- Prolonged absolute latency of wave V in air-conducted click ABR suggests a delay in the transmission of sound through the outer or middle ear, which is characteristic of conductive hearing loss.

- Normal interwave latencies indicate that the neural pathways in the brainstem are functioning normally, ruling out neural or retrocochlear pathologies.

- Abnormal bone-conducted click ABR further supports conductive hearing loss, as bone conduction bypasses the outer and middle ear. If the bone-conducted ABR is also abnormal, it suggests a problem in the outer or middle ear rather than the cochlea or auditory nerve.

Thus, the most likely diagnosis is B. Conductive hearing loss.

Question 38. Little Mi, a 2-year-old girl, has inconsistent auditory responses. She was diagnosed with hearing loss and fitted with hearing aids, but it was later discovered that she has auditory neuropathy spectrum disorder (ANSD). Which of the following statements is most appropriate?

Options:
A. The cochlear microphonic (CM) waveform will show phase reversal when stimulus polarity is changed.
B. Cochlear implantation is less suitable for Little Mi.
C. Both OAE and ABR results are abnormal.
D. Binaural ASSR recordings can diagnose ANSD.

Correct Answer: A. The cochlear microphonic (CM) waveform will show phase reversal when stimulus polarity is changed.

Explanation:

- A. The cochlear microphonic (CM) waveform will show phase reversal when stimulus polarity is changed.
  This is correct. In ANSD, the cochlear microphonic (CM) is typically present and will show a phase reversal when the stimulus polarity is reversed, indicating that outer hair cell function is preserved.
- B. Cochlear implantation is less suitable for Little Mi.
  This is incorrect. Cochlear implantation can be beneficial for individuals with ANSD, especially if hearing aids do not provide sufficient benefit.
- C. Both OAE and ABR results are abnormal.
  This is incorrect. In ANSD, OAEs are typically present (indicating normal outer hair cell function), while ABR is abnormal or absent (indicating neural dyssynchrony).
- D. Binaural ASSR recordings can diagnose ANSD.
  This is incorrect. While ASSR can provide information about hearing thresholds, it is not specific for diagnosing ANSD. Diagnosis of ANSD relies on the presence of OAEs with abnormal or absent ABR.

Thus, the most appropriate statement is A. The cochlear microphonic (CM) waveform will show phase reversal when stimulus polarity is changed.

Question 39. When performing distortion product otoacoustic emissions (DPOAE) testing in infants and young children, two tones with frequencies f1 and f2 (f2 > f1) are presented. At which of the following f2/f1 ratios is the strongest response typically observed?

Options:
A. 1.11
B. 1.22

C. 1.33
D. 1.44

Correct Answer: B. 1.22

Explanation:

- The optimal f2/f1 ratio for eliciting the strongest DPOAE response is typically around 1.22. This ratio provides the best balance between the interaction of the two tones and the generation of distortion products in the cochlea.
- Ratios significantly higher or lower than 1.22 (e.g., 1.11, 1.33, or 1.44) generally produce weaker DPOAE responses.

Thus, the correct answer is B. 1.22.

Question 40. A full-term newborn underwent hearing screening on the second day after birth. The results showed a pass on aABR but a fail on OAE. What is the most likely cause?

Options:
A. Moderate sensory hearing loss
B. Mild neural hearing loss
C. Moderate neural hearing loss
D. Mild conductive hearing loss

Correct Answer: D. Mild conductive hearing loss

Explanation:

- aABR (automated auditory brainstem response) assesses the entire auditory pathway, including the cochlea, auditory nerve, and brainstem. A "pass" on aABR suggests that the auditory pathway is functioning adequately.
- OAE (otoacoustic emissions) evaluates the function of the outer hair cells in the cochlea. A "fail" on OAE indicates a problem with outer hair cell function or sound transmission through the outer and middle ear.
- In this case, the most likely explanation is a mild conductive hearing loss, such as temporary middle ear fluid or debris in the ear canal, which can block OAE responses but not significantly affect aABR.
- Options A, B, and C are less likely because they would typically result in abnormal aABR findings as well.

Thus, the most likely cause is D. Mild conductive hearing loss.

Question 41. Which of the following is not a recommended tool for newborn hearing screening?

Options:
A. Automated auditory brainstem response (aABR)
B. Transient evoked otoacoustic emissions (TEOAE)
C. Distortion product otoacoustic emissions (DPOAE)
D. Auditory steady-state response (ASSR)

Correct Answer: D. Auditory steady-state response (ASSR)

Explanation:

- aABR, TEOAE, and DPOAE are widely used and recommended tools for newborn hearing screening because they are objective, non-invasive, and effective for detecting hearing loss in infants.
- ASSR is primarily used for estimating hearing thresholds in diagnostic testing, not for initial newborn hearing screening. It is more complex and time-consuming, making it less suitable for universal screening programs.

Thus, the tool not recommended for newborn hearing screening is D. Auditory steady-state response (ASSR).

Question 42. When using tympanometry for middle ear screening in children, which of the following is the most appropriate criterion for determining a pass or fail result?

Options:
A. Peak pressure (TPP) and/or tympanogram type
B. Static compensated admittance (YTM) and/or tympanogram width (TW)
C. Equivalent ear canal volume (Vec) and/or tympanogram type
D. Static compensated admittance (YTM) and/or tympanogram type

Correct Answer: B. Static compensated admittance (YTM) and/or tympanogram width (TW)

Explanation:

- Static compensated admittance (YTM) measures the mobility of the tympanic membrane and middle ear system. Abnormal values can indicate middle ear dysfunction.
- Tympanogram width (TW) reflects the compliance of the middle ear system. A wide TW is often associated with middle ear effusion or other pathologies.
- Peak pressure (TPP) and tympanogram type are also important, but they are less specific for detecting middle ear dysfunction in children compared to YTM and TW.
- Equivalent ear canal volume (Vec) is used to assess the volume of the ear canal and is not directly related to middle ear function.

Thus, the most appropriate criterion for determining a pass or fail result is B. Static compensated admittance (YTM) and/or tympanogram width (TW).

Question 43. Which of the following statements about newborn hearing screening and intervention is least appropriate?

Options:
A. All newborns should undergo newborn hearing screening.
B. Infants who do not pass newborn hearing screening should receive diagnostic ABR testing by 3 months of age.
C. Infants diagnosed with hearing loss should receive medical intervention by 6 months of age.
D. Infants with bilateral profound deafness should receive cochlear implantation by 8 months of age.

Correct Answer: D. Infants with bilateral profound deafness should receive cochlear implantation by 8 months of age.

Explanation:

- A. All newborns should undergo newborn hearing screening.
  This is appropriate. Universal newborn hearing screening is recommended to ensure early identification of hearing loss.
- B. Infants who do not pass newborn hearing screening should receive diagnostic ABR testing by 3 months of age.
  This is appropriate. Early diagnostic testing is critical to confirm hearing loss and initiate timely intervention.
- C. Infants diagnosed with hearing loss should receive medical intervention by 6 months of age.
  This is appropriate. The "1-3-6" guideline recommends hearing screening by 1 month, diagnosis by 3 months, and intervention by 6 months of age.
- D. Infants with bilateral profound deafness should receive cochlear implantation by 8 months of age.
  This is least appropriate. While early cochlear implantation is beneficial, the timing depends on individual factors such as medical readiness, family readiness, and thorough evaluation. There is no strict requirement for implantation by 8 months of age.

Thus, the least appropriate statement is D. Infants with bilateral profound deafness should receive cochlear implantation by 8 months of age.

Question 44. If an infant has risk factors for delayed or progressive sensorineural hearing loss, even if they pass newborn hearing screening, until what age is it recommended to continue hearing monitoring?

Options:
A. 3 years
B. 4 years
C. 5 years
D. 6 years

Correct Answer: A. 3 years

Explanation:

- Infants with risk factors for delayed or progressive hearing loss (e.g., family history of hearing loss, congenital infections, or syndromes) should undergo ongoing hearing monitoring, even if they pass initial newborn hearing screening.
- The Joint Committee on Infant Hearing (JCIH) recommends that these children receive regular hearing evaluations until 3 years of age to ensure timely identification of any developing hearing loss.

Thus, the recommended age for continued monitoring is A. 3 years.

Question 45. When conducting a hearing assessment for a 5-month-old infant with normal development, which of the following statements is most appropriate?

Options:
A. A 226 Hz probe tone should be used for middle ear function testing.
B. During sound field behavioral hearing testing, play conditioning can be used to obtain hearing thresholds.
C. If the difference between electrophysiological testing and minimal response levels (MRL) is 20 dB, the behavioral hearing test results are unreliable.
D. At this stage, electrophysiological testing should be the primary assessment, supplemented by behavioral testing for further management and recommendations.

Correct Answer: D. At this stage, electrophysiological testing should be the primary assessment, supplemented by behavioral testing for further management and recommendations.

Explanation:

- A. A 226 Hz probe tone should be used for middle ear function testing.
  This is incorrect. For infants under 6 months of age, a 1000 Hz probe tone is recommended for tympanometry because the ear canal and middle ear system are more compliant at this age.
- B. During sound field behavioral hearing testing, play conditioning can be used to obtain hearing thresholds.
  This is incorrect. Play conditioning is typically used for older children (e.g., 2-3 years and older). For a 5-month-old, behavioral observation audiometry (BOA) or visual reinforcement audiometry (VRA) is more appropriate.
- C. If the difference between electrophysiological testing and minimal response levels (MRL) is 20 dB, the behavioral hearing test results are unreliable.
  This is incorrect. A 20 dB difference between electrophysiological testing (e.g., ABR) and behavioral thresholds is within the expected range and does not necessarily indicate unreliability.
- D. At this stage, electrophysiological testing should be the primary assessment, supplemented by behavioral testing for further management and recommendations.
  This is correct. For infants under 6 months of age, electrophysiological tests (e.g., ABR, ASSR)

are the gold standard for hearing assessment. Behavioral testing can provide supplementary information but is not the primary tool at this age.

Thus, the most appropriate statement is D. At this stage, electrophysiological testing should be the primary assessment, supplemented by behavioral testing for further management and recommendations.

Question 46. 40-year-old Ms. Wang experienced sudden hearing loss that did not improve. Six months later, her audiogram is as shown. Which of the following statements about her electrophysiological test results is least appropriate?

Options:
A. If the patient has recruitment, the absolute latencies of waves I, III, and V in click ABR may be normal.
B. If waves I, III, and V are absent in click ABR, the lesion site cannot be determined.
C. A 500 Hz tone burst ABR will not elicit a wave V response.
D. Acoustic reflexes are absent at all frequencies in both ears.

Correct Answer: C. A 500 Hz tone burst ABR will not elicit a wave V response.

Explanation:

- A. If the patient has recruitment, the absolute latencies of waves I, III, and V in click ABR may be normal.
  This is appropriate. Recruitment is a phenomenon often seen in cochlear hearing loss, where the ABR latencies (waves I, III, and V) can remain normal despite the hearing loss.
- B. If waves I, III, and V are absent in click ABR, the lesion site cannot be determined.
  This is appropriate. The absence of all waves in click ABR suggests severe auditory pathway dysfunction, but it does not localize the lesion to a specific site (cochlea, auditory nerve, or brainstem).
- C. A 500 Hz tone burst ABR will not elicit a wave V response.
  This is least appropriate. A 500 Hz tone burst ABR can elicit a wave V response, especially in cases of low-frequency hearing loss. The tone burst ABR is designed to assess low-frequency hearing, and wave V is typically present if there is residual hearing in that frequency range.
- D. Acoustic reflexes are absent at all frequencies in both ears.
  This is appropriate. In cases of severe or profound hearing loss, acoustic reflexes are often absent because the stimulus is not loud enough to elicit a response.

Thus, the least appropriate statement is C. A 500 Hz tone burst ABR will not elicit a wave V response.

Question 47. A 24-year-old professional baseball player has experienced persistent hearing difficulties in his left ear over the past 6 weeks. The medical history reveals that the issue began after a head injury, where the left side of his head was struck. Otoscopic examination shows a normal and intact tympanic membrane. Audiometry indicates moderate conductive hearing loss in the left ear and normal hearing in

the right ear. Tympanometry shows a Type Ad pattern in the left ear and a Type A pattern in the right ear. Which of the following is the most appropriate diagnosis for the left ear?

Options:
A. Otic hematoma
B. Serous otitis media
C. Temporal bone fracture
D. Ossicular chain disruption

Correct Answer: D. Ossicular chain disruption

Explanation:

- A. Otic hematoma
  This is unlikely because an otic hematoma typically involves blood collection in the external ear (pinna) and does not cause conductive hearing loss with a normal tympanic membrane.
- B. Serous otitis media
  This is unlikely because serous otitis media usually presents with a Type B or Type C tympanogram (indicating fluid in the middle ear) and not a Type Ad pattern.
- C. Temporal bone fracture
  While a temporal bone fracture can cause conductive hearing loss, it is often associated with other symptoms such as facial nerve paralysis, cerebrospinal fluid leakage, or hemotympanum (blood behind the eardrum). The normal otoscopic findings make this less likely.
- D. Ossicular chain disruption
  This is the most appropriate diagnosis. The history of head trauma, moderate conductive hearing loss, and a Type Ad tympanogram (indicating hypermobility of the tympanic membrane, often due to ossicular discontinuity) strongly suggest ossicular chain disruption.

Thus, the most appropriate diagnosis is D. Ossicular chain disruption.

48. Which of the following statements about electrophysiological testing and other hearing tests is most appropriate?

Options:
A. Electrocochleography (ECochG) cannot be used to measure hearing thresholds.
B. Auditory brainstem response (ABR) cannot measure hearing thresholds.
C. Wave I of the ABR is the same as the action potential (AP) in electrocochleography.
D. Auditory steady-state response (ASSR) cannot be used to measure hearing thresholds.

Correct Answer: A. Electrocochleography (ECochG) cannot be used to measure hearing thresholds.

Explanation:

- A. Electrocochleography (ECochG) cannot be used to measure hearing thresholds.
  This is correct. ECochG is primarily used to assess cochlear function (e.g., in Ménière's disease) and is not designed to measure hearing thresholds.

- B. Auditory brainstem response (ABR) cannot measure hearing thresholds.
  This is incorrect. ABR can estimate hearing thresholds, especially in infants and individuals who cannot provide reliable behavioral responses.
- C. Wave I of the ABR is the same as the action potential (AP) in electrocochleography.
  This is incorrect. Wave I of the ABR represents the distal portion of the auditory nerve, while the AP in ECochG is a compound action potential generated by the auditory nerve fibers. They are related but not identical.
- D. Auditory steady-state response (ASSR) cannot be used to measure hearing thresholds.
  This is incorrect. ASSR is specifically used to estimate hearing thresholds, particularly in infants and individuals with hearing loss.

Thus, the most appropriate statement is A. Electrocochleography (ECochG) cannot be used to measure hearing thresholds.

Question 49. Which of the following statements about malingering is incorrect?

Options:
A. Late latency responses (LLR) can help confirm the possibility of malingering.
B. When the difference between pure-tone average (PTA) and speech recognition threshold (SRT) exceeds 15 dB, malingering is one of the possible differential diagnoses.
C. The Stenger test can be used to detect unilateral malingering.
D. Late latency responses (LLR) provide sub-cortical auditory function information.

Correct Answer: D. Late latency responses (LLR) provide sub-cortical auditory function information.

Explanation:

- A. Late latency responses (LLR) can help confirm the possibility of malingering.
  This is correct. LLR, such as the P300 response, can assess cortical auditory processing and help identify inconsistencies in responses that may suggest malingering.
- B. When the difference between pure-tone average (PTA) and speech recognition threshold (SRT) exceeds 15 dB, malingering is one of the possible differential diagnoses.
  This is correct. A significant discrepancy between PTA and SRT (e.g., >15 dB) may indicate non-organic hearing loss, including malingering.
- C. The Stenger test can be used to detect unilateral malingering.
  This is correct. The Stenger test is a behavioral test designed to identify unilateral non-organic hearing loss by exploiting the principle of binaural fusion.
- D. Late latency responses (LLR) provide sub-cortical auditory function information.
  This is incorrect. LLR, such as the P300, reflect cortical auditory processing, not sub-cortical function. Sub-cortical auditory function is assessed using tests like the auditory brainstem response (ABR).

Thus, the incorrect statement is D. Late latency responses (LLR) provide sub-cortical auditory function information.

Question 50. A patient complains of left ear tinnitus and dizziness when exposed to loud sounds. Audiometry shows low-frequency conductive hearing loss in the left ear, normal left ear acoustic reflexes, and enhanced amplitude with reduced threshold for the cervical vestibular evoked myogenic potential (cVEMP) P13-N23 response on the left side. What is the most likely diagnosis?

Options:
A. Ménière's disease
B. Otosclerosis
C. Cholesteatoma
D. Superior canal dehiscence syndrome (SCDS)

Correct Answer: D. Superior canal dehiscence syndrome (SCDS)

Explanation:

- A. Ménière's disease
  Ménière's disease typically presents with sensorineural hearing loss (not conductive), episodic vertigo, tinnitus, and aural fullness. The normal acoustic reflexes and enhanced cVEMP response do not align with Ménière's disease.
- B. Otosclerosis
  Otosclerosis causes conductive hearing loss, but it is typically associated with absent or elevated acoustic reflexes due to stapes fixation. The normal acoustic reflexes and enhanced cVEMP response are inconsistent with otosclerosis.
- C. Cholesteatoma
  Cholesteatoma can cause conductive hearing loss, but it often leads to abnormal acoustic reflexes and other middle ear abnormalities. The normal acoustic reflexes and enhanced cVEMP response do not support this diagnosis.
- D. Superior canal dehiscence syndrome (SCDS)
  SCDS is characterized by conductive hearing loss (often low-frequency), sound- or pressure-induced vertigo, and enhanced cVEMP responses (due to the third window effect). The normal acoustic reflexes and enhanced cVEMP amplitude with reduced threshold are classic findings in SCDS.

Thus, the most likely diagnosis is D. Superior canal dehiscence syndrome (SCDS).

4106

Question 1. Which of the following statements about the Janssen ear mold is least appropriate?

Options:
A. It is also called a CROS ear mold.
B. It is designed to maximize the openness of the ear canal and preserve the natural resonance of the ear canal.
C. Its acoustic and frequency response when connected to a hearing aid is very similar to that of an open ear mold.
D. Its design aims to increase the stability of the fit.

Correct Answer: A. It is also called a CROS ear mold.

Explanation:

- A. It is also called a CROS ear mold.
  This is incorrect. The Janssen ear mold is not related to CROS (Contralateral Routing of Signals) hearing aids. CROS ear molds are used in cases of unilateral hearing loss to route sound from the non-hearing ear to the hearing ear, whereas the Janssen ear mold is designed for open-fit hearing aids.
- B. It is designed to maximize the openness of the ear canal and preserve the natural resonance of the ear canal.
  This is correct. The Janssen ear mold is designed to keep the ear canal as open as possible, allowing for natural sound transmission and resonance.
- C. Its acoustic and frequency response when connected to a hearing aid is very similar to that of an open ear mold.
  This is correct. The Janssen ear mold aims to mimic the acoustic properties of an open ear canal, providing a natural sound experience.
- D. Its design aims to increase the stability of the fit.
  This is correct. While the Janssen ear mold is designed to be open, it also ensures a secure and stable fit for the hearing aid.

Thus, the least appropriate statement is A. It is also called a CROS ear mold.

Question 2. Which of the following statements about the ear impression technique for completely-in-canal (CIC) and high-gain hearing aids is incorrect?

Options:
A. Widening the second bend of the ear canal is particularly important.
B. The ear impression should be taken with the mouth open.
C. The ear impression should extend at least 5 mm beyond the second bend.
D. A particularly hard material can be used after the second bend to increase tightness.

Correct Answer: D. A particularly hard material can be used after the second bend to increase tightness.

Explanation:

- A. Widening the second bend of the ear canal is particularly important.
  This is correct. For CIC and high-gain hearing aids, ensuring proper fit at the second bend of the ear canal is crucial for stability and retention.
- B. The ear impression should be taken with the mouth open.
  This is correct. Taking the impression with the mouth open helps capture the ear canal in its widest and most natural state, ensuring a comfortable and accurate fit.
- C. The ear impression should extend at least 5 mm beyond the second bend.
  This is correct. Extending the impression beyond the second bend ensures proper retention and fit for the hearing aid.
- D. A particularly hard material can be used after the second bend to increase tightness.
  This is incorrect. Using a hard material after the second bend can cause discomfort and irritation. Instead, a flexible or soft material is preferred to ensure comfort while maintaining a secure fit.

Thus, the incorrect statement is D. A particularly hard material can be used after the second bend to increase tightness.

Question 3. Which of the following statements about frequency lowering techniques is most appropriate?

Options:
A. Non-linear frequency compression targets the high-frequency region, and each input frequency has only one corresponding output frequency.
B. Frequency transposition shifts frequencies, and the resulting overlap of low-frequency energy can enhance the recognition of low-frequency speech signals such as semi-vowels and nasals.
C. The principle of frequency lowering is to lower the starting frequency and increase the compression ratio to improve the perception of high-frequency sounds.
D. The benefits of frequency lowering have been fully recognized and can be widely applied to high-frequency hearing losses exceeding 80 dB.

Correct Answer: C. The principle of frequency lowering is to lower the starting frequency and increase the compression ratio to improve the perception of high-frequency sounds.

Explanation:

- A. Non-linear frequency compression targets the high-frequency region, and each input frequency has only one corresponding output frequency.
  This is incorrect. Non-linear frequency compression does target high frequencies, but it compresses a range of high frequencies into a lower range, not a one-to-one mapping.
- B. Frequency transposition shifts frequencies, and the resulting overlap of low-frequency energy can enhance the recognition of low-frequency speech signals such as semi-vowels and nasals.
  This is incorrect. Frequency transposition shifts high-frequency sounds to lower frequencies, but it does not specifically enhance low-frequency speech signals like semi-vowels and nasals.

- C. The principle of frequency lowering is to lower the starting frequency and increase the compression ratio to improve the perception of high-frequency sounds.
  This is correct. Frequency lowering techniques aim to make high-frequency sounds audible by shifting or compressing them into a lower frequency range where hearing is better.
- D. The benefits of frequency lowering have been fully recognized and can be widely applied to high-frequency hearing losses exceeding 80 dB.
  This is incorrect. While frequency lowering can be beneficial, its effectiveness varies among individuals, and it is not universally applicable to all cases of high-frequency hearing loss exceeding 80 dB.

Thus, the most appropriate statement is C. The principle of frequency lowering is to lower the starting frequency and increase the compression ratio to improve the perception of high-frequency sounds.

Question 4. A hearing aid uses linear frequency compression. If a 5000 Hz signal is compressed to 3000 Hz, what will be the output frequency of a 3000 Hz signal?

Options:
A. 1000 Hz
B. 1800 Hz
C. 2000 Hz
D. Insufficient information to determine

Correct Answer: B. 1800 Hz

Explanation:

- Linear frequency compression applies a constant compression ratio across all frequencies.
- If 5000 Hz is compressed to 3000 Hz, the compression ratio is 30005000=0.650003000 =0.6.
- Applying the same ratio to 3000 Hz: 3000×0.6=18003000×0.6=1800 Hz.

Thus, the output frequency of the 3000 Hz signal is B. 1800 Hz.

Question 5. In hearing aid signal processing, which is the only method that effectively improves the signal-to-noise ratio (SNR)?

Options:
A. Using sound bores, vents, or damping systems
B. Multi-channel compression systems
C. Filter systems to adjust frequency response
D. Using directional microphones

Correct Answer: D. Using directional microphones

Explanation:

- A. Using sound bores, vents, or damping systems
  These components affect the acoustic properties of the hearing aid but do not directly improve SNR.
- B. Multi-channel compression systems
  These systems adjust gain based on input levels but do not directly improve SNR.
- C. Filter systems to adjust frequency response
  Filters can shape the frequency response but do not directly improve SNR.
- D. Using directional microphones
  Directional microphones are designed to focus on sounds coming from the front while reducing background noise from other directions, effectively improving SNR.

Thus, the only method that effectively improves SNR is D. Using directional microphones.

Question 6. Which of the following wireless transmission technologies can only be received through the telecoil (T-coil) function of a hearing aid?

Options:
A. Magnetic loops
B. Radio-frequency wireless transmission
C. Infra-red transmission
D. Sound-field amplification

Correct Answer: A. Magnetic loops

Explanation:

- A. Magnetic loops
  This is correct. Magnetic loop systems generate an electromagnetic field that can be picked up by the telecoil (T-coil) in a hearing aid. The T-coil converts the electromagnetic signal into sound, allowing the user to hear the audio directly without background noise.
- B. Radio-frequency wireless transmission
  This is incorrect. Radio-frequency wireless transmission (e.g., Bluetooth) does not require a T-coil. Modern hearing aids with Bluetooth capabilities can receive radio-frequency signals directly.
- C. Infra-red transmission
  This is incorrect. Infra-red transmission systems require a receiver that detects infrared light, not a T-coil.
- D. Sound-field amplification
  This is incorrect. Sound-field amplification systems use speakers to amplify sound in a room and do not require a T-coil for reception.

Thus, the correct answer is A. Magnetic loops.

Question 7. Among the different filter structure designs in hearing aids, the serial-parallel structure is most effective in avoiding which of the following issues?

Options:
A. Irregular gain-frequency response curves
B. Limited frequency response options
C. Insufficient number of signal processing channels
D. Peak clipping due to excessive volume

Correct Answer: A. Irregular gain-frequency response curves

Explanation:

- A. Irregular gain-frequency response curves
  This is correct. The serial-parallel filter structure is designed to provide a smoother and more consistent gain-frequency response by combining multiple filters in series and parallel. This helps avoid irregularities or abrupt changes in the frequency response curve.
- B. Limited frequency response options
  This is incorrect. The serial-parallel structure does not directly address the issue of limited frequency response options. It focuses more on improving the smoothness and accuracy of the frequency response.
- C. Insufficient number of signal processing channels
  This is incorrect. The number of signal processing channels is determined by the hearing aid's design and processing capabilities, not by the filter structure.
- D. Peak clipping due to excessive volume
  This is incorrect. Peak clipping is typically addressed by compression or limiting circuits, not by the filter structure.

Thus, the serial-parallel filter structure is most effective in avoiding A. Irregular gain-frequency response curves.

Question 8. Which of the following statements about bone-anchored hearing aids (BAHA) compared to traditional air-conduction hearing aids is most appropriate?

Options:
A. The larger the air-bone gap, the more effective air-conduction hearing aids are.
B. The larger the air-bone gap, the greater the sensation level provided by air-conduction hearing aids.
C. For individuals with conductive hearing loss and ear infections, bone-anchored hearing aids are preferred.
D. The higher the bone-conduction threshold, the higher the satisfaction with bone-anchored hearing aids.

Correct Answer: C. For individuals with conductive hearing loss and ear infections, bone-anchored hearing aids are preferred.

Explanation:

- A. The larger the air-bone gap, the more effective air-conduction hearing aids are.
  This is incorrect. A larger air-bone gap indicates a greater degree of conductive hearing loss, which may limit the effectiveness of air-conduction hearing aids. Bone-anchored hearing aids (BAHA) are often more effective in such cases.
- B. The larger the air-bone gap, the greater the sensation level provided by air-conduction hearing aids.
  This is incorrect. A larger air-bone gap suggests that air-conduction hearing aids may not provide sufficient amplification, as the issue lies in the conductive pathway. BAHA bypasses the conductive pathway and directly stimulates the cochlea.
- C. For individuals with conductive hearing loss and ear infections, bone-anchored hearing aids are preferred.
  This is correct. BAHA is particularly beneficial for individuals with conductive hearing loss and chronic ear infections, as it avoids the ear canal and middle ear, reducing the risk of infection and discomfort associated with traditional air-conduction hearing aids.
- D. The higher the bone-conduction threshold, the higher the satisfaction with bone-anchored hearing aids.
  This is incorrect. Higher bone-conduction thresholds indicate poorer cochlear function, which may limit the effectiveness of BAHA. Satisfaction with BAHA is generally higher in individuals with good cochlear function and significant conductive hearing loss.

Thus, the most appropriate statement is C. For individuals with conductive hearing loss and ear infections, bone-anchored hearing aids are preferred.

Question 9. Which of the following statements about real-ear measurement (REM) is least appropriate?

Options:
A. Real-ear measurements include real-ear aided gain (REAG) and real-ear insertion gain (REIG), and the difference between them is the real-ear unaided gain (REUG): REAG - REIG = REUG.
B. Measuring real-ear gain is affected by the microphone location effect (MLE), which includes diffraction and resonance from the body, head, pinna, concha, and ear canal.
C. Behind-the-ear (BTE) hearing aids typically produce a greater microphone location effect (MLE) at high frequencies (2-4 kHz) with a 45-degree sound source compared to completely-in-canal (CIC) hearing aids.
D. For the average adult's REUR curve, there should be a peak at 2.2-3.2 kHz.

Correct Answer: A. Real-ear measurements include real-ear aided gain (REAG) and real-ear insertion gain (REIG), and the difference between them is the real-ear unaided gain (REUG): REAG - REIG = REUG.

Explanation:

- A. Real-ear measurements include real-ear aided gain (REAG) and real-ear insertion gain (REIG), and the difference between them is the real-ear unaided gain (REUG): REAG - REIG = REUG.
  This is incorrect. The correct relationship is REIG = REAG - REUG, not REAG - REIG = REUG. REIG represents the gain provided by the hearing aid after accounting for the natural resonance of the ear (REUG).
- B. Measuring real-ear gain is affected by the microphone location effect (MLE), which includes diffraction and resonance from the body, head, pinna, concha, and ear canal.
  This is correct. The microphone location effect (MLE) influences the measurement of real-ear gain due to the interaction of sound with the body and ear structures.
- C. Behind-the-ear (BTE) hearing aids typically produce a greater microphone location effect (MLE) at high frequencies (2-4 kHz) with a 45-degree sound source compared to completely-in-canal (CIC) hearing aids.
  This is correct. BTE hearing aids, due to their external placement, are more susceptible to MLE at high frequencies compared to CIC hearing aids, which are placed deeper in the ear canal.
- D. For the average adult's REUR curve, there should be a peak at 2.2-3.2 kHz.
  This is correct. The real-ear unaided response (REUR) curve typically shows a peak in the 2.2-3.2 kHz range due to the natural resonance of the ear canal.

Thus, the least appropriate statement is A. Real-ear measurements include real-ear aided gain (REAG) and real-ear insertion gain (REIG), and the difference between them is the real-ear unaided gain (REUG): REAG - REIG = REUG.

Question 10. When hearing loss is concentrated in the high-frequency region, which of the following methods can be used to increase the high-frequency gain of a hearing aid?

Options:
A. Gradually increase the diameter of the sound bore.
B. Add a vent.
C. Change the internal diameter of the earmold tubing.
D. Extend the length of the earmold.

Correct Answer: A. Gradually increase the diameter of the sound bore.

Explanation:

- A. Gradually increase the diameter of the sound bore.
  This is correct. Increasing the diameter of the sound bore reduces the high-frequency attenuation caused by the ear canal resonance, allowing more high-frequency sounds to reach the eardrum. This can improve high-frequency gain.
- B. Add a vent.
  This is incorrect. Adding a vent typically reduces low-frequency amplification and can increase the risk of feedback, but it does not directly improve high-frequency gain.

- C. Change the internal diameter of the earmold tubing.
  This is incorrect. Changing the tubing diameter primarily affects the overall gain and frequency response but does not specifically target high-frequency gain.
- D. Extend the length of the earmold.
  This is incorrect. Extending the length of the earmold can affect the fit and comfort but does not directly increase high-frequency gain.

Thus, the correct method to increase high-frequency gain is A. Gradually increase the diameter of the sound bore.

Question 11. What is the most appropriate compression ratio for expansion technology used in digital hearing aids?

Options:
A. 1:1
B. 1:2
C. 2:1
D. 10:1

Correct Answer: A. 1:1

Explanation:

- Expansion technology is used to reduce low-level noise by applying negative gain to sounds below a certain threshold.
- A 1:1 compression ratio means no compression is applied, which is typical for expansion. Expansion effectively "turns down" very soft sounds, making them even quieter, while leaving louder sounds unchanged.
- Ratios like 1:2, 2:1, or 10:1 are used for compression (amplifying or limiting sounds), not for expansion.

Thus, the most appropriate compression ratio for expansion technology is A. 1:1.

Question 12. According to Desjardins (2016), how do directional microphones and digital noise reduction affect cognitive effort when listening to speech in noise?

Options:
A. Only directional microphones significantly reduce cognitive effort when listening in noise.
B. Only digital noise reduction significantly reduces cognitive effort when listening in noise.
C. Both directional microphones and digital noise reduction significantly reduce cognitive effort when listening in noise.
D. Neither directional microphones nor digital noise reduction significantly reduce cognitive effort when listening in noise.

Correct Answer: C. Both directional microphones and digital noise reduction significantly reduce cognitive effort when listening in noise.

Explanation:

- Directional microphones focus on sounds coming from the front while reducing background noise from other directions, improving the signal-to-noise ratio (SNR) and reducing the cognitive effort required to understand speech.
- Digital noise reduction algorithms reduce the perceived level of background noise, making it easier to focus on speech and further reducing cognitive effort.
- Desjardins (2016) found that both technologies work synergistically to reduce cognitive effort in noisy listening environments.

Thus, the correct statement is C. Both directional microphones and digital noise reduction significantly reduce cognitive effort when listening in noise.

Question 13:
According to the ANSI S3.22 standard, when defining the frequency range of a hearing aid, it refers to the range where the gain value is 20 dB below which of the following?

Answer: B High-frequency average gain (HFA)

Explanation:
The ANSI S3.22 standard defines the frequency range of a hearing aid as the range where the gain value is 20 dB below the high-frequency average gain (HFA). HFA is the average gain measured at specific high frequencies (typically 1000 Hz, 1600 Hz, and 2500 Hz). This standard ensures that the hearing aid's gain performance across different frequencies meets specifications and provides adequate amplification.

Question 14:
Regarding hearing aid technology in noisy environments, which of the following statements is most appropriate?

Answer: D Hearing aids often use first-order subtraction processing with two omnidirectional microphones to create a directional effect, improving speech clarity in noise.

Explanation:
In noisy environments, hearing aids typically employ directional microphone technology to enhance speech clarity. Directional microphones use two omnidirectional microphones and apply first-order subtraction processing to amplify sounds coming from the front (usually speech) while reducing noise from other directions. This technology effectively improves speech recognition in noisy settings without significantly compromising speech clarity.

Question 15:
A hearing aid user complains that their own voice sounds different when wearing the hearing aid. Which of the following is least likely to be the cause?

Answer: D The hearing aid excessively amplifies high-frequency sounds.

Explanation:
When a hearing aid user feels that their own voice sounds different, it is often related to excessive amplification of low-frequency sounds (Option B) or occlusion caused by the ear mold (Option C). Over-amplification of low frequencies can make the user's voice sound boomy or hollow, while occlusion can create a "plugged-up" sensation. Option A (the user forgetting their natural voice characteristics) is possible but less common. Option D (excessive amplification of high-frequency sounds) is least likely to be the cause because over-amplification of high frequencies typically affects the perception of external high-pitched sounds rather than altering the user's perception of their own voice.

Question 16:
Regarding the issue mentioned above, which of the following is not a solution?

Answer: D Reduce the size of the existing vent

Explanation:
If a hearing aid user experiences the occlusion effect (e.g., their own voice sounds hollow or boomy), the issue is typically caused by a tightly sealed ear mold or ear tip. Common solutions include:

- A Adding a vent: This reduces the occlusion effect by allowing low-frequency sounds to escape.
- B Using an open-fit ear tip: This minimizes the occlusion effect by reducing the seal in the ear canal.
- C Using a softer ear mold material: This can improve comfort and reduce the perception of occlusion.

However, D Reducing the size of the existing vent would worsen the occlusion effect by further trapping low-frequency sounds, making the user's voice sound even more unnatural. Therefore, this is not a recommended solution.

Question 17:
Many individuals with hearing loss also experience tinnitus. Since high-frequency sounds are most effective at masking tinnitus, which type of hearing aid is most recommended for patients with mild high-frequency sloping hearing loss and tinnitus?

Answer: B Open fitting

Explanation:
For patients with mild high-frequency sloping hearing loss and tinnitus, open fitting hearing aids are often recommended. This is because:

- Open fittings allow natural low-frequency sounds to enter the ear, reducing the occlusion effect.
- They amplify high-frequency sounds effectively, which helps mask tinnitus (since high-frequency sounds are most effective at masking tinnitus).
- They provide a more natural sound quality and are less likely to cause discomfort.

Other options like A In-the-ear hearing aids or C Completely-in-the-canal hearing aids may not be as effective for high-frequency hearing loss and tinnitus due to their design limitations. D Behind-the-ear hearing aids can also work, but open fittings are specifically tailored for this type of hearing loss and tinnitus.

Question 18:
For which of the following cases is the signal-to-noise ratio (SNR) least important when fitting hearing aids?

Answer: A Otosclerosis

Explanation:
Signal-to-noise ratio (SNR) is a critical factor in hearing aid fitting for individuals who struggle to hear in noisy environments. However, A Otosclerosis primarily affects the middle ear, causing conductive hearing loss. These patients typically have better speech understanding in noise compared to those with sensorineural hearing loss, as their cochlear function (inner ear) is often preserved. Therefore, SNR is less of a concern for otosclerosis patients.

In contrast:

- B Presbycusis (age-related hearing loss): Age-related hearing loss often involves difficulty understanding speech in noise, making SNR a critical consideration.
- C Auditory neuropathy spectrum disorder: These patients have significant difficulty with speech perception, especially in noisy environments, so SNR is highly important.
- D Ménière’s disease: Fluctuating hearing loss and sensitivity to loud sounds make SNR an important factor in hearing aid fitting.

Thus, A Otosclerosis is the condition where SNR is least relevant.

Question 19:

Answer: C. 呈現聽覺皮質誘發電位（CAEP）

Explanation:
Auditory neuropathy spectrum disorder (ANSD) is characterized by normal outer hair cell function (as evidenced by present OAEs) but abnormal neural transmission, which can affect the auditory nerve or higher auditory pathways. Pure-tone thresholds (A) and ABR wave V latency (B) are not reliable predictors of hearing aid benefit in ANSD because they do not directly reflect cortical processing or functional hearing ability. OAE responses (D) only confirm outer hair cell function and do not provide information about neural synchrony or auditory processing.

Cortical auditory evoked potentials (CAEP), however, reflect the brain's ability to process sound at the cortical level. If CAEPs are present, it suggests that the auditory cortex is receiving and processing sound, which is a strong predictor of potential benefit from hearing aids. Therefore, CAEPs are the most relevant measure to determine if a child with ANSD might benefit from hearing aids.

Question 20:

Answer: B. SII 愈高代表該助聽器的設定愈合適嬰幼兒的增益量需求

Explanation:
The Speech Intelligibility Index (SII) quantifies the proportion of the speech signal that is audible to a listener, based on their hearing thresholds and the amplification provided by a hearing aid. A higher SII indicates that more of the speech signal is accessible to the listener, which suggests that the hearing aid settings are better matched to the individual's hearing loss and gain requirements.

- A is incorrect because SII is not limited to predicting performance in noisy environments at specific volumes; it is a general measure of speech audibility.
- C is incorrect because SII is not directly comparable between adults and infants due to differences in auditory development and listening needs.
- D is partially correct, but the primary clinical significance of SII is to evaluate the appropriateness of hearing aid settings for an individual, not to compare different hearing aids.

Thus, B is the most appropriate answer, as it directly relates SII to the suitability of hearing aid gain for infants and young children.

Question 21:

Answer: D. ①②③

Explanation:
In real-ear measurement systems, the type of earphone used (insert vs. supra-aural) affects the calibration and reference values for hearing thresholds, gain targets, and real-ear aided response (REAR).

1. Hearing thresholds (①): The conversion from dB HL to dB SPL depends on the earphone type because different earphones have different reference equivalent threshold sound pressure levels

(RETSPLs). Changing the earphone type will alter the SPL values corresponding to the hearing thresholds.

2. Gain formula target values (②): The target gain values are calculated based on the hearing thresholds and the earphone type. If the earphone type changes, the target gain values will also change.
3. Real-ear aided response (REAR) (③): The REAR is measured in the ear canal and is influenced by the hearing aid's output, which is calibrated based on the earphone type. Changing the earphone type will affect the REAR.

Therefore, all three items (hearing thresholds, gain formula target values, and REAR) are likely to change when switching from insert earphones to supra-aural earphones. The correct answer is D. ①②③.

Question 22:

Answer: C. 對 3000 Hz 量測結果造成的誤差不超出 3 dB

Explanation:
In real-ear measurements, the placement of the probe tube relative to the eardrum is critical for accurate results. If the probe tube is not placed close enough to the eardrum, high-frequency measurements (above 2000 Hz) can be underestimated due to standing wave effects. The question states that the probe tube is 1.6 cm from the eardrum, while the earmold tip is 2.0 cm from the eardrum, leaving a 0.4 cm gap.

- A is incorrect because low frequencies (e.g., 500 Hz) are less affected by probe tube placement errors, but this does not mean there is no error at all.
- B is incorrect because 2000 Hz is still within the range where small placement errors can cause measurable inaccuracies, though the error may be minimal.
- C is correct because 3000 Hz is a high frequency where probe tube placement errors can lead to underestimation, but the error is typically within 3 dB if the placement is within 0.4 cm of the ideal position.
- D is incorrect because 4000 Hz is even more sensitive to placement errors, and the error could exceed 5 dB in this case.

Thus, C is the most reasonable answer, as the error for 3000 Hz is likely to be within 3 dB.

Question 23:

Answer: B. 具耳蝸死區（dead region）之聽損者

Explanation:
Off-frequency listening occurs when a listener relies on frequencies adjacent to the target frequency because the auditory system cannot effectively process the target frequency. This phenomenon is most commonly observed in individuals with cochlear dead regions, where the hair cells at specific frequencies

are non-functional. In such cases, the brain may "shift" its attention to nearby frequencies where hearing is still intact.

- A (auditory neuropathy) is incorrect because off-frequency listening is not a hallmark of auditory neuropathy, which primarily affects neural synchrony rather than frequency-specific processing.
- C (CAPD) is incorrect because central auditory processing disorder (CAPD) affects higher-level auditory processing, not frequency-specific hearing loss.
- D (conductive hearing loss) is incorrect because conductive hearing loss does not typically lead to off-frequency listening, as it affects sound transmission rather than cochlear function.

Thus, B is the correct answer, as cochlear dead regions are most strongly associated with off-frequency listening.

Question 24:

Answer: B. NAL-NL2

Explanation:
The NAL-NL2 (National Acoustic Laboratories Nonlinear, Version 2) hearing aid fitting formula is designed to optimize speech intelligibility and loudness comfort for a wide range of hearing losses. It takes into account the specific characteristics of tonal languages (e.g., Mandarin Chinese) and non-tonal languages (e.g., English) by adjusting the amplification strategy to preserve pitch cues, which are critical for understanding tonal languages.

- A (FIG 6) is incorrect because FIG 6 is not a widely recognized hearing aid fitting formula and does not specifically address tonal languages.
- C (LGOB) is incorrect because LGOB (Libby Horn Gobble) is an older fitting method and does not incorporate considerations for tonal languages.
- D (DSL) is incorrect because the DSL (Desired Sensation Level) formula, while widely used, does not explicitly differentiate between tonal and non-tonal languages in its algorithm.

Thus, B (NAL-NL2) is the correct answer, as it is the only formula listed that specifically accounts for the differences between tonal and non-tonal languages.

Question 25:

Answer: A. REAR

Explanation:
Real-ear aided response (REAR) measures the sound pressure level in the ear canal when a hearing aid is worn and functioning. During REAR measurements, the hearing aid is turned on and amplifying sound,

which increases the risk of feedback (whistling) due to sound leakage from the ear canal. Feedback can distort the measurement results, so it is critical to monitor and manage feedback during REAR testing.

- B (RECD): Real-ear-to-coupler difference (RECD) measures the difference between sound pressure levels in the ear canal and a coupler. It is typically measured with the hearing aid turned off, so feedback is not a concern.
- C (REDD): Real-ear-to-dial difference (REDD) measures the difference between sound pressure levels in the ear canal and the hearing test dial level. Feedback is not a concern here because the hearing aid is not active during this measurement.
- D (REOR): Real-ear occluded response (REOR) measures the sound pressure level in the ear canal with the hearing aid in place but turned off. Since the hearing aid is not amplifying sound, feedback is not an issue.

Thus, A (REAR) is the correct answer because it is the only measurement that involves an active hearing aid, making feedback a potential concern.

Question 26:

Answer: A. RECD＝REDD＋參考等閾聲壓值（reference equivalent threshold SPL, RETSPL）

Explanation:
The real-ear-to-coupler difference (RECD) and real-ear-to-dial difference (REDD) are both measures used in hearing aid fitting, but they are not directly related through the addition of RETSPL. Let's evaluate each option:

- A (Incorrect): RECD is not calculated as REDD plus RETSPL. RECD is the difference between the sound pressure level (SPL) in the ear canal and the SPL in a coupler, while REDD is the difference between the SPL in the ear canal and the hearing test dial level (in dB HL). These two measures are independent and do not involve RETSPL in their relationship.
- B (Correct): REDD is calculated as the coupler SPL minus the hearing test dial level (in dB HL). This is a valid definition of REDD.
- C (Correct): RECD is calculated as the ear canal SPL minus the coupler SPL. This is the standard definition of RECD.
- D (Correct): Coupler SPL is calculated as the hearing test dial level (in dB HL) plus RETSPL. This is a valid conversion between dB HL and dB SPL.

Thus, A is the incorrect statement, as it misrepresents the relationship between RECD and REDD.

Question 27:

Answer: B. 客戶導向進步量表（Client Oriented Scale of Improvement, COSI）

Explanation:
The Client Oriented Scale of Improvement (COSI) is specifically designed to address the limitation described in the question. Unlike other questionnaires that use predefined questions, COSI allows patients to identify and prioritize their own specific listening situations where they experience difficulty. This personalized approach ensures that the questionnaire is directly relevant to the patient's unique needs and goals.

- A (APHAB): The Abbreviated Profile of Hearing Aid Benefit (APHAB) uses fixed questions about common listening situations, which may not align with the patient's specific concerns.
- C (SSQ): The Speech, Spatial, and Qualities of Hearing Scale (SSQ) assesses a wide range of listening scenarios but does not allow patients to customize the questions to their specific needs.
- D (EAR): The Effectiveness of Auditory Rehabilitation (EAR) is not a widely recognized or standardized questionnaire for hearing aid evaluation.

Thus, B (COSI) is the best choice because it directly addresses the issue of relevance by allowing patients to focus on their specific listening challenges.

Question 28:

Answer: A. 此問卷探討了助聽器使用者的使用狀況、選配效益（活動限制的改善）、滿意度、選配後仍殘存的活動限制、社交剝奪、其他影響與生活品質的改善等七大領域

Explanation:
The International Outcomes Inventory for Hearing Aids (IOI-HA) is a widely used questionnaire designed to evaluate the effectiveness of hearing aids across seven key domains:

1. Use: How often the hearing aid is used.
2. Benefit: Improvement in activity limitations.
3. Residual activity limitations: Remaining difficulties after hearing aid use.
4. Satisfaction: Overall satisfaction with the hearing aid.
5. Residual participation restrictions: Remaining social or participation challenges.
6. Impact on others: How hearing aid use affects others (e.g., family, friends).
7. Quality of life: Overall improvement in quality of life.

- B is incorrect because the IOI-HA does not include sub-questions for each of the seven items; it consists of seven standalone questions.
- C is incorrect because the scoring for each question ranges from 1 to 5, not 1 to 7.
- D is incorrect because the IOI-HA does not categorize its questions into two distinct "faces" or directly correlate scores with hearing thresholds in the way described.

Thus, A is the correct answer, as it accurately describes the seven domains covered by the IOI-HA.

Question 29:

Answer: C. 兒童居家聆聽困難清單（Children’s home inventory of listening difficulties, CHILD）適用於 3～12 歲的孩童，題目共 15 題

Explanation:
The Children’s Home Inventory of Listening Difficulties (CHILD) is a questionnaire designed to assess listening challenges in children aged 3–12 years. It consists of 15 questions that evaluate how well a child performs in various listening situations at home. This makes it a suitable tool for identifying specific listening difficulties in children within this age range.

- A (COSI-C): The Client Oriented Scale of Improvement for Children (COSI-C) is not a standardized or widely recognized questionnaire, and the description provided (10 questions for children over 5 years old) is inaccurate.
- B (MAIS): The Meaningful Auditory Integration Scale (MAIS) is designed for very young children (0.5–3 years old) to assess their ability to integrate auditory information into daily life. However, it does not consist of 10 questions; it typically includes fewer items.
- D (APHAP-C): The Abbreviated Profile of Hearing Aid Performance for Children (APHAP-C) is not a recognized or standardized questionnaire, and the description provided (24 questions for children aged 5–10 years) is incorrect.

Thus, C (CHILD) is the correct answer, as it accurately describes the age range and number of questions for this questionnaire.

Question 30:

Answer: A. 助聽器效果簡易評估表（Abbreviated Profile of Hearing Aid Benefit, APHAB）

Explanation:
The Abbreviated Profile of Hearing Aid Benefit (APHAB) is a widely used questionnaire that compares a patient's listening experiences before and after hearing aid use. It consists of 24 questions divided into four subscales: ease of communication, reverberation, background noise, and aversiveness. Patients rate their difficulty in various listening situations both with and without hearing aids, allowing for a direct comparison of performance.

- B (HAR): The Hearing Aid Review (HAR) is a questionnaire that assesses overall satisfaction with hearing aids but does not explicitly compare pre- and post-fitting performance.
- C (SHAPIE): The Shortened Hearing Aid Performance Inventory for the Elderly (SHAPIE) evaluates hearing aid performance in elderly users but does not provide a direct comparison of pre- and post-fitting experiences.

- D (HAPI): The Hearing Aid Performance Inventory (HAPI) assesses hearing aid performance in specific listening situations but does not include a pre-fitting assessment for comparison.

Thus, A (APHAB) is the correct answer, as it is specifically designed to compare listening experiences before and after hearing aid use.

Question 31:

Answer: B. 使用 90 dB SPL 的語音輸入音

Explanation:
The ANSI S3.22 standard specifies the procedures for testing hearing aids, including the measurement of OSPL90 (Output Sound Pressure Level for a 90 dB SPL input). The OSPL90 test is designed to measure the maximum output of a hearing aid when a 90 dB SPL pure tone or noise signal is used as the input.

- A (Correct): The hearing aid's volume control must be set to the full-on position to ensure the maximum output is measured.
- B (Incorrect): The input signal for OSPL90 testing is not a speech signal but rather a pure tone or broadband noise at 90 dB SPL. Using a speech signal would not provide a standardized measurement of the hearing aid's maximum output.
- C (Correct): The OSPL90 test is used to estimate the hearing aid's maximum output level, which is critical for ensuring the device does not exceed safe loudness levels for the user.
- D (Correct): The high-frequency average (HFA) OSPL90 value must be within 4 dB SPL of the manufacturer's specifications to ensure compliance with ANSI standards.

Thus, B is the incorrect statement because the input signal for OSPL90 testing is not a speech signal.

Question 32:

Answer: B. 提升低頻的時間解析度和頻譜解析度

Explanation:
Fine Structure Processing (FSP) is a sound coding strategy used in cochlear implants to improve the representation of fine temporal and spectral details in the signal, particularly in the low-frequency range. This strategy is especially beneficial for enhancing pitch perception and music appreciation, as well as improving speech understanding in noise.

- A (Incorrect): FSP does not uniformly improve temporal resolution across all frequency bands; it specifically targets low-frequency regions.
- B (Correct): FSP enhances both temporal and spectral resolution in the low-frequency range, which is critical for perceiving pitch and prosody in speech and music.

- C (Incorrect): FSP does not solely improve frequency resolution; it also improves temporal resolution, particularly in low frequencies.
- D (Incorrect): FSP does not increase the number of virtual stimulation channels; it improves the quality of information within existing channels.

Thus, B is the correct answer, as FSP primarily enhances temporal and spectral resolution in the low-frequency range.

Question 33:

Answer: D. 理論上能提供更多語音隨著時間變化的細節

Explanation:
HiResolution (HiRes) sound processing is a strategy used in cochlear implants to provide higher temporal and spectral resolution compared to older strategies like Continuous Interleaved Sampling (CIS). HiRes aims to deliver more detailed information about the temporal fine structure of sounds, which is particularly important for understanding speech in noise and appreciating music.

- A (Incorrect): HiRes does not necessarily provide more electrodes than CIS, and it typically uses higher stimulation rates, not lower ones.
- B (Incorrect): HiRes does not use lower cutoff frequencies; it focuses on preserving high-frequency details and temporal fine structure.
- C (Incorrect): HiRes does not sacrifice the number of simultaneously active electrodes due to high stimulation rates. Instead, it optimizes the timing and delivery of electrical pulses.
- D (Correct): HiRes is designed to provide more detailed temporal information, which helps users perceive subtle changes in speech and environmental sounds over time.

Thus, D is the most appropriate answer, as HiRes aims to enhance the temporal details of speech and other sounds.

Question 34:

Answer: B. 頻域（frequency domain）

Explanation:
Current steering is a technology used in cochlear implants to create virtual channels between physical electrodes, allowing for finer frequency resolution. By steering electrical currents between adjacent electrodes, the system can stimulate intermediate regions of the cochlea, effectively increasing the number of frequency bands available for sound coding.

- A (Incorrect): Current steering does not primarily address intensity domain coding, which relates to loudness perception.

- B (Correct): Current steering is used to enhance frequency resolution by creating virtual channels in the frequency domain.
- C (Incorrect): Current steering does not directly manipulate time-domain signals, such as temporal fine structure.
- D (Incorrect): Current steering is not related to spatial domain processing, which involves the localization of sounds in space.

Thus, B is the correct answer, as current steering is used to improve frequency resolution in cochlear implants.

Question 35:

Answer: C. 100%可偵測的最小電刺激量

Explanation:
The electrical threshold (T-level) in cochlear implants, including Cochlear's Nucleus system, is defined as the minimum level of electrical stimulation required for the user to detect the stimulus 100% of the time. This threshold is critical for setting the lower boundary of the dynamic range for each electrode.

- A (Incorrect): The T-level is not related to comfort levels (C-level), which represent the maximum comfortable stimulation level.
- B (Incorrect): The T-level is not defined as a 50% detection threshold; it is the level at which the stimulus is detectable 100% of the time.
- C (Correct): The T-level is the minimum electrical stimulation level that the user can reliably detect.
- D (Incorrect): The T-level is not related to speech discrimination or the ability to distinguish between two speech sounds.

Thus, C is the correct answer, as the T-level represents the minimum detectable electrical stimulation.

Question 36:

Answer: A. 對應耳蝸的自然頻率排列（natural tonotopic organization）

Explanation:
The cochlea has a natural tonotopic organization, where high-frequency sounds are processed at the base of the cochlea and low-frequency sounds are processed at the apex. Multi-electrode cochlear implants are designed to mimic this natural arrangement by delivering high-frequency signals to electrodes at the base of the cochlea and low-frequency signals to electrodes near the apex. This alignment helps preserve the natural frequency-to-place mapping, which is critical for sound perception and speech understanding.

- B (Incorrect): While minimizing electrical field interference is important in cochlear implant design, it is not the primary reason for aligning frequencies with specific electrode locations.
- C (Incorrect): Cochlear implants use pulsatile stimulation rather than analog signals, so this is not relevant to the frequency-to-place mapping.
- D (Incorrect): While unnatural sound quality can affect speech recognition, the primary goal of frequency-to-place mapping is to align with the cochlea's natural tonotopic organization.

Thus, A is the correct answer, as the frequency-to-place mapping in cochlear implants is designed to correspond to the cochlea's natural tonotopic organization.

Question 37:

Answer: B. 腦膜炎引起耳蝸鈣化

Explanation:
A hearing aid trial is typically required before cochlear implantation to determine if hearing aids can provide sufficient benefit. However, in cases of meningitis-induced cochlear ossification (calcification), the cochlea may become progressively obstructed, making it difficult or impossible to insert the electrode array later. In such cases, immediate cochlear implantation is often recommended to avoid delays that could compromise the surgical outcome.

- A (Incorrect): Sudden hearing loss does not necessarily preclude a hearing aid trial, as some patients may still benefit from hearing aids.
- C (Incorrect): Hearing loss following cholesteatoma surgery does not automatically justify skipping a hearing aid trial.
- D (Incorrect): Hearing loss due to stroke does not typically require immediate cochlear implantation without a hearing aid trial.

Thus, B is the correct answer, as cochlear ossification from meningitis is a medical urgency that may require immediate implantation.

Question 38:

Answer: C. 使用人工耳蝸再搭配 HAT，對於在噪音中聆聽語音沒有幫助

Explanation:
Hearing assistance technology (HAT), such as FM systems and telecoils, can significantly improve speech understanding in noisy environments for cochlear implant users. These technologies work by delivering clear, direct audio signals to the implant, reducing the impact of background noise and distance.

- A (Correct): Telecoils can be used with cochlear implants to connect to compatible hearing loop systems, improving accessibility in public venues.

- B (Correct): FM systems can be connected to cochlear implants via adapters, providing enhanced speech clarity in challenging listening situations.
- C (Incorrect): This statement is false. HAT, when used with cochlear implants, is highly effective in improving speech understanding in noise.
- D (Correct): When connecting HAT to cochlear implants, it is important to adjust the audio-mixing ratios to balance the direct signal from the HAT with environmental sounds.

Thus, C is the most inappropriate statement, as HAT is known to improve speech perception in noise for cochlear implant users.

Question 39:

Answer: C. 當某些電極必須關閉時，應特別考慮頻率分配

Explanation:
Frequency allocation in cochlear implants refers to how specific frequency ranges are assigned to individual electrodes. When certain electrodes need to be deactivated (e.g., due to non-functioning electrodes or discomfort), it is crucial to reconsider the frequency allocation to ensure that the remaining electrodes cover the necessary frequency range effectively. This helps maintain optimal speech perception and sound quality.

- A (Incorrect): Infants and young children typically require a wider frequency allocation range to support language development, not a narrower one.
- B (Incorrect): Wider channel bandwidths can reduce spectral resolution, which may negatively impact speech understanding. Optimal bandwidths are carefully determined based on individual needs.
- D (Incorrect): The frequency allocation range for adults is not limited to 4000 Hz; it typically covers a broader range (e.g., 100–8000 Hz) to ensure access to speech and environmental sounds.

Thus, C is the most appropriate answer, as frequency allocation must be carefully adjusted when electrodes are deactivated.

Question 40:

Answer: A. frequency allocation

Explanation:
The intensity domain in cochlear implant mapping refers to the loudness of the electrical stimulation, which is determined by parameters such as stimulation levels, threshold levels (T-levels), and upper-stimulation levels (C-levels). Frequency allocation, however, determines how frequencies are assigned to specific electrodes and does not directly affect the intensity or loudness of the stimulation.

- B (Incorrect): Stimulation levels directly influence the intensity domain by determining the strength of the electrical signal.
- C (Incorrect): The threshold of stimulation (T-level) defines the minimum level of electrical stimulation required for detection, which is part of the intensity domain.
- D (Incorrect): Upper-stimulation levels (C-levels) define the maximum comfortable level of stimulation, which is also part of the intensity domain.

Thus, A is the correct answer, as frequency allocation does not affect the intensity domain.

Question 41:

Answer: D. electrode impedance 測試

Explanation:
Before performing any programming or mapping of a cochlear implant, it is essential to conduct an electrode impedance test. This test checks the integrity of the electrode array and ensures that all electrodes are functioning properly. If an electrode has abnormal impedance (e.g., short or open circuit), it may need to be deactivated or adjusted during mapping.

- A (Incorrect): T-level testing is performed after impedance testing to determine the minimum detectable stimulation levels.
- B (Incorrect): C-level testing is performed after impedance testing to determine the maximum comfortable stimulation levels.
- C (Incorrect): Loudness balancing is performed after T- and C-level testing to ensure consistent loudness across electrodes.

Thus, D is the correct answer, as electrode impedance testing is the first step in cochlear implant programming.

Question 42:

Answer: A. 頻率分配（frequency allocation）

Explanation:
The stimulation rate in cochlear implants refers to how quickly electrical pulses are delivered to the electrodes. Changing the stimulation rate primarily affects temporal resolution and the perception of sound timing but does not directly influence frequency allocation, which is determined by the assignment of frequency ranges to specific electrodes.

- B (Incorrect): T-levels (behavioral threshold levels) may be affected by changes in stimulation rate, as the perception of loudness can vary with different rates.

- C (Incorrect): C-levels (maximum comfortable levels) may also be affected by changes in stimulation rate, as higher rates can influence perceived loudness.
- D (Incorrect): The dynamic range (the difference between T-levels and C-levels) can be influenced by changes in stimulation rate, as both T- and C-levels may shift.

Thus, A is the correct answer, as frequency allocation is independent of stimulation rate changes.

*Image was provided but DeepSeek could not process the diagram to answer the question

Question 43: The question refers to a diagram that is not provided in the text. However, based on the context, pulse width in cochlear implants typically refers to the duration of each electrical pulse delivered by the electrodes. This duration is crucial for determining the intensity and perception of the stimulus.

To answer the question accurately, the diagram would need to be analyzed to identify which part represents the pulse width. Generally, pulse width is measured from the start to the end of a single pulse in the waveform.

If you can provide or describe the diagram, I can give a more specific answer. Otherwise, based on typical representations:

- A (①): If this represents the start of the pulse.
- B (②): If this represents the end of the pulse.
- C (③): If this represents the duration of the pulse.
- D (④): If this represents another aspect of the waveform.

Without the diagram, the most likely answer is C (③), as it typically represents the duration or width of the pulse.

Question 44:

Answer: B. 將麥克風與發射器佩戴於領口處會比夾在腰間來得理想

Explanation:
The placement of the FM microphone and transmitter is crucial for optimal sound quality and speech clarity. Wearing the microphone and transmitter at the collar or lapel area is generally more effective than placing it at the waist because:

- A (Incorrect): Head-worn microphones typically provide better sound quality than lapel microphones because they are closer to the mouth and less affected by body movements or environmental noise.

- B (Correct): Placing the microphone and transmitter at the collar or lapel ensures that the microphone is closer to the speaker's mouth, reducing the distance sound must travel and minimizing background noise interference.
- C (Incorrect): Built-in microphones in transmitters should not be worn inside clothing, as this can muffle sound and reduce clarity.
- D (Incorrect): FM microphones often have directional designs to focus on the speaker's voice, and they can be worn in various positions, not just on the collar.

Thus, B is the most appropriate answer, as collar placement optimizes sound capture and clarity.

Question 45:

Answer: A. **比助聽器**內的任何信號處理方式更能改善信號品質，提高語音清晰度

Explanation:
FM systems are designed to improve speech clarity by transmitting the speaker's voice directly to the listener's hearing aid or cochlear implant, reducing the impact of distance and background noise.

- A (Correct): FM systems can significantly improve signal quality and speech clarity compared to internal hearing aid processing, especially in noisy environments or over distances.
- B (Incorrect): FM systems are beneficial for both children and adults, particularly in challenging listening situations like classrooms or meetings.
- C (Incorrect): Multiple transmitters can be used in FM systems to accommodate different speakers or environments, enhancing flexibility and usability.
- D (Incorrect): FM systems do not need to be used all day; they are typically used in specific situations where improved speech clarity is needed, such as in classrooms or during meetings.

Thus, A is the most appropriate answer, as FM systems are highly effective in improving signal quality and speech clarity.

Question 46:

Answer: C. **殘響半徑不因教師授課音量而改變**

Explanation:
The reverberation radius (critical distance) in a classroom is the distance from the sound source (e.g., the teacher) at which the direct sound and reverberant sound are equal in intensity. This radius is primarily determined by the room's acoustics, such as its size, shape, and surface materials, rather than the volume of the teacher's voice.

- A (Incorrect): The reverberation radius is influenced by the room's dimensions, including ceiling height, as it affects the overall reverberation characteristics.
- B (Incorrect): The reverberation radius is not used to calculate reverberation time; instead, it is a measure of the balance between direct and reverberant sound.
- C (Correct): The reverberation radius is determined by the room's acoustic properties and is independent of the teacher's speaking volume.
- D (Incorrect): The reverberation radius can vary with frequency because higher frequencies are more easily absorbed by surfaces, while lower frequencies may have a larger radius due to their longer wavelengths.

Thus, C is the most appropriate answer, as the reverberation radius is not affected by the teacher's speaking volume.

Question 47:

Answer: C. 測試行動電話，也測試助聽器

Explanation:
The ANSI/IEEE C63.19 standard evaluates the compatibility between hearing aids and wireless communication devices, such as mobile phones. This standard tests both the hearing aid and the mobile phone to ensure they can operate together without causing electromagnetic interference (EMI) or other issues.

- A (Incorrect): The standard does not test only mobile phones; it also evaluates hearing aids.
- B (Incorrect): The standard does not test only hearing aids; it also evaluates mobile phones.
- C (Correct): The ANSI/IEEE C63.19 standard tests both hearing aids and mobile phones to ensure compatibility.
- D (Incorrect): The standard explicitly tests both devices to ensure they work well together.

Thus, C is the correct answer, as the standard evaluates both hearing aids and mobile phones.

Question 48:

Answer: A. 完全不會有迴授音的問題

Explanation:
Middle ear implantable hearing devices (MEIs) offer several advantages over traditional air-conduction hearing aids, but they are not entirely free from feedback issues.

- A (Incorrect): While MEIs are less prone to feedback compared to traditional hearing aids, they are not completely immune to feedback, especially at high gain levels.

- B (Correct): MEIs can provide a broader frequency response, including higher frequencies, which can improve sound quality.
- C (Correct): MEIs do not cause occlusion effects because they do not block the ear canal, unlike traditional hearing aids.
- D (Correct): MEIs can improve speech understanding in both quiet and noisy environments due to their direct stimulation of the middle ear.

Thus, A is the least appropriate statement, as MEIs are not entirely free from feedback issues.

Question 49:

Answer: C. 增加聽覺系統的頻率解析度（frequency resolution）

Explanation:
Modern digital hearing aids are designed to enhance various aspects of hearing, particularly for individuals with hearing loss. One of the key features of digital hearing aids is their ability to improve frequency resolution, which allows users to better distinguish between different frequencies in complex listening environments.

- A (Incorrect): Hearing aids cannot improve unaided air conduction thresholds; they amplify sound to make it audible for individuals with hearing loss.
- B (Incorrect): Digital hearing aids often use compression to manage the dynamic range of sounds, making loud sounds softer and soft sounds louder, rather than reducing the overall dynamic range.
- C (Correct): Digital hearing aids can enhance frequency resolution by using advanced signal processing techniques, such as multi-channel processing, to improve the clarity of speech and other sounds.
- D (Incorrect): While hearing aids can improve the perception of frequency-specific sounds, they do not directly alter the cochlea's inherent frequency selectivity.

Thus, C is the correct answer, as increasing frequency resolution is a common and beneficial feature of modern digital hearing aids.

Question 50:

Answer: A. 頭影效應（head shadow effect）

Explanation:
When evaluating speech perception scores in noise for a single hearing aid user with an FM system in a sound field, it is important to consider the head shadow effect. This effect occurs because the head blocks high-frequency sounds from reaching the ear on the opposite side, which can significantly impact speech perception, especially in noisy environments.

- A (Correct): The head shadow effect can cause a reduction in high-frequency sound reaching the non-aided ear, which can affect the accuracy of speech perception scores.
- B (Incorrect): The far-field effect refers to the reduction in sound intensity as distance increases, but it is not specifically relevant to the head shadow effect in this context.
- C (Incorrect): Standing wave effects are related to sound reflections within enclosed spaces and are not directly relevant to the head shadow effect.
- D (Incorrect): Harmonic effects relate to the presence of harmonic frequencies in sounds and are not directly related to the head shadow effect.

Thus, A is the correct answer, as the head shadow effect is a critical factor to consider when evaluating speech perception in noise for single hearing aid users.

5106

Question 1:

Answer: C. ②①④③⑤⑥

Explanation:
The counseling process for individuals with hearing loss typically follows a structured sequence to ensure effective support and rehabilitation:

1. ②幫助個案敘說自己的故事 (Help the client tell their story): This step allows the client to express their experiences and challenges, providing a foundation for understanding their needs.
2. ①幫助個案釐清問題 (Help the client clarify the problem): After hearing the client's story, the counselor helps them identify and articulate the specific issues they are facing.
3. ④幫助個案負起自己聽力問題的責任 (Help the client take responsibility for their hearing issues): Encouraging the client to take ownership of their hearing loss is crucial for active participation in the rehabilitation process.
4. ③幫助個案建立復健目標 (Help the client establish rehabilitation goals): With a clear understanding of the problems, the client and counselor can set realistic and achievable goals.
5. ⑤擬出計畫 (Develop a plan): A detailed plan is created to address the identified goals and problems.
6. ⑥執行計畫 (Implement the plan): The final step involves putting the plan into action and monitoring progress.

Thus, the correct sequence is C. ②①④③⑤⑥.

Question 2:

Answer: C. 溝通策略的使用

Explanation:
Understanding how an adult client uses their hearing aids helps evaluate the effectiveness of the auditory rehabilitation program. Key aspects typically include:

- A (使用時長): The amount of time the client uses the hearing aids daily is important for assessing adherence and benefit.
- B (使用情境): The environments in which the hearing aids are used (e.g., quiet vs. noisy) help determine their effectiveness in different settings.
- D (使用助聽器時的熟練度): The client's proficiency in using the hearing aids, including handling and adjusting them, is crucial for optimal use.

C (溝通策略的使用): While communication strategies are important in auditory rehabilitation, they are not directly related to the use of hearing aids. This aspect focuses more on behavioral and conversational techniques rather than the technical use of hearing aids.

Thus, C is the least relevant aspect in this context.

Question 3:

Answer: D. 使用助聽器可以解決聽覺處理問題

Explanation:
Age-related hearing loss (presbycusis) involves several complex factors:

- A (音位退化): Phonemic regression, where the ability to understand speech deteriorates faster than the pure-tone thresholds, is common in elderly individuals.
- B (周邊與中樞聽覺處理的問題): Age-related hearing loss often involves both peripheral (cochlear) and central (brain) auditory processing issues.
- C (對高頻的聲音通常較聽不清楚): High-frequency hearing loss is a hallmark of presbycusis, affecting the perception of consonants and speech clarity.
- D (使用助聽器可以解決聽覺處理問題): While hearing aids can amplify sounds and improve audibility, they cannot fully resolve central auditory processing problems, which involve the brain's ability to interpret sounds.

Thus, D is the least appropriate statement, as hearing aids do not address central auditory processing issues.

Question 4:

Answer: C. 相關個人因素（Related personal factors）

Explanation:
In the CORE and CARE model for audiological rehabilitation, the CORE assessment evaluates various aspects of the individual's hearing and communication needs. The Related personal factors category includes demographic and personal characteristics such as intelligence quotient (IQ), age, ethnicity, and gender. These factors help tailor the rehabilitation plan to the individual's specific needs and circumstances.

- A (Communication status): This refers to the individual's current communication abilities and challenges, not personal demographics.
- B (Overall participation variables): This involves the individual's participation in daily activities and social roles, not personal characteristics.

- D (Environmental factors): This includes the physical and social environment that may affect communication, not personal demographics.

Thus, C is the correct answer, as it specifically addresses the personal factors like IQ, age, ethnicity, and gender.

Question 5:

Answer: C. 擔心無法融入群體而迴避社會互動的情境

Explanation:
The World Health Organization's (WHO) International Classification of Functioning, Disability, and Health (ICF) framework considers participation restrictions as limitations an individual may experience in involvement in life situations.

- A (言語聽辨測驗得分低於 60%): This relates to an impairment in auditory function, not directly to participation restrictions.
- B (在安靜的環境無法回應旁人提問的能力): This also relates to an impairment in auditory function or activity limitation, not participation.
- C (擔心無法融入群體而迴避社會互動的情境): This directly relates to participation restrictions, as it describes a limitation in social interaction due to hearing loss.
- D (佩戴助聽器效益不佳): This relates to the effectiveness of a device or intervention, not directly to participation restrictions.

Thus, C is the most relevant to participation restrictions, as it describes a social limitation resulting from hearing loss.

Question 6:

Answer: A. 先給予「夜市」這個提示

Explanation:
Providing contextual cues can significantly improve speech perception, especially for individuals with hearing loss. In this case, the sentence "很多人喜歡去夜市吃臭豆腐" (Many people like to go to the night market to eat stinky tofu) contains specific contextual information.

- A (先給予「夜市」這個提示): Giving the cue "夜市" (night market) helps the listener anticipate the context of the sentence, making it easier to recognize related words like "臭豆腐" (stinky tofu). This contextual information can enhance comprehension and improve listening performance.

- B (提示他：有 12 個字，六個節律線索): While providing information about the number of words and rhythmic cues might help with sentence structure, it does not provide meaningful context to aid in understanding the content.
- C (提示他：你不喜歡做什麼): This cue is unrelated to the sentence and does not provide helpful context for understanding the specific content.
- D (去除協同構音的影響，例如延長「豆」和「腐」之間的停頓時間): While reducing coarticulation effects might improve clarity, it does not provide contextual information that aids in understanding the sentence.

Thus, A is the most effective cue, as it provides relevant context that can enhance comprehension.

Question 7:

Answer: A. /ㄊ/、/ㄎ/

Explanation:
For children with moderate sensorineural hearing loss, the difficulty in distinguishing consonants often depends on the acoustic characteristics of the sounds, such as their frequency and duration.

- A (/ㄊ/、/ㄎ/): The consonants /ㄊ/ (t) and /ㄎ/ (k) are both voiceless stops with similar high-frequency components, making them difficult to distinguish, especially for individuals with hearing loss who may have reduced high-frequency hearing.
- B (/ㄇ/、/ㄏ/): The consonants /ㄇ/ (m) and /ㄏ/ (h) have different acoustic properties, with /ㄇ/ being a nasal sound and /ㄏ/ being a fricative, making them easier to distinguish.
- C (/ㄆ/、/ㄖ/): The consonants /ㄆ/ (p) and /ㄖ/ (r) have different acoustic characteristics, with /ㄆ/ being a stop and /ㄖ/ being a liquid, making them easier to distinguish.
- D (/ㄈ/、/ㄋ/): The consonants /ㄈ/ (f) and /ㄋ/ (n) have different acoustic properties, with /ㄈ/ being a fricative and /ㄋ/ being a nasal, making them easier to distinguish.

Thus, A is the correct answer, as /ㄊ/ and /ㄎ/ are the most challenging pair to distinguish due to their similar high-frequency components.

Question 8:

Answer: C. 成人語音知覺測驗中可加入背景噪音

Explanation:
Speech perception assessment is crucial for understanding how well individuals with hearing loss can understand speech in various conditions.

- A (年紀太小的嬰幼兒無法進行評估): This is incorrect because even very young children can be assessed using age-appropriate speech perception tests, such as conditioned play audiometry or observation-based methods.
- B (評估工具必須符合受試者的聽力損失程度): While it is important to use appropriate tools, this statement is too general. The key is to use tests that match the individual's cognitive and linguistic abilities, not just their hearing loss level.
- C (成人語音知覺測驗中可加入背景噪音): This is correct. Adding background noise to speech perception tests for adults can provide a more realistic assessment of their hearing abilities in everyday listening environments.
- D (評估時不可提供視覺線索): This is incorrect. Visual cues, such as lip-reading, are often included in speech perception assessments to evaluate the individual's ability to integrate auditory and visual information.

Thus, C is the most appropriate answer, as it reflects a realistic approach to assessing speech perception in adults.

Question 9:

Answer: B. 在超語段錯誤中表現不一，而嗓音常不受影響

Explanation:
Assessing and intervening in the speech production of children with profound hearing loss involves understanding various aspects of their speech errors and the impact of interventions like cochlear implants.

- A (在語段錯誤中常見韻母中性化及字尾聲母忽略): This is correct. Children with profound hearing loss often exhibit segmental errors, such as vowel neutralization and omission of final consonants.
- B (在超語段錯誤中表現不一，而嗓音常不受影響): This is incorrect. Suprasegmental errors, such as intonation, stress, and rhythm, are often affected in children with profound hearing loss. Additionally, voice quality (嗓音) can be impacted due to poor auditory feedback.
- C (早期人工電子耳植入者其整體說話清晰度，通常比只使用助聽器者來得好): This is correct. Early cochlear implantation generally leads to better speech clarity compared to using hearing aids alone, due to improved access to auditory information.
- D (人工電子耳植入在語言形式內容和語用方面均可能改善): This is correct. Cochlear implants can improve various aspects of language, including form, content, and pragmatics.

Thus, B is the least appropriate statement, as suprasegmental errors and voice quality are often affected in children with profound hearing loss.

Question 10:

Answer: A. 轉錄分析（transcription analysis）為結構化互動溝通的評估方式之一

Explanation:
Assessing conversational fluency in individuals with hearing loss involves various methods to understand their communication abilities.

- A (轉錄分析為結構化互動溝通的評估方式之一): This is correct. Transcription analysis involves recording and transcribing conversations to analyze aspects like turn-taking, repair strategies, and fluency. It is a structured way to assess interactive communication.
- B (以聽損者輪替的平均說話量為分母，計算平均會話輪替比率（MLT ratio）值，高於 10 表示交談流暢度越高): This is incorrect. The Mean Length of Turn (MLT) ratio is not typically used to measure fluency, and a higher value does not necessarily indicate better fluency.
- C (訪談式的評估方式較難得到定量的資訊): This is incorrect. Interview-based assessments can provide both qualitative and quantitative data, depending on how they are structured.
- D (客戶導向進步量表（COSI）屬於封閉式問卷評估方式): This is incorrect. COSI is a client-centered, open-ended tool that allows individuals to identify and prioritize their specific communication needs and goals.

Thus, A is the most appropriate answer, as transcription analysis is a structured method for evaluating interactive communication.

Question 11:

Answer: C. ①③④⑥

Explanation:
Children with hearing loss often exhibit specific language characteristics due to limited auditory input and delayed language development.

- ① (平均語句長度（MLU）較短): Correct. Children with hearing loss often have shorter mean length of utterance due to delayed language development.
- ② (較少使用被動語句): Incorrect. The use of passive sentences is not specifically associated with hearing loss.
- ③ (多使用類別標籤): Correct. Children with hearing loss may rely more on category labels due to limited vocabulary and syntactic complexity.

- ④ (欠缺理解對話規則的知識): Correct. Difficulty understanding conversational rules is common in children with hearing loss due to reduced auditory input.
- ⑤ (用道具玩角色扮演遊戲的能力差): Incorrect. This is not a specific language characteristic of children with hearing loss.
- ⑥ (表達性和接受性詞彙較少): Correct. Children with hearing loss often have limited expressive and receptive vocabularies.

Thus, C (①③④⑥) is the correct answer, as these are common language characteristics of children with hearing loss.

Question 12:

Answer: C. 說話速度由快速轉為中等速度

Explanation:
Increasing the difficulty of auditory test materials involves modifying factors that challenge the listener's auditory processing abilities.

- A (反應模式由選擇題改為開放式作答): This is appropriate. Open-ended responses are more challenging than multiple-choice, as they require greater auditory processing and recall.
- B (情境由安靜音轉為增加噪音): This is appropriate. Adding background noise increases the difficulty by masking the target speech.
- C (說話速度由快速轉為中等速度): This is inappropriate. Slowing speech from fast to medium speed actually reduces difficulty, as it provides more time for processing.
- D (說話者由男性換為女性): This is appropriate. Changing the speaker's gender can introduce variability in pitch and timbre, increasing the difficulty of speech perception.

Thus, C is the least appropriate option, as reducing speech speed decreases the difficulty of the task.

Question 13:

Answer: C. 人工電子耳植入者訓練的音樂歌曲通常是偏低音的

Explanation:
Improving music perception in cochlear implant (CI) users involves understanding their limitations and tailoring training accordingly.

- A (人工電子耳植入者聆聽音樂感受性較佳的通常是節奏): This is correct. CI users typically perceive rhythm better than pitch or melody due to the temporal resolution of the device.

- B (整首歌的音樂感知訓練（Whole Song Approach）主要為音質及旋律的訓練): This is correct. The Whole Song Approach focuses on improving the perception of melody and timbre, which are challenging for CI users.
- C (人工電子耳植入者訓練的音樂歌曲通常是偏低音的): This is incorrect. CI users often struggle with low-frequency sounds due to the device's limitations in transmitting fine pitch information. Training typically focuses on higher frequencies, where CI users have better pitch perception.
- D (人工電子耳在位置音調（place-pitch）及速率音調（rate-pitch）的限制影響了旋律的感知): This is correct. CI users have difficulty perceiving melody due to limitations in place-pitch and rate-pitch encoding.

Thus, C is the least appropriate statement, as training for CI users usually does not focus on low-pitched music.

Question 14:

Answer: A. 年齡

Explanation:
Lipreading performance can be influenced by various factors, but age is not a primary predictor.

- A (年齡): This is incorrect. While age may affect cognitive processing speed, it is not a direct predictor of lipreading ability.
- B (習語前失聰者): This is correct. Prelingually deaf individuals often develop better lipreading skills due to early reliance on visual cues for communication.
- C (工作記憶): This is correct. Working memory capacity is important for processing and integrating visual and auditory information during lipreading.
- D (失聰期的長短): This is correct. The duration of deafness can impact lipreading ability, as longer periods of deafness may lead to greater reliance on visual cues.

Thus, A is the least relevant factor in predicting lipreading performance.

Question 15:

Answer: D. 用問題確認對方講的主題

Explanation:
Expressive repair strategies are techniques used by individuals with hearing loss to clarify or repair communication breakdowns.

- A (將長句子分成幾個短句重述): This is appropriate. Breaking down long sentences into shorter ones can improve clarity and understanding.
- B (將說話速度變慢一點，並讓咬字清楚一點): This is appropriate. Slowing speech and enunciating clearly can help the listener better understand the message.
- C (利用額外的手勢): This is appropriate. Using gestures can provide additional visual cues to aid comprehension.
- D (用問題確認對方講的主題): This is inappropriate. While asking questions can help clarify misunderstandings, it is not an expressive repair strategy. It is more of a receptive strategy used by the listener.

Thus, D is the least appropriate option, as it does not involve expressive repair strategies.

Question 16:

Answer: D. 強調一邊說話、一邊打出自然手語

Explanation:
Total communication (TC) is an approach that combines various methods of communication to support individuals with hearing loss. It emphasizes the use of both spoken language and sign language simultaneously to ensure effective communication.

- A (又稱為同時溝通法（simultaneous communication）): This is incorrect. While total communication involves simultaneous use of speech and sign, it is not exclusively referred to as simultaneous communication.
- B (以聾人社群的手語作為第一語言，並且也包括主流社會的口語): This is incorrect. Total communication does not prioritize sign language as the first language; it integrates both sign and spoken language equally.
- C (強調先用口語表達，然後再加上自然手語補充訊息): This is incorrect. Total communication does not prioritize spoken language over sign language; both are used simultaneously.
- D (強調一邊說話、一邊打出自然手語): This is correct. Total communication emphasizes the simultaneous use of spoken language and natural sign language to enhance understanding and communication.

Thus, D is the most appropriate answer, as it accurately describes the total communication approach.

Question 17:

Answer: D. 就相同的語句而言，兒童重述的清晰度高於仿說

Explanation:
Speech clarity can be influenced by various factors, including context, familiarity, and multimodal cues.

- A (兒童朗讀一段文章的清晰度，會高於朗讀一段沒關聯的句子): This is appropriate. Reading a coherent passage provides context, which can enhance clarity compared to unrelated sentences.
- B (曾經聽過聽損孩童說話的評分者，比較能聽懂孩童所說的話): This is appropriate. Familiarity with the speaker's speech patterns can improve the listener's ability to understand them.
- C (相較於只聽到說話聲音，若評分者可以聽到並看到說話的兒童，會有助於他們辨識): This is appropriate. Visual cues, such as lip-reading, can significantly enhance speech perception.
- D (就相同的語句而言，兒童重述的清晰度高於仿說): This is inappropriate. Typically, imitating speech (仿說) tends to be clearer than spontaneous repetition (重述) because imitation allows for more precise articulation.

Thus, D is the least appropriate statement, as it inaccurately suggests that repetition is clearer than imitation.

Question 18:

Answer: A. 強調多感官教學法，促進聽與說

Explanation:
The auditory-verbal approach focuses on developing listening and spoken language skills through the use of residual hearing, without relying on visual cues like sign language or lip-reading.

- A (強調多感官教學法，促進聽與說): This is incorrect. The auditory-verbal approach emphasizes auditory input and does not rely on multi-sensory teaching methods. It focuses solely on listening and spoken language.
- B (指導家長或主要照顧者成為重要的教導者): This is correct. Parents and caregivers are integral to the auditory-verbal approach, as they are trained to support the child's listening and spoken language development in everyday situations.
- C (其目標設計是參照聽常者的正常發展模式與階段來進行的): This is correct. The auditory-verbal approach aims to help children with hearing loss achieve language development milestones similar to those of their hearing peers.

- D (採用特定的說話技巧，例如使用完整的句子、聽覺三明治等): This is correct. Specific techniques, such as using complete sentences and the "auditory sandwich" (presenting information auditorily, then visually if needed, and then auditorily again), are used to reinforce listening and spoken language skills.

Thus, A is the incorrect statement, as the auditory-verbal approach does not emphasize multi-sensory teaching methods.

Question 19:

Answer: A. 僅①②③

Explanation:
Formal auditory training principles are designed to enhance listening skills through structured activities and exercises.

- ① (可由不同說話者說出訓練內容，有助於其類化): This is correct. Using different speakers helps individuals generalize their listening skills to various voices and speaking styles.
- ② (非語音的訓練材料只用於習語前聽損幼童): This is incorrect. Non-speech training materials can be used for individuals of all ages, not just prelingually deaf children.
- ③ (短時間內要呈現大量的訓練項目材料): This is correct. Presenting a variety of training materials in a short period can help maintain engagement and improve listening skills.
- ④ (聽覺訓練的活動不可以同時包括分析式和綜合式的刺激材料): This is incorrect. Auditory training activities can and often do include both analytic (focusing on specific sounds or words) and synthetic (focusing on whole sentences or conversations) stimuli.
- ⑤ (每天至少應練習 50 分鐘，且最好在每天同一時間進行，以維持正常作息): This is incorrect. While regular practice is important, the duration and timing should be flexible and tailored to the individual's needs and schedule.

Thus, A (僅①②③) is the correct answer, as these principles align with effective auditory training practices.

Question 20:

Answer: A. 察覺

Explanation:
The activity described involves teaching a child with hearing loss to respond to a sound by hiding. This is a basic level of auditory skill, which is detection (察覺). Detection refers to the ability to perceive the presence or absence of sound.

- A (察覺): This is correct. The child is learning to detect the presence of sound.
- B (分辨): This refers to the ability to distinguish between different sounds, which is not the focus of this activity.
- C (辨識): This involves recognizing and labeling specific sounds, which is a higher-level skill than detection.
- D (理解): This involves comprehending the meaning of sounds or speech, which is more advanced than detection.

Thus, A is the correct answer, as the activity focuses on detecting sound.

Question 21:

Answer: C. 詞性

Explanation:
The Neighborhood Activation Model (NAM) explains how words are activated in the mental lexicon based on their phonological similarity and frequency. When applied to audiovisual integration, the following factors influence lexical activation:

- A (視覺上的相似度): Visual similarity (e.g., lip-reading cues) can influence word activation.
- B (聽覺上的相似度): Auditory similarity (e.g., phonological overlap) is a key factor in NAM.
- C (詞性): Grammatical category (e.g., noun, verb) is not a factor in the NAM. It does not influence lexical activation in this model.
- D (詞頻): Word frequency is a critical factor in NAM, as higher-frequency words are more easily activated.

Thus, C is the correct answer, as word class (詞性) is not a factor in the NAM.

Question 22:

Answer: B. ①③②

Explanation:
Analytic speechreading training progresses from easier to more difficult tasks. The difficulty of distinguishing initial consonants depends on their visual and auditory distinctiveness:

- ① (辨識聲母「呆」與「塞」): This is relatively easy because the consonants /d/ and /s/ are visually and auditorily distinct.

- ③ (辨識聲母「掰」與「拍」): This is moderately difficult because /b/ and /p/ are visually similar (both are bilabial) but auditorily distinct.
- ② (辨識聲母「掰」與「該」): This is the most difficult because /b/ and /g/ are visually and auditorily less distinct.

Thus, the correct order from easiest to hardest is B (①③②).

Question 23:

Answer: C.「住在城市裡的青年人」對比「住在城市裡的老年人」

Explanation:
Research has shown that speechreading ability can vary significantly based on age, even when hearing levels are controlled. Older adults often experience declines in cognitive processing speed and visual perception, which can affect their speechreading performance compared to younger adults.

- A (「最高學歷為高中的中年人」對比「最高學歷為大學的中年人」): Educational level may influence cognitive abilities, but it is not the most significant factor in speechreading differences.
- B (「成年男性」對比「成年女性」): Gender differences in speechreading ability are not well-supported by research.
- C (「住在城市裡的青年人」對比「住在城市裡的老年人」): This is correct. Age-related declines in cognitive and visual processing can lead to significant differences in speechreading ability.
- D (「一般智商的大學生」對比「優秀智商的大學生」): While IQ may influence cognitive abilities, it is not the primary factor in speechreading differences.

Thus, C is the correct answer, as age is a significant factor in speechreading ability.

Question 24:
The audiologist asks the hearing-impaired individual to circle words that express their feelings about using hearing aids within one minute. Then, the audiologist discusses these words with the individual to reduce the likelihood of negative emotions hindering constructive actions. Which individual adjustment counseling model is the audiologist most likely using to help the individual?
A. Affective Model
B. Informational Counseling Model
C. Cognitive Model
D. Psychosocial Model

Answer: A. Affective Model

Explanation:
The affective model focuses on addressing the emotional and psychological aspects of hearing loss and the use of hearing aids. By having the individual identify and discuss words that express their feelings, the audiologist is directly engaging with the individual's emotions. This approach aims to reduce negative emotions and facilitate a more positive and constructive attitude toward hearing aid use. The affective model is particularly effective in helping individuals process their feelings and develop a healthier emotional response to their hearing loss and rehabilitation.

Question 25:
Which of the following responses from a hearing-impaired individual to a speaker is not considered a specific repair strategy?
A. Repeating part of the message
B. "Please rephrase that."
C. "What did you say?"
D. "Are you saying you will come to see me later?"

Answer: C. "What did you say?"

Explanation:
Specific repair strategies are techniques used by individuals with hearing loss to clarify or confirm misunderstood parts of a conversation. These strategies are targeted and specific, aiming to resolve communication breakdowns effectively.

- A. Repeating part of the message: This is a specific repair strategy because it involves repeating a portion of the message to confirm understanding.
- B. "Please rephrase that.": This is a specific repair strategy because it asks the speaker to rephrase their statement for better clarity.
- C. "What did you say?": This is not a specific repair strategy. It is a general request for repetition and does not target a specific part of the message or provide context for clarification.
- D. "Are you saying you will come to see me later?": This is a specific repair strategy because it confirms a specific part of the message for accuracy.

Thus, option C is the correct answer as it does not qualify as a specific repair strategy.

Question 26:
Which of the following methods cannot reduce noise levels and reverberation in a classroom?
A. Laying carpet on the floor
B. Installing rubber pads on chair legs
C. Raising the ceiling
D. Placing bookshelves or partitions in the classroom

Answer: C. Raising the ceiling

Explanation:
Reducing noise and reverberation in a classroom involves using materials and strategies that absorb sound rather than reflect it.

- A. Laying carpet on the floor: Carpets absorb sound, reducing noise levels and reverberation.
- B. Installing rubber pads on chair legs: This reduces impact noise (e.g., scraping sounds) and contributes to a quieter environment.
- C. Raising the ceiling: This does not reduce noise or reverberation. In fact, it can increase reverberation because sound waves have more space to travel and reflect, making the room acoustically worse.
- D. Placing bookshelves or partitions in the classroom: These act as sound barriers and absorbers, reducing noise and reverberation.

Thus, raising the ceiling (option C) is the correct answer because it does not help reduce noise or reverberation and may even worsen the acoustic environment.

Question 27:
Xinyi's school is located in a suburban area, where traffic is only heavy during drop-off and pick-up times. One day at noon, the entire school gathered on the playground, and the principal asked everyone to listen quietly to a teacher speaking on the stage. Xinyi's class was positioned at the far right rear of the playground, more than 40 meters from the speaker. Xinyi wears hearing aids and can communicate easily with classmates nearby and has no issues listening in the classroom. However, she cannot hear the teacher on the stage clearly. What is the most significant factor contributing to this result?
A. Reverberation makes the speech sound blurry
B. Noise is too loud, overpowering the speaker's voice
C. Distance is too far, reducing the speaker's volume
D. Insufficient gain from the hearing aids, making it hard for Xinyi to hear

Answer: C. Distance is too far, reducing the speaker's volume

Explanation:
In this scenario, the key factors to consider are:

1. Distance: Xinyi is more than 40 meters away from the speaker. Sound intensity decreases significantly with distance due to the inverse square law, making it harder to hear the speaker clearly.
2. Environment: While the school is in a quiet suburban area, the open outdoor setting lacks walls or ceilings to reflect or amplify sound, further reducing the speaker's volume at a distance.
3. Hearing Aids: Xinyi's hearing aids work well in close-range and classroom settings, indicating they are functioning properly. However, hearing aids have limitations in amplifying distant sounds, especially in open spaces.

- A. Reverberation: This is unlikely to be a significant factor outdoors, as reverberation is more common in enclosed spaces with hard surfaces.

- B. Noise: The scenario states that the area is quiet, so noise is not a major issue here.
- C. Distance: This is the most significant factor. The teacher's voice is naturally quieter at 40 meters, and hearing aids cannot fully compensate for this distance in an open outdoor setting.
- D. Insufficient gain: While hearing aids have limitations, the problem here is primarily due to the distance, not the hearing aids themselves.

Thus, the most obvious factor is the distance (option C), which reduces the speaker's volume and makes it difficult for Xinyi to hear clearly.

Question 28:
Which of the following statements about conversation-based therapy is most appropriate?

1. Teach the listener to check the coherence of the message to ensure understanding.
2. Teach the speaker to speak in a way that is easy to understand.
3. Use linguistic rules and existing knowledge to interpret the meaning of the message.
4. Improve the acoustic and optical conditions of the environment to enhance signals and reduce interference.
   A. Only ②③④
   B. Only ①②③
   C. Only ①②④
   D. ①②③④

Answer: D. ①②③④

Explanation:
Conversation-based therapy focuses on improving communication by addressing both the speaker's and listener's roles, as well as the environment. All four statements are relevant:

1. Teach the listener to check the coherence of the message: This ensures the listener actively verifies their understanding, which is a key part of effective communication.
2. Teach the speaker to speak clearly: This helps the speaker convey their message in a way that is easier for the listener to understand.
3. Use linguistic rules and existing knowledge: This helps the listener interpret the message accurately, especially in challenging listening situations.
4. Improve environmental conditions: Enhancing acoustics (e.g., reducing noise) and optimizing visual cues (e.g., lighting) can significantly improve communication.

Thus, all four statements are appropriate, making D. ①②③④ the correct answer.

Question 29:
Which of the following is not part of the auditory management services provided by an audiologist for hearing-impaired students in schools?
A. Assessing and adjusting the acoustic environment of the classroom for hearing-impaired students
B. Developing individualized transition plans for hearing-impaired students
C. Regularly performing electroacoustic analysis of hearing aids and FM systems for hearing-impaired students
D. Training classroom teachers to monitor and check students' hearing assistive devices

Answer: B. Developing individualized transition plans for hearing-impaired students

Explanation:
Audiologists provide a range of services to support hearing-impaired students in schools, but their primary focus is on auditory and technical aspects rather than educational planning.

- A. Acoustic environment assessment and adjustment: This is within the scope of an audiologist's role, as they ensure the classroom environment is optimized for hearing.
- B. Developing individualized transition plans: This is typically the responsibility of special education professionals or school counselors, not audiologists. Transition plans focus on educational and life goals, which fall outside the audiologist's expertise.
- C. Electroacoustic analysis of hearing aids and FM systems: This is a core responsibility of audiologists, ensuring that hearing devices are functioning properly.
- D. Training teachers to monitor hearing assistive devices: Audiologists often provide training to teachers to ensure proper use and maintenance of hearing devices.

Thus, B. Developing individualized transition plans is not part of an audiologist's auditory management services, making it the correct answer.

Question 30:
The Multiple Auditory Processing Assessment (MAPA) test battery follows Musiek and Chermak's recommendations and primarily evaluates the three key areas of Central Auditory Processing Disorder (CAPD) proposed by ASHA. Which of the following tests is not included in MAPA?
A. Monaural Selective Auditory Attention Test (MSAAT)
B. Listening in Spatialized Noise-Sentences Test (LISN-S)
C. Dichotic Digits Test
D. Pitch Pattern Test

Answer: B. Listening in Spatialized Noise-Sentences Test (LISN-S)

Explanation:
The MAPA test battery is designed to assess the three key areas of CAPD as outlined by ASHA: auditory discrimination, temporal processing, and binaural integration/separation.

- A. Monaural Selective Auditory Attention Test (MSAAT): This test evaluates auditory attention and discrimination in the presence of competing noise, which aligns with CAPD assessment goals.

- B. Listening in Spatialized Noise-Sentences Test (LISN-S): This test is not part of the MAPA battery. It assesses spatial hearing and speech perception in noise but is not specifically included in MAPA.
- C. Dichotic Digits Test: This is a core test in MAPA, evaluating binaural integration and separation, which is one of the key CAPD areas.
- D. Pitch Pattern Test: This test evaluates temporal processing and pattern recognition, which is another key CAPD area and is included in MAPA.

Thus, B. Listening in Spatialized Noise-Sentences Test (LISN-S) is the correct answer, as it is not part of the MAPA test battery.

Question 31:
Yuchen is a patient with Central Auditory Processing Disorder (CAPD). Which of the following descriptions about him are possible?

1. Difficulty detecting sounds
2. Difficulty localizing sounds
3. Diagnosed with CAPD in the second grade
4. Difficulty following instructions
   A. Only ①③
   B. ②③④
   C. Only ②④
   D. ①③④

Answer: B. ②③④

Explanation:
Central Auditory Processing Disorder (CAPD) affects how the brain processes auditory information, even though hearing sensitivity may be normal. Common characteristics include:

1. Difficulty detecting sounds: This is not typical of CAPD. Patients with CAPD usually have normal hearing thresholds but struggle with processing auditory information.
2. Difficulty localizing sounds: This is a common issue for CAPD patients, as sound localization relies on binaural processing, which can be impaired in CAPD.
3. Diagnosed with CAPD in the second grade: CAPD is often identified in school-aged children when auditory processing difficulties become apparent in academic or social settings.
4. Difficulty following instructions: This is a hallmark of CAPD, as patients may struggle to process and retain complex or multi-step verbal instructions.

Thus, the correct answer is B. ②③④, as these descriptions align with the characteristics of CAPD.

Question 32:
The Masking Level Difference (MLD) test is most sensitive to lesions in which part of the auditory system?
A. Low brainstem
B. High brainstem
C. Auditory cortex
D. Corpus callosum

Answer: A. Low brainstem

Explanation:
The Masking Level Difference (MLD) test measures binaural hearing abilities, specifically the brain's ability to detect differences in phase or timing between the ears. This test is highly sensitive to lesions in the low brainstem, particularly the superior olivary complex, which is responsible for binaural processing and sound localization.

- A. Low brainstem: Correct. The MLD test is most sensitive to lesions in this area.
- B. High brainstem: Lesions in the high brainstem (e.g., inferior colliculus) may affect auditory processing but are not the primary focus of the MLD test.
- C. Auditory cortex: The MLD test is not primarily used to assess cortical lesions.
- D. Corpus callosum: This structure is involved in interhemispheric communication but is not the primary focus of the MLD test.

Thus, the correct answer is A. Low brainstem.

Question 33:
Regarding the management and treatment of children with Central Auditory Processing Disorder (CAPD) in educational settings, which of the following statements is most appropriate?
A. Choosing the seat closest to the speaker is the most useful approach.
B. Improving the signal-to-noise ratio (SNR) is the most effective approach.
C. For students with temporal processing issues, an RM system can solve the problem.
D. All CAPD patients benefit from auditory training.

Answer: B. Improving the signal-to-noise ratio (SNR) is the most effective approach.

Explanation:
Managing CAPD in educational settings requires addressing the specific auditory processing challenges faced by the student.

- A. Choosing the seat closest to the speaker: While this can help, it is not the most comprehensive or effective solution for all CAPD-related issues.
- B. Improving the signal-to-noise ratio (SNR): This is the most effective approach. CAPD patients struggle to hear in noisy environments, so reducing background noise and enhancing the speaker's

voice (e.g., using FM systems) can significantly improve their ability to process auditory information.

- C. For students with temporal processing issues, an RM system can solve the problem: RM (Remote Microphone) systems can help improve SNR but do not directly address temporal processing deficits. Temporal processing issues require targeted auditory training or therapy.
- D. All CAPD patients benefit from auditory training: While auditory training can be beneficial, its effectiveness varies depending on the individual's specific deficits and the type of training provided. It is not a universal solution.

Thus, the most appropriate statement is B. Improving the signal-to-noise ratio (SNR) is the most effective approach.

Question 34:
An elderly individual with mild hearing loss reports that even when the speaker's volume is loud enough, speech still sounds chaotic and unclear. Which of the following is the least likely reason for this phenomenon?
A. It is due to sensorineural hearing loss.
B. The perceived noise is actually tinnitus.
C. The dynamic range of hearing has narrowed.
D. This phenomenon is phonemic regression.

Answer: B. The perceived noise is actually tinnitus.

Explanation:
The described issue—difficulty understanding speech despite adequate volume—is commonly associated with sensorineural hearing loss and related auditory processing changes. Let’s evaluate each option:

- A. It is due to sensorineural hearing loss: This is a likely reason. Sensorineural hearing loss often involves reduced speech discrimination, especially in noisy environments, due to damage to the inner ear or auditory nerve.
- B. The perceived noise is actually tinnitus: This is the least likely reason. Tinnitus is the perception of sound (e.g., ringing or buzzing) in the absence of an external source. While tinnitus can be distracting, it does not typically cause speech to sound "chaotic" or "unclear."
- C. The dynamic range of hearing has narrowed: This is a likely reason. Narrowed dynamic range (reduced difference between the threshold of hearing and the level of discomfort) is common in sensorineural hearing loss and can make speech sound distorted or unclear, even at comfortable volumes.
- D. This phenomenon is phonemic regression: This is a possible reason. Phonemic regression refers to age-related difficulty in processing speech sounds, which can occur even with mild hearing loss.

Thus, the least likely reason is B. The perceived noise is actually tinnitus.

Question 35:
Regarding the efficacy of tinnitus treatments, which of the following statements is incorrect?
A. The efficacy of tinnitus treatments should be determined based on well-designed clinical studies.
B. Determining the efficacy of tinnitus treatments only requires validation through well-designed clinical studies, and the results do not need to be replicable.
C. There is no systematic evidence that herbal supplements, such as ginkgo, are effective for tinnitus.
D. There is no systematic evidence that dietary supplements, such as vitamin B12, are effective for tinnitus.

Answer: B. Determining the efficacy of tinnitus treatments only requires validation through well-designed clinical studies, and the results do not need to be replicable.

Explanation:
The efficacy of any medical treatment, including tinnitus treatments, must be based on rigorous scientific evidence. Let's evaluate each option:

- A. The efficacy of tinnitus treatments should be determined based on well-designed clinical studies: This is correct. Well-designed clinical studies (e.g., randomized controlled trials) are the gold standard for evaluating treatment efficacy.
- B. Determining the efficacy of tinnitus treatments only requires validation through well-designed clinical studies, and the results do not need to be replicable: This is incorrect. Scientific validity requires not only well-designed studies but also replicable results. Replication ensures that findings are consistent and not due to chance or bias.
- C. There is no systematic evidence that herbal supplements, such as ginkgo, are effective for tinnitus: This is correct. While some individuals report subjective benefits, systematic reviews and meta-analyses have not found strong evidence supporting the use of ginkgo or other herbal supplements for tinnitus.
- D. There is no systematic evidence that dietary supplements, such as vitamin B12, are effective for tinnitus: This is correct. Similar to herbal supplements, there is no robust evidence that dietary supplements like vitamin B12 are effective for tinnitus.

Thus, the incorrect statement is B. Determining the efficacy of tinnitus treatments only requires validation through well-designed clinical studies, and the results do not need to be replicable.

Question 36:
Which of the following statements about the Dynamic Visual Acuity (DVA) test is incorrect?
A. It uses the vestibulo-ocular reflex (VOR) mechanism to stabilize foveal vision for vestibular function assessment.
B. The patient actively or passively shakes their head at 2 Hz (120–180 deg/sec) while undergoing a visual acuity test.
C. A positive result is indicated if the best visual acuity shifts by more than three lines on the standard visual acuity chart compared to the static condition.

D. Unilateral vestibular dysfunction causes more severe dynamic visual acuity decline due to nystagmus compared to bilateral dysfunction.

Answer: D. Unilateral vestibular dysfunction causes more severe dynamic visual acuity decline due to nystagmus compared to bilateral dysfunction.

Explanation:
The Dynamic Visual Acuity (DVA) test evaluates the vestibulo-ocular reflex (VOR) by assessing the ability to maintain visual acuity during head movement. Let's evaluate each option:

- A. It uses the VOR mechanism to stabilize foveal vision: This is correct. The VOR helps stabilize vision during head movement by coordinating eye movements to compensate for head motion.
- B. The patient shakes their head at 2 Hz (120–180 deg/sec): This is correct. The test involves head movement at a specific frequency to challenge the VOR.
- C. A positive result is indicated by a shift of more than three lines on the visual acuity chart: This is correct. A significant decline in visual acuity during head movement (compared to static conditions) suggests VOR dysfunction.
- D. Unilateral vestibular dysfunction causes more severe dynamic visual acuity decline due to nystagmus compared to bilateral dysfunction: This is incorrect. In bilateral vestibular dysfunction, the VOR is severely impaired in both ears, leading to greater difficulty stabilizing vision during head movement. Unilateral dysfunction typically results in asymmetric but less severe impairment.

Thus, the incorrect statement is D. Unilateral vestibular dysfunction causes more severe dynamic visual acuity decline due to nystagmus compared to bilateral dysfunction.

Question 37:
Which of the following statements about cervical Vestibular Evoked Myogenic Potentials (cVEMP) is incorrect?
A. It refers to changes in myogenic potentials evoked by sound, tapping, or electrical stimulation of the saccule.
B. The output cranial nerve is the 11th accessory nerve.
C. The most commonly recorded myogenic potential change is in the contralateral sternocleidomastoid (SCM) muscle.
D. It is an inhibitory postsynaptic potential.

Answer: C. The most commonly recorded myogenic potential change is in the contralateral sternocleidomastoid (SCM) muscle.

Explanation:
cVEMP is a test used to assess the function of the saccule and inferior vestibular nerve. Let's evaluate each option:

- A. It refers to changes in myogenic potentials evoked by sound, tapping, or electrical stimulation of the saccule: This is correct. cVEMP measures muscle responses (in the SCM) evoked by stimulating the saccule.
- B. The output cranial nerve is the 11th accessory nerve: This is correct. The accessory nerve (cranial nerve XI) innervates the sternocleidomastoid muscle, where cVEMP responses are recorded.
- C. The most commonly recorded myogenic potential change is in the contralateral sternocleidomastoid (SCM) muscle: This is incorrect. cVEMP responses are recorded from the ipsilateral SCM muscle, not the contralateral side.
- D. It is an inhibitory postsynaptic potential: This is correct. cVEMP reflects an inhibitory response in the SCM muscle due to activation of the vestibulo-collic reflex.

Thus, the incorrect statement is C. The most commonly recorded myogenic potential change is in the contralateral sternocleidomastoid (SCM) muscle.

*Image could not be identified by DeepSeek, answered question without the image

Question 38:
When the center of gravity exceeds the limit of stability, which reflexive postural control strategy does the human body employ?
A. Ankle strategy
B. Hip strategy
C. Stepping strategy
D. Suspension strategy

Answer: C. Stepping strategy

Explanation:
The human body uses different postural control strategies to maintain balance, depending on the magnitude and direction of the perturbation (disturbance to balance). These strategies are hierarchical and progress from the least to the most disruptive:

1. Ankle strategy: Used for small perturbations. The body shifts weight by moving around the ankle joint to maintain balance.
2. Hip strategy: Used for larger perturbations. The body generates larger movements at the hip to counteract the disturbance.
3. Stepping strategy: Used when the center of gravity exceeds the limit of stability. The body takes a step to widen the base of support and regain balance.
4. Suspension strategy: Not a recognized postural control strategy.

When the center of gravity moves beyond the limit of stability, the ankle and hip strategies are insufficient to restore balance. At this point, the stepping strategy is employed to prevent a fall by re-establishing a new base of support.

Thus, the correct answer is C. Stepping strategy.

Question 39:
Which of the following statements about the caloric test (ice water caloric test) is incorrect?
A. It is used to test the residual vestibular function of the patient.
B. It should not be performed if the patient has a perforated eardrum.
C. The recommended volume of ice water to be injected is 2 c.c.
D. It is suitable for cases where the total response from binaural bithermal caloric testing is less than 8 deg/s.

Answer: C. The recommended volume of ice water to be injected is 2 c.c.

Explanation:
The caloric test evaluates vestibular function by inducing temperature-dependent convection currents in the horizontal semicircular canal. Let's evaluate each option:

- A. It is used to test residual vestibular function: This is correct. The ice water caloric test is a strong stimulus used to assess remaining vestibular function, especially when no response is observed in standard bithermal caloric testing.
- B. It should not be performed if the patient has a perforated eardrum: This is correct. Introducing water into the ear canal when the eardrum is perforated can lead to infection or other complications.
- C. The recommended volume of ice water to be injected is 2 c.c.: This is incorrect. The standard volume for ice water caloric testing is 0.2–0.4 c.c., not 2 c.c. Using 2 c.c. would be excessive and could cause discomfort or adverse effects.
- D. It is suitable for cases where the total response from binaural bithermal caloric testing is less than 8 deg/s: This is correct. The ice water caloric test is used when there is minimal or no response to standard caloric testing, indicating severe vestibular hypofunction.

Thus, the incorrect statement is C. The recommended volume of ice water to be injected is 2 c.c.

Question 40:
Regarding the head-shaking nystagmus test (HSN) and the video head impulse test (vHIT), which of the following statements is correct?
A. Both are highly specific tests for detecting unilateral vestibular dysfunction.
B. Both are based on the vestibulo-ocular reflex (VOR).
C. Both observe horizontal nystagmus.
D. Both are related to the central velocity storage mechanism.

Answer: B. Both are based on the vestibulo-ocular reflex (VOR).

Explanation:
The head-shaking nystagmus test (HSN) and the video head impulse test (vHIT) are both used to assess vestibular function, but they differ in their mechanisms and applications. Let's evaluate each option:

- A. Both are highly specific tests for detecting unilateral vestibular dysfunction: This is partially correct. While both tests can detect unilateral vestibular dysfunction, the vHIT is more specific and provides direct information about semicircular canal function. The HSN test is less specific and can also be influenced by central vestibular pathways.
- B. Both are based on the vestibulo-ocular reflex (VOR): This is correct. Both tests rely on the VOR, which stabilizes gaze during head movements by coordinating eye movements in the opposite direction of head motion.
- C. Both observe horizontal nystagmus: This is incorrect. The vHIT primarily assesses the VOR for all three semicircular canals (horizontal, anterior, and posterior), while the HSN test typically induces horizontal nystagmus in cases of unilateral vestibular dysfunction.
- D. Both are related to the central velocity storage mechanism: This is incorrect. The central velocity storage mechanism is primarily associated with the HSN test, which can reveal asymmetries in vestibular tone. The vHIT, however, directly measures the VOR gain and does not rely on velocity storage.

Thus, the correct statement is B. Both are based on the vestibulo-ocular reflex (VOR).

Question 41:
Which of the following statements about the structure of the semicircular canals is correct?
A. One end of the semicircular canal is enlarged into the ampulla, which contains the crista and cupula. The cupula contains otoconia.
B. The otoconia have a lower density than the cupula and endolymph. When endolymph flows, the otoconia cause the cupula to deflect due to gravity.
C. Hair cells are located within the crista, and their cilia extend into the cupula. Deflection of the cupula causes excitation or inhibition of the hair cells.
D. When the stereocilia of the hair cells bend toward the kinocilium, hyperpolarization occurs, leading to excitation.

Answer: C. Hair cells are located within the crista, and their cilia extend into the cupula. Deflection of the cupula causes excitation or inhibition of the hair cells.

Explanation:
The semicircular canals are part of the vestibular system and detect rotational head movements. Let's evaluate each option:

- A. One end of the semicircular canal is enlarged into the ampulla, which contains the crista and cupula. The cupula contains otoconia: This is incorrect. The cupula does not contain otoconia. Otoconia are found in the otolith organs (utricle and saccule), not in the semicircular canals.
- B. The otoconia have a lower density than the cupula and endolymph. When endolymph flows, the otoconia cause the cupula to deflect due to gravity: This is incorrect. Otoconia are not

involved in semicircular canal function. The cupula is deflected by endolymph movement due to angular acceleration, not gravity.

- C. Hair cells are located within the crista, and their cilia extend into the cupula. Deflection of the cupula causes excitation or inhibition of the hair cells: This is correct. The crista ampullaris contains hair cells whose cilia are embedded in the cupula. When the cupula is deflected by endolymph movement, the hair cells are either excited or inhibited, depending on the direction of deflection.
- D. When the stereocilia of the hair cells bend toward the kinocilium, hyperpolarization occurs, leading to excitation: This is incorrect. Bending of stereocilia toward the kinocilium causes depolarization (excitation), not hyperpolarization. Hyperpolarization occurs when stereocilia bend away from the kinocilium.

Thus, the correct statement is C. Hair cells are located within the crista, and their cilia extend into the cupula. Deflection of the cupula causes excitation or inhibition of the hair cells.

Question 42:
Which cranial nerves control the medial rectus and lateral rectus muscles of the eye?
A. 2nd and 3rd cranial nerves
B. 4th and 5th cranial nerves
C. 3rd and 6th cranial nerves
D. 4th and 6th cranial nerves

Answer: C. 3rd and 6th cranial nerves

Explanation:
The extraocular muscles of the eye are controlled by three cranial nerves: the oculomotor nerve (3rd cranial nerve), the trochlear nerve (4th cranial nerve), and the abducens nerve (6th cranial nerve).

- Medial rectus muscle: This muscle is responsible for adducting the eye (moving it toward the nose). It is controlled by the oculomotor nerve (3rd cranial nerve).
- Lateral rectus muscle: This muscle is responsible for abducting the eye (moving it away from the nose). It is controlled by the abducens nerve (6th cranial nerve).

Thus, the correct answer is C. 3rd and 6th cranial nerves, as these are the nerves that control the medial rectus and lateral rectus muscles, respectively.

Additional Notes:

- The 2nd cranial nerve (optic nerve) is responsible for vision, not eye movement.
- The 4th cranial nerve (trochlear nerve) controls the superior oblique muscle, which is not relevant to this question.
- The 5th cranial nerve (trigeminal nerve) is primarily responsible for facial sensation and mastication, not eye movement.

Question 43:
Which of the following statements about head-shaking nystagmus (HSN) is incorrect?
A. Normal individuals rarely exhibit this type of nystagmus.
B. The presence of horizontal nystagmus must indicate peripheral vestibular dysfunction.
C. Individuals with cerebellar dysfunction can also exhibit nystagmus.
D. A second-phase nystagmus may occur (nystagmus appears immediately after head shaking, followed by a brief pause, and then nystagmus in the opposite direction).

Answer: B. The presence of horizontal nystagmus must indicate peripheral vestibular dysfunction.

Explanation:
Head-shaking nystagmus (HSN) is a test used to assess vestibular asymmetry. Let's evaluate each option:

- A. Normal individuals rarely exhibit this type of nystagmus: This is correct. HSN is typically absent in individuals with normal vestibular function.
- B. The presence of horizontal nystagmus must indicate peripheral vestibular dysfunction: This is incorrect. While horizontal nystagmus is often associated with peripheral vestibular dysfunction, it can also occur in central vestibular disorders (e.g., cerebellar or brainstem lesions). Therefore, it is not exclusive to peripheral dysfunction.
- C. Individuals with cerebellar dysfunction can also exhibit nystagmus: This is correct. Cerebellar dysfunction can lead to abnormal nystagmus due to impaired central vestibular processing.
- D. A second-phase nystagmus may occur: This is correct. HSN can exhibit a biphasic pattern, where the initial nystagmus is followed by a brief pause and then nystagmus in the opposite direction.

Thus, the incorrect statement is B. The presence of horizontal nystagmus must indicate peripheral vestibular dysfunction.

Question 44:
During the caloric test, which parameter is used to calculate unilateral weakness (UW)?
A. Duration of nystagmus
B. Amplitude of nystagmus
C. Velocity of nystagmus
D. Latency of nystagmus

Answer: C. Velocity of nystagmus

Explanation:
The caloric test evaluates vestibular function by measuring the nystagmus response to temperature-induced stimulation of the horizontal semicircular canal. Unilateral weakness (UW) is calculated using the peak slow-phase velocity (SPV) of nystagmus, which reflects the strength of the vestibular response.

The formula for unilateral weakness (UW) is:

UW=(RW+RC)−(LW+LC)RW+RC+LW+LC×100UW=RW+RC+LW+LC(RW+RC)−(LW+LC) ×100

Where:

- RW = Right warm stimulation SPV
- RC = Right cold stimulation SPV
- LW = Left warm stimulation SPV
- LC = Left cold stimulation SPV

Thus, the correct answer is C. Velocity of nystagmus, as the slow-phase velocity is the key parameter used in the calculation.

Question 45:
Which of the following vestibular function tests can assess unilateral vestibular function independently, without being influenced by the contralateral vestibular system?
A. Head-shaking nystagmus test (HSN)
B. Caloric test
C. Posturography
D. Rotational chair test

Answer: B. Caloric test

Explanation:
The ability to test unilateral vestibular function independently is a key feature of certain vestibular tests. Let's evaluate each option:

- A. Head-shaking nystagmus test (HSN): This test assesses vestibular asymmetry but does not isolate unilateral function. It relies on the interaction between both vestibular systems.
- B. Caloric test: This is the correct answer. The caloric test stimulates each ear independently using warm or cold water/air, allowing for the assessment of unilateral vestibular function without influence from the contralateral side.
- C. Posturography: This test evaluates overall balance and postural control but does not isolate unilateral vestibular function.
- D. Rotational chair test: This test assesses bilateral vestibular function by measuring the vestibulo-ocular reflex (VOR) during rotational movements. It does not isolate unilateral function.

Thus, the correct answer is B. Caloric test, as it is the only test listed that can independently assess unilateral vestibular function.

Question 46:
Which of the following statements about the vestibular balance system and postural control is incorrect?

A. In older adults, the amplitude of cervical vestibular evoked myogenic potentials (cVEMP) is smaller compared to younger adults, but there is no reduction in amplitude for ocular vestibular evoked myogenic potentials (oVEMP).
B. Older adults rely more on the visual system to maintain postural balance compared to younger adults.
C. Lower limb strength is important for maintaining and controlling posture and is also an indicator of balance ability.
D. Somatosensory input from the feet contributes to postural maintenance and control.

Answer: A. In older adults, the amplitude of cervical vestibular evoked myogenic potentials (cVEMP) is smaller compared to younger adults, but there is no reduction in amplitude for ocular vestibular evoked myogenic potentials (oVEMP).

Explanation:
The vestibular system, along with visual and somatosensory inputs, plays a critical role in maintaining balance and posture. Let's evaluate each option:

- A. In older adults, cVEMP amplitude is smaller, but oVEMP amplitude is not reduced: This is incorrect. Both cVEMP and oVEMP amplitudes tend to decrease with age due to age-related degeneration of the vestibular system. The statement that oVEMP amplitude does not decrease in older adults is false.
- B. Older adults rely more on the visual system to maintain postural balance: This is correct. As vestibular and somatosensory functions decline with age, older adults often compensate by relying more on visual cues for balance.
- C. Lower limb strength is important for posture and balance: This is correct. Strong lower limbs are essential for maintaining stability and preventing falls, especially in older adults.
- D. Somatosensory input from the feet contributes to postural control: This is correct. Proprioceptive and tactile information from the feet helps the body adjust to changes in posture and terrain.

Thus, the incorrect statement is A. In older adults, the amplitude of cervical vestibular evoked myogenic potentials (cVEMP) is smaller compared to younger adults, but there is no reduction in amplitude for ocular vestibular evoked myogenic potentials (oVEMP).

Question 47:
Which of the following statements about the pathophysiology of left-sided vestibular neuritis is incorrect?
A. Symptoms include severe vertigo, vomiting, and right-beating nystagmus.
B. Typically, nystagmus and vertigo gradually improve over several days due to the recovery of the damaged left vestibular system.
C. In the acute phase, the resting activity of the right vestibular nucleus increases because the left vestibular nucleus's function is reduced or lost, leading to decreased inhibition from the left side.
D. After the onset of the condition, vestibular adaptation occurs, which is related to changes and adjustments in vestibular nucleus activity.

Answer: B. Typically, nystagmus and vertigo gradually improve over several days due to the recovery of the damaged left vestibular system.

Explanation:
Vestibular neuritis is an inflammatory condition affecting the vestibular nerve, leading to acute vertigo and imbalance. Let's evaluate each option:

- A. Symptoms include severe vertigo, vomiting, and right-beating nystagmus: This is correct. In left-sided vestibular neuritis, the loss of left vestibular input causes an imbalance in vestibular tone, leading to right-beating nystagmus (fast phase toward the unaffected side).
- B. Nystagmus and vertigo improve over several days due to recovery of the damaged left vestibular system: This is incorrect. The improvement in symptoms is primarily due to central compensation (vestibular adaptation), not recovery of the damaged vestibular system. The damaged vestibular nerve often does not fully recover.
- C. In the acute phase, the resting activity of the right vestibular nucleus increases due to reduced inhibition from the left side: This is correct. The loss of input from the left vestibular nucleus reduces its inhibitory effect on the right vestibular nucleus, leading to increased activity on the right side.
- D. Vestibular adaptation occurs after the onset, involving changes in vestibular nucleus activity: This is correct. Central compensation (vestibular adaptation) helps restore balance by recalibrating the vestibular system through changes in vestibular nucleus activity.

Thus, the incorrect statement is B. Typically, nystagmus and vertigo gradually improve over several days due to the recovery of the damaged left vestibular system.

Question 48:
Mr. Wang, a 54-year-old man, experienced acute vertigo after a cold. Both the emergency and outpatient physicians observed spontaneous right-beating nystagmus. Pure-tone audiometry results were normal. After using anti-vertigo medication, his symptoms improved, and within a week, he no longer had vertigo or spontaneous nystagmus. What is the most likely diagnosis for this patient?
A. Suspected left vestibular neuritis; absence of vertigo indicates recovery of vestibular function.
B. Suspected right vestibular neuritis; absence of vertigo indicates recovery of vestibular function.
C. Suspected left vestibular neuritis; absence of vertigo does not indicate recovery of vestibular function.
D. Suspected right vestibular neuritis; absence of vertigo does not indicate recovery of vestibular function.

Answer: C. Suspected left vestibular neuritis; absence of vertigo does not indicate recovery of vestibular function.

Explanation:

- Spontaneous right-beating nystagmus: This indicates an imbalance in vestibular tone, with the fast phase of nystagmus beating toward the unaffected side. In this case, the right-beating nystagmus suggests left vestibular hypofunction (e.g., left vestibular neuritis).
- Normal hearing: Vestibular neuritis typically does not affect hearing, as it involves the vestibular nerve, not the cochlear nerve.

- Improvement in symptoms: The resolution of vertigo and nystagmus is due to central compensation (vestibular adaptation), not necessarily recovery of the damaged vestibular system. The absence of vertigo does not mean the vestibular function has fully recovered.

Thus, the correct answer is C. Suspected left vestibular neuritis; absence of vertigo does not indicate recovery of vestibular function.

Question 49:
Which of the following is not a typical symptom of superior semicircular canal dehiscence (SSCD)?
A. Paracusis of Willis
B. Drop attacks
C. Tullio phenomenon
D. Hennebert’s sign

Answer: B. Drop attacks

Explanation:
Superior semicircular canal dehiscence (SSCD) is characterized by a thinning or absence of the bone overlying the superior semicircular canal, leading to abnormal pressure transmission and symptoms such as:

- A. Paracusis of Willis: This is a typical symptom of SSCD. Patients may hear internal sounds (e.g., their own eye movements or heartbeat) unusually loudly.
- C. Tullio phenomenon: This is a typical symptom of SSCD. It refers to vertigo or nystagmus induced by loud sounds or pressure changes.
- D. Hennebert’s sign: This is a typical symptom of SSCD. It refers to vertigo or nystagmus induced by pressure changes in the ear canal (e.g., during Valsalva maneuver).
- B. Drop attacks: This is not a typical symptom of SSCD. Drop attacks are more commonly associated with conditions like Meniere’s disease or vertebrobasilar insufficiency.

Thus, the correct answer is B. Drop attacks.

Question 50:
Which of the following statements about the pathogenesis of benign paroxysmal positional vertigo (BPPV) is correct?

1. Otoconia detach from the ampulla of the semicircular canal.
2. Detached otoconia float freely in the semicircular canal (canalithiasis).
3. Detached otoconia adhere to the cupula (cupulolithiasis).

4. The density of otoconia is equal to that of perilymph.
   A. ①②
   B. ②③
   C. ③④
   D. ①④

Answer: B. ②③

Explanation:
BPPV is caused by displaced otoconia (calcium carbonate crystals) from the utricle, which interfere with the normal function of the semicircular canals. The two main mechanisms are:

- Canalithiasis (②): Otoconia float freely in the endolymph of the semicircular canal, causing abnormal fluid movement and vertigo when the head changes position.
- Cupulolithiasis (③): Otoconia adhere to the cupula of the semicircular canal, making it gravity-sensitive and causing vertigo when the head changes position.
- ① Otoconia detach from the ampulla: This is incorrect. Otoconia detach from the utricle, not the ampulla.
- ④ The density of otoconia is equal to that of perilymph: This is incorrect. Otoconia are denser than endolymph and perilymph, which is why they move under the influence of gravity.

Thus, the correct answer is B. ②③.

6106

Question 1:
Which of the following statements about dysphagia (swallowing disorders) is most appropriate?
A. The prevalence of dysphagia is unrelated to age.
B. Dysphagia includes the inability to move a food bolus from the stomach to the small intestine.
C. Most pediatric swallowing difficulties stem from specific medical diagnoses.
D. Frequent choking while swallowing saliva does not count as dysphagia.

Answer: C. Most pediatric swallowing difficulties stem from specific medical diagnoses.

Explanation:

- A. The prevalence of dysphagia is unrelated to age: This is incorrect. Dysphagia is more prevalent in older adults due to age-related changes in swallowing muscles and neurological conditions.
- B. Dysphagia includes the inability to move a food bolus from the stomach to the small intestine: This is incorrect. Dysphagia refers to difficulty in the oral, pharyngeal, or esophageal phases of swallowing, not the movement of food from the stomach to the small intestine.
- C. Most pediatric swallowing difficulties stem from specific medical diagnoses: This is correct. Pediatric dysphagia is often associated with conditions such as cerebral palsy, developmental delays, or congenital abnormalities.
- D. Frequent choking while swallowing saliva does not count as dysphagia: This is incorrect. Difficulty swallowing saliva is a sign of dysphagia, particularly in the oral or pharyngeal phases.

Thus, the most appropriate statement is C. Most pediatric swallowing difficulties stem from specific medical diagnoses.

Question 2:
Which of the following communication disorders is more likely to occur in females than males?
A. Speech sound disorders
B. Hearing loss
C. Selective mutism
D. Fluency disorders

Answer: C. Selective mutism

Explanation:

- A. Speech sound disorders: These are more common in males than females.
- B. Hearing loss: Hearing loss prevalence is generally similar between genders, though some types (e.g., noise-induced) may be more common in males.
- C. Selective mutism: This condition, characterized by an inability to speak in specific social situations, is more frequently diagnosed in females than males.

- D. Fluency disorders (e.g., stuttering): These are more common in males than females.

Thus, the correct answer is C. Selective mutism.

Question 3:
An aphasia patient has the following conversation with a therapist:
Patient: "I went to my son, my son, son's building, not an apartment, a place (patient intended to say 'place'), to see him... cook a cake."
Therapist: "Okay, please repeat after me: 'place.'" Patient: "... (does not say it)."
Therapist: "Please say 'place.'" Patient: "I can't."
What type of aphasia does this patient most likely have?
A. Conduction aphasia
B. Transcortical sensory aphasia
C. Subcortical aphasia
D. Transcortical motor aphasia

Answer: A. Conduction aphasia

Explanation:
The patient's speech shows:

- Fluent speech with paraphasias (e.g., substituting "place" with "building").
- Difficulty with repetition (unable to repeat "place").
- Relatively preserved comprehension (understands the therapist's instructions).

These features are characteristic of conduction aphasia, which is caused by damage to the arcuate fasciculus, a pathway connecting Broca's and Wernicke's areas. Patients with conduction aphasia typically have:

- Fluent speech with phonemic paraphasias.
- Severe repetition difficulties.
- Relatively intact comprehension and expression.

Thus, the correct answer is A. Conduction aphasia.

Question 4:
Which of the following statements about communication disorders is least appropriate?
A. Speech disorders include abnormalities in pragmatics, fluency, or voice quality.
B. Language disorders are impairments in the use of spoken, written, or symbolic systems.
C. Dysarthria is a speech disorder caused by neurological damage.
D. Individuals with intellectual disabilities may have difficulty understanding abstract vocabulary, which is considered a language disorder.

Answer: A. Speech disorders include abnormalities in pragmatics, fluency, or voice quality.

Explanation:

- A. Speech disorders include abnormalities in pragmatics, fluency, or voice quality: This is incorrect. Pragmatics (the social use of language) is a component of language disorders, not speech disorders. Speech disorders primarily involve articulation, fluency (e.g., stuttering), and voice quality.
- B. Language disorders are impairments in the use of spoken, written, or symbolic systems: This is correct. Language disorders affect comprehension and/or expression of language in various modalities.
- C. Dysarthria is a speech disorder caused by neurological damage: This is correct. Dysarthria is a motor speech disorder resulting from neurological impairment.
- D. Individuals with intellectual disabilities may have difficulty understanding abstract vocabulary, which is considered a language disorder: This is correct. Language disorders often co-occur with intellectual disabilities, particularly in understanding complex or abstract concepts.

Thus, the least appropriate statement is A. Speech disorders include abnormalities in pragmatics, fluency, or voice quality.

Question 5:
In phonological development, the phenomenon of pronouncing "飛機" (airplane) as "胚機" is an example of which common phonological process?
A. Assimilation
B. Stopping
C. Fricativization
D. Fronting

Answer: D. Fronting

Explanation:
Phonological processes are patterns of sound errors that children use to simplify speech as they learn to talk. In this case:

- Fronting (D): This process involves replacing a back sound (e.g., /f/ in "飛機") with a front sound (e.g., /p/ in "胚機"). Fronting is a common phonological process in early speech development.
- Assimilation (A): This refers to when one sound becomes similar to a neighboring sound, which is not the case here.
- Stopping (B): This involves replacing a fricative or affricate with a stop consonant, which is not applicable here.
- Fricativization (C): This involves replacing a stop consonant with a fricative, which is also not applicable here.

Thus, the correct answer is D. Fronting.

Question 6:
Which of the following statements about stuttering is least appropriate?
A. Stuttering typically begins in early childhood.
B. The natural recovery rate before age 6 is quite high.
C. The prevalence of stuttering is higher in girls than boys during school age.
D. Stuttering may be related to genetic factors.

Answer: C. The prevalence of stuttering is higher in girls than boys during school age.

Explanation:

- A. Stuttering typically begins in early childhood: This is correct. Stuttering often emerges between the ages of 2 and 5.
- B. The natural recovery rate before age 6 is quite high: This is correct. Many children who stutter recover spontaneously without intervention.
- C. The prevalence of stuttering is higher in girls than boys during school age: This is incorrect. Stuttering is more prevalent in males than females, with a ratio of about 3:1 to 4:1.
- D. Stuttering may be related to genetic factors: This is correct. Family history and genetic predisposition are significant risk factors for stuttering.

Thus, the least appropriate statement is C. The prevalence of stuttering is higher in girls than boys during school age.

Question 7:
Which of the following methods is most appropriate for assessing the language abilities of bilingual children?
A. Non-standardized assessments should not be used to avoid affecting evaluation results.
B. Different languages share common structural elements, so cultural differences can be ignored during assessment.
C. It is appropriate to switch language codes as needed to achieve the best assessment results.
D. Using an assessment tool in one language is sufficient.

Answer: C. It is appropriate to switch language codes as needed to achieve the best assessment results.

Explanation:

- A. Non-standardized assessments should not be used: This is incorrect. Non-standardized assessments (e.g., language samples, dynamic assessment) are valuable for understanding a bilingual child's language abilities in a natural context.

- B. Cultural differences can be ignored: This is incorrect. Cultural and linguistic differences must be considered to avoid bias and ensure accurate assessment.
- C. Switching language codes is appropriate: This is correct. Code-switching (using both languages) can help elicit the child's best performance and provide a more comprehensive understanding of their language abilities.
- D. Using an assessment tool in one language is sufficient: This is incorrect. Assessing only one language does not provide a complete picture of a bilingual child's language skills.

Thus, the most appropriate method is C. It is appropriate to switch language codes as needed to achieve the best assessment results.

Question 8:
Which of the following statements about dynamic assessment is correct?
A. It is a one-way and structured assessment method.
B. The assessment process typically follows a "intervention-assessment-reintervention" sequence.
C. It is not suitable for children from multicultural or bilingual backgrounds.
D. It is a non-standardized assessment method.

Answer: D. It is a non-standardized assessment method.

Explanation:
Dynamic assessment is an interactive approach that evaluates a child's learning potential and responsiveness to instruction. Let’s evaluate each option:

- A. It is a one-way and structured assessment method: This is incorrect. Dynamic assessment is interactive and flexible, focusing on the child's ability to learn with guidance.
- B. The assessment process typically follows a "intervention-assessment-reintervention" sequence: This is incorrect. The process usually involves pretest-teach-posttest, where the examiner assesses the child's baseline, provides teaching or mediation, and then reassesses to measure learning progress.
- C. It is not suitable for children from multicultural or bilingual backgrounds: This is incorrect. Dynamic assessment is particularly useful for culturally and linguistically diverse children because it focuses on learning potential rather than static knowledge.
- D. It is a non-standardized assessment method: This is correct. Dynamic assessment is non-standardized and tailored to the individual's needs, making it flexible and adaptable.

Thus, the correct answer is D. It is a non-standardized assessment method.

Question 9:
Which of the following statements about specific language impairment (SLI) is most appropriate?
A. The prevalence is similar in males and females.

B. Family history does not affect the prevalence of SLI.
C. Brain imaging of children with SLI shows symmetrical left and right hemispheres.
D. In addition to poor language performance, their non-verbal IQ is significantly lower than that of typical children.

Answer: C. Brain imaging of children with SLI shows symmetrical left and right hemispheres.

Explanation:
Specific language impairment (SLI) is a developmental language disorder not attributable to other conditions. Let's evaluate each option:

- A. The prevalence is similar in males and females: This is incorrect. SLI is more common in males than females.
- B. Family history does not affect the prevalence of SLI: This is incorrect. SLI has a strong genetic component, and family history is a significant risk factor.
- C. Brain imaging of children with SLI shows symmetrical left and right hemispheres: This is correct. Research has shown that children with SLI often exhibit atypical brain asymmetry, with reduced left hemisphere dominance for language processing.
- D. Their non-verbal IQ is significantly lower than that of typical children: This is incorrect. By definition, children with SLI have non-verbal IQ within the normal range. Their language difficulties are disproportionate to their cognitive abilities.

Thus, the most appropriate statement is C. Brain imaging of children with SLI shows symmetrical left and right hemispheres.

Question 10:
Which of the following descriptions of augmentative and alternative communication (AAC) is least appropriate?
A. Using AAC can limit the development of oral expression.
B. Non-aided AAC includes gestures and sign language.
C. Aided AAC includes the use of tablets or communication boards.
D. Using pen and paper for communication is also a form of AAC.

Answer: A. Using AAC can limit the development of oral expression.

Explanation:
AAC refers to methods used to supplement or replace speech for individuals with communication difficulties. Let's evaluate each option:

- A. Using AAC can limit the development of oral expression: This is incorrect. Research shows that AAC does not hinder speech development and may actually support it by reducing frustration and providing a means to practice communication.
- B. Non-aided AAC includes gestures and sign language: This is correct. Non-aided AAC does not require external tools and includes body language, gestures, and sign language.

- C. Aided AAC includes the use of tablets or communication boards: This is correct. Aided AAC involves tools or devices, such as tablets, communication boards, or speech-generating devices.
- D. Using pen and paper for communication is also a form of AAC: This is correct. Low-tech methods like writing or drawing are considered forms of AAC.

Thus, the least appropriate statement is A. Using AAC can limit the development of oral expression.

Question 11:
Which of the following statements about the intervention for auditory neuropathy spectrum disorder (ANSD) is least appropriate?
A. Sometimes, delayed diagnosis leads to the inability to provide necessary treatment plans.
B. Even after diagnosis, there is often disagreement about the best treatment approach.
C. Audiologists typically start by trying hearing aids for these children.
D. Berlin et al. (2010) stated that hearing aids are helpful for most patients, while cochlear implants are effective only for a few.

Answer: D. Berlin et al. (2010) stated that hearing aids are helpful for most patients, while cochlear implants are effective only for a few.

Explanation:

- A. Delayed diagnosis can hinder treatment: This is correct. Early diagnosis is critical for timely intervention in ANSD.
- B. Disagreement exists about the best treatment approach: This is correct. ANSD management is highly individualized, and there is no one-size-fits-all solution.
- C. Hearing aids are often tried first: This is correct. Hearing aids are typically the first intervention, though their effectiveness varies depending on the severity of the disorder.
- D. Hearing aids help most patients, while cochlear implants help only a few: This is incorrect. Research, including studies by Berlin et al., shows that cochlear implants are often more effective than hearing aids for individuals with ANSD, particularly those with severe hearing loss. Hearing aids may not provide sufficient benefit due to the neural dyssynchrony characteristic of ANSD.

Thus, the least appropriate statement is D. Berlin et al. (2010) stated that hearing aids are helpful for most patients, while cochlear implants are effective only for a few.

Question 12:
Which of the following statements about otitis media is most appropriate?
A. Statistically, 90% of preschool children have had at least one episode of acute otitis media, with the peak incidence occurring between 4 and 5 years of age.
B. Serous otitis media is defined as middle ear effusion lasting more than 12 weeks, with common symptoms including ear fullness, hearing loss, and tinnitus.

C. According to WHO guidelines, chronic suppurative otitis media is defined as ear discharge due to inflammation lasting more than 6 months.
D. Acute otitis media can lead to cholesteatoma, which impedes ossicular movement and causes significant conductive hearing loss.

Answer: B. Serous otitis media is defined as middle ear effusion lasting more than 12 weeks, with common symptoms including ear fullness, hearing loss, and tinnitus.

Explanation:

- A. 90% of preschool children have had acute otitis media, peaking at 4–5 years: This is incorrect. The peak incidence of acute otitis media is typically between 6 and 18 months, not 4–5 years.
- B. Serous otitis media is defined as middle ear effusion lasting more than 12 weeks: This is correct. Serous otitis media (also called otitis media with effusion) involves fluid in the middle ear without infection, often causing symptoms like ear fullness, hearing loss, and tinnitus.
- C. Chronic suppurative otitis media is defined as ear discharge lasting more than 6 months: This is incorrect. Chronic suppurative otitis media is defined by persistent ear discharge through a perforated tympanic membrane for more than 2 weeks, not 6 months.
- D. Acute otitis media can lead to cholesteatoma: This is incorrect. Cholesteatoma is typically associated with chronic otitis media, not acute otitis media.

Thus, the most appropriate statement is B. Serous otitis media is defined as middle ear effusion lasting more than 12 weeks, with common symptoms including ear fullness, hearing loss, and tinnitus.

Question 13:
Which of the following statements about noise-induced hearing loss (NIHL) is least appropriate?
A. Different types of noise cause varying degrees of damage; at the same exposure level, continuous noise is more harmful than intermittent noise.
B. When using hearing protection, combining earmuffs with earplugs provides better attenuation, with the key factor being the earmuffs.
C. In the absence of other factors, noise-induced hearing loss typically presents as bilateral and symmetrical sensorineural hearing loss.
D. If hearing loss is not addressed, the rate of deterioration slows as hearing thresholds increase.

Answer: B. When using hearing protection, combining earmuffs with earplugs provides better attenuation, with the key factor being the earmuffs.

Explanation:

- A. Continuous noise is more harmful than intermittent noise at the same exposure level: This is correct. Continuous noise exposure generally poses a greater risk than intermittent noise due to prolonged exposure.
- B. Combining earmuffs with earplugs provides better attenuation, with the key factor being the earmuffs: This is incorrect. While combining earmuffs and earplugs does provide better

attenuation, the key factor is the combined effect, not just the earmuffs. The total attenuation is not simply additive but follows a specific formula.

- C. Noise-induced hearing loss is typically bilateral and symmetrical: This is correct. NIHL usually affects both ears equally, assuming similar noise exposure.
- D. Hearing loss deterioration slows as thresholds increase: This is correct. As hearing thresholds worsen, the rate of further deterioration often slows due to the nonlinear nature of hearing damage.

Thus, the least appropriate statement is B. When using hearing protection, combining earmuffs with earplugs provides better attenuation, with the key factor being the earmuffs.

Question 14:
Xiao Wang is a long-time sufferer of Meniere's disease. Which of the following descriptions about his daily life is least appropriate?
A. In addition to moderate sensorineural hearing loss, he often experiences dizziness and vomiting.
B. He has fluctuating low- to mid-frequency hearing loss, which can affect the effectiveness of hearing aids.
C. The condition is currently thought to be caused by an unknown accumulation of endolymph in the saccule.
D. The condition typically occurs bilaterally and is accompanied by severe tinnitus and vertigo.

Answer: D. The condition typically occurs bilaterally and is accompanied by severe tinnitus and vertigo.

Explanation:
Meniere's disease is characterized by a triad of symptoms: sensorineural hearing loss, tinnitus, and vertigo. Let's evaluate each option:

- A. Moderate sensorineural hearing loss with dizziness and vomiting: This is correct. Meniere's disease often causes fluctuating hearing loss and episodic vertigo, which can lead to nausea and vomiting.
- B. Fluctuating low- to mid-frequency hearing loss affecting hearing aid effectiveness: This is correct. The fluctuating nature of hearing loss in Meniere's disease can make hearing aid fitting and use challenging.
- C. Caused by endolymph accumulation in the saccule: This is correct. Meniere's disease is associated with endolymphatic hydrops, an abnormal buildup of fluid in the inner ear, though the exact cause is unknown.
- D. Typically occurs bilaterally with severe tinnitus and vertigo: This is incorrect. Meniere's disease is usually unilateral (affecting one ear) in the early stages, though it can become bilateral over time in some cases.

Thus, the least appropriate statement is D. The condition typically occurs bilaterally and is accompanied by severe tinnitus and vertigo.

Question 15:
Recurrent otitis media can affect the communication and learning abilities of preschool children. Which of the following statements is least appropriate?
A. It is often defined as repeated acute episodes within 3 to 6 months, with the highest incidence in winter and spring.
B. Children with cleft lip/palate, craniofacial abnormalities, or Down syndrome have a higher incidence than typical children.
C. If hearing thresholds continue to worsen without improvement, a tympanostomy or grommet placement may be recommended.
D. It usually causes severe or profound hearing loss immediately.

Answer: D. It usually causes severe or profound hearing loss immediately.

Explanation:
Recurrent otitis media is a common condition in young children and can impact hearing and development. Let’s evaluate each option:

- A. Defined as repeated acute episodes within 3 to 6 months, with higher incidence in winter and spring: This is correct. Recurrent otitis media is characterized by multiple episodes, often peaking during colder months due to increased respiratory infections.
- B. Higher incidence in children with cleft lip/palate, craniofacial abnormalities, or Down syndrome: This is correct. These conditions are associated with Eustachian tube dysfunction, increasing the risk of otitis media.
- C. Tympanostomy or grommet placement may be recommended for persistent hearing loss: This is correct. Surgical intervention is often considered when hearing loss persists or recurrent infections do not resolve with medical treatment.
- D. It usually causes severe or profound hearing loss immediately: This is incorrect. Recurrent otitis media typically causes mild to moderate conductive hearing loss due to fluid buildup in the middle ear, not severe or profound hearing loss. Severe hearing loss is rare and usually occurs only with complications like chronic suppurative otitis media or cholesteatoma.

Thus, the least appropriate statement is D. It usually causes severe or profound hearing loss immediately.

Question 16:
Which of the following descriptions about age-related hearing loss (ARHL) and its impact is least appropriate?
A. It is a sensorineural hearing loss, with high-frequency hearing sensitivity declining more significantly than mid- to low-frequency hearing.
B. Degeneration of central auditory function and cognitive decline result in slower auditory processing compared to younger individuals.
C. Due to insufficient auditory input, individuals often mishear parts of sentences and misunderstand the meaning.

D. The cortical reticular system becomes more complex with age, leading to better speech recognition in challenging listening environments compared to younger individuals.

Answer: D. The cortical reticular system becomes more complex with age, leading to better speech recognition in challenging listening environments compared to younger individuals.

Explanation:
Age-related hearing loss (ARHL), or presbycusis, is characterized by progressive hearing loss, particularly in high frequencies, and is associated with aging. Let's evaluate each option:

- A. High-frequency hearing sensitivity declines more than mid- to low-frequency hearing: This is correct. ARHL typically begins with high-frequency hearing loss, affecting the perception of consonants and speech clarity.
- B. Central auditory and cognitive decline slow auditory processing: This is correct. Older adults often experience slower auditory processing and difficulty understanding speech in noisy environments due to age-related changes in the brain.
- C. Mishearing parts of sentences and misunderstanding meaning: This is correct. ARHL can lead to difficulty distinguishing speech sounds, especially in noisy or complex listening situations.
- D. The cortical reticular system becomes more complex, improving speech recognition in challenging environments: This is incorrect. With age, the auditory system and brain undergo degenerative changes, leading to reduced speech recognition in challenging environments, not improved performance.

Thus, the least appropriate statement is D. The cortical reticular system becomes more complex with age, leading to better speech recognition in challenging listening environments compared to younger individuals.

Question 17:
Which of the following statements about middle ear function assessment in infants is least appropriate?
A. When using a 220 Hz probe tone to assess middle ear compliance in newborns, a W-shaped or flat tympanogram may sometimes appear.
B. Newborns' ear canal walls are soft and can be easily affected by pressure, altering middle ear test results.
C. Infants must be at least 10 months old to produce a single-peak tympanogram.
D. Infants are more likely to produce a single-peak tympanogram with a 678 Hz or 1000 Hz probe tone than with a 226 Hz tone.

Answer: C. Infants must be at least 10 months old to produce a single-peak tympanogram.

Explanation:
Middle ear assessment in infants requires special considerations due to anatomical and developmental differences. Let's evaluate each option:

- A. A W-shaped or flat tympanogram may appear with a 220 Hz probe tone in newborns: This is correct. Newborns often produce atypical tympanograms due to their small ear canal volume and compliant ear canal walls.
- B. Newborns' soft ear canal walls can be affected by pressure, altering test results: This is correct. The pliability of infants' ear canals can lead to inaccurate measurements if not accounted for.
- C. Infants must be at least 10 months old to produce a single-peak tympanogram: This is incorrect. Single-peak tympanograms can be observed in infants younger than 10 months, especially when using higher-frequency probe tones (e.g., 678 Hz or 1000 Hz).
- D. A single-peak tympanogram is more likely with a 678 Hz or 1000 Hz probe tone than with a 226 Hz tone: This is correct. Higher-frequency probe tones are more appropriate for infants because they better reflect the mechanical properties of the infant middle ear.

Thus, the least appropriate statement is C. Infants must be at least 10 months old to produce a single-peak tympanogram.

Question 18:
Which of the following statements about remote microphone systems (RMS) is most appropriate?
A. The signal-to-noise ratio (SNR) improvement, according to ASHA recommendations, should be at least 35 dB.
B. RMS primarily consists of a transmitter and a sound source cable.
C. All in-the-ear hearing aids can be paired with a remote microphone system.
D. Connection methods include sound field, induction loop, or direct audio input (DAI) devices.

Answer: D. Connection methods include sound field, induction loop, or direct audio input (DAI) devices.

Explanation:
Remote microphone systems (RMS) are assistive listening devices that improve speech understanding in noisy or distant listening situations. Let's evaluate each option:

- A. ASHA recommends an SNR improvement of at least 35 dB: This is incorrect. ASHA recommends an SNR improvement of at least 15–20 dB for effective speech understanding, not 35 dB.
- B. RMS primarily consists of a transmitter and a sound source cable: This is incorrect. RMS typically consists of a microphone (transmitter) worn by the speaker and a receiver worn by the listener, not a sound source cable.
- C. All in-the-ear hearing aids can be paired with RMS: This is incorrect. Not all in-the-ear hearing aids are compatible with RMS. Compatibility depends on the hearing aid's technology and connectivity options.
- D. Connection methods include sound field, induction loop, or direct audio input (DAI) devices: This is correct. RMS can be connected to hearing aids or cochlear implants via various methods, including sound field systems, induction loops, or direct audio input.

Thus, the most appropriate statement is D. Connection methods include sound field, induction loop, or direct audio input (DAI) devices.

Question 19:
Which of the following statements about hearing loss is most appropriate?
A. Noise-induced hearing loss only occurs in adults.
B. Long-term exposure to loud noise typically results in greater sensitivity loss in the low-frequency range.
C. Hearing loss due to aural atresia is primarily mixed hearing loss.
D. Meningitis often leads to severe or profound hearing loss.

Answer: D. Meningitis often leads to severe or profound hearing loss.

Explanation:
Let's evaluate each option:

- A. Noise-induced hearing loss only occurs in adults: This is incorrect. Noise-induced hearing loss can occur in individuals of any age, including children and adolescents, especially with exposure to loud music or recreational noise.
- B. Long-term noise exposure causes greater sensitivity loss in the low-frequency range: This is incorrect. Noise-induced hearing loss typically affects the high-frequency range first (e.g., 3–6 kHz), not the low frequencies.
- C. Hearing loss due to aural atresia is primarily mixed hearing loss: This is incorrect. Aural atresia (congenital absence or closure of the ear canal) typically causes conductive hearing loss, not mixed hearing loss. Mixed hearing loss would involve both conductive and sensorineural components.
- D. Meningitis often leads to severe or profound hearing loss: This is correct. Meningitis, particularly bacterial meningitis, can cause damage to the cochlea or auditory nerve, resulting in severe to profound sensorineural hearing loss.

Thus, the most appropriate statement is D. Meningitis often leads to severe or profound hearing loss.

Question 20:
Which of the following statements about Schuknecht's (1989) classification of congenital aural atresia is least appropriate?
A. The classification includes Type A, B, C, and D.
B. Type A is meatal atresia, Type B is partial atresia, and Type C is total atresia.
C. Type B refers to ear canal stenosis where the eardrum is usually not visible, but the ossicles are normal.
D. Hypopneumatic atresia refers to poor mastoid pneumatization and facial nerve abnormalities in the temporal bone.

Answer: B. Type A is meatal atresia, Type B is partial atresia, and Type C is total atresia.

Explanation:
Schuknecht’s classification of congenital aural atresia is based on the degree of ear canal and middle ear abnormalities. Let’s evaluate each option:

- A. The classification includes Type A, B, C, and D: This is correct. Schuknecht’s classification system divides aural atresia into four types (A, B, C, and D).
- B. Type A is meatal atresia, Type B is partial atresia, and Type C is total atresia: This is incorrect. Schuknecht’s classification is as follows:
  - Type A: Minor meatal atresia (narrowing of the ear canal).
  - Type B: Partial atresia with stenosis and malformation of the ossicles.
  - Type C: Total atresia with a completely absent ear canal and malformed ossicles.
  - Type D: Hypopneumatic atresia with poor mastoid pneumatization and facial nerve abnormalities.
- C. Type B refers to ear canal stenosis where the eardrum is usually not visible, but the ossicles are normal: This is incorrect. In Type B, the ossicles are abnormal, not normal.
- D. Hypopneumatic atresia refers to poor mastoid pneumatization and facial nerve abnormalities: This is correct. Type D (hypopneumatic atresia) involves underdeveloped mastoid air cells and potential facial nerve anomalies.

Thus, the least appropriate statement is B. Type A is meatal atresia, Type B is partial atresia, and Type C is total atresia.

Question 21:
Which of the following interpretations of the audiogram is least appropriate?
A. It represents mixed hearing loss.
B. There is an abnormality in the outer or middle ear conduction.
C. If sound bypasses the outer or middle ear and directly stimulates the inner ear, the hearing thresholds are normal.
D. The circle symbols represent unmasked air conduction.

Answer: A. It represents mixed hearing loss.

Explanation:
To determine the correct interpretation, let’s analyze the audiogram and the options:

1. Audiogram Analysis:
   - Air Conduction (AC): Represented by circles (right ear) and crosses (left ear). If these symbols show elevated thresholds (e.g., above 20 dB HL), it indicates hearing loss.
   - Bone Conduction (BC): Represented by angle brackets (e.g., < or >). If BC thresholds are normal (e.g., within 0–20 dB HL) but AC thresholds are elevated, it suggests conductive

hearing loss. If both AC and BC thresholds are elevated, it suggests sensorineural or mixed hearing loss.

2. Weber Test:
   - The Weber test lateralizes to the right (R) or left (L) ear. Lateralization to the poorer ear suggests conductive hearing loss, while lateralization to the better ear suggests sensorineural hearing loss.
3. Options Evaluation:
   - A. It represents mixed hearing loss: This is incorrect if the audiogram shows a significant air-bone gap (elevated AC with normal BC), which would indicate conductive hearing loss, not mixed hearing loss. Mixed hearing loss requires both conductive and sensorineural components (elevated AC and BC).
   - B. There is an abnormality in the outer or middle ear conduction: This is correct if the audiogram shows an air-bone gap, indicating a problem in the outer or middle ear.
   - C. If sound bypasses the outer or middle ear and directly stimulates the inner ear, the hearing thresholds are normal: This is correct if BC thresholds are normal, indicating that the inner ear (cochlea) is functioning properly.
   - D. The circle symbols represent unmasked air conduction: This is correct. Circles typically represent unmasked air conduction thresholds for the right ear.

Thus, the least appropriate interpretation is A. It represents mixed hearing loss.

Question 22:
Which of the following is not a cause of sensorineural hearing loss?
A. Cholesteatoma
B. Auditory neuropathy
C. Inner ear malformation
D. Hair cell damage in the inner ear

Answer: A. Cholesteatoma

Explanation:
Sensorineural hearing loss (SNHL) results from damage to the inner ear (cochlea) or the auditory nerve. Let’s evaluate each option:

- A. Cholesteatoma: This is not a cause of SNHL. Cholesteatoma is a growth of skin in the middle ear that can cause conductive hearing loss by damaging the ossicles or eardrum.
- B. Auditory neuropathy: This is a cause of SNHL. Auditory neuropathy involves dysfunction of the auditory nerve or inner ear hair cells, leading to impaired sound transmission to the brain.
- C. Inner ear malformation: This is a cause of SNHL. Congenital malformations of the cochlea or auditory structures can result in hearing loss.

- D. Hair cell damage in the inner ear: This is a cause of SNHL. Damage to the hair cells in the cochlea, often due to noise exposure, aging, or ototoxic drugs, leads to hearing loss.

Thus, the correct answer is A. Cholesteatoma, as it causes conductive hearing loss, not sensorineural hearing loss.

Question 23:
Which of the following statements about the Eustachian tube is most appropriate?
A. The Eustachian tube is longer in adults than in children.
B. Abnormal patency of the Eustachian tube does not cause autophony.
C. The Eustachian tube is horizontally oriented in both adults and children.
D. Eustachian tube function is unaffected in individuals with cleft lip/palate.

Answer: A. The Eustachian tube is longer in adults than in children.

Explanation:
The Eustachian tube connects the middle ear to the nasopharynx and plays a crucial role in pressure regulation and fluid drainage. Let's evaluate each option:

- A. The Eustachian tube is longer in adults than in children: This is correct. The Eustachian tube is shorter and more horizontal in children, making them more prone to middle ear infections. As children grow, the tube lengthens and becomes more vertical.
- B. Abnormal patency of the Eustachian tube does not cause autophony: This is incorrect. Patulous Eustachian tube (abnormal openness) can cause autophony, where individuals hear their own voice or breathing loudly.
- C. The Eustachian tube is horizontally oriented in both adults and children: This is incorrect. The Eustachian tube is more horizontal in children but becomes more vertical with age.
- D. Eustachian tube function is unaffected in individuals with cleft lip/palate: This is incorrect. Cleft lip/palate often leads to Eustachian tube dysfunction due to abnormal muscle insertion and poor tube opening, increasing the risk of otitis media.

Thus, the most appropriate statement is A. The Eustachian tube is longer in adults than in children.

Question 24:
Which of the following statements about otosclerosis is least appropriate?
A. It is more common in Caucasians and less common in Asians and Africans.
B. It usually occurs bilaterally, but the progression of hearing loss may differ between ears.
C. The incidence is higher in males than females, especially during adolescence.
D. A characteristic dip at 2000 Hz on the audiogram, known as the Carhart notch, is present.

Answer: C. The incidence is higher in males than females, especially during adolescence.

Explanation:
Otosclerosis is a condition where abnormal bone growth in the middle ear causes fixation of the stapes, leading to conductive hearing loss. Let's evaluate each option:

- A. It is more common in Caucasians and less common in Asians and Africans: This is correct. Otosclerosis has a higher prevalence in Caucasian populations.
- B. It usually occurs bilaterally, but the progression of hearing loss may differ between ears: This is correct. Otosclerosis often affects both ears, but the severity and progression can vary.
- C. The incidence is higher in males than females, especially during adolescence: This is incorrect. Otosclerosis is more common in females than males, and it typically presents in early adulthood (20–40 years), not adolescence.
- D. A characteristic dip at 2000 Hz on the audiogram, known as the Carhart notch, is present: This is correct. The Carhart notch is a hallmark of otosclerosis, reflecting the mechanical resonance of the ossicular chain.

Thus, the least appropriate statement is C. The incidence is higher in males than females, especially during adolescence.

Question 25:
Which of the following statements about presbycusis (age-related hearing loss) is least appropriate?
A. The likelihood of hearing impairment in older adults increases with age.
B. Degeneration of the stria vascularis is the primary cause of presbycusis.
C. Presbycusis typically begins with high-frequency hearing loss.
D. Increasing volume is more helpful than slowing speech for improving speech understanding.

Answer: D. Increasing volume is more helpful than slowing speech for improving speech understanding.

Explanation:
Presbycusis is the gradual, bilateral hearing loss associated with aging. Let's evaluate each option:

- A. The likelihood of hearing impairment increases with age: This is correct. Hearing loss prevalence rises significantly with age, affecting over 50% of individuals aged 75 and older.
- B. Degeneration of the stria vascularis is the primary cause of presbycusis: This is correct. The stria vascularis, which maintains the endocochlear potential, degenerates with age, contributing to hearing loss.
- C. Presbycusis typically begins with high-frequency hearing loss: This is correct. High-frequency hearing loss is a hallmark of presbycusis, affecting the perception of consonants and speech clarity.
- D. Increasing volume is more helpful than slowing speech for improving speech understanding: This is incorrect. While amplification (increasing volume) can help, slowing speech and using clear communication strategies are often more effective for improving understanding in individuals with presbycusis, as they struggle with speech processing and temporal resolution.

Thus, the least appropriate statement is D. Increasing volume is more helpful than slowing speech for improving speech understanding.

Question 26:
According to ASHA (1997) guidelines for preschool hearing screening, which of the following statements is most appropriate?
A. Use 500, 2000, and 4000 Hz as screening frequencies, omitting 1000 Hz, to save time and assess low- and high-frequency hearing.
B. Use 500, 1000, and 2000 Hz as screening frequencies, omitting 4000 Hz, to save time and because it has less impact on speech.
C. Use 1000, 2000, and 4000 Hz as screening frequencies, omitting 500 Hz, to save time and because low frequencies are easily distorted by environmental noise.
D. The recommended screening intensity is 10–15 dB.

Answer: C. Use 1000, 2000, and 4000 Hz as screening frequencies, omitting 500 Hz, to save time and because low frequencies are easily distorted by environmental noise.

Explanation:
ASHA guidelines for hearing screening aim to efficiently identify hearing loss that could impact speech and language development. Let’s evaluate each option:

- A. Use 500, 2000, and 4000 Hz, omitting 1000 Hz: This is incorrect. 1000 Hz is a critical frequency for speech perception and should not be omitted.
- B. Use 500, 1000, and 2000 Hz, omitting 4000 Hz: This is incorrect. 4000 Hz is important for detecting high-frequency hearing loss, which is common in noise-induced or age-related hearing loss.
- C. Use 1000, 2000, and 4000 Hz, omitting 500 Hz: This is correct. 500 Hz is often omitted in screening because low-frequency sounds are more susceptible to environmental noise interference, and 1000, 2000, and 4000 Hz are more critical for speech perception.
- D. The recommended screening intensity is 10–15 dB: This is incorrect. ASHA recommends screening at 20–25 dB HL, not 10–15 dB, to ensure detection of mild hearing loss.

Thus, the most appropriate statement is C. Use 1000, 2000, and 4000 Hz as screening frequencies, omitting 500 Hz, to save time and because low frequencies are easily distorted by environmental noise.

Question 27:
Ms. Chen reports two issues: (1) frequent tinnitus in her left ear, and (2) dizziness when exposed to loud sounds. Which of the following does issue #2 (dizziness with loud sounds) most likely indicate?
A. Tullio phenomenon
B. Hennebert’s sign

C. Hyperacusis
D. Schwartze’s sign

Answer: A. Tullio phenomenon

Explanation:
Let’s evaluate each option:

- A. Tullio phenomenon: This is correct. Tullio phenomenon refers to dizziness or vertigo triggered by loud sounds, often due to abnormal pressure transmission in the inner ear (e.g., superior semicircular canal dehiscence).
- B. Hennebert’s sign: This refers to dizziness or nystagmus caused by pressure changes in the ear canal (e.g., during the Valsalva maneuver), not loud sounds.
- C. Hyperacusis: This refers to heightened sensitivity to sound, causing discomfort or pain, but it does not involve dizziness.
- D. Schwartze’s sign: This refers to a reddish hue visible through the tympanic membrane in cases of otosclerosis, unrelated to dizziness or loud sounds.

Thus, the correct answer is A. Tullio phenomenon.

Question 28:
Which of the following statements about individuals with hearing impairment is least appropriate?
A. Individuals with hearing impairment have better vowel discrimination but struggle with consonant discrimination, especially fricatives.
B. Cochlear implant users do not need visual feedback during initial training to achieve excellent results in auditory discrimination tasks.
C. Individuals with hearing impairment can still learn to use acoustic cues to distinguish speech sounds.
D. Speech perception difficulties may be accompanied by otitis media, language disorders, and articulation problems.

Answer: B. Cochlear implant users do not need visual feedback during initial training to achieve excellent results in auditory discrimination tasks.

Explanation:
Let’s evaluate each option:

- A. Better vowel discrimination but difficulty with consonants, especially fricatives: This is correct. Vowels are louder and lower in frequency, making them easier to hear, while consonants (especially fricatives like /s/ and /f/) are softer and higher in frequency, making them harder to distinguish.
- B. Cochlear implant users do not need visual feedback during initial training: This is incorrect. Visual feedback (e.g., lip-reading, visual cues) is often critical during the initial stages of cochlear implant training to help users associate sounds with meaning and improve auditory discrimination.

- C. Individuals with hearing impairment can learn to use acoustic cues: This is correct. With training, individuals with hearing loss can learn to use residual hearing and acoustic cues to improve speech perception.
- D. Speech perception difficulties may accompany otitis media, language disorders, and articulation problems: This is correct. Hearing loss, especially in children, can lead to speech and language delays, articulation issues, and difficulties with speech perception.

Thus, the least appropriate statement is B. Cochlear implant users do not need visual feedback during initial training to achieve excellent results in auditory discrimination tasks.

Question 29:
Which of the following descriptions about the referral and screening of children with language disorders is least appropriate?
A. Referral for language disorder assessment can occur at any time, including at birth, during infancy, or in school-age years.
B. Children with language disorders can be referred to speech-language pathology services by parents or school teachers.
C. After referral to speech-language pathology services, therapists can use screening tests to diagnose the child’s language problems.
D. Follow-up assessments after referral can be conducted by a multidisciplinary team of experts.

Answer: C. After referral to speech-language pathology services, therapists can use screening tests to diagnose the child’s language problems.

Explanation:
Let’s evaluate each option:

- A. Referral can occur at any time: This is correct. Language disorders can be identified and referred for assessment at any stage, from infancy to school age.
- B. Referral can be made by parents or teachers: This is correct. Parents and teachers are often the first to notice language difficulties and can initiate referrals.
- C. Screening tests can diagnose language problems: This is incorrect. Screening tests are used to identify potential issues, but a comprehensive diagnostic evaluation is required to diagnose language disorders. Screening tests alone are not sufficient for diagnosis.
- D. Follow-up assessments can involve a multidisciplinary team: This is correct. A team approach (e.g., speech-language pathologists, psychologists, educators) is often used for comprehensive assessment and intervention planning.

Thus, the least appropriate statement is C. After referral to speech-language pathology services, therapists can use screening tests to diagnose the child’s language problems.

Question 30:
Which of the following is not a phonological process in young children?
A. Reducing CVC syllable structures to CV structures
B. Syllable repetition
C. Palatalization
D. Stopping

Answer: B. Syllable repetition

Explanation:
Phonological processes are patterns of sound errors that children use to simplify speech as they learn to talk. Let’s evaluate each option:

- A. Reducing CVC syllable structures to CV structures: This is a common phonological process known as final consonant deletion, where the final consonant in a syllable is omitted (e.g., "cat" becomes "ca").
- B. Syllable repetition: This is not a recognized phonological process. Syllable repetition (e.g., "baba" for "bottle") is more related to early babbling or stuttering, not systematic sound simplification.
- C. Palatalization: This is a phonological process where non-palatal sounds are replaced with palatal sounds (e.g., "shoe" becomes "sue").
- D. Stopping: This is a phonological process where fricatives or affricates are replaced with stop consonants (e.g., "sun" becomes "tun").

Thus, the correct answer is B. Syllable repetition, as it is not a recognized phonological process.

Question 31:
Experts recommend that deaf children be exposed to visual language (e.g., sign language) from birth. What is the most appropriate primary purpose of this recommendation?
A. To take advantage of the sensitive period for language acquisition and establish a language foundation.
B. Because severe hearing loss prevents the learning of spoken language.
C. Learning sign language supports the development of spoken language later.
D. Because parents are deaf and cannot provide spoken language input, so the child must learn sign language.

Answer: A. To take advantage of the sensitive period for language acquisition and establish a language foundation.

Explanation:
The critical period for language acquisition is the time during which the brain is most receptive to learning language. Let’s evaluate each option:

- A. To take advantage of the sensitive period for language acquisition: This is correct. Early exposure to visual language (e.g., sign language) ensures that deaf children develop a strong language foundation during this critical period, which supports cognitive and social development.

- B. Severe hearing loss prevents the learning of spoken language: This is incorrect. While severe hearing loss can make spoken language acquisition challenging, it does not prevent it entirely, especially with interventions like hearing aids or cochlear implants.
- C. Learning sign language supports spoken language development: This is partially correct but not the primary reason. While sign language can support spoken language development, the main goal is to ensure early language access.
- D. Parents are deaf and cannot provide spoken language input: This is incorrect. The recommendation applies regardless of parents' hearing status, as the focus is on the child's language development.

Thus, the most appropriate answer is A. To take advantage of the sensitive period for language acquisition and establish a language foundation.

Question 32:
When primary caregivers use a child's visual attention to provide language stimulation, which of the following statements is most appropriate?
A. Language stimulation is only effective when the infant's attention is on the caregiver.
B. Hearing children do not need visual attention for language stimulation because they use spoken language.
C. Infants' visual attention cannot be elicited; caregivers must wait for the infant to initiate eye contact before providing language stimulation.
D. When the infant makes eye contact, the caregiver can immediately provide language stimulation.

Answer: D. When the infant makes eye contact, the caregiver can immediately provide language stimulation.

Explanation:
Visual attention is a key component of early language development, especially for deaf or hard-of-hearing children. Let's evaluate each option:

- A. Language stimulation is only effective when the infant's attention is on the caregiver: This is incorrect. While attention is important, caregivers can also use strategies to attract and maintain the infant's attention.
- B. Hearing children do not need visual attention for language stimulation: This is incorrect. Visual attention (e.g., eye contact, gestures) is important for all children, including hearing children, as it supports joint attention and language learning.
- C. Infants' visual attention cannot be elicited: This is incorrect. Caregivers can use gestures, facial expressions, and objects to elicit and maintain infants' visual attention.
- D. When the infant makes eye contact, the caregiver can immediately provide language stimulation: This is correct. Eye contact is a natural opportunity for caregivers to engage the infant and provide meaningful language input.

Thus, the most appropriate statement is D. When the infant makes eye contact, the caregiver can immediately provide language stimulation.

Question 33:
Which of the following sequences correctly represents the typical order of speech development in infants?

1. Babbling
2. Cooing
3. Variegated babbling
4. Reduplicated babbling
   A. ①→②→④→③
   B. ②→①→③→④
   C. ①→②→③→④
   D. ②→①→④→③

Answer: D. ②→①→④→③

Explanation:
The typical sequence of speech development in infants is as follows:

1. Cooing (②): Around 2–4 months, infants produce vowel-like sounds (e.g., "oo," "aa").
2. Babbling (①): Around 6 months, infants begin producing repetitive consonant-vowel combinations (e.g., "ba-ba," "ma-ma").
3. Reduplicated babbling (④): Around 6–9 months, infants produce repeated syllables (e.g., "ba-ba-ba").
4. Variegated babbling (③): Around 9–12 months, infants produce varied syllables with different consonants and vowels (e.g., "ba-da-ma").

Thus, the correct sequence is D. ②→①→④→③.

Question 34:
Which of the following methods is most appropriate for testing a child's pragmatic language skills?
A. Here are some pictures; please point to the word I say.
B. Please rearrange the following sentences into a logical order.
C. Please repeat the sentence I say.
D. Please tell me: What would you do if you got lost on the street?

Answer: D. Please tell me: What would you do if you got lost on the street?

Explanation:
Pragmatic language skills involve the social use of language, including understanding and producing appropriate communication in context. Let's evaluate each option:

- A. Pointing to pictures based on spoken words: This tests vocabulary and comprehension, not pragmatic skills.
- B. Rearranging sentences into a logical order: This tests syntactic and sequencing skills, not pragmatic skills.
- C. Repeating sentences: This tests auditory memory and articulation, not pragmatic skills.
- D. Responding to a social scenario (e.g., getting lost): This is correct. It assesses the child's ability to use language appropriately in a social context, which is a key aspect of pragmatic language.

Thus, the most appropriate method is D. Please tell me: What would you do if you got lost on the street?

Question 35:
Ai Ai, a 2.5-year-old, reacts to sound-making activities like other children but does not imitate others in any situation, rarely uses gestures, speaks only about 5 words, and cannot follow single-step instructions or understand questions. Which of the following is least likely to be her medical diagnosis?
A. Language impairments
B. Social communication disorders
C. Developmental delay
D. Language delay

Answer: B. Social communication disorders

Explanation:
Ai Ai's symptoms include limited speech, lack of imitation, poor gesture use, and difficulty understanding instructions and questions. Let's evaluate each option:

- A. Language impairments: This is likely. Her limited vocabulary and difficulty understanding instructions suggest language impairments.
- B. Social communication disorders: This is least likely. Social communication disorders (e.g., autism spectrum disorder) typically involve difficulties with social interaction, such as lack of eye contact, limited interest in others, and challenges with social reciprocity, which are not mentioned here.
- C. Developmental delay: This is likely. Her overall delays in language and communication suggest a broader developmental delay.
- D. Language delay: This is likely. Her limited speech and comprehension difficulties are consistent with language delay.

Thus, the least likely diagnosis is B. Social communication disorders.

Question 36:
Which of the following statements about speech recognition threshold (SRT) and word recognition test (WRT) is least appropriate?
A. SRT is the lowest volume at which a person can repeat at least 50% of the speech stimuli.
B. WRT most commonly tests the recognition of trisyllabic words.
C. SRT and WRT are used to understand the listener’s auditory skills.
D. WRT assesses the listener’s ability to recognize speech.

Answer: B. WRT most commonly tests the recognition of trisyllabic words.

Explanation:
Let’s evaluate each option:

- A. SRT is the lowest volume at which a person can repeat at least 50% of the speech stimuli: This is correct. SRT measures the lowest hearing level at which a person can correctly repeat 50% of spondee words (two-syllable words with equal stress, e.g., "baseball").
- B. WRT most commonly tests the recognition of trisyllabic words: This is incorrect. WRT typically uses monosyllabic words (e.g., "cat," "dog") to assess speech recognition ability, not trisyllabic words.
- C. SRT and WRT are used to understand the listener’s auditory skills: This is correct. Both tests evaluate different aspects of auditory perception and speech understanding.
- D. WRT assesses the listener’s ability to recognize speech: This is correct. WRT measures the percentage of words a person can correctly identify at a comfortable listening level.

Thus, the least appropriate statement is B. WRT most commonly tests the recognition of trisyllabic words.

Question 37:
Which of the following situations is least likely to require referral to a speech-language pathologist for speech assessment?
A. A 3.5-year-old who cannot produce all vowels.
B. A 5-year-old who cannot correctly produce /ts/.
C. A 6-year-old who says "阿" instead of "安."
D. A 7-year-old whose /s/ sound is distorted after losing front teeth.

Answer: D. A 7-year-old whose /s/ sound is distorted after losing front teeth.

Explanation:
Let’s evaluate each option:

- A. A 3.5-year-old who cannot produce all vowels: This warrants referral. By age 3, children should be able to produce all vowels correctly.
- B. A 5-year-old who cannot correctly produce /ts/: This warrants referral. By age 5, children should be able to produce most consonant sounds, including affricates like /ts/.

- C. A 6-year-old who says "阿" instead of "安": This warrants referral. By age 6, children should have mastered most speech sounds and syllable structures.
- D. A 7-year-old whose /s/ sound is distorted after losing front teeth: This is least likely to require referral. Temporary distortion of /s/ after losing front teeth is common and often resolves once permanent teeth grow in.

Thus, the least likely situation requiring referral is D. A 7-year-old whose /s/ sound is distorted after losing front teeth.

Question 38:
Which of the following is an example of a suprasegmental error in children with hearing impairment?
A. Errors in stress or tone.
B. Nasalized speech.
C. Substituting velar sounds for alveolar sounds.
D. Omitting the final sound of "ㄠ."

Answer: A. Errors in stress or tone.

Explanation:
Suprasegmental errors involve prosody, which includes stress, intonation, rhythm, and tone. Let's evaluate each option:

- A. Errors in stress or tone: This is correct. Suprasegmental errors include incorrect stress patterns (e.g., emphasizing the wrong syllable) or tone (e.g., monotone speech).
- B. Nasalized speech: This is a segmental error related to articulation, not suprasegmental features.
- C. Substituting velar sounds for alveolar sounds: This is a segmental error involving place of articulation, not suprasegmental features.
- D. Omitting the final sound of "ㄠ": This is a segmental error involving syllable structure, not suprasegmental features.

Thus, the correct answer is A. Errors in stress or tone.

Question 39:
Which of the following is not a typical English speech error pattern in children with hearing loss/deafness?
A. Substituting diphthongs for monophthongs.
B. Substituting monophthongs for diphthongs.
C. Substituting voiced sounds for voiceless sounds.
D. Substituting voiceless sounds for voiced sounds.

Answer: A. Substituting diphthongs for monophthongs.

Explanation:
Children with hearing loss often exhibit specific speech error patterns due to difficulties perceiving and producing speech sounds. Let’s evaluate each option:

- A. Substituting diphthongs for monophthongs: This is not a typical error. Diphthongs are more complex than monophthongs, so children with hearing loss are more likely to simplify diphthongs into monophthongs, not the reverse.
- B. Substituting monophthongs for diphthongs: This is a typical error. Children with hearing loss may simplify diphthongs (e.g., "boy" becomes "bo").
- C. Substituting voiced sounds for voiceless sounds: This is a typical error. Difficulty distinguishing voicing contrasts (e.g., /p/ vs. /b/) is common in children with hearing loss.
- D. Substituting voiceless sounds for voiced sounds: This is also a typical error. Children may struggle to produce voiced sounds correctly, leading to substitutions like /t/ for /d/.

Thus, the correct answer is A. Substituting diphthongs for monophthongs.

Question 40:
Which of the following statements is most appropriate about deaf children with hearing parents who communicate orally?
A. They cannot become part of the Deaf community.
B. Learning spoken language from an early age prevents language development issues.
C. They should learn sign language only after developing mature spoken language.
D. Learning sign language allows them to navigate both hearing and Deaf communities.

Answer: D. Learning sign language allows them to navigate both hearing and Deaf communities.

Explanation:
Let’s evaluate each option:

- A. They cannot become part of the Deaf community: This is incorrect. Deaf children with hearing parents can still become part of the Deaf community, especially if they learn sign language and engage with Deaf culture.
- B. Learning spoken language from an early age prevents language development issues: This is incorrect. Even with early spoken language intervention, deaf children may still experience language delays or difficulties due to limited auditory access.
- C. They should learn sign language only after developing mature spoken language: This is incorrect. Research supports bimodal bilingualism (learning both spoken and signed language simultaneously) as beneficial for language development in deaf children.
- D. Learning sign language allows them to navigate both hearing and Deaf communities: This is correct. Sign language provides access to Deaf culture and community while also supporting communication in the hearing world.

Thus, the most appropriate statement is D. Learning sign language allows them to navigate both hearing and Deaf communities.

Question 41:
A Deaf person repeatedly signs "tell" in different directions to people in the room. What is the most appropriate meaning of this sign?
A. He talks too much.
B. I told him repeatedly.
C. No matter what I say, it’s useless.
D. I told each of them one by one.

Answer: D. I told each of them one by one.

Explanation:
In sign language, directional verbs like "tell" can change meaning based on the direction and repetition of the sign. Let’s evaluate each option:

- A. He talks too much: This does not align with the directional use of the sign "tell."
- B. I told him repeatedly: While repetition is involved, the directional aspect suggests communication with multiple people, not just one.
- C. No matter what I say, it’s useless: This interpretation does not fit the context of directional signing.
- D. I told each of them one by one: This is correct. The directional and repeated use of the sign "tell" indicates that the speaker communicated with multiple individuals sequentially.

Thus, the most appropriate meaning is D. I told each of them one by one.

Question 42:
Which of the following statements about Chinese Sign Language (CSL) grammar is most appropriate?
A. Compared to natural sign language, Deaf students find Chinese Sign Language grammar easier to understand.
B. Chinese Sign Language grammar lacks spatiality, leading to confusion in comprehension.
C. Chinese Sign Language grammar is artificially created and not a conventional language.
D. According to the National Language Development Act, Chinese Sign Language is a national language.

Answer: D. According to the National Language Development Act, Chinese Sign Language is a national language.

Explanation:
Let’s evaluate each option:

- A. Deaf students find Chinese Sign Language grammar easier to understand than natural sign language: This is incorrect. Natural sign languages (e.g., American Sign Language, Taiwanese

Sign Language) are typically easier for Deaf individuals to acquire and understand than manually coded systems like Signed Chinese.

- B. Chinese Sign Language grammar lacks spatiality, leading to confusion: This is incorrect. Sign languages, including Chinese Sign Language, are highly spatial and use space to convey meaning (e.g., indicating subjects, objects, and locations).
- C. Chinese Sign Language grammar is artificially created and not a conventional language: This is incorrect. Chinese Sign Language is a natural language with its own grammar and conventions, not an artificially created system.
- D. According to the National Language Development Act, Chinese Sign Language is a national language: This is correct. In some regions, such as Taiwan, Chinese Sign Language is recognized as a national language under language development laws.

Thus, the most appropriate statement is **D. According to the National Language Development Act, Chinese Sign Language is a national language.

Question 43:
In a village in Bali, Indonesia, a high percentage of the population is Deaf, leading to the development of a sign language called "Kata Kolok." Deaf and hearing children attend school together, and teachers use Kata Kolok for instruction. Which of the following statements about this Deaf village is least appropriate?
A. Kata Kolok is the core of the village's Deaf culture.
B. Hearing children learn Kata Kolok from a young age, so Deaf individuals are not disadvantaged.
C. The hearing villagers do not view Deaf individuals as disabled.
D. The transmission of Kata Kolok primarily relies on school teachers.

Answer: D. The transmission of Kata Kolok primarily relies on school teachers.

Explanation:
Let's evaluate each option:

- A. Kata Kolok is the core of the village's Deaf culture: This is correct. Kata Kolok is central to the Deaf community in the village, serving as a primary means of communication.
- B. Hearing children learn Kata Kolok from a young age, so Deaf individuals are not disadvantaged: This is correct. The widespread use of Kata Kolok by both Deaf and hearing individuals reduces barriers and promotes inclusivity.
- C. The hearing villagers do not view Deaf individuals as disabled: This is correct. In such communities, Deafness is often seen as a natural variation rather than a disability.
- D. The transmission of Kata Kolok primarily relies on school teachers: This is incorrect. The transmission of Kata Kolok is primarily a community-driven process, with children learning the language naturally through interaction with family and peers, not just through formal instruction by teachers.

Thus, the least appropriate statement is D. The transmission of Kata Kolok primarily relies on school teachers.

Question 44:
Xiao Hua was caught shoplifting, and his mentor served as the sign language interpreter during his court hearing. The mentor added exculpatory language during interpretation. Which of the following issues does this scenario reflect?

1. The mentor has a parentalistic attitude.

2. The mentor violated the Registry of Interpreters for the Deaf (RID) Code of Ethics.

3. The constitution mandates reduced sentences for disadvantaged groups.

4. Sign language interpreters must adhere to confidentiality principles.
   A. Only ③
   B. Only ①②
   C. Only ②③
   D. ①②③④

Answer: B. Only ①②

Explanation:
Let's evaluate each option:

- 1. The mentor has a parentalistic attitude: This is correct. By adding exculpatory language, the mentor overstepped their role as an impartial interpreter, reflecting a parentalistic approach.

- 2. The mentor violated the RID Code of Ethics: This is correct. The RID Code of Ethics requires interpreters to remain neutral and accurate, without adding or omitting information.

- 3. The constitution mandates reduced sentences for disadvantaged groups: This is incorrect. While some legal systems may consider mitigating factors, there is no universal constitutional mandate for reduced sentences based on group disadvantage.

- 4. Sign language interpreters must adhere to confidentiality principles: This is incorrect in this context. While confidentiality is important, the primary issue here is the mentor's lack of neutrality and accuracy, not a breach of confidentiality.

Thus, the correct answer is B. Only ①②.

Question 45:
Xiao Ming falsely claimed to be a licensed audiologist and practiced before obtaining his credentials. If verified, what penalty will Xiao Ming face?
A. Prohibition from taking the audiologist exam for 3 years.
B. A fine of NT10,000toNT10,000*toNT*50,000.
C. A fine of NT30,000toNT30,000*toNT*150,000.
D. Lifetime prohibition from taking the audiologist exam.

Answer: C. A fine of NT30,000toNT30,000*toNT*150,000.

Explanation:
Practicing as an audiologist without proper credentials is a serious violation of professional regulations. Let's evaluate each option:

- A. Prohibition from taking the audiologist exam for 3 years: This is incorrect. The penalty for unauthorized practice typically involves fines, not exam restrictions.
- B. A fine of NT10,000toNT10,000*toNT*50,000: This is incorrect. The fine for such a violation is generally higher.
- C. A fine of NT30,000toNT30,000*toNT*150,000: This is correct. Unauthorized practice of audiology typically results in significant fines within this range.
- D. Lifetime prohibition from taking the audiologist exam: This is incorrect. While penalties are severe, a lifetime ban is not a standard punishment for this offense.

Thus, the correct answer is C. A fine of NT30,000toNT30,000*toNT*150,000.

Question 46:
Ziyu is currently 16 years old. According to legal requirements, how many years must the audiology clinic retain his hearing test report?
A. 5 years
B. 7 years
C. 9 years
D. 13 years

Answer: B. 7 years

Explanation:
Medical and audiological records, including hearing test reports, are typically required to be retained for a specific period to ensure continuity of care and legal compliance. In many jurisdictions, the standard retention period for medical records, including audiological records, is 7 years. This ensures that records are available for future reference, legal purposes, or follow-up care.

Thus, the correct answer is B. 7 years.

Question 47:
According to the Audiologist Act, which of the following is not a legally defined scope of practice for audiologists?
A. Non-organic hearing assessment
B. Hearing aid evaluation
C. Aural rehabilitation
D. Selling hearing aids

Answer: D. Selling hearing aids

Explanation:
The scope of practice for audiologists typically includes diagnostic, rehabilitative, and consultative services related to hearing and balance. Let's evaluate each option:

- A. Non-organic hearing assessment: This is within the scope of practice. Audiologists assess both organic and non-organic hearing loss.
- B. Hearing aid evaluation: This is within the scope of practice. Audiologists evaluate, fit, and adjust hearing aids.
- C. Aural rehabilitation: This is within the scope of practice. Audiologists provide rehabilitation services to improve communication for individuals with hearing loss.
- D. Selling hearing aids: This is not typically within the scope of practice. While audiologists may recommend and fit hearing aids, the act of selling hearing aids is often considered a commercial activity and may be regulated separately.

Thus, the correct answer is D. Selling hearing aids.

Question 48:
If an individual holds both audiologist and speech-language pathologist (SLP) licenses, which of the following situations complies with current regulations?
A. They must register in both professions simultaneously and can practice both.
B. They register as an SLP but can still perform hearing aid fittings.
C. They can register in both professions at two locations but can only practice one profession.
D. They can register at only one location but may lend their other license to someone else.

Answer: C. They can register in both professions at two locations but can only practice one profession.

Explanation:
Regulations for dual-licensed professionals typically aim to prevent conflicts of interest and ensure that individuals practice within their registered scope. Let's evaluate each option:

- A. Register in both professions and practice both: This is incorrect. Most regulations require professionals to practice only within their registered scope at a given time.
- B. Register as an SLP but perform hearing aid fittings: This is incorrect. Hearing aid fitting is typically within the scope of audiologists, not SLPs, unless explicitly permitted by regulations.
- C. Register in both professions at two locations but practice only one profession: This is correct. Dual-licensed professionals can register in both fields but must practice only one profession at a time to avoid conflicts and ensure compliance.
- D. Register at one location and lend the other license: This is incorrect. Lending or transferring a professional license to another person is illegal and unethical.

Thus, the correct answer is C. They can register in both professions at two locations but can only practice one profession.

Question 49:
Audiologist Wang needs to collect patient data for research and statistical analysis. Before collecting the data, which of the following committees must review and approve the study?
A. Professional Ethics Committee of the Audiology and Speech-Language Association
B. Medical Rights Promotion Committee
C. Ethics Review Committee of the National Science and Technology Council
D. Institutional Review Board (IRB) for Human Subjects Research

Answer: D. Institutional Review Board (IRB) for Human Subjects Research

Explanation:
Research involving human subjects, especially in healthcare, requires ethical oversight to ensure the protection of participants' rights and well-being. Let’s evaluate each option:

- A. Professional Ethics Committee of the Audiology and Speech-Language Association: This committee may provide ethical guidelines but does not typically conduct formal reviews of research protocols.
- B. Medical Rights Promotion Committee: This committee focuses on patient rights and advocacy, not research ethics review.
- C. Ethics Review Committee of the National Science and Technology Council: While this committee may oversee research funding, it does not typically handle the ethical review of individual studies involving human subjects.
- D. Institutional Review Board (IRB) for Human Subjects Research: This is correct. The IRB is responsible for reviewing and approving research involving human subjects to ensure ethical standards are met.

Thus, the correct answer is D. Institutional Review Board (IRB) for Human Subjects Research.

Question 50:
According to the Audiologist Act, audiologists must join the local audiologist association to practice. If the association refuses to admit an audiologist, what is the fine imposed on the association by the civil organization authority?
A. NT10,000toNT10,000*toNT*50,000
B. NT20,000toNT20,000*toNT*100,000
C. NT30,000toNT30,000*toNT*150,000
D. NT40,000toNT40,000*toNT*200,000

Answer: C. NT30,000toNT30,000*toNT*150,000

Explanation:
The Audiologist Act mandates that audiologists join their local professional association to practice. If an association unlawfully refuses membership, it can be penalized by the civil organization authority. The

standard fine for such a violation is typically NT30,000toNT30,000*toNT*150,000, as specified in the regulations.

Thus, the correct answer is C. NT30,000toNT30,000*toNT*150,000.
