# Supplementary material for: Comparison of ChatGPT and DeepSeek on a Standardized Audiologist Qualification Examination in Chinese: Observational Study
Source: JMIR Form Res. 2025 Nov 28;9:e79534. doi: 10.2196/79534 (PMC12701348; doi:10.2196/79534)
Supplement: Multimedia Appendix 3 [file formative_v9i1e79534_app3.pdf]

測驗題標準答案更正

考試名稱： 113年第二次專技人員高等考試營養師、護理師、社會工作師考試、113年專技人員高等考試心理師、法醫師、語言治療師、聽力師、牙體技術師、公共衛生師考試、高等暨普通考試驗光人員考試

類科名稱： 聽力師

科目名稱： 基礎聽力科學

單選題數：50題　　單選每題配分：2.00分

複選題數：　　複選每題配分：

標準答案：答案標註#者，表該題有更正答案，其更正內容詳見備註。

題號	第1題	第2題	第3題	第4題	第5題	第6題	第7題	第8題	第9題	第10題
答案	B	A	#	B	B	D	D	A	A	D

題號	第11題	第12題	第13題	第14題	第15題	第16題	第17題	第18題	第19題	第20題
答案	B	A	B	B	D	D	C	A	B	A

題號	第21題	第22題	第23題	第24題	第25題	第26題	第27題	第28題	第29題	第30題
答案	B	D	A	B	C	A	C	C	B	B

題號	第31題	第32題	第33題	第34題	第35題	第36題	第37題	第38題	第39題	第40題
答案	C	D	B	B	D	A	C	B	D	B

題號	第41題	第42題	第43題	第44題	第45題	第46題	第47題	第48題	第49題	第50題
答案	D	A	C	#	A	C	C	A	A	B

題號	第51題	第52題	第53題	第54題	第55題	第56題	第57題	第58題	第59題	第60題
答案										

題號	第61題	第62題	第63題	第64題	第65題	第66題	第67題	第68題	第69題	第70題
答案										

題號	第71題	第72題	第73題	第74題	第75題	第76題	第77題	第78題	第79題	第80題
答案										

題號	第81題	第82題	第83題	第84題	第85題	第86題	第87題	第88題	第89題	第90題
答案										

題號	第91題	第92題	第93題	第94題	第95題	第96題	第97題	第98題	第99題	第100題
答案										

備　　註： 第3題答Ａ或Ｂ或AB者均給分，第44題答Ｂ或Ｃ或BC者均給分。

測驗式試題標準答案

考試名稱： 113年第二次專技人員高等考試營養師、護理師、社會工作師考試、113年專技人員高等考試心理師、法醫師、語言治療師、聽力師、牙體技術師、公共衛生師考試、高等暨普通考試驗光人員考試

類科名稱： 聽力師

科目名稱： 行為聽力學（試題代號：2106）

單選題數：50題　　單選每題配分：2.00分

複選題數：　　複選每題配分：

標準答案：

題號	第1題	第2題	第3題	第4題	第5題	第6題	第7題	第8題	第9題	第10題
答案	D	D	D	D	D	C	D	D	D	A
題號	第11題	第12題	第13題	第14題	第15題	第16題	第17題	第18題	第19題	第20題
答案	D	D	C	B	D	B	B	A	B	B
題號	第21題	第22題	第23題	第24題	第25題	第26題	第27題	第28題	第29題	第30題
答案	B	C	D	B	B	C	C	D	A	B
題號	第31題	第32題	第33題	第34題	第35題	第36題	第37題	第38題	第39題	第40題
答案	A	D	C	D	D	C	D	D	D	A
題號	第41題	第42題	第43題	第44題	第45題	第46題	第47題	第48題	第49題	第50題
答案	C	A	B	C	D	C	B	A	C	C
題號	第51題	第52題	第53題	第54題	第55題	第56題	第57題	第58題	第59題	第60題
答案										
題號	第61題	第62題	第63題	第64題	第65題	第66題	第67題	第68題	第69題	第70題
答案										
題號	第71題	第72題	第73題	第74題	第75題	第76題	第77題	第78題	第79題	第80題
答案										
題號	第81題	第82題	第83題	第84題	第85題	第86題	第87題	第88題	第89題	第90題
答案										
題號	第91題	第92題	第93題	第94題	第95題	第96題	第97題	第98題	第99題	第100題
答案										

備　　註：

測驗式試題標準答案

考試名稱：113年第二次專技人員高等考試營養師、護理師、社會工作師考試、113年專技人員高等考試心理師、法醫師、語言治療師、聽力師、牙體技術師、公共衛生師考試、高等暨普通考試驗光人員考試

類科名稱：聽力師

科目名稱：電生理聽力學（試題代號：3106）

單選題數：50題　　單選每題配分：2.00分

複選題數：　　複選每題配分：

標準答案：

題號	第1題	第2題	第3題	第4題	第5題	第6題	第7題	第8題	第9題	第10題
答案	A	A	B	C	C	D	A	D	C	B

題號	第11題	第12題	第13題	第14題	第15題	第16題	第17題	第18題	第19題	第20題
答案	C	D	A	D	D	A	D	B	C	C

題號	第21題	第22題	第23題	第24題	第25題	第26題	第27題	第28題	第29題	第30題
答案	B	B	D	C	D	B	D	C	B	B

題號	第31題	第32題	第33題	第34題	第35題	第36題	第37題	第38題	第39題	第40題
答案	C	C	D	A	A	B	C	A	B	D

題號	第41題	第42題	第43題	第44題	第45題	第46題	第47題	第48題	第49題	第50題
答案	D	B	D	A	D	A	D	C	D	D

題號	第51題	第52題	第53題	第54題	第55題	第56題	第57題	第58題	第59題	第60題
答案										

題號	第61題	第62題	第63題	第64題	第65題	第66題	第67題	第68題	第69題	第70題
答案										

題號	第71題	第72題	第73題	第74題	第75題	第76題	第77題	第78題	第79題	第80題
答案										

題號	第81題	第82題	第83題	第84題	第85題	第86題	第87題	第88題	第89題	第90題
答案										

題號	第91題	第92題	第93題	第94題	第95題	第96題	第97題	第98題	第99題	第100題
答案										

備　　註：

測驗題標準答案更正

考試名稱： 113年第二次專技人員高等考試營養師、護理師、社會工作師考試、113年專技人員高等考試心理師、法醫師、語言治療師、聽力師、牙體技術師、公共衛生師考試、高等暨普通考試驗光人員考試

類科名稱： 聽力師

科目名稱： 聽覺輔具原理與實務學

單選題數：50題　　單選每題配分：2.00分

複選題數：　　複選每題配分：

標準答案：答案標註#者，表該題有更正答案，其更正內容詳見備註。

題號	第1題	第2題	第3題	第4題	第5題	第6題	第7題	第8題	第9題	第10題
答案	D	D	A	B	D	A	A	C	C	A

題號	第11題	第12題	第13題	第14題	第15題	第16題	第17題	第18題	第19題	第20題
答案	B	A	B	D	D	D	B	A	C	A

題號	第21題	第22題	第23題	第24題	第25題	第26題	第27題	第28題	第29題	第30題
答案	A	A	B	B	A	#	B	A	C	A

題號	第31題	第32題	第33題	第34題	第35題	第36題	第37題	第38題	第39題	第40題
答案	B	B	D	B	C	A	B	C	C	A

題號	第41題	第42題	第43題	第44題	第45題	第46題	第47題	第48題	第49題	第50題
答案	D	A	A	B	A	C	C	A	B	A

題號	第51題	第52題	第53題	第54題	第55題	第56題	第57題	第58題	第59題	第60題
答案										

題號	第61題	第62題	第63題	第64題	第65題	第66題	第67題	第68題	第69題	第70題
答案										

題號	第71題	第72題	第73題	第74題	第75題	第76題	第77題	第78題	第79題	第80題
答案										

題號	第81題	第82題	第83題	第84題	第85題	第86題	第87題	第88題	第89題	第90題
答案										

題號	第91題	第92題	第93題	第94題	第95題	第96題	第97題	第98題	第99題	第100題
答案										

備　　註： 第26題答Ａ或Ｂ或AB者均給分。

測驗式試題標準答案

考試名稱：113年第二次專技人員高等考試營養師、護理師、社會工作師考試、113年專技人員高等考試心理師、法醫師、語言治療師、聽力師、牙體技術師、公共衛生師考試、高等暨普通考試驗光人員考試

類科名稱：聽力師

科目名稱：聽覺與平衡系統之創健與復健學（試題代號：5106）

單選題數：50題　　單選每題配分：2.00分

複選題數：　　複選每題配分：

標準答案：

題號	第1題	第2題	第3題	第4題	第5題	第6題	第7題	第8題	第9題	第10題
答案	C	D	D	C	C	A	A	C	B	C

題號	第11題	第12題	第13題	第14題	第15題	第16題	第17題	第18題	第19題	第20題
答案	A	C	B	D	D	A	D	A	A	A

題號	第21題	第22題	第23題	第24題	第25題	第26題	第27題	第28題	第29題	第30題
答案	C	C	C	A	C	C	C	D	B	B

題號	第31題	第32題	第33題	第34題	第35題	第36題	第37題	第38題	第39題	第40題
答案	B	A	B	B	B	D	C	D	D	B

題號	第41題	第42題	第43題	第44題	第45題	第46題	第47題	第48題	第49題	第50題
答案	C	C	B	C	B	A	B	C	A	B

題號	第51題	第52題	第53題	第54題	第55題	第56題	第57題	第58題	第59題	第60題
答案										

題號	第61題	第62題	第63題	第64題	第65題	第66題	第67題	第68題	第69題	第70題
答案										

題號	第71題	第72題	第73題	第74題	第75題	第76題	第77題	第78題	第79題	第80題
答案										

題號	第81題	第82題	第83題	第84題	第85題	第86題	第87題	第88題	第89題	第90題
答案										

題號	第91題	第92題	第93題	第94題	第95題	第96題	第97題	第98題	第99題	第100題
答案										

備　　註：

測驗式試題標準答案

考試名稱：113年第二次專技人員高等考試營養師、護理師、社會工作師考試、113年專技人員高等考試心理師、法醫師、語言治療師、聽力師、牙體技術師、公共衛生師考試、高等暨普通考試驗光人員考試

類科名稱：聽力師

科目名稱：聽語溝通障礙學(包括專業倫理)（試題代號：6106）

單選題數：50題　　單選每題配分：2.00分

複選題數：　　複選每題配分：

標準答案：

題號	第1題	第2題	第3題	第4題	第5題	第6題	第7題	第8題	第9題	第10題
答案	C	C	A	A	B	C	C	D	C	A

題號	第11題	第12題	第13題	第14題	第15題	第16題	第17題	第18題	第19題	第20題
答案	D	B	B	D	D	D	C	D	D	C

題號	第21題	第22題	第23題	第24題	第25題	第26題	第27題	第28題	第29題	第30題
答案	A	A	A	C	D	C	A	B	C	C

題號	第31題	第32題	第33題	第34題	第35題	第36題	第37題	第38題	第39題	第40題
答案	A	D	D	D	B	B	D	A	D	D

題號	第41題	第42題	第43題	第44題	第45題	第46題	第47題	第48題	第49題	第50題
答案	D	C	D	B	C	C	D	B	D	A

題號	第51題	第52題	第53題	第54題	第55題	第56題	第57題	第58題	第59題	第60題
答案										

題號	第61題	第62題	第63題	第64題	第65題	第66題	第67題	第68題	第69題	第70題
答案										

題號	第71題	第72題	第73題	第74題	第75題	第76題	第77題	第78題	第79題	第80題
答案										

題號	第81題	第82題	第83題	第84題	第85題	第86題	第87題	第88題	第89題	第90題
答案										

題號	第91題	第92題	第93題	第94題	第95題	第96題	第97題	第98題	第99題	第100題
答案										

備　　註：
